# Niche evolution in a northern temperate tree lineage: biogeographical legacies in cork oaks (*Quercus* section *Cerris*)

**DOI:** 10.1093/aob/mcad032

**Published:** 2023-02-20

**Authors:** Thomas Denk, Guido W Grimm, Andrew L Hipp, Johannes M Bouchal, Ernst-Detlef Schulze, Marco C Simeone

**Affiliations:** Department of Palaeobiology, Swedish Museum of Natural History, 10405 Stockholm, Sweden; Unaffiliated, 45100 Orléans, France; The Morton Arboretum, Lisle, IL 60532-1293, USA; Department of Botany and Biodiversity Research, University of Vienna, 1030 Vienna, Austria; Max-Planck Institute for Biogeochemistry, 07701 Jena, Germany; Department of Agricultural and Forestry Sciences, University of Tuscia, 01100 Viterbo, Italy

**Keywords:** *Quercus*, next-generation sequencing, fossilized birth–death model, fossil, niche evolution, biogeography, leaf lifespan

## Abstract

**Background and Aims:**

Cork oaks (*Quercus* section *Cerris*) comprise 15 extant species in Eurasia. Despite being a small clade, they display a range of leaf morphologies comparable to the largest sections (>100 spp.) in *Quercus*. Their fossil record extends back to the Eocene. Here, we explore how cork oaks achieved their modern ranges and how legacy effects might explain niche evolution in modern species of section *Cerris* and its sister section *Ilex*, the holly oaks.

**Methods:**

We inferred a dated phylogeny for cork and holly oaks using a reduced-representation next-generation sequencing method, restriction site-associated DNA sequencing (RAD-seq), and used *D-*statistics to investigate gene flow hypotheses. We estimated divergence times using a fossilized birth–death model calibrated with 47 fossils. We used Köppen profiles, selected bioclimatic parameters and forest biomes occupied by modern species to infer ancestral climatic and biotic niches.

**Key Results:**

East Asian and Western Eurasian cork oaks diverged initially in the Eocene. Subsequently, four Western Eurasian lineages (subsections) differentiated during the Oligocene and Miocene. Evolution of leaf size, form and texture was correlated, in part, with multiple transitions from ancestral humid temperate climates to mediterranean, arid and continental climates. Distantly related but ecologically similar species converged on similar leaf traits in the process.

**Conclusions:**

Originating in temperate (frost-free) biomes, Eocene to Oligocene ranges of the primarily deciduous cork oaks were restricted to higher latitudes (Siberia to north of Paratethys). Members of the evergreen holly oaks (section *Ilex*) also originated in temperate biomes but migrated southwards and south-westwards into then-(sub)tropical southern China and south-eastern Tibet during the Eocene, then westwards along existing pre-Himalayan mountain ranges. Divergent biogeographical histories and deep-time phylogenetic legacies (in cold and drought tolerance, nutrient storage and fire resistance) thus account for the modern species mosaic of Western Eurasian oak communities, which are composed of oaks belonging to four sections.

## INTRODUCTION

The genus *Quercus* L. (oak trees) is one of the most economically and ecologically important woody angiosperm genera in the Northern Hemisphere. Oaks comprise ~425 species and occur in a wide range of habitats, from dry woodlands to swamp forests and from lowlands to elevations ≤4500 m a.s.l. (Camus, 1936–1954; Denk *et al.*, 2017*a*). They are a dominant component of the northern temperate forests (Martinetto *et al.*, 2020). Traditionally, the taxonomy of this genus has been based on key morphological characters, and different classification schemes have been proposed over the centuries (e.g. Ørsted, 1871; Trelease, 1924; Schwarz, 1936; Camus, 1936–1954; Menitsky, 1984; Nixon, 1993; for differences in these schemes, see Denk and Grimm, 2010; Denk *et al.*, 2017*a*). In recent years, a number of morphological (Solomon, 1983*a*, 1983*b*; Denk and Grimm, 2009; Denk and Tekleva, 2014) and molecular (Manos *et al.*, 2001, 2008; Oh and Manos, 2008; Denk and Grimm, 2010; Hipp *et al*., 2014, 2018, 2020; Hubert *et al.*, 2014; Cavender-Bares *et al.*, 2015; Simeone *et al*., 2016, 2018; McVay *et al*., 2017*a*, 2017*b*; Pham *et al.*, 2017; Vitelli *et al.*, 2017; Deng *et al.*, 2018; Ortego *et al.*, 2018; Cavender-Bares, 2019; Jiang *et al.*, 2019; Crowl *et al.*, 2020) studies have provided a robust phylogenetic framework along with a revised subgeneric and sectional classification. Together, these studies provide a framework to place the extensive fossil record of the genus in a phylogenetic context (e.g. Bouchal *et al.*, 2014; Grímsson *et al.*, 2015, 2016).

Leaf phenology and climatic niche have evolved in concert across woody angiosperms (Woodward *et al.*, 2004; Hawkins *et al.*, 2014; Zanne *et al.*, 2014; Edwards *et al.*, 2017; Hipp *et al.*, 2018). A broad phylogenetic comparative study has shown that leaf phenology can evolve as a response to a change in environment (‘climate first’) or arise first and predispose lineages to freezing tolerance (‘trait first’), with the ‘climate first’ pathway being more frequent, particularly in deciduous woody plant lineages (Zanne *et al.*, 2014). Understanding the detailed history of these patterns of evolution requires dissection of individual clades. In *Viburnum*, for example, deciduousness evolved *in situ* as populations were subjected to gradual cooling (Edwards *et al.*, 2017). In the American (‘New World’) oak clade (*Quercus* subgenus *Quercus*), one of two major clades within *Quercus*, >20 independent shifts from deciduous to evergreen leaf phenology in Mexican white and red oaks (*Quercus* sections *Quercus* and *Lobatae*) are associated with climatic and edaphic shifts, and the evergreen habit is inferred to have evolved in response to decreases in temperature seasonality and decreases in both winter and summer temperature extremes (Hipp *et al.*, 2018). In contrast, in the Eurasian (‘Old World’) oak clade (subgenus *Cerris*), leaf phenology is stable within sections. Both the deciduous cork oaks (section *Cerris*) and the evergreen holly oaks (section *Ilex*) show considerable distributional overlap with each other and subgenus *Quercus*. Moreover, section *Cerris* is the only oak section to reach its highest species richness and absolute phylogenetic diversity in Western Eurasia.

Here, we investigate these two closely related sections of the Eurasian (‘Old World’) oak clade. We test the trait first and climate first pathways and provide detailed insights into two sister lineages of oaks. We use a reduced-representation next-generation sequencing method, restriction-site associated DNA sequencing (RAD-seq; Ree and Hipp, 2015), to infer a fully resolved species phylogeny of the Eurasian cork oaks, including 25 ingroup specimens representing 14 of the 15 species (all except for the narrow-endemic *Quercus euboica*). We use 47 fossil taxa to date the phylogeny using a fossilized birth–death (FBD) approach and to reconstruct the biogeographical history of the cork oaks. Based on habitats, climatic preferences and leaf morphologies of modern and fossil cork oaks, we investigate the history of climatic niche evolution and the correlation of niche shifts with leaf evolution. Furthermore, we compare the biogeographical histories of sections *Cerris* and *Ilex* to explain modern ranges and niche occupancy of these sections across Eurasia. Finally, we discuss our results against the background of palaeogeographical and tectonic changes in Eurasia during the past 40 million years and diversification patterns established from previous studies of the nuclear and plastid genomes.

## MATERIALS AND METHODS

### Sampling

Samples from 62 individuals included in the study by Hipp *et al.* (2020) were re-analysed for this study. Sampling details, vouchers, National Center for Biotechnology Information (NCBI) short-read archive (SRA) project and accession numbers are provided in the Supplementary data (Table S1). Twenty-five samples covered all species of section *Cerris* except for *Q. euboica* (Papaioannou) K.I.Chr., for which we were unable to obtain fresh material with sufficient DNA yield. To represent the sister section *Ilex*, we included all its Western Eurasian species: four Mediterranean species (five if *Quercus calliprinos* Webb is considered a separate species) plus the western Himalayan–Hindukush *Quercus baloot* Griff. (Clade VI in the study by Jiang *et al.*, 2019; see also Simeone *et al*., 2016, 2018). Additional East Asian *Ilex* species were selected to represent the major lineages within this section: five species, including *Quercus floribunda* Lindl. ex A.Camus, for the Himalayan clade carrying *Ilex*-specific plastomes (cf. Yan *et al.*, 2019; Hipp *et al.*, 2020; Clade IV+V in the study by Jiang *et al.*, 2019); and three species representing the East Asian clade: the (central) Chinese *Quercus baronii* Skan, morphologically similar to *Cerris* oaks but with a unique plastome; *Quercus dolicholepis* A.Camus, a montane central Chinese species; and *Quercus phillyreoides* A.Gray, a widespread north-eastern Asian subtropical to temperate species. The latter two have *Cerris-*similar plastid signatures (Simeone *et al.*, 2016; Clade II in the study by Jiang *et al.*, 2019). Five species of section *Cyclobalanopsis* (Oerst.) Benth. & Hook.f. represented the third lineage within subgenus *Cerris*, resolved as early diverged sister lineage of sections *Cerris + Ilex* (Hipp *et al.*, 2020). As a further outgroup, we included 15 samples covering Western Eurasian members of subgenus *Quercus* (sections *Quercus* and *Ponticae* Stef.), one eastern North American red oak (*Quercus coccinea* Münchh., section *Lobatae* Loudon), and the western North American relict genus *Notholithocarpus* Manos, Cannon & S.H.Oh, the most probable closest living relative of oaks (Hipp *et al.*, 2020; Zhou *et al.*, 2022).

### RAD-seq data generation and clustering

Next-generation sequencing libraries were prepared at Floragenex (Portland, OR, USA) following the methods of Baird *et al.* (2008) with PstI, barcoded by individual, and sequenced in 150-bp single-end reactions on an Illumina HiSeq 2000, 2500 or 4000 at the University of Oregon Genomic Facility; past analyses (Hipp *et al*., 2014, 2018, 2020) demonstrate that phylogenetic results in this sample set are not obviously influenced by variation in the sequencing platform. FASTQ files were demultiplexed and filtered to remove sequences with more than five bases of quality score <20 and assembled into loci for phylogenetic analysis using ipyrad v.0.7.24 (Eaton, 2014) at 85 % sequence similarity. Consensus sequences for each individual for each locus were then clustered across individuals, initially retaining loci present in at least four individuals and possessing ≤20 single-nucleotide polymorphisms and eight indels across individuals. Data were imported into R using the RADami package (Hipp *et al.*, 2014) to filter loci for analysis into three datasets, containing a minimum of 15, 20 or 25 individuals per locus (m15, m20 and m25, respectively). Loci were concatenated into a single data partition for maximum likelihood (ML) and Bayesian phylogenetic analyses, and locus identities were preserved for *D-*statistic analyses of possible introgression (see below).

### Maximum likelihood tree inference and bootstrapping

Initial phylogenetic tree inference and bootstrap analyses were performed under ML with RAxML v.8 (Stamatakis, 2014). Analysis was conducted using the general time-reversible model with rate variation (GTR+Γ; Rodriguez *et al.*, 1990), and 200 fast non-parametric bootstraps to estimate branch support. To assess the possible role of introgressive hybridization in the clade, we used Patterson’s *D*-statistic test (Durand *et al.*, 2011) as implemented in ipyrad (Eaton and Overcast, 2020). Set-up details and full results are provided in Supplementary Data S1. Two primary hypotheses were tested: the hybrid origins of *Quercus afares* (Mir *et al.*, 2009) and of *Quercus crenata* (Schwarz, 1936; Pignatti, 1982). In addition, we performed follow-up tests for admixture between *Quercus cerris* and *Q. afares*; among subsections *Aegilops*, *Suber* and *Libani*; and between *Quercus ilex* and *Q. suber*, representing increasingly large phylogenetic distances. Supplementary files, data matrices and analysis scripts are archived at https://github.com/andrew-hipp/cerris-fbd (v.1.0-1; https://doi.org/10.5281/zenodo.7547523).

### Fossils

We compiled a set of 47 fossils as age distribution priors for the fossilized birth–death model (see next subsection) and mapped the spatiotemporal distribution of oaks, with a focus on section *Cerris*. Of these fossil occurrences, 24 localities are dated by radiometric dating and/or by vertebrate fossils; for two localities, ages are constrained using palaeomagnetic data; and the remaining localities are dated by lithostratigraphic correlation or dinocyst stratigraphy (Supplementary data Tables S2, S3). Four fossil taxa are represented by fruit/cup remains, seven by pollen, and 36 by leaves. Pollen taxa were assigned to sections based on synapomorphies shared with particular lineages (Denk and Grimm, 2009; Denk *et al.*, 2017*a*), whereas fruit and leaf fossils were chosen based on (sub)section-diagnostic traits. The full list of selected fossil taxa, with their taxonomic assignments, information on the plant organ, the branch to which they are assigned, geographical origin, ages, constraints and relevant references (if not occurring in the main text) are provided in the Supplementary data (Table S2). See Supplementary Data S1 and S2 for notes on stratigraphic units and mapping of fossils mapped onto palaeoglobes (Scotese, 2013*a*, 2013*b*, 2013*c*, 2013*d*, 2013*e*; early Eocene to Last Glacial Maximum).

### Fossilized birth–death dating analyses

For FBD analyses, the RAD-seq matrix was reduced to 29 tips, with a single tip per species within both section *Cerris* and section *Ilex*, with the exception of *Quercus cerris*, for which two individuals were kept that did not group together in any analyses and might represent pseudo-cryptic or cryptic species. Loci were retained if they were present in at least ten individuals. A NEXUS file was exported using the RADami package, including 47 additional lines of undetermined positions (coded as ‘?’), one per fossil included in the FBD analyses. The FBD analyses were conducted in BEAST2 (Bouckaert *et al.*, 2014). Markov chain Monte Carlo (MCMC) runs of 50 million generations each were run from ten independent random starting points on each of three random draws from the uniform distribution of the fossil age ranges. Analyses were conducted using a nucleotide substitution model that allows for rate variation and invariant sites (Γ+I), with the shape parameter (α) and proportion of invariant positions estimated, and four gamma categories. The relaxed log-normal clock was used, with the clock rate estimated. Details of the analysis are in the Supplementary Methods (Supplementary Data S1). Scripts and RAD-seq data matrices are archived in the code repository for this paper (release v.1.0-1: https://github.com/andrew-hipp/cerris-fbd; https://doi.org/10.5281/zenodo.7547523).

### Köppen–Geiger climate types, WorldClim climate data and major forest biomes

We used grid-weighted ‘Köppen signatures’ (Denk *et al.*, 2013; Grímsson *et al.*, 2018; Bouchal *et al.*, 2020), henceforth ‘Köppen profiles’, to summarize the climatic niches occupied by species of *Cerris* and to investigate climatic niche evolution within and among subsections of *Cerris* (Table 1; Supplementary Data S3). A Köppen profile reflects the proportional coverage of the various Köppen–Geiger climate zones (cf. Kottek *et al.*, 2006; Peel *et al.*, 2007) by a modern species based on gridded distribution data. To simplify interpretation, Köppen profiles are summarized into five climatic niches (see the subsection *Maximum likelihood reconstructions of major climatic niches and main biomes*); additional details on interpretation and coding are in the Supplementary Methods (Supplementary Data S1).

**Table 1.** Currently recognized members of *Quercus* section *Cerris* and information about geographical distribution, representative Köppen–Geiger climate and biome types and some morphological characteristics

Species	Region	Köppen–Geiger climate types	Representative climate types ^a^	Environmental stress	Major biogeographical region	Terrestrial biome	Elevation	Cup diameter	Leaf size	Leaf type
		(cf. Kottek *et al.*, 2006; Peel *et al.*, 2007 for definition of climate types)			(Olson *et al.*, 2001)	(Olson *et al.*, 2001)	(m a.s.l.)	(cm, including bracts)	(length, cm)	(cf. Fig. 5)
*Q. acutissima*	E Asia	(*A*), *Cw*, *Cf*, *Dw*, *Df*, (*BSk*)	***Cfa***, *Cwa*	Humid = no stress [Snow, Continentality]	Palaearctic, Indo-Malay	TBMF, TSMBF	100–2200	1.2–4.2	8–19	**0**
*Q. chenii*	E Asia	*Cf*	***Cfa***	Humid = no stress	Palaearctic, Indo-Malay	TBMF, TSMBF	<600	0.8–1.7	7–12	**0**
*Q. variabilis*	E Asia	*Cw*, *Cf*, *Dw*, *Df*, *BSk*	***Cfa***, *Cwa*, *Cwb*	Humid = no stress [Snow, Continentality]	Palaearctic, Indo-Malay	TBMF, TSMBF	<3000	1.5–4	8–15(–20)	**0**
*Q. crenata*	W Eurasia	*Cf*, *Cs*	***Csa***, *Csb*, *Cfb*	Drought [Humid = no stress]	Palaearctic	TBMF	<1000	2.5	4–9	**1**
*Q. suber*	W Eurasia	*Cf*, *Cs*, (*BSk*)	***Csa***, *Csb*	Drought, Fire	Palaearctic	MFWS	<800	2–2.5	3–7	**2**
*Q. brantii*	W Eurasia	*Cs*, *Ds*, *BSk*	***Csa***, *Dsa*	Drought [Snow, Continentality]^b^	Palaearctic	MFWS	350–1700	1.5–4	6–10(–13)	**1**
*Q. ithaburensis*	W Eurasia	*Cs*, (*BSk*), (*BWk*)	***Csa***, *Csb*	Drought [Continentality]^b^	Palaearctic	MFWS	50–500	3–5	4–9	**1**
*Q. macrolepis* [incl. *Q. vallonea*]	W Eurasia	(*Cf*), *Cs*	***Csa***, *Csb*	Drought	Palaearctic	MFWS	50-1700	2.5–6.5	5–9	**3**
*Q. afares*	W Eurasia	*Cs*	***Csa***, *Csb*	Drought	Palaearctic	MFWS	900–1900	1.8–3.5	7–14	**1**
*Q. libani*	W Eurasia	*Cs*, *Ds*, (*BSk*)	***Csa***, *Csb*, *Dsa*, *Dsb*	Drought [Snow, Continentality]^b^	Palaearctic	MFWS	700–2000	2–3.5	7–12	**0**
*Q. trojana*	W Eurasia	(*Cf*), *Cs*, (*Ds*)	***Csa***, *Csb*	Drought [Snow]	Palaearctic	TBMF, MFWS	300–1800	2.2–3	3–8(–10)	**1**
*Q. euboica*	W Eurasia	*Cs*	***Csa***	Drought	Palaearctic	MFWS	100–500	Not known	5–9(–11)	**1**
*Q. castaneifolia*	W Eurasia	*Cs*, *Ds*^c^	***Csa*** ^c^	Humid = no stress^c^ [Snow]	Palaearctic	TBMF	<2400	1.5–2	10–20	**1**
*Q. look*	W Eurasia	*Cs*	***Csa***	Drought	Palaearctic	MFWS	1000–2000	2–3.3	5–7.5	**3**
*Q. cerris*	W Eurasia	*Cf*, *Cs*, *Df*, (*Ds*)	***Cfb***, ***Csa***, *Csb*	Humid = no stress, Drought [Snow]	Palaearctic	TBMF, MFWS	<1900	2.5–3.5	5.5–14(–20)	**4**

Distribution data are from GBIF (https://www.gbif.org/) and Fang *et al.* (2009).

^a^ ≥10 % of occurrences (unique grid cells); dominating occurrences in bold. For complete occurrence data, See Supplementary Data S3. ^b^ Hot dry summers plus very cold winters; (*A*) ≤ 3 %. ^c^ Transitional to fully humid *Cf* climate.

Köppen climate types: *A*, equatorial climate; *BSk*, cold steppe climates; *BWk*, cold desert climate; *C*, warm temperate climates (*f*, fully humid; *s*, summer-dry; *w*, winter-dry with *a*, hot, *b*, warm summers); *D*, snow climates (as for *C*). Biome abbreviations: MFWS, Mediterranean Forests, Woodlands and Scrub; TBMF, Temperate Broadleaf and Mixed Forests; TSMBF, Tropical and Subtropical Moist Broadleaf Forests.

Data on vertical distribution and quantitative morphological traits are from the following: le Hardÿ de Beaulieu & Lamant (2010); Hedge & Yaltirik (1982); Hegi (1981); Hélardot (1987 onwards); Huang *et al.* (1999); Kotschy (1862); Menitsky (2005); Mucina and Dimopoulos (2000); Stephan *et al.* (2018).

Modern species distributions were connected to fossil distributions by using georeferenced occurrence data for each species, downloaded from the GBIF database (www.gbif.org; Supplementary Data S3). Each dataset was checked for natural distribution outliers (e.g. specimens from botanical gardens). Published chorological data were used to detect these outliers (e.g. Browicz and Zieliński, 1982; Fang *et al.*, 2009; San-Miguel-Ayanz *et al*., 2016; Caudullo *et al.*, 2017). The cleaned georeferenced occurrence data were then plotted onto a 5 arc min Köppen–Geiger grid (1986–2010 data; Rubel *et al.*, 2017) to establish Köppen profiles for all species of section *Cerris*; and on major terrestrial biome maps (Olson *et al.*, 2001; Supplementary Data S3) to assess the forest biome preferences of species.

### Characterization of modern leaf types

Leaf morphologies of modern section *Cerris* species were characterized using leaf texture, lamina size, tooth type and other traits (Table 1; Supplementary Data S1). The overall morphological differentiation patterns were visualized using a neighbour net (Bryant and Moulton, 2004), a planar (meta-)phylogenetic network (cf. Denk and Grimm, 2009). Twelve traits were scored as a categorical character matrix (11 binary and 1 ternary character; matrix *LeafMorphs* in Supplementary Data S1: Data File S1-1) and used to infer simple (Hamming) pairwise distances and to reconstruct character evolution on the dated topology under the *Mk1* model in Mesquite v.2.75 (Maddison and Maddison, 2011; Supplementary Data S1).

### Maximum likelihood reconstructions of major climatic niches and main biomes

Based on the quantitative assessment of biome and climate zone preferences of the modern-day species, we binned extant and fossil species into five basic categories, accounting either for biome or climate zone preferences (Table 1; Supplementary Data S3). Our generalization and categorization make use of the terminology and concepts introduced by Schroeder (1998; cf. Denk *et al.*, 2013) and allow: (1) direct comparison of biome and climate zones preferences, which are commonly correlated but not synonymous; and (2) relation of quantitative modern-day categorization qualitatively to our fossil taxon set. Towards that end, we first defined the putative covered biomes for each fossil taxon of section *Cerris* (columns *Biome/major Köppen climate type* in Supplementary data Table S2). Assignment of fossils to climates is described in the Supplementary Methods (see also Supplementary data Table S2; Supplementary Data S1: Data File S1-1). Explicit connection of biomes to Köppen profiles is explained in the Supplemental Methods (Supplementary Data S1). In brief, the biomes and general climate preferences reconstructed are as follows:

**0–Moist**-**Subtropical:** associated exclusively with the Tropical and Subtropical Moist Broadleaf Forests biome. In the case of modern-day *Cerris* oaks, species are associated almost exclusively with summer-moist climates with hot summers. The only modern species with an accordingly characteristic climatic niche is the East Asian *Quercus chenii*, firmly restricted to the *Cfa* climate of central-eastern China.
**1–Meridio-Nemoral:** associated with the ecotone between Tropical and Subtropical Moist Broadleaf Forests and Temperate Broadleaf and Mixed Forests biomes.
**2–Nemoral:** either restricted to Temperate Broadleaf and Mixed Forests or extending into both Tropical and Subtropical Moist Broadleaf Forests and Temperate Coniferous Forests biomes.
**3–Meridional:** generalists tolerating summer drought; otherwise, similar to the Nemoral category.
**4–Full-Mediterranean:** summer drought-tolerant specialists restricted to summer-hot and winter-mild biomes and climates.

Ancestral states of the unordered five-state categorical climate character were reconstructed under the *Mk1* model in Mesquite v.2.75 (Maddison and Maddison, 2011). We used two different input trees: (1) the original dated tree for standard top-down reconstruction of ancestral states (i.e. using only the information scored for the modern-day species); and (2) the dated tree with nodes and tips added to account for states of fossil taxa. Fossil taxa that could be associated with a distinct branch (lineage) were treated as sister lineages and used to break down the according branch. We used the oldest possible age of the fossil taxon as the age of the putative most recent common ancestor (MRCA) and the youngest possible age to define the MRCA-added tip distance. The Mesquite-NEXUS file is included in the Github repository/Zenodo submission (Supplementary Data S1: Data File S1-1).

## RESULTS

### Phylogenetic inference

**Fig. 1.**
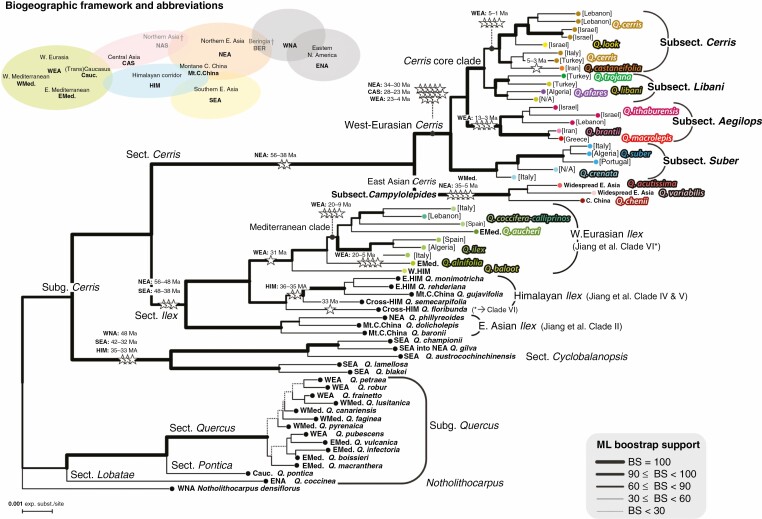
Maximum likelihood tree inferred from RAD-seq data of *Quercus* section *Cerris*, including members of *Quercus* sections *Ilex* and *Cylclobalanopsis* (subgenus *Cerris*), *Quercus* subgenus *Quercus*, and *Notholithocarpus* as an extended outgroup. Annotated are the number of fossil priors (stars) that can be linked to each clade (cf. Supplementary data Table S3) and their maximum possible age range, separately for main geographical regions where applicable. Tip labels give the geographical distribution of species (shown in bold; for abbreviations, see top left) or provenance of the individual sample (shown in normal text, in square brackets).

The RAD-seq locus dataset maintaining loci with a minimum of 15 individuals (m15 dataset; Fig. 1) yielded 5300 loci, totalling 464 762 aligned nucleotide positions and 64.1 % missing data. The m20 dataset yielded 3145 loci, 277 006 aligned nucleotide positions and 58.2 % missing data. The m25 dataset yielded 1132 loci, 100 841 aligned nucleotide positions and 46.4 % missing data. All datasets yielded the same ML topology for the section *Cerris* subtree (Fig. 1; Supplementary data Figs S1 and S2), with two exceptions. The m25 dataset placed one of the *Quercus libani* samples sister to *Quercus castaneifolia* + *Q. cerris* with weak support (Bootstrap support Percentage [BP] = 30 %; the second-best supported alternative, BP = 17 %, grouped it with the other *Q. libani* and *Quercus trojana*, in agreement with the m20 and m15 ML trees), and placed *Quercus variabilis* and *Q. chenii* sister to each other (BP = 78 %; no alternative with BP ≥ 15 %). The m15 and m20 datasets recovered the same topologies, and the m15 provided the strongest mean bootstrap support. Consequently, we report on the m15 dataset topology here (Fig. 1) and used that topology for our FBD constraints. The FBD dataset included 5075 loci and 444 591 aligned nucleotide positions, with 52.2 % missing data for the extant species (those with RAD-seq data).

**Fig. 2.**
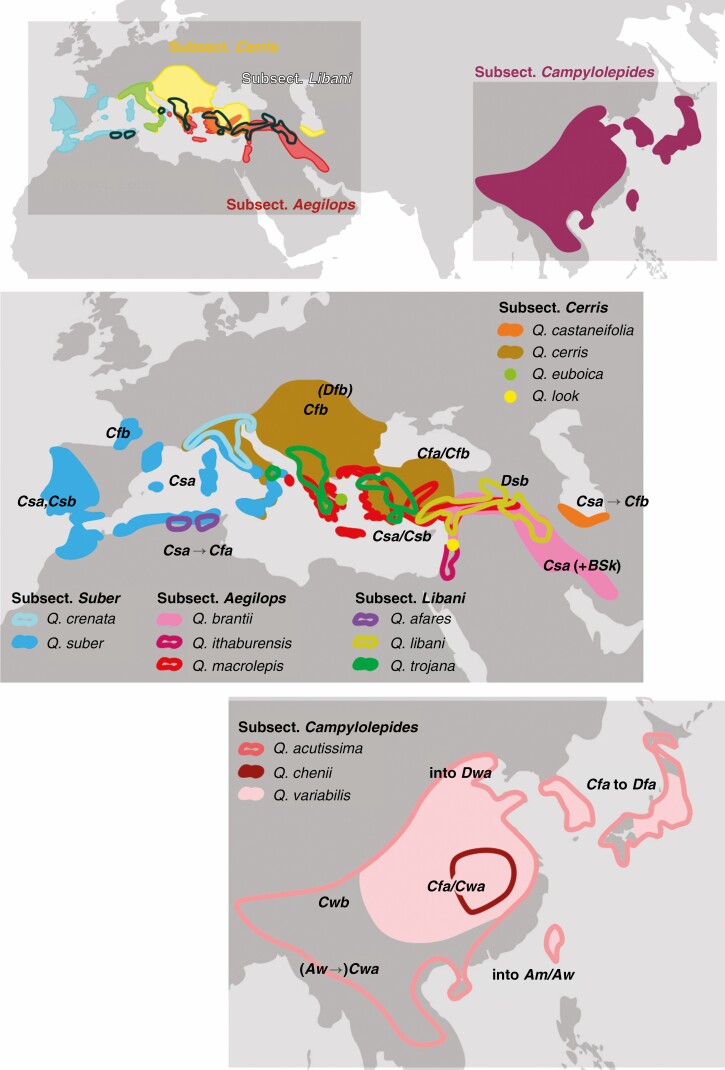
Distribution of *Quercus* section *Cerris* (main intrasectional clades: subsections and their constituent species) across Eurasia and main Köppen–Geiger climate types (cf. Table 1; Supplementary Data S3).

The RAD-seq ML phylogeny using the complete tip set (Fig. 1) indicates an initial divergence between the East Asian (subsection *Campylolepides* A.Camus) and the Western Eurasian species of section *Cerris* (Fig. 2). Within the Western Eurasian clade, the earlier nuclear ribosomal 5S Intergenic Spacer (5S-IGS)-identified species groups (Cluster 1–4 in the study by Simeone *et al.*, 2018) comprise four unambiguously supported clades, with two corresponding to subsections (*Suber* and *Aegilops*) and two unnamed (within what we are calling the *Cerris* core clade) (Table 2). Subsection *Suber* (Spach) Maleev comprises the Western Mediterranean cork oak, *Q. suber*, and the southern French–Italian–Croatian *Q. crenata*, commonly considered a hybrid between *Q*. *cerris* × *Q. suber*. Diverging next is subsection *Aegilops* (Reichenb.) Menitsky, including *Quercus macrolepis*, which ranges from south-eastern Italy to southern Turkey, and *Quercus ithaburensis* occurring further east and south to Israel (Browicz and Zieliński, 1982; Jalas and Suominen, 1988). The third species, *Quercus brantii*, ranges from south-eastern Turkey and north-western Syria to the Persian Gulf. The most recently diverged section *Cerris* core clade collects members of subsections *Libani* (new) and *Cerris* (Dumort.) Guerke. The former includes the North African and East Mediterranean–Near East species *Q. afares*, *Q. trojana* and *Q. libani*; subsection *Cerris* includes the narrow endemics *Q. castaneifolia* (Hyrcanian forest region of Azerbaijan and northern Iran) and *Quercus look* (northern Israel to Syria), and the widespread, genetically and morphologically heterogeneous Western Eurasian *Q. cerris*. The *D*-statistic tests demonstrate inter-locus phylogenetic discordance in the relative placement of subsections *Aegilops*, *Suber* and *Libani*, but none affects the conclusions presented here. There is no evidence of species-level introgression involving *Q. afares*, *Q. canariensis*, *Q. suber* or *Q. ilex* (Supplemental Data S1). According to Simeone *et al.* (2018), *Q. euboica*, not included in the present study, is a distinct species with subsection *Libani* morphology but genetically closer to subsection *Cerris* as defined here. It would thus be part of the section *Cerris* core clade.

**Table 2.** Subsectional classification of *Quercus* section *Cerris* in the present study and comparisons with previous classification schemes

Species	This study	Ørsted (1871)	Schwarz (1936)	Camus (1936–1954)	Menitsky (1984)
	Subsection	Section	Section	Subsection	Subsection
*Q. acutissima* Carruth.	*Campylolepides* A.Camus	[Not considered]	*Erythrobalanopsis*	*Campylolepides*	*Aegilops*
*Q. chenii* Nakai	*Campylolepides* A.Camus	[Described later]	[Not covered]	*Campylolepides*	*Aegilops*
*Q. variabilis* Blume	*Campylolepides* A.Camus	*Mucronatæ*	[Not covered]	*Campylolepides*	*Aegilops*
*Q. crenata* Lam.	*Suber* (Spach) Maleev	[Described later]	*Aegilops*	[Not recognized]	[Not covered]
*Q. suber* L.	*Suber* (Spach) Maleev	*Suber*	*Suber*	*Suber*	*Suber*
*Q. brantii* Lindl.	*Aegilops* (Reichenb.) Menitsky	*Mucronatæ*	*Aegilops*	*Macrolepides*	*Aegilops*
*Q. ithaburensis* Decne.	*Aegilops* (Reichenb.) Menitsky	*Dentatæ*	*Aegilops*	*Macrolepides*	*Aegilops*
*Q. macrolepis* Kotschy [incl.* Q. vallonea* Kotschy]	*Aegilops* (Reichenb.) Menitsky	*Eucerris*	*Aegilops*	*Macrolepides*	*Aegilops*
*Q. afares* Pomel	*Libani* subsect. nov.	[Described later]	*Eucerris*	*Macrolepides*	*Cerris*
*Q. libani* Olivier	*Libani* subsect. nov.	*Mucronatæ*	*Erythrobalanopsis*	*Macrolepides*	*Aegilops*
*Q. trojana* Webb	*Libani* subsect. nov.	*Mucronatæ*	*Erythrobalanopsis*	*Macrolepides*	*Aegilops*
*Q. euboica* (Papioann.) K.I.Chr.^a^	*Cerris* (Dumort.) Guerke vel *Libani*	[Described later]	[Described later]	[Not recognized]	[Not covered]
*Q. castaneifolia* C.A.Mey.	*Cerris* (Dumort.) Guerke	*Serratæ*	*Eucerris*	*Macrolepides*	*Cerris*
*Q. look* Kotschy	*Cerris* (Dumort.) Guerke	*Mucronatæ*	[Not covered]	*Macrolepides*	*Aegilops*
*Q. cerris* L.	*Cerris* (Dumort.) Guerke	*Eucerris*	*Eucerris*	*Eucerris*	*Cerris*

^a^ *Quercus euboica* was recently recognized as a distinct species (Simeone *et al.*, 2018).

Remarks on newly recognized (amended) subsectional classification:

**Subsection *Campylolepides*** Camus, 1934, included the East Asian members of section *Cerris* but also *Quercus acutissima* subsp. *roxburghii* (Endl.) A.Camus.

**Subsection *Suber*** (Spach) Maleev, 1935. Published in *Bot. Zhur*. 20, 2: 162.

**Subsection *Aegilops*** (Reichenb.) Menitsky, 1971, is the valid name. Subsection *Macrolepides* in Camus, 1936–38 is an invalid name because the same name was used by Camus in 1934 for a group of white oaks. Published in *Fl. Iranica* [Browicz and Menitsky, 1971 in Rechinger] 77: 12.

**Subsection *Libani.*** Typus: *Quercus libani* Oliv., 1801. Leaves thin or thick, glabrous or tomentose, lamina narrow elliptic, narrow ovate or oblong; tips of teeth pointed, short to long bristles; cupule hemispherical and barrel-shaped, cupule scales broad, rhombic or broadly triangular, adpressed.

**Subsection *Cerris*** (Dumort.) Guerke, 1897. Published in Richter, Pl. Eur. 2: 69.

### Dating and historical biogeography

**Fig. 3.**
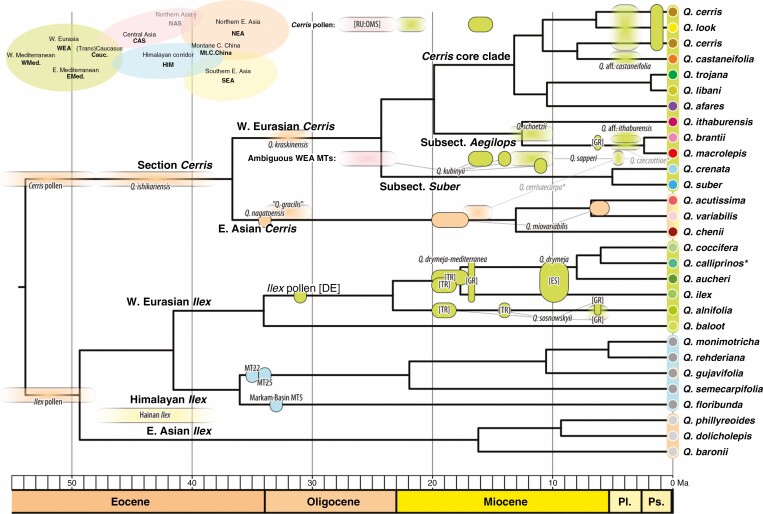
First of three chronograms for *Quercus* section *Cerris* and its sister clade section *Ilex* inferred with the FBD approach (pruned to modern-day tip set). The phylogenetic, stratigraphic (time/time slice) and geographical position of the used fossil dataset is indicated (23 and 14 fossil species for section *Cerris* and section *Ilex*, respectively; see Supplementary data Table S2). See Supplementary data (Table S5) for details across all three runs. Median rates and 95 % highest posterior density intervals are depicted in the Supplementary data (Fig. S4).

For each of three random draws from the uniform age distribution for all fossils, six to nine of the ten independent MCMC runs converged (Supplementary data Table S4). Burn-in was assessed by visual inspection of model likelihoods and estimated age distributions of the constrained nodes, and independent post-burn-in MCMC runs were pooled for each random draw of ages. Given that confidence intervals overlap strongly for all node ages across all three random draws from the fossil age distributions (Supplementary data Table S5), we report only the first of the three age draws in the main text of this paper, but provide results of all three in the Supplementary data (Tables S4, S5). The FBD dating with 47 fossils (Fig. 3; Supplementary data Fig. S3) indicates a pre-Oligocene (late Eocene) divergence between the Western Eurasian and East Asian lineages of *Cerris* (36.7 [40.6–35.0] Ma). Crown-group radiation (i.e. divergences leading to the modern-day species) started ~11 million years earlier in the Western Eurasian clade (latest Oligocene, 24.3 [28.6–20.8] Ma) than in their East Asian sister lineage, subsection *Campylolepides* (mid-Miocene, 13.1 [17.7–6.2] Ma). The Oligocene and Eocene north-eastern Asian *Cerris* fossils thus form the stem group of the section. The lineages leading to the modern widespread *Quercus acutissima* and *Q. variabilis* diverged in the Middle Miocene to Pleistocene, with high uncertainty (6.8 [13.3–0.1] Ma).

Among Western Eurasian *Cerris*, the western Mediterranean subsection *Suber* diverged from the remaining Western Eurasian *Cerris* in the late Oligocene to Early Miocene (24.3 [28.6–20.8] Ma). The split follows the expansion of section *Cerris* from north-eastern Asia into Central Asia as seen in the fossil record (Fig. 3). The second modern lineage, subsection *Aegilops*, diverged from the *Cerris* core clade (subsections *Libani* and *Cerris*) ~4 million years later (19.9 [22.7–16.9] Ma), before the Mid-Miocene Climatic Optimum. Crown-group radiation in the *Cerris* core clade, the split into subsections *Libani* and *Cerris*, coincided with crown-group radiation in the East Asian subsection *Campylolepides* and the start of speciation in subsection *Aegilops* (all mid-Miocene, within ±1 million years). Most of the modern species lineages diverged in the Late Miocene (Tortonian, 10 Ma) until earliest Pliocene (Zanclean, 5 Ma). The most recent speciation events were the Pleistocene split between the disjunct eastern Mediterranean *Q. macrolepis* and its eastern sister species, *Q. brantii* of Southeast Anatolia to Zagros Mountains (2.4 [0.1, 4.7] Ma) and the Italian to Turkish *Q. trojana* and the Near East *Q. libani* (1.8 [0.1, 10.8] Ma).

### *Modern distributions, climatic niches and major biome types of* Cerris *oaks*

*Quercus* section *Cerris* is the only oak section to reach its highest species richness and absolute phylogenetic diversity in Western Eurasia (12 spp., vs. 3 spp. in East Asia; Table 1). Each clade shows a broadly cohesive geographical distribution of parapatric to allopatric species (Fig. 2) that replace each other along a climatic cline (details in Supplementary Data S3).

The East Asian subsection *Campylolepides* covers a region from eastern Nepal to Japan and Laos. Its species can be categorized as Moist-Subtropical, Meridio-Nemoral or Nemoral. They thrive predominantly in warm, fully humid or winter-dry climates, occasionally extending into arid and cool climates. Their habitats are characterized by synchronous temperature and precipitation yearly minima (December–January). Only one species, the Nemoral *Q. variabilis* tolerates substantial frost during winter.

The Meridional to Full-Mediterranean subsection *Suber* of the Western Eurasian clade covers a region from Croatia and northern Italy south- and westwards to Tunisia, Algeria and Morocco, and to France, Spain and Portugal, specializing in Mediterranean climates but ranging into temperate climates. Here, *Q. suber*, the most west-extending species of the section, occurs predominantly in distinctly Mediterranean climates (hot summers with pronounced drought), whereas its eastern sister species, *Q. crenata*, thrives in sub-Mediterranean to fully humid climates (rare droughts). Both species form part of the Mediterranean Forests, Woodlands and Scrub and Temperate Broadleaf and Mixed Forests biomes.

Within subsection *Aegilops*, *Q. macrolepis* has a disjunct distribution from south-eastern Italy to eastern Turkey and occurs in Full-Mediterranean climates (Mediterranean Forests, Woodlands and Scrub biome) with extensive summer drought and mild winters. In more continental parts of the Balkan Peninsula and central Turkey, it grows within the Temperate Broadleaf and Mixed Forests biome and the Temperate Grasslands, Savannas and Shrublands biome as part of steppe forests (Supplementary Data S3). *Quercus macrolepis* is replaced eastwards by the more continental (cooler winters) *Q. ithaburensis* (Full-Mediterranean) and *Q. brantii* (Mediterranean into cool climates with summer drought, and into arid climates, with pronounced winter-cold). Both species occur in the Mediterranean Forests, Woodlands and Scrub biome; but *Q. brantii* has its main distribution in steppe forests and steppes (Temperate Broadleaf and Mixed Forests, Temperate Grasslands, Savannas and Shrublands; categorized as Meridional) of south-eastern Turkey, Iraq and Iran.

In the Meridional to Full-Mediterranean subsection *Libani*, *Q. afares* (Mediterranean climate) occurs in the northern parts of Algeria and Tunisia and is replaced to the north-east by *Q. trojana*, which has a disjunct distribution between Italy and Greece–Turkey (mostly Mediterranean climates), and is replaced in eastern Turkey by *Q. libani*, which thrives in Mediterranean but also in cold summer-dry and cold arid climates. *Quercus libani* represents the most frost- and snow-tolerant species in Western Eurasia, matching the most extreme habitats of *Q. variabilis* in north-eastern Asia (equally cold but drier winters). *Quercus afares* thrives in Mediterranean Forests, Woodlands and Scrub and, at higher elevations, in Temperate Conifer Forests with mild winters and summers without an extensive drought period. Regarding their climatic niches, *Q. afares* and *Q. libani* are almost mutually exclusive, with *Q. trojana* being intermediate. Both *Q. libani* and *Q. trojana* form part of the Mediterranean Forests, Woodlands and Scrub biome, and further east, *Q. libani* occurs in sympatry with *Q. brantii* (Temperate Broadleaf and Mixed Forests and Temperate Grasslands, Savannas and Shrublands biomes).

The widespread *Q. cerris* is ecologically variable (summer-dry Mediterranean into cool, frost- and snow-prone climates). Its climatic niche covers most of the total niche of section *Cerris*. It is the only species in its section with a main distribution in areas with a fully humid temperate climate with warm summers, and the only (extant) species in the section producing lobed leaves. The Full-Mediterranean *Q. look* is a narrow endemic in the Levant (Middle East: Mount Hermon, Anti-Lebanon and Lebanon Mountains), in a distinct Mediterranean climate setting, most similar to that of *Q. ithaburensis* in subsection *Aegilops*. *Quercus castaneifolia* is another endemic species south of the Caspian Sea separated from the nearest *Q. cerris* population by >600 km. Like other species categorized as Full-Mediterranean, it prefers (sub-)Mediterranean climates, which commonly are transitional to fully humid or summer-dry warm and cool climates. Its general niche resembles that of *Q. afares*, with mild but drier winters and no extensive summer drought; the precipitation maxima are concentrated in autumn. In contrast to Full-Mediterranean species, *Q. castaneifolia* typically occurs in mesic Temperate Broadleaf and Mixed Forests (i.e. it can be categorized as Meridional). *Quercus cerris* (Meridional or Full-Mediterranean) forms part of Mediterranean Forests, Woodlands and Scrub and of deciduous Temperate Broadleaf and Mixed Forests and, at higher elevations, Temperate Conifer Forests.

### Maximum likelihood reconstructions of ancestral climate zones and biomes

**Fig. 4.**
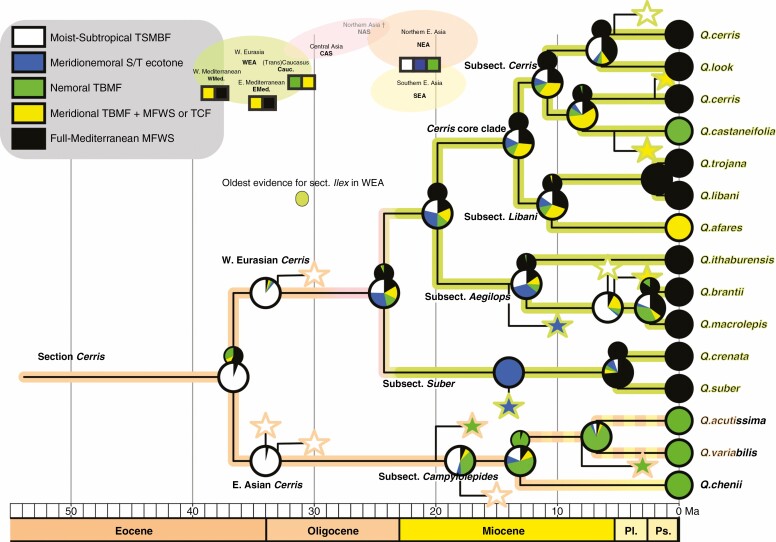
Maximum likelihood mapping of preferred biomes on the chronogram of the first run (Fig. 3), scored as five categories: Moist-Subtropical, Meridio-Nemoral, Nemoral, Meridional and Full-Mediterranean. Large pie charts give proportional likelihoods for most recent common ancestors (MRCAs) of modern-day species (at nodes) and (along branches) additional (shadow) MRCAs inferred by using fossil taxa (stars connected to tree) to break down subsequent branches. Coloured outlines of stars indicate the provenance of included fossil taxa and fossil taxa that could not be assigned clearly to a branch in the dated tree (unconnected stars). Small pie charts above big charts give the results when only modern-day states are considered.

Using only extant species, the MRCA of Western Eurasian *Cerris* oaks is reconstructed as Full-Mediterranean/Meridional, given that most modern species thrive in Mediterranean Forests, Woodlands and Scrub biomes under a Mediterranean climate with hot and dry summers, including the oro-Mediterranean belt (perhumid). For the East Asian subsection *Campylolepides* (biome-wise nemoral, climate-wise variable), the result is similarly biased (MRCA biome: nemoral; climate: ambiguous; small signatures in Fig. 4; Supplementary data Fig. S4).

In contrast, ML reconstructions incorporating fossil taxa suggest late (Early to Middle Miocene) biome and climatic niche shifts in East Asian members of section *Cerris* from Moist-Subtropical to (Meridio-)Nemoral. A shift from warm fully humid (Subtropical) to winter-dry monsoon climates (Meridio-Nemoral) is reconstructed for the Late Miocene (11.6–5.33 Ma; Supplementary data Fig. S4). In Western Eurasia, probable climate shifts from fully humid climates to climates with temperature and/or precipitation seasonality are inferred for the Middle Miocene (16.0–11.6 Ma) for the *Cerris* core group (sections *Cerris* and *Libani*). This is in accordance with high-resolution palynological data from East Mediterranean strata, which suggest a transition from equable warm-humid temperate climates to more seasonal (precipitation) and cooler climates (Bouchal *et al.*, 2020). Shifts to fully Mediterranean climates are reconstructed with high confidence for the Pleistocene (2.58–0.012 Ma). No shifts are reconstructed with high confidence for the Mediterranean Forests, Woodlands and Scrub biome before the Pliocene when fossil assemblages containing the fossil taxa are considered. The strong effect of including fossils in ancestral state reconstructions is illustrated in subsection *Suber*, where the fossil(s) cause a shift in inferred biome and climate from summer-wet to summer-dry conditions during the latest Miocene. Without information from the fossil record, no biome shift is reconstructed, while already by the Oligocene a preference for summer-dry climates (Meridional; Full-Mediterranean) is reconstructed within the Western Eurasian clade of section *Cerris*.

### *Leaf evolution and climatic niches in section* Cerris

**Fig. 5.**
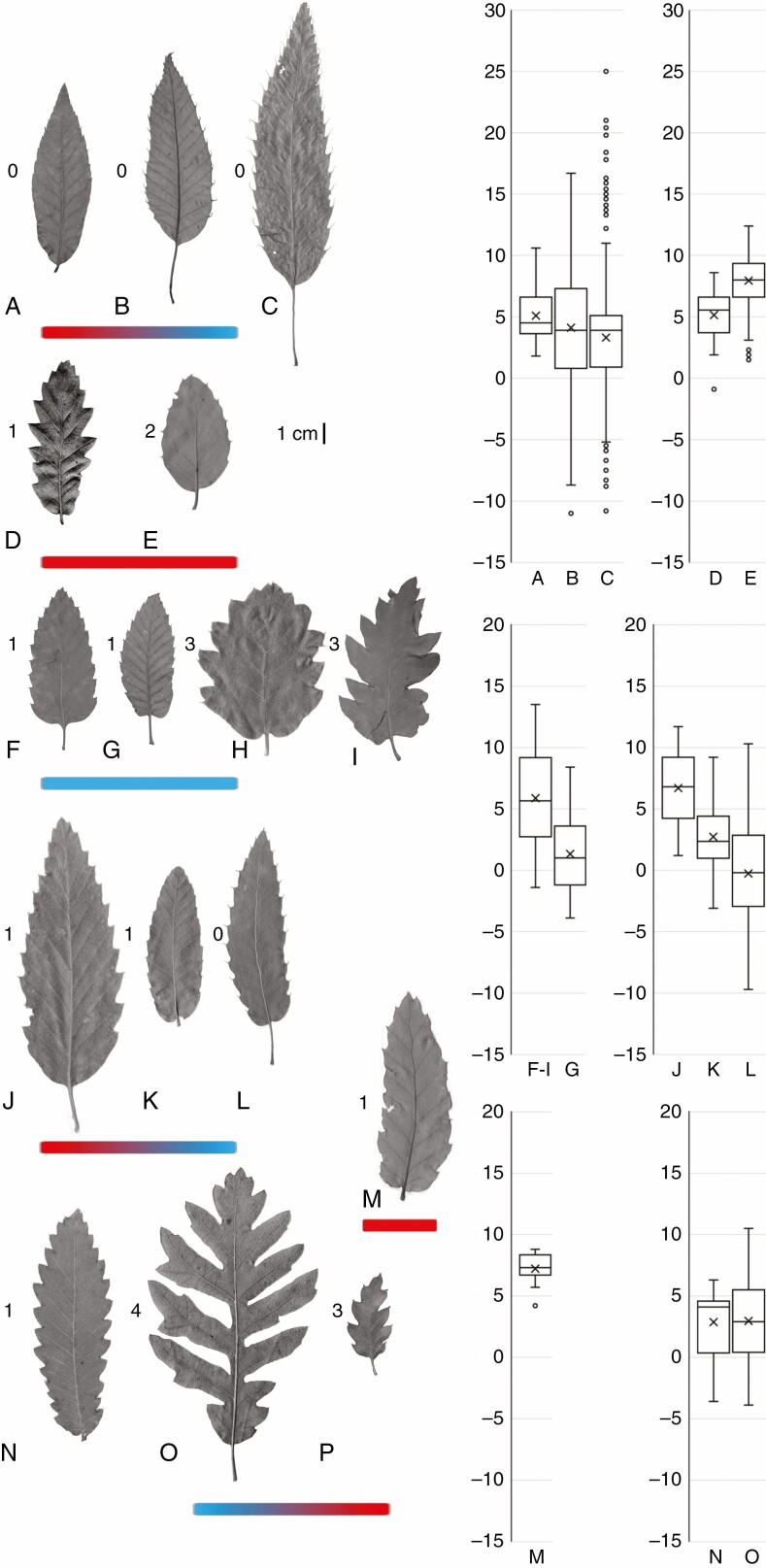
Leaf morphologies in extant subsections of *Quercus* sect. *Cerris*. (A–C) Subsection *Campylolepides*: (A) *Q. chenii* (herbarium P; P06859115); (B) *Q. variabilis* (herbarium E; E00294063_5); (C) *Q. acutissima* (E; E00671511_5). (D, E) Subsection *Suber*: (D) *Q. crenata* (P; P06856456); (E) *Q. suber* (herbarium S; Denk & Gruber 2005265_02). (F–I) Subsection *Aegilops*: (F) *Q. ithaburensis* (P; P06859855); (G) *Q. brantii* (E; E00404287_6); (H) *Q. macrolepis* (S; Denk & Grimm 2006051); (I) *Q. macrolepis* (P; P06859855). (J–L) Subsection *Libani*: (J) *Q. afares* (S; Denk & Gruber 2005256_02); (K) *Q. trojana* (S; Denk & Grimm 2006365); (L) *Q. libani* (Denk & Grimm 2006203). (M) *Cerris* core clade: *Q. euboica* (S; Denk, Ruhri, Ruhri 20081018_4). (N–P) Subsection *Cerris*: (N) *Q. castaneifolia* (E; E00404346_6); (O) *Q. cerris* (S; Denk & Grimm 2006110); (P) *Q. look* (Avishai 25_4). Thermometers indicate climate (temperature) niche evolution within subsections. For climate data, see Supplementary Data S3. Numbers to the left of representative leaves refer to leaf (tooth) types as defined in Supplementary Data S1 (Fig. S1-7).

Section *Cerris* exhibits high leaf variability in response to temperature (mean temperature of the coolest month; Fig. 5). A potentially ancestral leaf type, with narrow elliptic lamina, triangular teeth or reduced teeth with long bristle-like extensions, is present in all members of the East Asian subsection *Campylolepides* (Fig. 5A–C) thriving in summer-wet climates across a wide temperature range (Supplementary Data S3). This leaf type is also found amongst the earliest known leaf fossils of the section (*Quercus gracilis* [Pavlyutkin] Pavlyutkin; Supplementary data Table S2) and has been retained in the Western Eurasian subsection *Libani*, part of the *Cerris* core clade (Fig. 5L). Within *Campylolepides*, a correlation between leaf size and petiole length and cold tolerance is seen in the increase in both from *Q. chenii* to *Q. variabilis* and *Q. acutissima* (Fig. 5A–C; Supplementary Data S3).

A second leaf type is represented by the fossil species *Quercus kraskinensis* Pavlyutkin, which co-occurs with the fossil species *Q. gracilis* in the early Oligocene site of Kraskino (Supplementary data Table S2). This leaf morphotype is potentially symplesiomorphic in section *Cerris*, because it shares features with Western Eurasian species that are absent from the modern East Asian oaks (Supplementary Data S1: Fig. S1-6): teeth are usually strongly developed, with mucronate to cuspidate apexes and convex to sigmoid basal and apical sides. Early-diverging species in all Western Eurasian clades, irrespective of their diverse niche preferences, possess such leaves (subsection *Suber*: *Q. crenata*, Fig. 5D; subsection *Aegilops*: *Q. brantii*, Fig. 5G; subsection *Libani*: *Q. afares* and *Q. trojana*, Fig. 5J, K; subsection *Cerris*: *Q. castaneifolia*, Fig. 5N; and in *Q. euboica*, *Cerris* core clade, Fig. 5M).

Subsection *Suber* exhibits a leaf morphological gradient from a *crenata* type to a *suber* type, expressed by a reduction in leaf size and tooth area and a change from (semi)deciduous to (semi)evergreen, more leathery leaves. This gradient is associated with a climatic gradient from mesic (Nemoral; *Q. crenata*) to (Full-)Mediterranean conditions (*Q. suber*; Supplementary Data S1 and S3). Small ovate leaves with reduced cuspidate teeth lacking soft bristle-like extensions are found only in the semi-evergreen western Mediterranean *Q. suber* (Fig. 5E) and superficially resemble evergreen leaves of *Q. ilex* in section *Ilex*. Subsection *Libani* (Fig. 5J–L) exhibits a similar decreasing gradient in leaf size and tooth area associated with a decreasing gradient in cold tolerance and aridity from *Q. afares* to *Q. trojana* to *Q. libani*. In contrast to the diversity found in subsections *Suber* and *Libani*, all species in subsection *Aegilops* (the only subsection in which all members are adapted to pronounced summer-drought as well as winter-cold climates; Supplementary Data S3) have medium-sized leaves with teeth that range from weakly developed to complex, coarse teeth with subsidiary teeth (Fig. 5H, I).

Finally, in subsection *Cerris*, the greatest tooth area and largest leaf size are seen in *Q. cerris*, the most Nemoral species in Western Eurasia, and *Q. look*, a Full-Mediterranean species occupying a climatic niche that overlaps with both subsections *Aegilops* and *Libani*. Lobed leaves displaying an enormous variability are found exclusively in the widespread *Q. cerris* (Fig. 5O), which also exhibits a broad climatic niche, surpassed in the section only by the East Asian *Q. variabilis*. The remarkable leaf polymorphism of *Q. cerris* includes leaf types seen in *Q. castaneifolia* and *Q. look* (Fig. 5N, P; cf. fig. 7 in the study by Denk *et al.*, 2021*a*). The ecological–climatic and leaf-morphological variation of *Q. cerris* parallels the lack of genetic coherence in our RAD-seq dataset, with some *Q. cerris* sharing the genotype of *Q. castaneifolia*, whereas others exhibit a high degree of genetic similarity to *Q. look* (hence, represented by two tips in the subset used for dating). This might indicate ongoing speciation in subsection *Cerris*.

## DISCUSSION

### *Evolutionary and biogeographical history of section* Cerris

Section *Cerris* appears to have originated and diversified morphologically in northern East Asia by the early Oligocene. The oldest fossils of section *Cerris* are dispersed pollen grains from early Eocene (Ypresian, 56.0–47.8 Ma) strata of the Russian Far East (Shkotovskii Basin; Naryshkina and Evstigneeva, 2020; Pavlyutkin *et al.*, 2020). By the early Oligocene, section *Cerris* was present with at least two distinct fossil species based on leaves in the Russian Far East Kraskino Flora (34–30 Ma; Pavlyutkin *et al.*, 2014; Pavlyutkin, 2015). Importantly, these oldest East Asian fossil records pre-date the earliest known fossils of *Cerris* in Western Eurasia (dispersed pollen from Germany, Altmittweida; earliest Miocene, 23–20.5 Ma; Standke *et al.*, 2010; Kmenta, 2011) by >10 million years, and unambiguous leaf records of section *Cerris* are not known in Western Eurasia before the Miocene (e.g. Knobloch and Kvaček, 1976; Mai, 1995). Foliage described as ‘*Q. gracilis*’ (Pavlyutkin) Pavlyutkin (nom. illegit.) is very similar to modern leaves of East Asian members of section *Cerris* (Fig. 5). Another species described from the Kraskino Flora, *Q. kraskinensis* Pavlyutkin (Pavlyutkin, 2015), is strikingly similar to a number of modern Western Eurasian *Cerris* oaks, in particular to *Q. crenata*, the root-proximal species in the second-diverging subsection *Suber*, and to a lesser degree to *Q. trojana* and *Q. cerris*, members of *Cerris* core clade (present study; Supplementary Data S1: Plate S1-1). Thus, this leaf morphology is characterized by shared features, ancestral (possibly symplesiomorphic) within section *Cerris*. *Quercus kraskinensis* might represent a precursor or early member of what would become the Western Eurasian clade of *Cerris* (Fig. 3). The East Asian Palaeogene record thus demonstrates that most of the range of *Cerris* leaf morphological diversity evolved at the dawn of the section, with the ‘*kraskinensis-crenata*’ leaf type originating in East Asia and surviving in Western Eurasia (Supplementary Data S1).

**Fig. 6.**
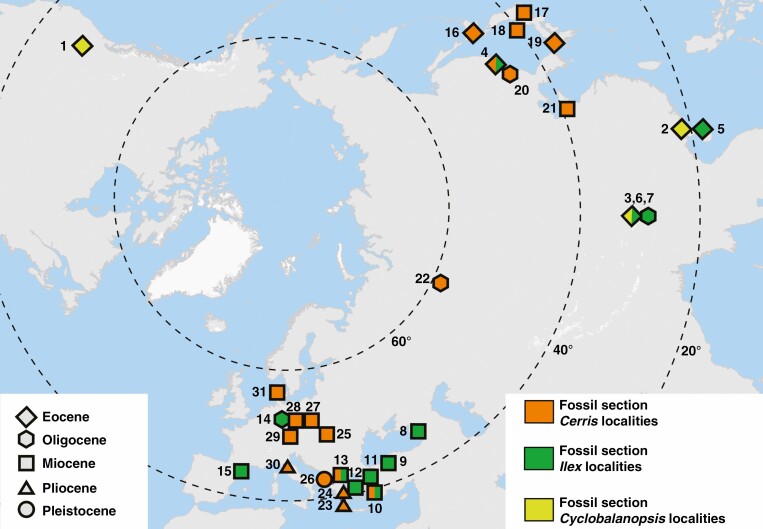
Distribution of fossil members of section *Cerris* used for the chronogram (Fig. 3). The map was produced with the software QGIS (QGIS, 2021).

The northern East Asian origin of section *Cerris* (cf. Fig. 3) is also supported by evidence from previous molecular studies. First, all modern *Cerris* share the same, section-unique plastid lineage, indicative of a single point of origin and quick dispersal, with the East Asian subsection *Campylolepides* showing the overall highest plastid divergence (Simeone *et al.*, 2018; Zhang *et al.*, 2020; Li *et al.*, 2022). Second, the *Cerris* plastomes are part of a haplotype lineage shared with a group of section *Ilex* species thriving in modern-day Japan and the mountains of northern and central China (East Asian clade in Figs 1 and 3; Simeone *et al.*, 2016: *Quercus engleriana* Seemen, *Q. phillyreoides* and *Q. spinosa* David; Zhou *et al.*, 2022: *Quercus dolicholepis*, *Q. engleriana*, *Q. spinosa* and *Q. pseudosetulosa* Q.S.Li & T.Y.Tu), i.e. geo-historically close to the oldest *Cerris* fossils of the Russian Far East (Fig. 6). Most of these species belong to Jiang *et al.*’s (2019) early-diverging ‘clade II’ (before the mid-Eocene, ≥ ~40 Ma, in Jiang *et al.*, 2019; median divergence stem age of 49 Ma, present study, Fig. 3). In combination, the fossil and molecular data work together to provide a robust picture of the East Asian origins for the iconic European section *Cerris*.

The fossil record of *Cerris* then leads westwards along a northern route, via northern and Central Asia, chiefly following the then-high-latitude (≥60°N; Scotese *et al.*, 2014) ‘warm’ climate zone and avoiding the shrinking boreotropics (*sensu*Wolfe, 1975; paratropical floras *sensu*Mai, 1995). Fossil taxa comparable to section *Cerris* have been reported from Oligocene and Early Miocene central and eastern Kazakhstan and Russia under various names (*Quercus pseudocastanea*, *Q. furuhjelmii*, *Q. pseudorobur*, *Q.* sp. etc.; e.g. Kryshtofovich *et al.*, 1956; Yakubovskaya, 1957; Kornilova, 1960; Rajushkina, 1979; Takhtajan, 1982). From late Oligocene strata of south-western Siberia, Denk *et al.* (2021*b*) reported dispersed pollen and leaves [*Quercus* cf. *kubinyii* (Kováts in Ettingsh.) W.Berger] unambiguously belonging to section *Cerris*. The large potential area and relatively smooth palaeotopography (Scotese, 2014) would fit with the overall low plastid differentiation observed in the section, because topographic variation is broadly associated with phylogenetic diversity and speciation in woody plants (Verboom *et al.*, 2015; Feng *et al.*, 2016; Jin *et al.*, 2021) and oaks in particular (Hipp *et al.*, 2018). Furthermore, our FBD dating places the initial radiation of *Cerris* ~10 million years after that of its sister lineage section *Ilex* (Fig. 3), which took a southern route via the ‘Himalayan corridor’ (Simeone *et al.*, 2016; Jiang *et al.*, 2019).

After the earliest appearance of *Cerris* in Western Eurasia in Early Miocene deposits of Germany, dispersed pollen grains of section *Cerris* are also known from slightly younger Burdigalian strata of Turkey and Greece (Denk *et al.*, 2017*b*). Given the complex tectonic situation and the availability of different more or less temperate niches during the Early and Middle Miocene, it is conceivable that *Cerris* evolved several lineages within its first 10 million years (subsects *Suber*, *Aegilops* and *Cerris* core clade; Fig. 3). One outcome of a rapid diversification might be partial reproductive compatibility between these lineages, as evidenced by admixture among subsections (Supplementary Data S1). A rapid origin and spread with gene flow between lineages is also supported by the low plastid differentiation, decoupled from main intrasectional lineages (subsections) and species. *Cerris* oaks then became relatively widespread (*Quercus kubinyii*) in Western Eurasia during the Middle Miocene, ranging from Denmark to Anatolia (Kováts, 1856; Christensen, 1976; Knobloch, 1986; Güner *et al.*, 2017; Denk and Bouchal, 2021). These leaf remains resemble modern species of section *Cerris* that possess ancestral leaf types (Fig. 5; leaf types ‘0’ and ‘1’). Section *Cerris* thus appears to have colonized and diversified in Europe and the Mediterranean from the Early to mid-Miocene, providing the ancestry for the modern-day species and subsections. Our combination of phylogenomic data with a rich set of fossils suggests that main lineages in Western Eurasia might have been established before their modern morphologies (Figs 3 and 5; Supplementary Data S1).

The *Cerris* history becomes all the more interesting in light of its parallels with the section *Ilex* history. Fossil pollen of both *Quercus* sections *Cerris* and *Ilex* in early Eocene strata of the Russian Far East (Naryshkina and Evstigneeva, 2020) indicate that both lineages might have originated in high-latitude warm temperate biomes (cf. Scotese *et al.,* 2014). But while section *Cerris* had a first radiation in Northeast Asia and subsequently migrated to Western Eurasia north of the progressively enlarging Qinghai–Tibet Plateau, its sister clade, the evergreen section *Ilex*, initially migrated southwards and south-westwards into tropical China and south-eastern Tibet (Linnemann *et al.*, 2017; Su *et al.*, 2019; Hofmann, 2010), thence westwards into Europe and the Mediterranean along the proto-Himalayas south of the Qinghai–Tibet Plateau (Jiang *et al.*, 2019). Section *Cerris* makes it into southern China only in Late Miocene strata of western Yunnan (Xia *et al.*, 2009; Xu *et al.*, 2012). The co-occurrence of the sections in East Asia is further supported by shared plastids of northern East Asian species of section *Ilex* (mostly Jiang *et al.,* 2019, clade II) with section *Cerris* (Simeone *et al.*, 2016; Yan *et al.*, 2019; Zhou *et al.*, 2022). Moreover, the *Cerris*–*Ilex* shared plastome lineages differ substantially from plastome lineages shared between section *Ilex* and the East Asian section *Cyclobalanopsis* (Yang *et al.*, 2018), further pointing to the divergent history of these sections in Southeast Asia. Thus, molecular data corroborate that Western Eurasian *Cerris* came into contact with *Ilex* only after the East Asian Eocene and Oligocene members of *Cerris* had begun to move westwards. Plastid phylogeography thus fits with a scenario of largely isolated early evolutionary histories of *Cerris* (northern Asia) and *Ilex* (Himalayan Corridor).

### Ecological and climatic niche evolution

Our reconstructions of biome and climatic niche suggest persistence of the Tropical and Subtropical Moist Broadleaf Forests biome during the Oligocene, consistent with fossil records (e.g. Kvaček, 2010; Pavlyutkin *et al.*, 2014). In contrast, reconstructions that ignore the fossils (Fig. 4) suggest shifts into the Mediterranean Forests, Woodlands and Scrub biome that are at odds with the Western Eurasian fossil record, where this biome is not recorded before the Plio-Pleistocene (Suc, 1984; Velitzelos *et al.*, 2014). The early Eocene (Ypresian, 56–47.87 Ma) split between sections *Cerris* and *Ilex* must have coincided with the origins of deciduousness in *Cerris*, whereas *Ilex* retained the original evergreen leaf habit of subgenus *Cerris* (which is also characteristic of section *Cyclobalanopsis*, sister to the *Cerris* + *Ilex* clade). The subsequent biogeographical histories of the two lineages reflect this change. Members of section *Cerris* moved westwards as a northern lineage, part of a more or less temperate forest biome dominated by deciduous tree species during the Oligocene (Kryshtofovich *et al.*, 1956; Popova *et al.*, 2013; Pavlyutkin *et al.*, 2014; Scotese *et al.*, 2014; Willis and McElwain, 2014; Averyanova *et al.*, 2021; Denk *et al.*, 2021*b*). Extensive lowlands in large parts of Siberia and northern Kazakhstan provided nutrient-rich substrates dominated by deciduous woody plants (‘warm temperate biome’ in the paper by Willis and McElwain, 2014; ‘warm [temperate]’ climate zone in the paper by Scotese *et al.*, 2014). To the south of this more or less temperate forest belt, a drier region was occupied by the ‘subtropical summerwet biome’ (coined wooded savannah to semi-desert; Willis and McElwain, 2014) corresponding to an arid climate zone with frosts according to Scotese *et al.* (2014). Here, section *Cerris* oaks would have had an advantage owing to the earlier origins of deciduous leaf phenology.

This trait diversity is likely also to have shaped the ecological diversification of section *Cerris* and the high community-level diversity of Mediterranean oaks. In Western Eurasia, *Cerris* oaks colonized a wide range of habitats, reflected in their Miocene distribution from southern Scandinavia to southern Turkey, a range of almost 20° latitude (Fig. 6). Three key morphological features of *Cerris* oaks might have provided advantages during subsequent shifts into their modern Mediterranean habitats: deciduous or semi-deciduous leaves, large acorns protected by sturdy cups, and corky stems. In contrast, the evergreen oaks of section *Ilex* that spread southwards and south-westwards during the Eocene from the temperate to the subtropical summer-wet biome differentiated geographically (Jiang *et al.*, 2019) but remained evergreen. The resulting climatic niche partitioning in the Western Eurasian subsections of *Cerris* oaks (Supplementary Data S3) and between sections *Ilex* and *Cerris* might have enabled the coexistence of several summer- and wintergreen oak lineages: ≤13 species of both deciduous and evergreen oaks from three sections (*Quercus*, *Cerris* and *Ilex*) presently co-occur in southern Turkey, for example (Hedge and Yaltirik, 1982; Blumler, 2015).

Ecological lability of section *Cerris* also manifests in both niche convergence and trait convergence. *Quercus suber*, for example, is the only European tree species that is able to resprout after fire damage (Pausas, 1997; Houston Durrant *et al.*, 2016). It is also highly flexible in the timing and duration of leaf abscission and can rapidly replace old leaves with new shoots (Escudero and del Arco, 1987), characteristics typical of all members of section *Cerris*. Yet a number of *Q. suber* adaptations are more reminiscent of evergreen species of section *Ilex*: its ability to switch between annual and biennial fruit maturation in response to climate, semi ring-porous wood anatomy that reduces the risk of embolism in droughty springs, and sandy lowland habitat (Elena-Rosello *et al.*, 1993; Sousa *et al.,* 2009). In contrast, *Q. crenata*, the sister species of *Q. suber*, retains many putatively ancestral traits of section *Cerris* (corky bark, leaf texture, leaf abscission, leaf shape, ring-porosity and partly humid temperate climatic niche) and is morphologically similar to some of the oldest known fossil species of *Cerris* (e.g. *Q. kraskinensis*), perhaps owing to niche conservatism and repeated phases of introgression (Table 3). *Quercus cerris*, the most temperate of the *Cerris* oaks, provides an even more pronounced example of convergence between sections. It is the only oak in all of subgenus *Cerris* with complex lobed leaves, reminiscent of the lobed white oaks of *Quercus* section *Quercus* (subgenus *Quercus*). Lower hydraulic resistance in deeply lobed leaves might provide a mechanism for improving water balance in dry atmospheric conditions (Siso *et al.*, 2001) and also enable leaves to pack more efficiently into buds (Edwards *et al.*, 2016; Givnish and Kriebel, 2017; Zohner *et al.*, 2019). Lobedness is thus a convergent trait that might contribute to the ability of *Q. cerris* to grow sympatrically with (co-)dominant lobed white oaks, such as *Quercus robur* L., *Q. petraea*, *Q. frainetto* Ten. and *Q. vulcanica* Boiss. ex Kotschy, and account for its long history of cultivation in the British Isles (Loudon, 1838). Section *Cerris* thus contributes to our growing understanding of the importance of both divergence within sections and convergence between them in shaping oak diversity and patterns of coexistence (cf. Cavender-Bares *et al*., 2004, 2015, 2018).

### Conclusions

Two different migration routes resulted in distinct diversity patterns in the sister sections *Cerris* and *Ilex*. The deciduous section *Cerris* is most ecologically and taxonomically diverse in Western Eurasia (12 of 15 species), whereas the evergreen section *Ilex* is most diverse in East and Southeast Asia into the Himalayas (21 of ~25 species). Globally, evergreen broadleaf species occur in relatively humid (~1500–3000 mm mean annual precipitation) and warm (mean temperature of the coldest month > 0 °C) climates, whereas winter deciduous broadleaf species typically occur in relatively less humid (mean annual precipitation ~700–1500 mm) and cooler climates (mean temperature of the coldest month < 0 °C; Woodward *et al.*, 2004). Nevertheless, both evergreen sclerophyllous and deciduous oak species are, at present, highly diverse in summer-dry Mediterranean areas. Extensive research in modern Mediterranean ecosystems suggests that neither evergreen nor deciduous species grow optimally there (Escudero *et al.*, 2017).

Our time-calibrated phylogenetic reconstruction using 47 fossils suggests that suboptimal adaptation to current Mediterranean climate might be a deep-time evolutionary legacy in both the evergreen section *Ilex* and the deciduous section *Cerris*, resulting from their differential early range expansions from Northeast Asia. Section *Cerris* shifted to deciduous leaves in frost-free environments, which would have preadapted the lineage to the dry and cold climates it encountered in its westward expansion (trait first pattern). Section *Ilex* retained its evergreen leaf phenology and did not shift to deciduous leaves when colonizing winter-cold and -dry habitats in the Himalayas and warm, summer-dry environments in the Mediterranean region. Western Eurasia thus became a meeting ground for old relatives of divergent sources: the Northeast Asian *Cerris*, the Southeast Asian *Ilex* and, ultimately, the eastern North American white oaks of section *Quercus*, which joined them between 10 and 20 Ma (Denk and Grimm, 2010; Denk *et al.*, 2010; Hipp *et al*., 2018, 2020). These legacies explain why species of all three sections co-occur in contemporary Mediterranean climates of Western Eurasia and how their distributions follow environmental and climatic gradients within the wider Mediterranean region.

## Supplementary Material

mcad032_suppl_Supplementary_Data

## Data Availability

All data, scripts and supplemental information (Supplementary Data S1–S3; Tables S1–S5; Figs S1–S4) is included in the associated Github repository https://github.com/andrew-hipp/cerris-fbd; release v.1.0-1 has been archived in Zenodo (https://doi.org/10.5281/zenodo.7547523).

## SUPPLEMENTARY DATA

Supplementary Figure S1. Raw maximum Likelihood tree inferred from the m20 RAD-Seq data (3145 loci, 277,006 aligned nucleotide positions, and 58.2% missing data), rooted with Notholithocarpus. Numbers at branches indicate non-parametric bootstrap support.

Supplementary Figure S2. Raw maximum Likelihood tree inferred from the m25 RAD-Seq data (1132 loci, 100,841 aligned nucleotide positions, and 46.4% missing data), rooted with Notholithocarpus. Numbers at branches indicate non-parametric bootstrap support.

Supplementary Figure S3. Same dated phylogenetic tree as shown in main-text Figure 3 (first of three runs). Top: chronogram with fossil-species included (unlabelled tips) and branches coloured according to the estimated median substitution rates. Bottom: chronogram showing the 95% HPD intervals for inferred species divergences of extant species.

Supplementary Figure S4. Maximum likelihood mapping of preferred Köppen climates on the chronogram of the first run (main-text Fig. 3); scored as five categories: Moist-Subtropical, Meridio-Nemoral, Nemoral, Meridional, Full-Mediterranean. Large pie charts give proportional likelihoods for most-recent common ancestors (MRCAs) of modern-day species (at nodes) and (along branches) additional (shadow) MRCAs inferred by using fossil-taxa (stars connected to tree) to break down subsequent branches. Coloured outlines of stars indicate the provenance of included fossil-taxa and fossil-taxa that could not be clearly assigned to a branch in the dated tree (unconnected stars). Small pie charts above big charts give the results when only modern-day states are considered.

Supplementary Data S1. Extended methods and results.

Supplementary Data S2. GoogleEarth locality (kmz) files indicating the probable position of all here-used fossils within their palaeogeographic context (palaeogeographic globes from Scotese, 2013*a*, 2013*b*, 2013*c*, 2013*d*, 2013*e*)

Supplementary Data S3. Köppen distribution and biome maps for all species of section *Cerris*, as well as x-y and box-plots for selected bioclimatic parametres.

Supplementary Table S1–S5. COPY FROM HEADERS.
