# Supplementary material for: Niche evolution in a northern temperate tree lineage: biogeographical legacies in cork oaks (Quercus section Cerris)
Source: Ann Bot. 2023 Feb 20;131(5):769–87. doi: 10.1093/aob/mcad032 (PMC10184457; doi:10.1093/aob/mcad032)

Supplementary Figure 1. Raw maximum Likelihood tree inferred from the m20 RAD-Seq data (3145 loci, 277,006 aligned nucleotide positions, and 58.2% missing data), rooted with *Notholithocarpus.* Numbers at branches indicate non-parametric bootstrap support.

Supplementary Figure 2. Raw maximum Likelihood tree inferred from the m25 RAD-Seq data (1132 loci, 100,841 aligned nucleotide positions, and 46.4% missing data), rooted with *Notholithocarpus.* Numbers at branches indicate non-parametric bootstrap support.

Supplementary Figure 3. Same dated phylogenetic tree as shown in main-text Figure 3 (first of three runs). Top: chronogram with fossil-species included (unlabelled tips) and branches coloured according to the estimated median substitution rates. Bottom: chronogram showing the 95% HPD intervals for inferred species divergences of extant species.

Supplementary Figure 4. Maximum likelihood mapping of preferred Köppen climates on the chronogram of the first run (main-text Fig. 3); scored as five categories: Moist-Subtropical, Meridio-Nemoral, Nemoral, Meridional, Full-Mediterranean. Large pie charts give proportional likelihoods for most-recent common ancestors (MRCAs) of modern-day species (at nodes) and (along branches) additional (shadow) MRCAs inferred by using fossil-taxa (stars connected to tree) to break down subsequent branches. Coloured outlines of stars indicate the provenance of included fossil-taxa and fossil-taxa that could not be clearly assigned to a branch in the dated tree (unconnected stars). Small pie charts above big charts give the results when only modern-day states are considered.

Supplementary Figure 1

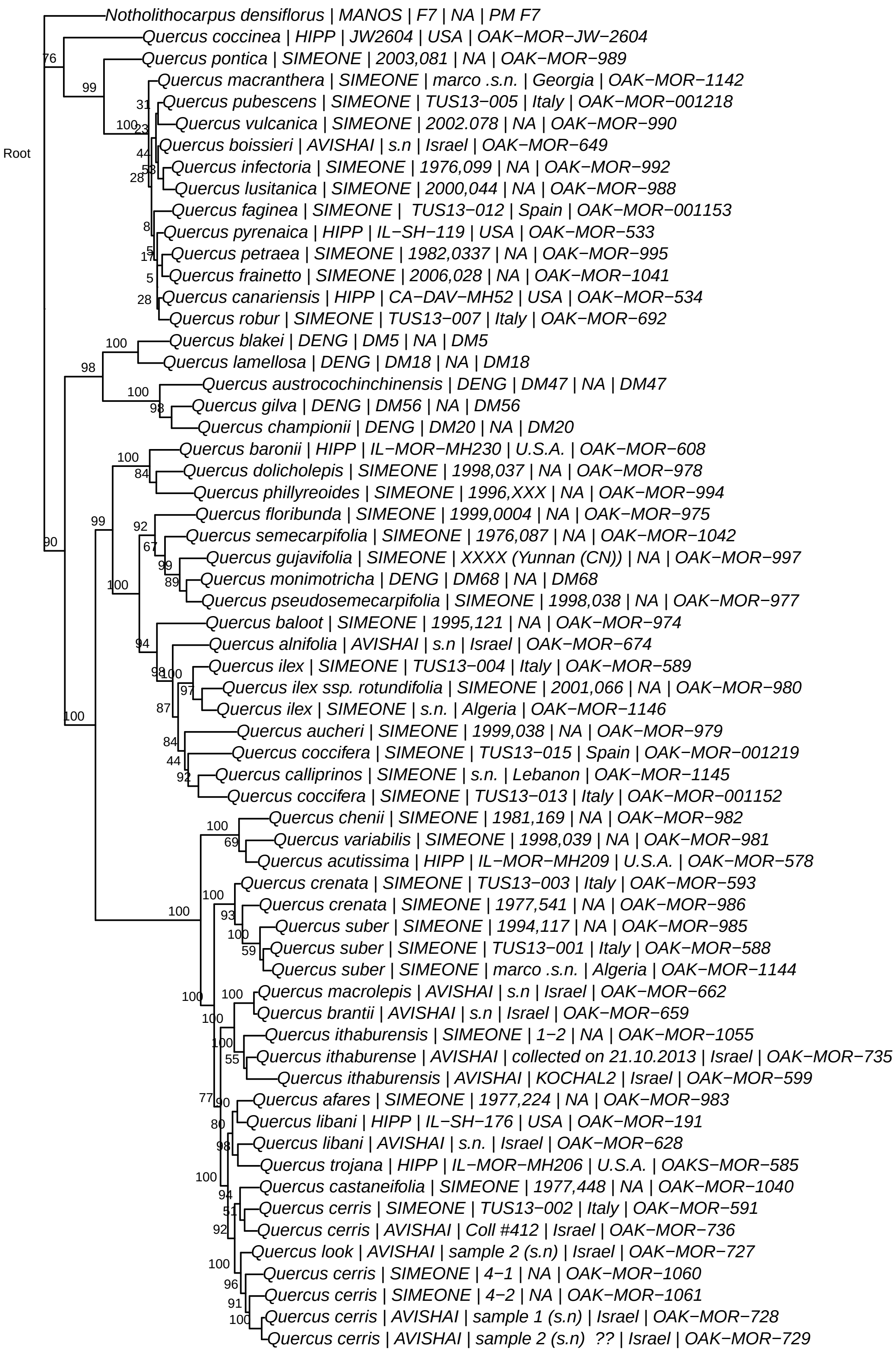

Supplementary Figure 2

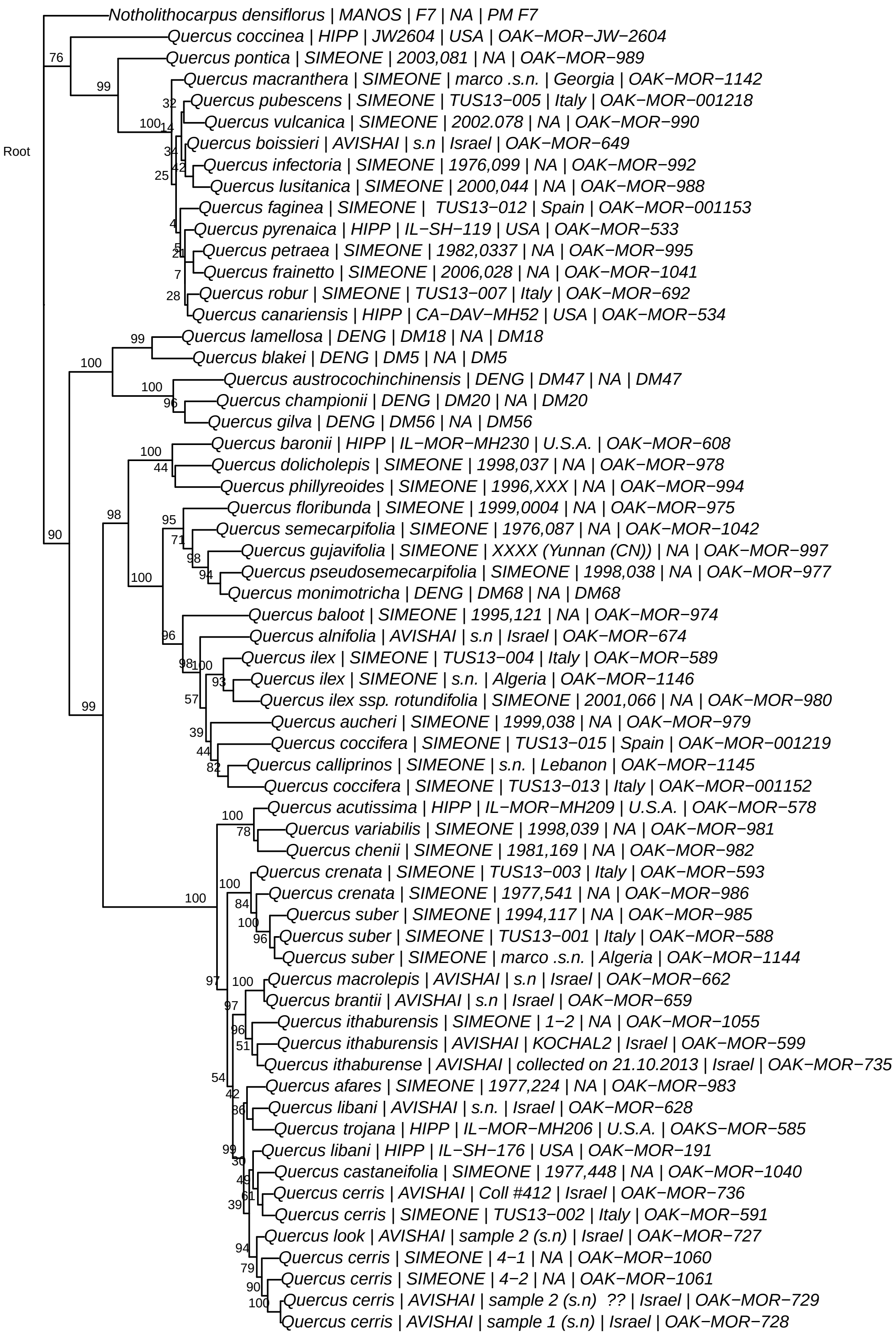

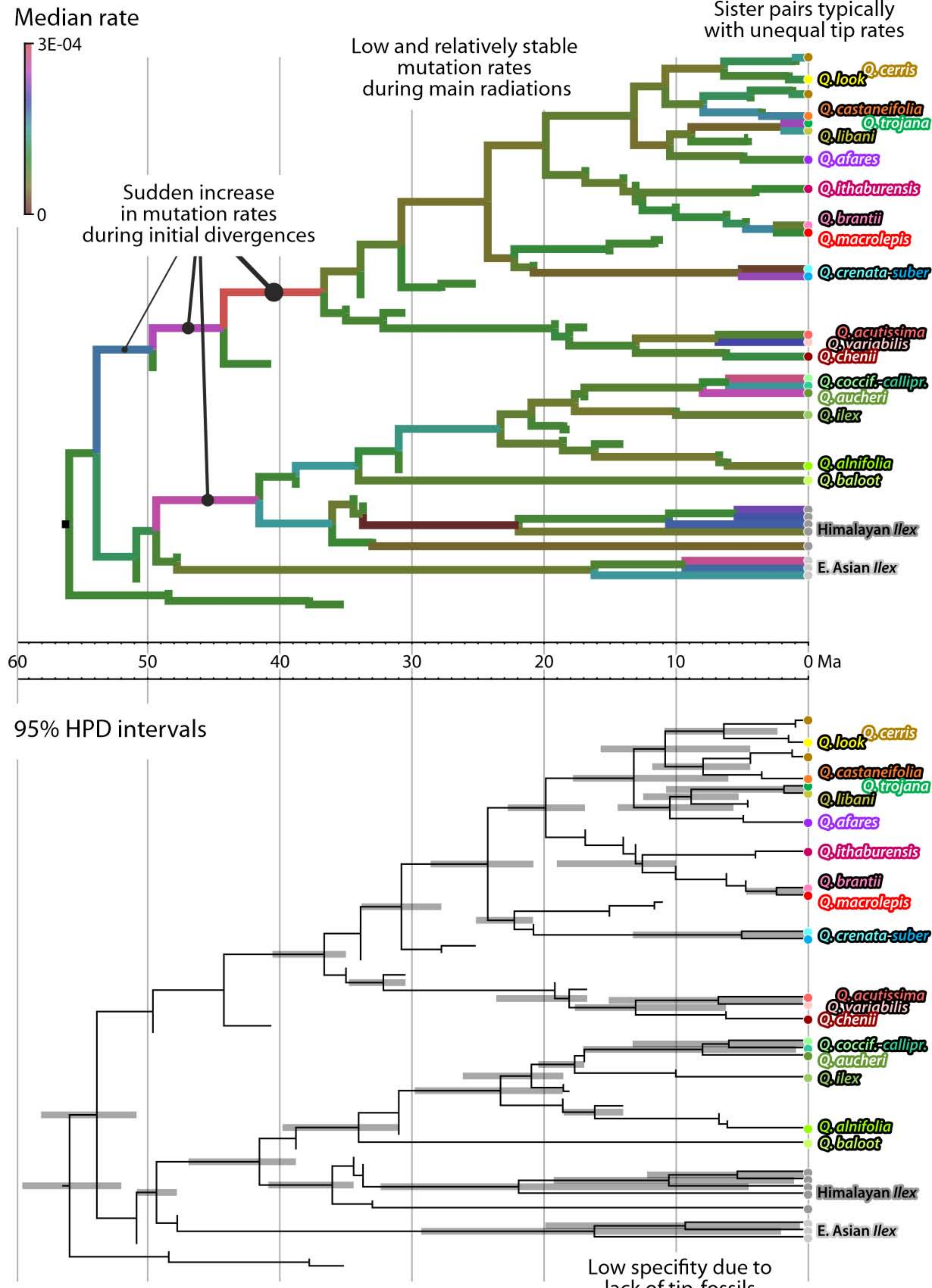

Supplementary Figure 3

Supplementary Figure 4

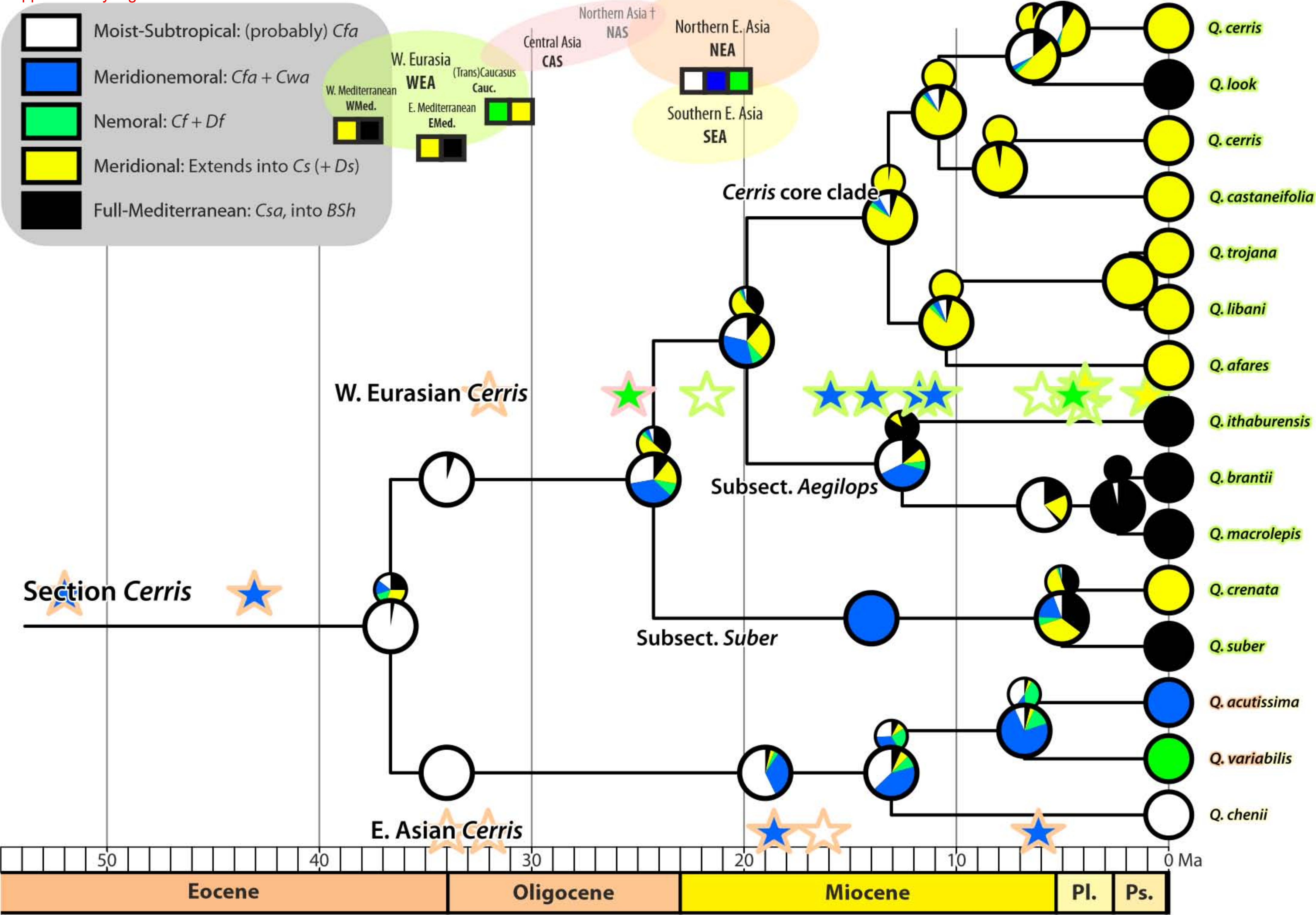

Supplement: mcad032_suppl_Supplementary_Data [file mcad032_suppl_supplementary_data.zip › Supplementary Figures 1 to 4.pdf]
