# Supplementary material for: Niche evolution in a northern temperate tree lineage: biogeographical legacies in cork oaks (Quercus section Cerris)
Source: Ann Bot. 2023 Feb 20;131(5):769–87. doi: 10.1093/aob/mcad032 (PMC10184457; doi:10.1093/aob/mcad032)
Supplement: mcad032_suppl_Supplementary_Data [file mcad032_suppl_supplementary_data.zip › Supplementary Data 1/Supplementary Data 1 - methods results discussion.pdf]

Supplementary Data 1: Extended Methods and Results

Introduction into *Quercus* Section *Cerris*

Quercus section *Cerris* Dumort. (Denk *et al*., 2017*a*) is a small group of oaks of the Eurasian subgenus *Cerris* Oerst., which includes the iconic cork oak, *Q. suber* L., the source of commercial cork. Well-developed cork layers are present not only in the western Mediterranean *Q. suber* but also in the East Asian *Q. variabilis* Blume ("Chinese cork oak"). Both are used industrially (Huang *et al.*, 1999). Clearly developed but much thinner cork layers occur in *Q. crenata* Lam., *Q. afares* Pomel, and some varieties and forms of *Q. cerris* L. (for example var. *gussonei* Borzì in Sicily; "subsp./var. *pseudocerris*" in Turkey and Lebanon; Maire, 1961; Schirone *et al.*, 2015; Pignatti, 2017–2019). Distinctly corky bark does not occur in any other oak section, but it is present in at least five out of the 15 species included in section *Cerris* and justifies the name "cork oaks" for the section.

The cork oak section is remarkable biogeographically and ecologically. First, it is the only group of oaks that is most diverse in western Eurasia, despite also being widespread in East Asia (*Supplementary Data 3*). Second, it is strictly deciduous but comprises several semi-evergreen species adapted to summer-dry and winter-cold, continental climates. Third, a single species in this group, *Q. cerris*, the most widespread western Eurasian cork oak, independently evolved lobed leaves reminiscent of many species in *Quercus* section *Quercus*, the white oaks. Finally, the cork oaks provide well-documented examples of intrasectional and intersectional introgressive hybridization that provide insight into the timing of diversification and gene flow in the genus. In a recent study, Simeone *et al.* (2018) investigated western Eurasian members of section *Cerris* using a broad-sampled chloroplast (*trnH-psbA*) and a multi-copy nuclear (5S-IGS) marker. Chloroplast haplotypes reflected shared biogeographic histories of all western Eurasian *Cerris* species and its sister section *Ilex*, indicating several phases of "chloroplast capture" and secondary, species-decoupled, geographic sorting. Simeone *et al.* (2018) suggested that the precursors of western Eurasian *Cerris* species came into contact with *Ilex* only after the initial range expansion of *Cerris* from north-eastern Asia during the early Oligocene. In addition, four main nuclear 5S-IGS lineages were identified within *Cerris*, three of which were private to potential sibling species: (*i*) root-proximal 'Oriental' lineage in *Q. afares*,

Q. libani Olivier, *Q. trojana* Webb; (*ii*) 'Occidental' lineage found in the widespread *Q. cerris* and the narrow-endemics *Q. castaneifolia* C.A.Mey. (northern Iran), *Q. look* Kotschy (Levant), and *Q. euboica* (Papioann.) K.I.Chr. (Euboea), and as intra-individual dimorphism in western *Q. trojana*; (*iii*) 'Vallonea' lineage exclusive to the eastern Mediterranean *Q. brantii* Lindl., *Q. ithaburensis* Decne., and *Q. macrolepis* Kotschy; and (*iv*) a derived lineage characterizing the western-most species pair *Q. crenata*-*Q. suber* (**Fig. S4-1**).

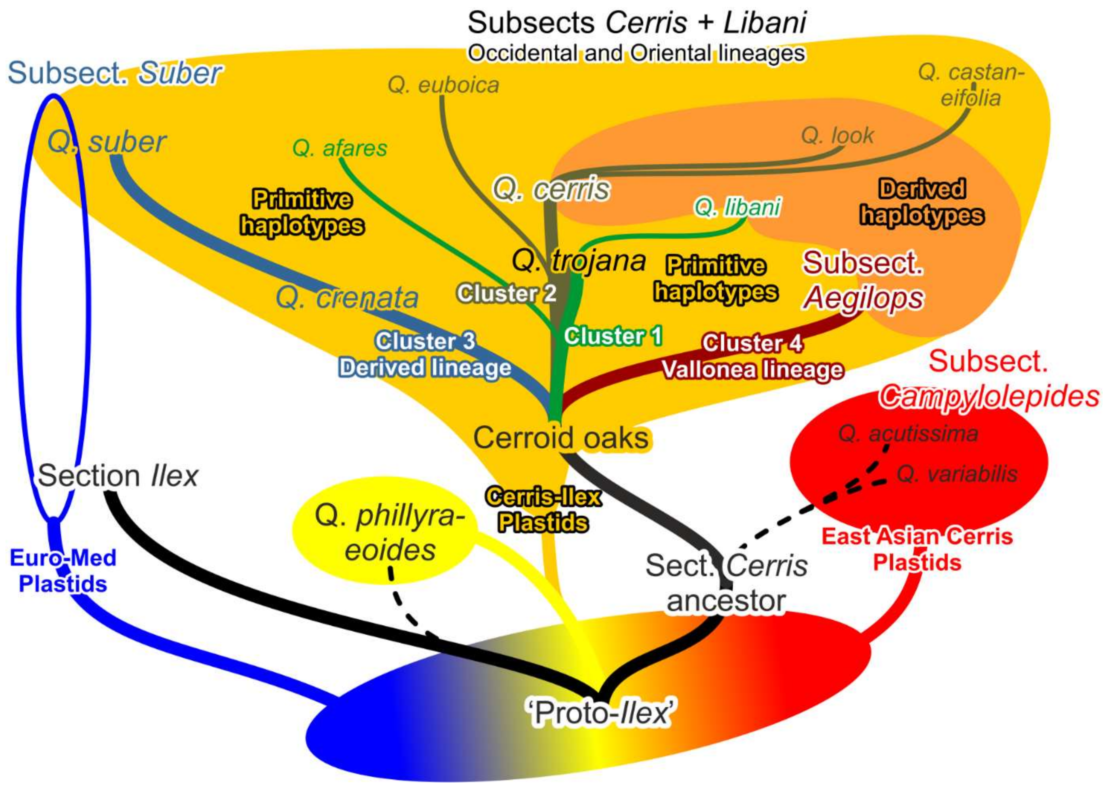

Figure S1-1. Cactus-type tree metaphor summarizing putative reticulate-evolutionary pathways in western Eurasian species of section *Cerris* (modified after Simeone *et al.*, 2018, fig. 6). Lines indicate major nuclear (5S-IGS) lineages, coloured fields shared plastid (*trnH-psbA*) haplotypes.

Stratigraphic note

We follow the latest version (v. 2022/10) of the International Chronostratigraphic Chart (https://stratigraphy.org/; Cohen *et al.*, 2013, updated). The major change has been the re-recognition of lower/early, middle, and upper/late Neogene and Quaternary strata as formally accepted and named units, in some cases between series/epochs and stages/ages (Aquitanian +

Burdigalian = Early Miocene; Langhian + Serravallian = Middle Miocene; Tortonian + Messinian = Late Miocene; Gelasian + Calabrian = Early Pleistocene).

Methods: Biogeography and Climatic Niche

Fossilized Birth-Death analysis

For fossilized birth-death (FBD) analyses, the RAD-seq matrix was reduced to 29 tips, a single tip per species within both sects *Cerris* and *Ilex*, with the exception of *Q. cerris*, for which two individuals were kept that did not group together in any analyses and might represent cryptic species. Loci were retained if they were present in at least 10 individuals. A NEXUS file was exported using the RADAMI package, including 47 additional lines of undetermined positions (coded as "?"), one per fossil included in the FBD analyses.

FBD analyses were conducted in BEAST2 (Bouckaert *et al.*, 2014). Markov chain Monte Carlo (MCMC) runs of 50 million generations each were run from ten independent random starting points on each of three random draws from the uniform distribution of the fossil age ranges. Analyses were conducted using a nucleotide substitution model that allows for rate variation and invariant sites (Γ + I), with the shape parameter (α) and proportion of invariant positions estimated, and four gamma categories. The relaxed log normal clock was used, with the clock rate estimated. Priors were specified using defaults with these exceptions: origin of the clade was set at an initial position of 58 million years (Ma), with a range of 60 to 49 Ma, based on previous results (Hipp *et al.*, 2020) that suggest the origin of subgenus *Cerris* to be close to the origin of the genus; and sampling proportion was estimated with bounds between 0.4 and 0.6, with a starting proportion of 0.45. Clade priors were specified by assigning each fossil in section *Cerris* or *Ilex* to one of thirteen pre-defined clades identified in the maximum likelihood analysis as MRCA priors, enforcing monophyly of the clade including the fossils and extant taxa (*Supplementary Table 3*). The position of the three *Cyclobalanopsis* fossils was designated by assigning all taxa except the *Cyclobalanopsis* fossils to a single "not Cyclobalanopsis" clade. Scripts for exporting data, BEAST2 XML configuration files, and RAD-seq data matrices are all archived in the code repository for this paper (release v0.99-1: https://github.com/andrew-hipp/cerris-fbd; https://doi.org/10.5281/zenodo.7369341), along with output files and instructions for executing the scripts.

Köppen profiles and biome profiles

We used grid-weighted 'Köppen signatures' (Denk *et al.*, 2013; Bouchal *et al.*, 2018; Grímsson *et al.*, 2018), henceforth 'Köppen profiles', to summarize the climate niches occupied by species of *Cerris* and to investigate climate niche evolution within and among subsections of *Cerris* (*main-text Table 1*; *Supplementary Data 3*). A Köppen profile (**Fig. S1-2**) reflects the proportional coverage of the various Köppen-Geiger climate zones (cf. Kottek *et al.*, 2006; Peel *et al.*, 2007) by a modern species based on gridded distribution data.

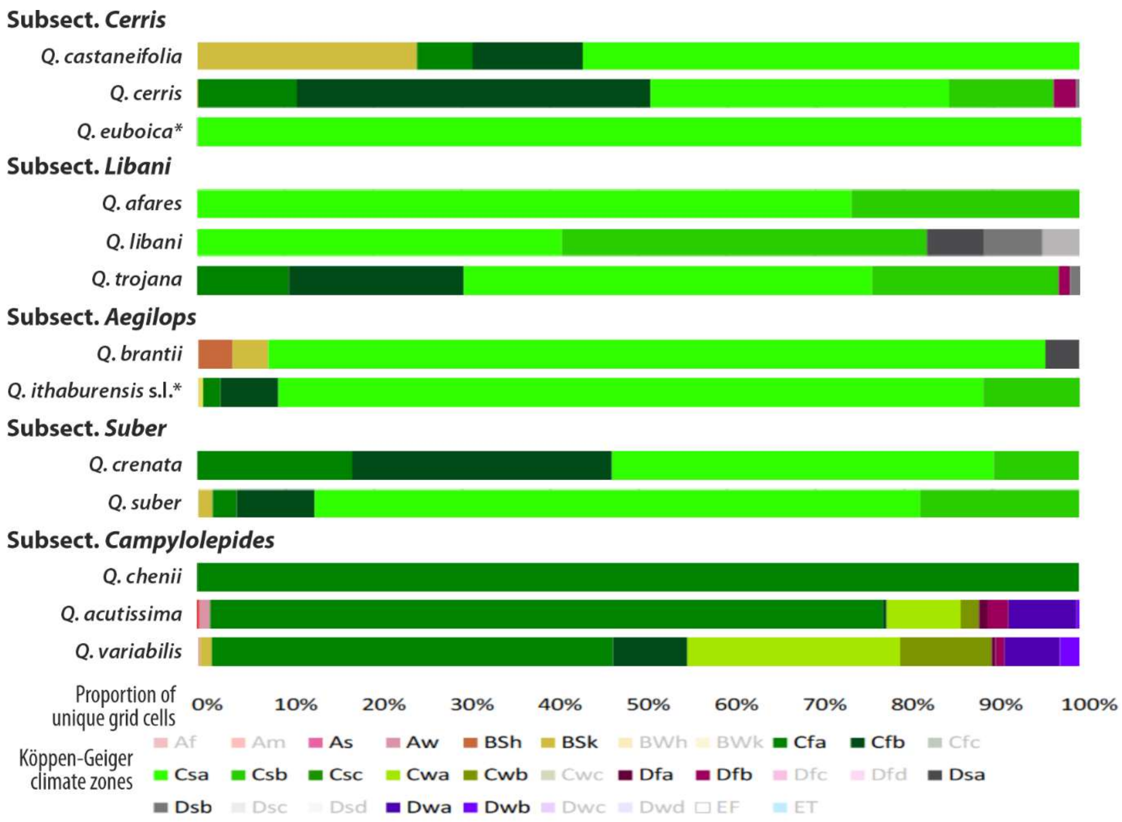

Figure S1-2. Köppen profiles for species of section *Cerris*, sorted by phylogeny. Bottom: earliest diverging subsectional lineage; top: *Cerris* core clade comprising sects *Cerris* and *Libani* (see *Supplementary Data 3* for full details). * GBIF data doesn't distinguish between *Q. macrolepis* and *Q. ithaburensis* (s.str.) and the geographic overlap between the two species has so far not been assessed *in situ*; no SNP data are available for *Q. euboica*, placed based on Simeone *et al.* (2018).

Today, *Cerris* oaks thrive in three main climate types: Subtropical to mild-temperate "warm temperate" *C* climates with average temperatures of the coldest month $T_{min} > -3$ °C and $< +18$ °C, (cool-)temperate "snow climates" with pronounced winter cold, *D*, $T_{min} < -3$ °C, and

tropical "equatorial" *A* climates (marginally, Indochina) with T_{min} > +18 °C. The second letters in the two-/three-letter climate formula used in the Köppen-Geiger classification indicate fully humid (*f*) summer-dry (*s*), or winter-dry (*w*) conditions. In addition, for equatorial climates, the second letter *m* indicates monsoon climates. For details of the three-letter code, see Kottek *et al.* (2006) and Peel *et al.* (2007). Some species further occur in arid *B* climates (*main-text Table 1; Supplementary Data 3*). We opted for using the Köppen-Geiger system, which does not recognize a subtropical zone, instead of the Köppen-Trewartha system because there are no ready-to-use high-resolution Köppen-Trewartha (Trewartha and Horn, 1980) climate grids. Furthermore, the Scotese *et al.* (2014) palaeo-climate zones used to cross-check climate preference of fossil-taxa (see below) correspond to the Köppen-Geiger system, distinguishing five major zones: 'tropical' (= equatorial *A*-climates), 'arid' (= *B*-climates), 'warm (temperate)' (≈ warm temperate *C*-climates), 'cool (temperate)' (≈ *Dxa/Dxb*, snow climates with summer) and 'cold' (continental-dry *D* and *E* climates without summer).

Modern species distributions were connected to fossil distributions by using georeferenced occurrence data for each species, downloaded from the GBIF database (www.gbif.org; *Supplementary Data 3*). Each data set was checked for natural distribution outliers (e.g. specimens from botanical gardens outside the natural distribution range of a species). Published chorological data were used to detect these outliers (e.g. Browicz and Zieliński, 1982; Fang *et al.*, 2009; San-Miguel-Ayanz *et al.*, 2016; Caudullo *et al.*, 2017). The cleaned georeferenced occurrence data were then plotted onto 5 arc minutes Köppen-Geiger grid (1986–2010 data; Rubel *et al.*, 2017) to establish Köppen profiles for all species of section *Cerris* (**Fig. S1-2**); and on major terrestrial biome maps (Olson *et al.*, 2001; **Fig. S1-3**) to assess species' forest biome preferences (full details in *Supplementary Data 3*). For the Köppen-Geiger plots, the georeferenced dataset was filtered so that multiple occurrences in a single grid cell were only counted once (labelled 'unique grid cells' in the diagrams). Likewise, for the biome plots, georeferenced data were filtered so that multiple occurrences with the same coordinates were treated as single occurrences (labelled 'unique localities' in the diagrams).

The georeferenced data and the Köppen-Geiger map with resolution of 5 arc minutes were processed using the 'Sample Raster Values Toolbox' in QGIS Version 3.16.4-Hannover (http://www.qgis.org). The biome shape files were processed using the 'Geoprocessing Tool' in QGIS. The biomes and Köppen-Geiger climates occupied by extant members of section *Cerris* are shown as maps generated with QGIS and as frequency (proportional distribution)

diagrams. The raw coordinate data are archived in EXCEL spreadsheet format (*Supplementary Data 3:* Tabulated Data S3-1).

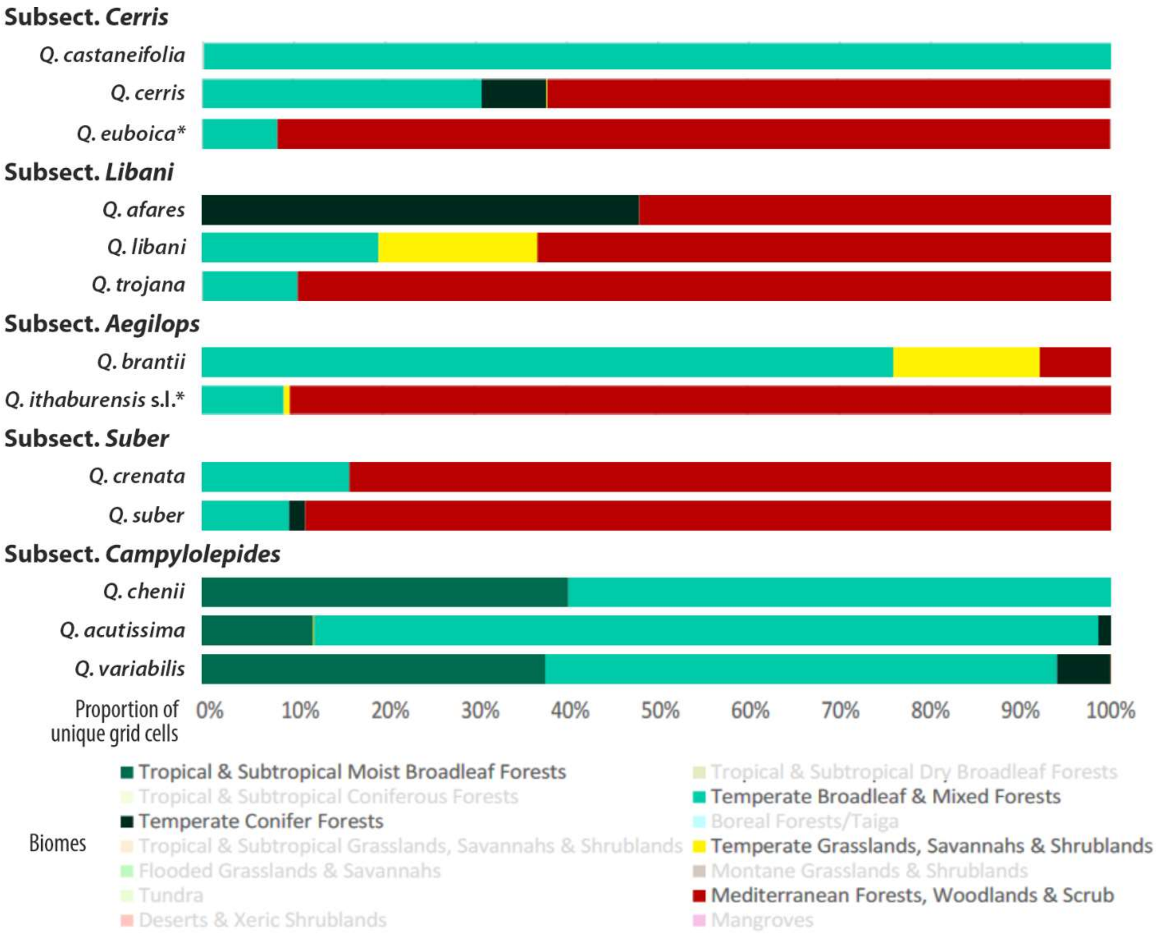

Figure S1-3. Biome profiles for species of section *Cerris*, sorted by phylogeny. See *Supplementary Data 3* for full details. * GBIF data doesn't distinguish between *Q. macrolepis* and *Q. ithaburensis* (s.str.); *Q. euboica* placed after Simeone *et al.* (2018).

In addition, historical climate data (1970–2000) were compiled for 2,779 georeferenced occurrences for members of *Quercus* sect. *Cerris* using WORLDCLIM vers. 2.1 (https://www.worldclim.org/data/worldclim21.html) at a resolution of 30 seconds (ca. 1 km^2, Fick *et al.*, 2017; *Supplementary Data 3*: Tabulated Data S3-3). To characterize the climate envelope of modern-day species, we plotted the mean temperature of the coldest month (MTCM) against precipitation during the winter quarter (PCQ; WORLDCLIM variable BIO19), as well as monthly temperature and precipitation averages (MMT, MMP) and monthly minimal temperatures (MTmin; *Supplementary Data 3*).

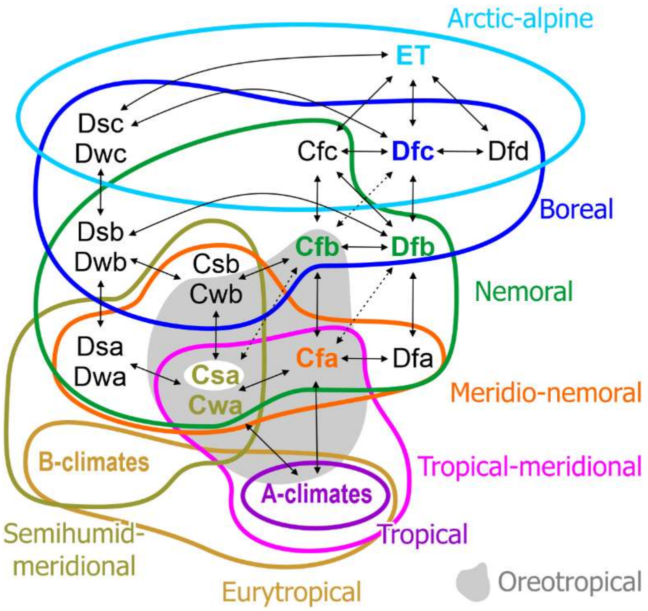

Figure S1-4. Categorisation of plants as vegetation elements based on their 'Köppen signatures' (after Denk *et al.*, 2013; emended by Bouchal *et al.*, 2018, and Grímsson *et al.*, 2018). Arrows indicate modern-day latitudinal/longitudinal and altitudinal transitions (neighbouring climates). Oaks of sect. *Cerris* do not extend into climates inhabited by 'arctic-alpine' vegetation elements; using this scheme they represent 'semihumid-meridional', 'meridio-nemoral' and 'nemoral' vegetation elements.

Maximum likelihood reconstructions of major climate niches and main biomes

Based on the quantitative assessment of biome and climate zone preferences of the modern-day species, we binned extant and fossil species into five basic categories (*Supplementary Table 2*), accounting either for biome or climate zone preferences (*main-text Table 1*; *Supplementary Data 3*). Our generalisation and categorisation make use of the terminology and concepts introduced by Schroeder (1998) and Denk *et al.* (2013; see **Fig. S1-4**) and allow (*i*) direct comparison of biome and climate zones preferences, which are commonly correlated but not synonymous, and (*ii*) relation of quantitative modern-day categorisation qualitatively to our fossil-taxon set. Towards that end, we first defined for each fossil-taxon of section *Cerris* (columns *Biome* and *Major Köppen climate type* in *Supplementary Table 2*) the putative covered biomes: 'Tropical & Subtropical Coniferous Forest', 'Tropical & Subtropical Moist Broadleaf Forest', 'Mediterranean Forests, Woodlands, and Scrubs', and 'Temperate Conifer Forest'. We also specified climate zones for each fossil taxon, distinguishable in the fossil record as five principal combinations: (*i*) *Cf* + *Cw* (may include *Aw* for oldest fossils); (*ii*)

chiefly *Cf*; (*iii*) *Cf/Cw* extending into *Df/Dw*; (*iv*) *Cf* + *Df*; and (*v*) *Cf* + *Cs*. The scoring is based on our knowledge about the fossil floras (place, time, plant association) and general background information about the climatic history of the northern hemisphere (Scotese *et al.*, 2014). We then identified common patterns and assigned the following five categories to the modern-day and fossil-taxa (**Fig. S1-5**; columns *Category according biome*; *Category according climate zone* in *Supplementary Table 2*; matrix *MainClimates* in **Data File S1-1**; characters #10 – biome class, and #11 – climate class).

0–Moist-Subtropical: Associated exclusively with the Tropical and Subtropical Moist Broadleaf Forests biome; fossil species that can be associated with *Cf* climates during global greenhouse phases or at low latitudes, i.e. exclusively within the southern half of the 'warm' zones (≈ modern-day humid subtropics), possibly extending in the extinct 'boreal tropical' palaeo-climate zones in Scotese *et al.* (2014). In case of modern-day *Cerris* oaks, species are associated almost exclusively with summer-moist climates with hot summers (*Cfa*, *Cwa* climates). The only modern species with an accordingly characteristic climate niche is the East Asian *Q. chenii,* firmly restricted to the *Cfa* climate of central-eastern China.

1–Meridio-Nemoral (modified from Denk *et al.*, 2013): Associated with the ecotone between Tropical and Subtropical Moist Broadleaf Forests and Temperate Broadleaf and Mixed Forests biomes. Fossil-species are linked exclusively to *Cf* climates and are placed within Scotese *et al.*'s (2014) 'warm(-temperate)' palaeo-climate zones. A modern-day Meridio-Nemoral species has its main distribution in subtropical to temperate climates with ample precipitation in the hot summer (typically *Cfa* + *Cwa* extending into warm *Cfb/Cwb* variants).

2–Nemoral (cf. Schroeder 1998; Denk *et al.*, 2013): Either restricted to Temperate Broadleaf and Mixed Forests or extending into both Tropical and Subtropical Moist Broadleaf Forests and Temperate Coniferous Forests biomes; fossil species associated with fully humid *Cf* and *Df* climates ranging from 'warm' into 'cool(-temperate)' palaeo-climate zones. Nemoral species are fully temperate species preferring mild climates with ample precipitation during the typically long (~8 months with MTCM > 10 °C) growing season. Key taxon for this category is *Fagus* (beech), which has its climax distribution in fully temperate *Cfb* climates, extending into subtropical *Cfa* lowland and, latitudinally and altitudinally, into cool-temperate *Dfa/Dfb* climates. Fossil assemblages of this category would typically contain broadleaved oaks of subgenus *Quercus* associated with beech and maples with palmate leaves (as found in modern-day species of *Acer* sects *Acer*, *Macrophylla*, *Palmata* p.p., and *Platanoidea*). The only modern-

day *Cerris* oak that qualifies for this category is *Q. variabilis*, being the most widespread species of section *Cerris* with a strong preference for the Temperate Broadleaf and Mixed Forests biome and the full range of summer-humid warm temperate (*Cf*, *Cw*) climates.

3–Meridional (cf. Schroeder, 1998): Generalists tolerating summer-drought, otherwise similar to the Nemoral category. Fossil-species of this category can be associated with Tropical and Subtropical Moist Broadleaf Forests *and* biomes with seasonal drought/ water stress (Mediterranean Forests, Woodlands, and Scrub and Temperate Coniferous Forests biomes); they thrive in fully humid *Cf* as well as summer-dry *Cs* climates. The natural distribution of modern-day Meridional species is restricted to the subtropical belt but characterized by a split preference for both fully Mediterranean lowland climates (unlike in species of the previous categories), summer-dry with hot summers (*Csa*), and their altitudinal, potentially moister successions: summer-dry (*Csb*) or perhumid (*Cfb*) climates with warm summers. In contrast to the species categorized as Full-Mediterranean (next category), they can endure substantial winter cold and frost phases, with some species extending into the boreal, continental, summer-dry snow climates found at mid-latitudes in Western Eurasia. They do not usually form part of coastal vegetation but prefer higher altitudes and the interior.

4–Full-Mediterranean: Summer-drought tolerant specialists restricted to summer-hot and winter-mild biomes and climates. Modern-day species of this category are restricted to Mediterranean Forests, Woodlands, and Scrub biome (or Temperate Coniferous Forests biome: *Q. afares*) and hot, summer-dry *Csa* climates, while only marginally extending into moister biomes and moister (Mediterranean-*Cfa*) or colder (*Csb*) climates. Further, Full-Mediterranean species can be found in hot or cold "steppe" climates (*BSh*, *BSk*).

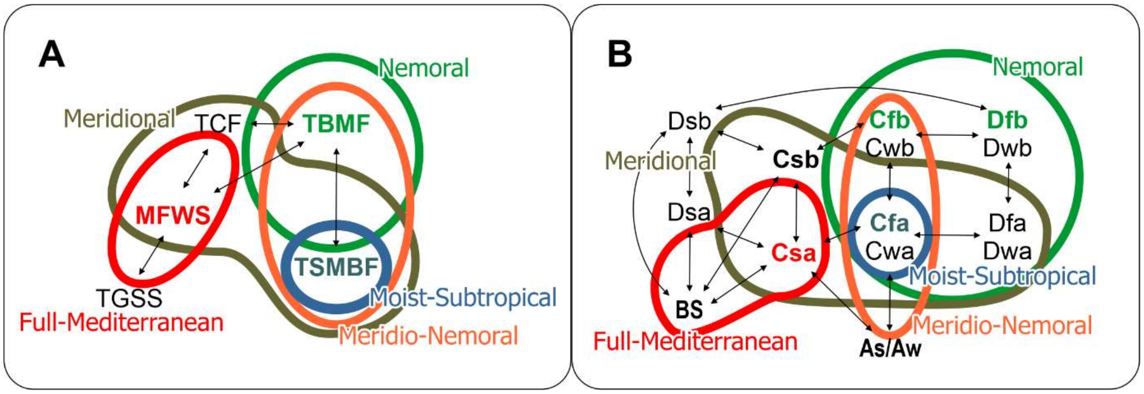

Figure S1-5. Schematic illustrating the biomes (**A**) and climates (**B**) covered by the here used general categories.

Ancestral states of the unordered five-state categorical climate character were reconstructed under the *Mk1* model in MESQUITE v 2.75 (Maddison and Maddison, 2011). We used two different input trees: *(i)* the original dated tree for standard top-down reconstruction of ancestral states, i.e. using only the information scored for the modern-day species; *(ii)* the dated tree with nodes and tips added to account for states of fossil-taxa. Fossil-taxa that could be associated with a distinct branch (lineage) were treated as sister lineages and used to break down the according branch. We used the oldest possible age of the fossil-taxon as age of the putative MRCA, and the youngest possible age to define the MRCA-added-tip distance. The resultant Mesquite-NEXUS file is included in the Github repository/ Zenodo submission (*Supplementary Data 4*: Data File S4-1).

Tests for Inter-Species Gene Flow

Evidence of introgressive hybridisation was investigated using Patterson's *D*-statistic test (Durand *et al.*, 2011) as implemented in IPYRAD (Eaton and Overcast, 2020). The following text describes the rational (background), experimental set-up and summarizes the main results.

Background: Quercus afares *hybrid origins*

The only non-morphological evidence for a hybrid origin of *Q. afares* was the study of Mir *et al.* (2006), which included data from the two postulated (based on morphology and geography) donors of *Q.* × *afares*: the Cerris oak *Q. suber*, molecularly, a distant relative, and the white oak *Q. canariensis,* sym-/parapatric with *Q. afares*, a species from a different subgenus and much different evolutionary and geographic origin (see Denk and Grimm, 2010; see also the Fagaceae phylogenies and data compiled by Zhou *et al.*, 2022). Without including an additional tip as outgroup not involved in the tested hybrid scenario, e.g. *Q. ilex,* Mir *et al.* (2006) were unable to perform a topology test, so their evidence is based on isozyme allele frequencies. The test is particularly interesting because a section *Cerris* × section *Quercus* hybrid is unlikely, at least in natural populations. While the occurrence of (±ancient) inter-sectional hybrids can be assumed for sections of the same subgenus, there has been so far no evidence for mixing between the two main lineages, the exclusively Old World (Eurasian) subgenus *Cerris* and the originally New World (American) subgenus *Quercus* in areas (Eurasia) where they grow sympatrically today or in the past (Denk and Grimm, 2010; Simeone *et al.*, 2018). In contrast, species groups and lineages characterized by sharing of plastid haplotypes such as Mediterranean Cerris and Ilex oaks and subtropical-tropical East Asian cycle

cup (sect. *Cyclobalanopsis*) and Ilex oaks (Simeone *et al.*, 2016, 2018; Zhou *et al.*, 2022), or for which past introgression has been inferred using nuclear data (sects *Ponticae* and *Quercus*, McVay *et al.*, 2017; sect. *Protobalanus* and *Quercus* in western North America, cf. ITS data of Manos *et al.,* 2001) always were members of the same subgenus. Inter-generic reticulation possibly can be found even within major core Fagaceae lineages such as *Notholithocarpus* and American members of subgenus *Quercus* (P. Manos, pers. comm., 2012): subgenus *Quercus* and the western North American relict genera *Notholithocarpus* and *Chrysolepis* share the same plastome lineage (Zhou *et al.*, 2022), possibly including the "Euro-Med" haplotype of *Q. ilex* in section *Ilex* (*Q. ilex* included in Yang et al., 2019; cf. Grimm 2022). In contrast, there is up to date no evidence at all for inter-subgeneric gene flow (past or present) in oaks, despite the fact that e.g. species of section *Quercus* frequently can be found in sympatry with (widespread) species of sections *Cerris* and *Ilex* across Eurasia.

Background: Quercus crenata *hybrid origins*

Hybridisation and introgression are more likely in the case of *Q. crenata,* postulated to be a *Q. cerris* × *Q. suber* hybrid or *Q. cerris* populations introgressed by *Q. suber*, because of its intermediate morphology, specifically the presence of well-developed cork layers in *Q. crenata* (Schwarz, 1936–1939; Pignatti, 1982). Also in this case, broadly-sampled nuclear spacer data did not produce any evidence so far for *Q. cerris*-unique (diagnostic) sequence variants in *Q. crenata*, while strongly supporting an (inclusive) common origin (holophyly, i.e. monophyly in a strict sense; cf. Ashlock, 1971) of *Q. crenata* and *Q. suber* (Denk and Grimm, 2010; Simeone *et al.*, 2018; Piredda *et al.*, 2020; the current study using phylogenomic data from carefully selected placeholder tip-set).

D-*statistics*

To test for introgression and hybridisation using the *D*-statistic tests, one needs SNP data from at least four tips: an outgroup defining the root of the three tested tips (p4); a tip (p3) representing one potentially introgressing/introgressed population or species; and two other tips (p2 and p1) of which one is hypothesized to introgress with p3, the other of which is not. SNPs are subsetted to the included tips and include only binary SNPs that form one of two patterns: ABBA, where A represents one nucleotide and B the other, and the pattern references p1 to p4 in order; or BABA. The relative frequencies of ABBA and BABA are compared, and *Z*-scores on bootstrap-resamples are used to calculate a *P*-value, which we report in the paper using

Holm-correction (see *Methods*) for each *D*-statistic test performed to correct for multiple testing. A significant over-abundance of the ABBA pattern suggests that phylogenetic discordance implied by the SNPs is likely due to introgression between p3 and p2; a significant over-abundance of the BABA pattern suggests introgression between p3 and p1. A non-significant *D*-statistic result suggests that phylogenetic discordance implied by the SNPs can be accounted for as an outcome of incomplete lineage sorting.

Our tests focused on two primary hypotheses: the hybrid origins of *Q. afares* (as proposed in, e.g., Mir *et al.*, 2006, Welter *et al.*, 2012, and Mhamdi *et al.*, 2013) and of *Q. crenata*. We specifically tested for admixture in *Q. afares* and *Q. crenata* because both taxa have been traditionally viewed as (F1) hybrids: *Q. afares* as *Q. suber* × *Q. canariensis* (the latter belonging to sect. *Quercus*; e.g. Mir *et al.*, 2006); *Q. crenata* as *Q. cerris* × *Q. suber.* In addition, we performed follow-up tests for admixture between *Q. ilex* and *Q. suber* (locally sharing *Q. ilex*-unique "Euro-Med" plastid haplotypes in the western Mediterranean; Simeone *et al.*, 2018), *Q. cerris* and *Q. afares*, and among subsections *Aegilops*, *Suber*, and *Libani*.

Methods

RAD-seq data were clustered *de novo* in IPYRAD v 0.9.84 for 34 individuals comprising all section *Cerris* individuals, all *Quercus ilex* individuals, four individuals from section *Cyclobalanopsis*, *Quercus canariensis*, and *Notholithocarpus densiflorus* as an outgroup, using the same clustering thresholds used above but retaining loci with a minimum of four samples, to increase the number of potential loci usable for *D*-statistic tests. Tests were conducted by generating all possible combinations of the outgroups, the first potential introgressing individuals (introgressant) in our dataset (first species listed in each comparison) as taxon p3, the individuals of the second potential introgressant in our dataset as taxon p2, and all other taxa compatible with the pectinate topology used in the *D*-statistic test as taxon p1 (see Durand *et al.*, 2011, fig. 1), with ABBA as the expected dominant pattern under the introgression hypothesis being tested in each case. One test ("canariensis-afares") was limited to 16 tests with a range of possible sisters because the unconstrained test caused errors. *Z*-scores were calculated for each test, and two-tailed *P*-values for *Z*-scores were corrected for multiple test biases using Holm-Bonferroni correction in R, with correction applied separately for each of the ten hypotheses investigated. For all hypotheses performed, effect of the p3 or p1 taxon was investigated by summarizing *D*-stats, *Z*-scores, and corrected p-values for the tests in which each taxon was included. Scripts for performing and summarizing analyses are in the 'D_stats'

subfolder of https://github.com/andrew-hipp/cerris-fbd (https://doi.org/10.5281/zenodo.6595965).

A total of 13 hypotheses are summarized in **Table S1-1**, which summarizes the results over all tests performed for each hypothesis; and **Tabulated Data S1-1**, which summarizes test results for specific focal individuals in either the p3 or p1 position for the tests performed. Summaries are provided by indicating the number of tests performed, and then *D*, *Z*, and Holm-Bonferroni-corrected *P*, all with mean and 95% quantiles, as well as the percent of tests significant at $P \leq 0.01$. Test numbers in **Tabulated Data S1-1** correspond to test numbers reported in *Table 3* of the main text.

Two additional tests were performed but not included in the results or discussion of the main paper for clarity: a test of relative importance of introgression with the two *Q. crenata* individuals included, using all species potentially introgressing with *Q. crenata* as tested above with each of the *Q. crenata* individuals as sister species. This test result is reported in **Tabulated Data S1-1**. The second is a five-taxon ("partitioned") *D*-statistic test evaluating the direction of introgression with *Q. crenata* and potential introgressants. This is reported in **Tabulated Data S1-2**.

Taxa included in D-*statistic tests performed*

The following summarizes what taxa were included in each test performed, and how many topologies (*n*) were evaluated for each test. Details of which individuals were included are in the associated Python scripts (as indexed and detailed in https://github.com/andrew-hipp/cerris-fbd/blob/main/D_stats/finalForPaper/AAA.testsSummarized.md).

- A1 -- cerris-crenata (*n* = 500): *Q. cerris* with *Q. crenata*
    - p4 - *Q. chenii*, *Q. variabilis*, and *Q. acutissima* as outgroups
    - p3 - *Q. cerris,* potential introgressant 1
    - p2 - *Q. crenata,* potential introgressant 2
    - p1 - *Q. suber* as sister species to *Q. crenata*
- A2 -- crenata-cerris (*n* = 2560): *Q. crenata* with *Q. cerris*
    - p4 - *Q. chenii*, *Q. variabilis*, and *Q. acutissima* as outgroups
    - p3 - *Q. crenata,* potential introgressant 1
    - p2 - *Q. cerris,* potential introgressant 2
    - p1 - undefined --- anyone who fits the topology

- B1 -- canariensis-afares (*n* = 16): *Q. canariensis* with *Q. afares*
  - p4 - *Notholithocarpus* as outgroup
  - p3 - *Q. canariensis*, potential introgressant 1
  - p2 - *Q. afares*, potential introgressant 2
  - p1 - *Q. libani* [sister to *Q. afares*]
- B2 -- suber - afares (*n* = 700): *Q. suber* with *Q. afares*
  - p4 - *Q. chenii*, *Q. variabilis*, and *Q. acutissima* as outgroups
  - p3 - *Q. suber*, potential introgressant 1
  - p2 - *Q. afares*, potential introgressant 2
  - p1 - undefined --- anyone who fits the topology
- C1 -- Aegilops-crenata (n = 450 tests): introgression of subsect. *Aegilops* with *Q. crenata*
  - p4 - *Q. chenii*, *Q. variabilis*, and *Q. acutissima* as outgroups
  - p3 - Aegilops oaks *Q. macrolepis*, *Q. brantii*, *Q. ithaburensis* as potential introgressant 1
  - p2 - *Q. crenata,* potential introgressant 2
  - p1 - *Q. suber* as sister species to *Q. crenata*
- C2 -- crenata - afares (*n* = 280 tests): *Q. crenata* and *Q. afares*
  - p4 - *Q. chenii*, *Q. variabilis*, and *Q. acutissima* as outgroups
  - p3 - *Q. crenata* potential introgressant 1
  - p2 - *Q. afares* potential introgressant 2
  - p1 - undefined --- anyone who fits the topology
- C3 -- suber-cerris (*n* = 6400 tests): *Q. suber* and *Q. cerris*
  - p4 - *Q. chenii*, *Q. variabilis*, and *Q. acutissima* as outgroups
  - p3 - *Q. suber*, potential introgressant 1
  - p2 - *Q. cerris*, potential introgressant 2
  - p1 - undefined --- anyone who fits the topology
- D -- Libani-crenata (*n* = 350): subsect. *Libani* with *Q. crenata*
  - p4 - *Q. chenii*, *Q. variabilis*, and *Q. acutissima* as outgroups
  - p3 - *Q. libani*, *Q. trojana*, or *Q. afares* as potential introgressant 1
  - p2 - *Q. crenata*, potential introgressant 2
  - p1 - *Q. suber* as sister species to Q. crenata
- E1 -- cerris-afares (*n* = 270): *Q. cerris* with *Q. afares*
  - p4 - Aegilops oaks as outgroups: *Q. macrolepis*, *Q. brantii*, *Q. ithaburensis*
  - p3 - *Q. cerris*, potential introgressant 1
  - p2 - *Q. afares*, potential introgressant 2
  - p1 - *Q. libani* 628 - *Q. trojana* 585
- E2 -- cerris-afares | libani (*n* = 90): *Q. cerris* with *Q. afares*, *Q. libani* as p1
  - p4 - Aegilops oaks as outgroups: *Q. macrolepis*, *Q. brantii*, *Q. ithaburensis*
  - p3 - *Q. cerris*, potential introgressant 1
  - p2 - *Q. afares*, potential introgressant 2
  - p1 - *Q. libani* 628

- E3 -- cerris-afares | trojana (n = 90): *Q. cerris* with *Q. afares*, *Q. trojana* as p1
    - p4 - Aegilops oaks as outgroups: *Q. macrolepis*, *Q. brantii*, *Q. ithaburensis*
    - p3 - *Q. cerris*, potential introgressant 1
    - p2 - *Q. afares*, potential introgressant 2
    - p1 - *Q. trojana* 585
- E4 -- cerris-trojana (n = 90): *Q. cerris* with *Q. trojana*, *Q. libani* as p1
    - p4 - Aegilops oaks as outgroups - Q. macrolepis, Q. brantii, Q. ithaburensis
    - p3 - *Q. cerris*, potential introgressant 1
    - p2 - *Q. trojana* 585
    - p1 - *Q. libani* 628
- F -- suber-ilex (*n* = 6,240): *Q. suber* with *Q. ilex*
    - p4 - sect. *Cyclobalanopsis* as outgroup
    - p3 - *Q. ilex*, potential introgressant 1
    - p2 - *Q. suber*, potential introgressant 2
    - p1 - any species sister to p2 w/ respect in context of p4 and p3 used
- Supplemental Test 1 – crenata vs. crenata (*n* = 165)
    - p5 - *Q. chenii*, *Q. variabilis*, and *Q. acutissima* as outgroups
    - p3_1 = p4 - varies (see below)
    - p3_2 = p3 - varies (see below)
    - p2 - *Q. crenata*, potential introgressant 2
    - p1 - *Q. suber* as sister species to *Q. crenata*
- Supplemental Test 2 – 5-taxon *D*-statistic tests (n = 3)
    - p5 - *Q. chenii*, *Q. variabilis*, and *Q. acutissima* as outgroups
    - p3_1 = p4 - varies (see scripts)
    - p3_2 = p3 - varies (see scripts)
    - p2 - *Q. crenata* potential introgressant 2
    - p1 - *Q. suber* as sister species to *Q. crenata*

Results: summary of D*-statistic tests*

Four-taxon *D*-statistic tests (**Table S1-1**) provide no evidence of admixture between *Q. afares* and its two putative parents (*Q. canariensis* and *Q. suber*). All tests for introgression with *Q. canariensis* are non-significant. Many of the tests for introgression with *Q. suber* suggested significant results, but they all have a member of subsection *Aegilops* sister to *Q. afares*. Follow-up tests for introgression between subsections *Aegilops* and *Suber* as well as *Libani* and *Suber* suggest phylogenetic discordance in relative placement of these three subsections, rather than species-level introgression involving *Q. afares* and *Q. suber*. The tests for introgression between *Q. cerris* and *Q. crenata* are all significant when *Q. cerris* is in the P3 position; the vast majority are significant with *Q. crenata* in the P3 position, and always when a subsection *Aegilops* species is sister to *Q. cerris*. There is inconsistent evidence of

introgression between *Q. cerris* and *Q. afares* that varies depending on which *Q. cerris* individuals are included in the test: unknown provenance for some of the samples included makes generalisation difficult. There is no evidence for nuclear admixture between *Q. suber* and *Q. ilex*, as reported previously based on plastome data (Simeone *et al.* 2018) (**Table S1-1**).

D-statistic tests recover strong and consistent support for admixture between *Q. cerris* and *Q. crenata* (100% of tests significant at $p \leq 0.01$; *main-text Table 3*). Five-taxon *D*-statistic tests (Supplemental Test 2) suggest that the direction of past or recent introgression has been from *Q. cerris* to *Q. crenata*, as the alleles that are shared by *Q. cerris* and either section *Aegilops* or section *Libani* are also significantly shared with *Q. crenata* relative to *Q. suber* (Tabulated Data S1-1). There is also strong and consistent support for admixture between *Q. cerris* and *Q. afares* when *Q. libani* is used as the sister to *Q. afares* (Test E2: 98.9% of tests significant; main-text Table 3). When *Q. trojana* (Italy, Balkans, Turkey) is used as the sister to *Q. afares* (endemic to Tunisia, N. Algeria), only the two westernmost *Q. cerris* individuals show significant levels of admixture: OAK-MOR-591 (Latium, Italy) and OAK-MOR-736 (Samsun, Turkey; 88.9% of tests significant; Test E3, Tabulated Data S1-1). There is minimal evidence of admixture/ introgression between *Q. ilex* and *Q. suber* (Test F: 19.0% of tests significant; Table 3). Partitioning the section *Aegilops* (p3)-*Q. crenata* (p4) test (Test C1) by individuals in the p3 position indicates that two of the *Q. ithaburensis* individuals (OAK-MOR-599, OAK-MOR-735; Levante) may have been weakly involved in introgression with *Q. crenata* (endemic to Tyrrhenian side of Italy; 61.5–88.0% of tests significant) in the past, while the other section *Aegilops* individuals (SE. Italy, Turkey, Israel) were more strongly involved (99.3–100% of tests significant; **Tabulated Data S1-1**). Partitioning the "crenata vs. crenata" test (Supplemental Test 1) by p3 individuals demonstrates that *Q. crenata* TUS13-003 (OAK-MOR-593), which falls sister to *Q. crenata* 1977-541 (OAK-MOR-986) + *Q. suber*, has been disproportionately affected by admixture from the *Cerris* core clade (**Tabulated Data S1-1**).

Tests involving *Q. afares* showed no evidence of introgression with *Q. canariensis* in any topologies (Test B1, **Table S1-1**), but high variability in test results for *Q. suber*, with *P*-values ranging from <0.001 to 0.859 (Test B2, **Table S1-1**). Subsequent tests between subsections *Aegilops* and *Suber* (C1, C2, C3) as well as subsection *Libani* and *Q. crenata* (D) partitioned tests ("cerris – afares | libani"; "cerris – afares | trojana") show that some ABBA-patterns probably represent a deeper history of introgression or other source of imbalanced shared ancestral alleles rather than recent introgression.

Table S1-1. Summary of *D*-statistic (ABBA-BABA) tests of introgression. Taxa used are summarized in the methods section. Introgression hypotheses tested are numbered following **Tabulated Data S1-1**. Column headers: # tests—number of topologies that were tested corresponding to the hypothesis tested; *D*—Patterson's *D* statistic, mean and 95% quantile over tests; *Z*—Z-score, mean and quantile over tests. *P*—Holm-Bonferroni-corrected p-values, mean and quantile over tests; % significant—percent of tests with Holm-Bonferroni corrected P-values ≤ 0.01.

	**# tests**	***D***	***Z***	**P (Holm-corrected)**	**% significant (α = 0.01)**	**Notes**
***Q. crenata* admixture tests**						
A1. Putative parent: Q. *cerris* (*Q. cerris* as P3)	500	0.384 [0.294,0.526]	22.227 [12.003,43.01]	<0.001 [<0.001,<0.001]	100.0	
A2. Putative parent: Q. *cerris* (*Q. crenata* as P3)	2560	0.133 [-0.234,0.420]	10.746 [0.322,30.558]	0.059 [<0.001,0.747]	82.9	[1]
***Q. afares* admixture tests**						
B1. Putative parent: Q. *canariensis*	16	-0.057 [-0.208,0.044]	1.276 [0.552,2.151]	0.270 [0.032,0.581]	0.0	
B2. Putative parent: Q. *suber*	700	0.056 [-0.046,0.174]	2.665 [0.178,6.825]	0.191 [<0.001,0.859]	34.4	[2]
**Subsect. *Aegilops* - subsect. *Suber***						
C1. *Aegilops* - Q. *crenata*	450	0.124 [0.026,0.193]	5.412 [0.324,10.046]	0.055 [<0.001,0.746]	88.0	
C2. Q. *crenata* - Q. *afares*	280	0.099 [-0.222,0.343]	7.669 [0.180,20.160]	0.110 [<0.001,0.857]	72.5	[3]
C3. Q. *suber* - Q. *cerris*	6400	0.037 [-0.057,0.141]	2.576 [0.066,6.964]	0.237 [<0.001,0.947]	39.7	[4]
**Subsects. *Libani* - *Suber***						
D. All possibilities	350	0.310 [0.195,0.424]	17.058 [7.707,30.821]	<0.001 [<0.001,<0.001]	100.0	
Q. cerris* - *Q. afares						
E1. All possibilities	270	0.159 [0.026,0.373]	8.046 [1.268,21.956]	0.022 [<0.001,0.205]	85.2	
E2. Q. *libani* as sister (P1)	90	0.212 [0.101,0.388]	10.754 [2.943,23.817]	<0.001 [<0.001,0.003]	98.9	
E3. Q. *trojana* as sister (P1)	90	0.100 [0.023,0.265]	4.631 [0.466,13.983]	0.063 [<0.001,0.652]	58.9	[5]
E4. Q. *cerris* - Q. *trojana*	90	0.095 [0.023,0.200]	4.455 [1.107,7.902]	0.020 [<0.001,0.269]	83.3	
Q. suber* - *Q. ilex						
F. All possibilities	6240	0.057 [-0.014,0.134]	1.792 [0.180,3.660]	0.177 [<0.001,0.857]	19.0	

Notes: [1] All tests with an *Aegilops* species in P1, sister to *Q. cerris*, are significant; 59.0% of other tests are significant as well. [2] Only tests with an *Aegilops* species in P1, sister to *Q. afares*, are significant. [3] All tests with an *Aegilops* species in P1, sister to *Q. afares*, are significant; 30.6% of other tests are significant as well. [4] 99.5% of the significant tests have an *Aegilops* species in P1, sister to *Q. cerris*. [5] 82.1% of the significant tests involve admixture with *Q. cerris* TUS13-002 (Italy) or Avishai 412 (cultivated, Israel, provenance unknown)

Against the background of the FBD-dated tree (main-text Fig. 4), the fossil and niche history (following sections), the *D*-statistics point to ancestral gene flow from the precursors of subsection *Cerris* into those of subsection *Libani,* especially in the central Mediterranean region. We interpret this result as a likely outcome of introgression during the crown diversification of the Western Eurasian *Cerris*, resulting in phylogenetic discordance regarding resolution of the subsections rather than recent introgression.

However, there is evidence for more recent geographically structured introgression that bears additional study with more samples. We observe generally higher and more discriminative *D*-values for admixture when *Q. libani* is used than when *Q. trojana* is used as p4 in the test. The most involved *Q. cerris* individuals in the latter test (E3) are OAK-MOR-591 from Italy ($D \geq 0.22$; 89% of tests significant; geographically closest *Q. cerris*) and, to nearly the same level, the northern Turkish OAK-MOR-736 ($D \geq 0.18$; 89% of tests significant; **Tabulated Data S1-1**). These two individuals furthermore constitute the subsection *Cerris* subclade that includes also the Iranian *Q. castaneifolia*, a species with a distinctly primitive (potentially ancestral within section *Cerris*) leaf morphology not unlike *Q. afares* (following section) and the widespread fossil-species *Q. kubinyii*.

The *D* estimates for *Q. crenata* differ considerably from those of *Q. afares.* We find low to high levels of admixture for all tested scenarios (**Table S1-1**), continuously increasing along the phylogenetic tree and peaking – as in the case of *Q. afares* – in the Italian and northern Turkish individuals of *Q. cerris*. At least two reticulation scenarios might explain such a result:

Scenario 1—*Quercus crenata* and its precursors, representing the first diverging and westernmost section *Cerris* lineage (*main-text Figs 2–3*), were repeatedly affected by introgression when newly diverging eastern Mediterranean lineages radiated. The *D*-statistic results would thus reflect in this scenario a history of gene flow with other sympatric western Eurasian subsections.

Scenario 2—Alternatively, recent introgression of *Q. cerris* with sympatric *Q. crenata* in its western range may account for the observed *D*-statistics. The inferred admixture from other lineages under this scenario would reflect patterns shared by *Q. cerris* and its sister lineage, subsection *Libani* (*D*-values approaching the level as seen in southeasternmost *Q. cerris* individuals from the Levante) and the phylogenetically more distant subsection *Aegilops*.

We consider Scenario 2—modern-day introgression from *Q. cerris* into *Q. crenata*—the less likely: the partitioned *D*-statistics identified OAK-MOR-593 as the *Q. crenata* individual most affected by admixture from outside subsection *Suber*. Yet this individual exhibits relatively low root-tip distance within its clade (*main-text Fig. 1*). We would expect relatively recent introgression to introduce tree-incompatible signal into the RAD-seq data, inflating the tip branch length beyond that observed in the less-impacted second *Q. crenata* individual, OAK-MOR-986. We consequently consider either (1) ancient introgression among the precursors of modern subsections of west Eurasian *Cerris* or (2) demographic processes that result in imbalanced maintenance of ancestral polymorphisms to be a more plausible explanation for the significant *D*-statistic results.

Discussion: Past and recent gene flow in cork oaks

The radiation of Western Eurasian *Cerris* likely involved reticulation. Our analysis of introgression suggests that a history of gene flow influenced the topology of the Western Eurasian *Cerris* clade. Our *D*-statistic tests were designed to differentiate between incomplete lineage sorting and introgression as explanations of phylogenetic discordance implied by SNPs. As evidenced by statistically clear introgression results, species comprising the crown of the Western Eurasian *Cerris* clade were interbreeding at ca. 24–20 Ma, just as *Q. cerris* and *Q. crenata* appear to be introgressing with co-occurring species (until) today (**Table S1-1**). In contrast, our analyses also indicates that *Q. afares*, hypothesized to be a species of hybrid origin, shows no signs of admixture from *Q. canariensis* nor *Q. suber*. This aligns with the lead-author (Denk's) field observations, which suggests that *Q. afares* is not morphologically intermediate between the two putative parents. Rather, it has the vegetative characters typical of sect. *Cerris*—leaf indumentum, texture, and dentition—as well as acorn cups and pollen typical of section *Cerris*. There is, in our view, no morphological character that allies *Q. afares* with the distantly related *Q. canariensis* of section *Quercus*. There may of course be localized gene flow between *Q. afares* and *Q. suber*: in the lead author's experience, *Q. suber* form larger and more elongate leaves at higher elevations in Tunisia, where it becomes sympatric with *Q. afares*. Further in-depth, broad-sampled studies are needed on the genetic and morphological structure of the *Cerris* core clade.

Taxonomy and Previous Schemes

Some of the clades recovered from the RAD-seq data in the present study have previously been recognized as infra-sectional groups. Camus (1936–1938) united the East Asian members of section *Cerris* within her subsection *Campylolepides*. *Quercus suber* has previously been accommodated within its own subsection *Suber* (Menickij, 1984). Nevertheless, the majority of clades recognized here do not correspond to previously recognized infrageneric taxa (*main-text Table 2*) but agree with the 5S-IGS-defined species groups in Simeone *et al.* (2018; **Fig. S1-1**) and are here treated as subsections. The discrepancy between morphological groupings recognized previously and the clades recognized in this study on molecular grounds may represent a combination of convergence (parallelism; **Fig. S1-6, Table S1-2**) among clades and ecological divergence within clades.

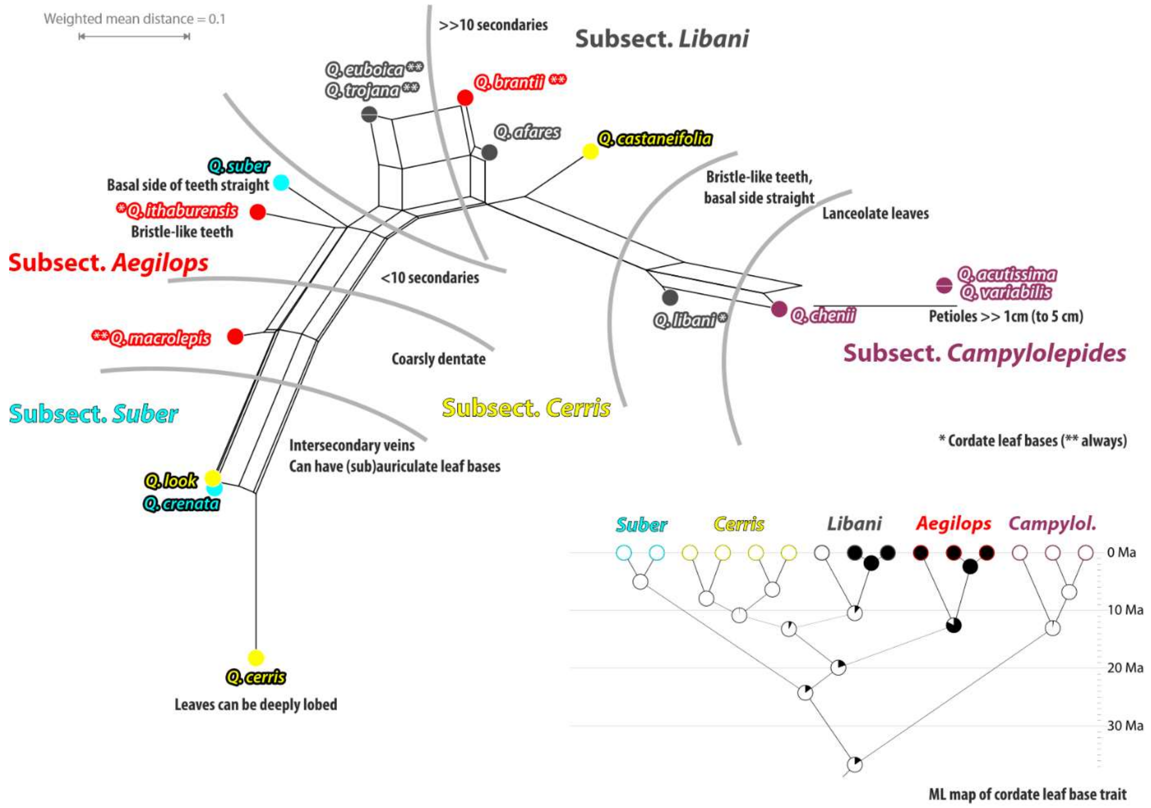

Figure S1-6. Neighbour-net splits graph inferred from pairwise (weighted) distances calculated from a leaf-morphological matrix (**Data File S1-1**; cf. **Table S1-2**). Note the poor phylogenetic sorting (subsections represent molecular clades inferred based on nuclear data) of leaf-morphological groups (species being part of the same neighbourhood) in spite of the relative tree-like graph. Bottom left: Maximum likelihood mapping of the cordate leaf base trait on the dated phylogeny. For terminology, see **Figure S1-7**.

Subsection Suber

Although the cork oak *Q. suber* is generally considered an evergreen tree, and therefore has been associated with section *Ilex* by Menickij (1984), it behaves differently from fully evergreen oak species. Its leaves are thinner and there is a tendency to shed most of the leaves in spring or early summer as response to early moisture stress, giving the tree a pronounced deciduous aspect (Schwarz, 1936–39; Escudero and del Arco, 1987; le Hardÿ de Beaulieu and Lamant, 2010). Menickij (1984) speculated (translation of the Russian original 2005, p. 444) "*The only species of the subsection* Suber *– the Mediterranean* Q. suber *– gives the impression of an intersectional hybrid between some initial species of the preceding subsection* [the subsection *Heterobalanus*; = sect. *Ilex*, the Ilex oaks] *and species of the* Q. aegilops *group (section* Cerris*).*" Indeed, *Q. suber* has a number of highly specialized ecological adaptations. Most conspicuous is the thick cork layer of the stem, which protects the trees from wildfires. Further, the sudden abscission of leaves in dry periods is an effective adaptation to seasonal dry climates, and the plastic cycle of fruit maturation may be a response to changeable availability of nutrients and water. A thinner cork layer is also produced by *Q. crenata*, which our analysis resolves as the only other member of the clade comprising *Q. suber*. This cork layer has been one reason for assuming that *Q. crenata* was a hybrid between *Q. suber* and *Q. cerris* (Schwarz, 1936–1939; Pignatti, 1982). In sum, the now available data point to an ancestor-descendant relationship, with *Q. suber* representing a highly adapted species that may have evolved from a *Q. crenata*-like ancestor in course of niching (cf. *main-text Fig. 1*; *Supplementary Data 3*; Simeone *et al.*, 2018, figs 5–6). **Plate S1-1** illustrates the ancestral-primitive (plesiomorphic) character of *Q. crenata* leaves in comparison to one of the earliest and widespread leaf fossils that can be assigned to section *Cerris*: *Q. kraskinensis* Pavlyutkin. The disparate partitioned *D*-statistics results (**Tabulated Data S1-2**) could result from an ancestor-descendant relationship. Being a recent and strongly bottlenecked offshoot of the subsection *Suber* lineage, we find no evidence for past or recent admixture in the *Q. suber* individuals from outside the lineage. The statistically significant *D*-values observed for the *Q. crenata* individuals may reflect retained ancestral polymorphism inherited from the subsection's precursors as well as a higher susceptibility for introgression of this less evolved and niched sibling (*main-text Fig. 4*; **Table S1-1**; **Tabulated Data S1-1, S1-2**).

Subsection Aegilops

The 'Vallonea-oaks', subsection *Aegilops*, share several morphological similarities (**Fig. S1-6**; Avishai, 2016). The definition of the Vallonea-oaks has changed through time (*main-text Table 2*). The concept of subsect. *Aegilops* proposed here, including only *Q. brantii*, *Q. ithaburensis*, and *Q. macrolepis* (see also Schwarz, 1936), is supported both by our RAD-seq dataset and by 5S-IGS differentiation patterns (Denk and Grimm, 2010; Simeone *et al.*, 2018). The species share unique 5S-IGS variants not found in any other species of section *Cerris*. Their nuclear SNP patterns show a high coherence (well-developed, unambiguously supported root branch despite substantial tip divergence, *main-text Fig. 1*).

Cerris core group: *Subsection* Libani

Ørsted (1871) and later Schwarz (1936) and Menickij (1984) united the East Asian and western Eurasian fully deciduous species of section *Cerris* with a prominent mucronate leaf margin (Fig. S3-2) in the same section or subsection. Likewise, most monographers considered *Q. afares* and *Q. castaneifolia* as belonging to the same section/ subsection (*main-text Table 2*; cf. **Fig. S1-6**), associations that can be rejected based on nuclear data (Simeone *et al.*, 2018; this study). In our analysis, *Q. afares* is resolved in a clade together with *Q. libani* and *Q. trojana*; these three species share a lineage of 5S-IGS variants ('Oriental lineage', Cluster 1 in Simeone *et al.*, 2018; cf. **Fig. S1-1**) not found in subsection *Cerris* as defined below. As in the case of nuclear-encoded ribosomal spacer data (Denk and Grimm, 2010; Simeone *et al.*, 2018), we found no evidence for a white oak parentage in *Q. afares* (**Table S1-1**; **Tabulated Data S1-1**).

Cerris *core group: Subsect.* Cerris

Schwarz (1936) suggested a close relationship between the geographically isolated and ecologically narrow *Q. castaneifolia* and *Q. cerris*. Our data demonstrate that the narrow endemic *Q. look*, traditionally associated with the Vallonea-oaks (subsect. *Aegilops*), also belongs to subsection *Cerris*. As seen in the full-tip ML tree (*main-text Fig. 1*), the genetic variation found in *Q. cerris* encompasses its two endemic siblings, in striking analogy to earlier 5S-IGS data ('Occidental lineage', Cluster 2 in Simeone *et al.*, 2018). Thus, subsect. *Cerris* represents a case example for the retention of ancestral morphologies in locally isolated populations leading either to the formation of relict species (*Q. castaneifolia*) or to highly adapted (*Q. look*) Mediterranean species. Further in-depth nucleome analyses are likely to

reveal cryptic or previously unrecognized species (pseudo-cryptic species) in *Q. cerris* and/or recurrent ancient hybridisation events in subsection *Cerris*, probably involving members of subsection *Libani* (5S-IGS dimorphism in *Q. trojana*; Simeone *et al.*, 2018; cf. Test E4: very low *D*-values, 66–89% of the tests statistically significant for introgression between *Q. cerris* and *Q. trojana*).

Species not yet studied

The only section *Cerris* species not included in the present study, *Q. euboica*, has recently been investigated using one chloroplast and one nuclear marker (Simeone *et al.*, 2018). Traditionally considered a subspecies of *Q. trojana*, the only species of section *Cerris* with individuals showing a prominent 5S-IGS dimorphism, *Q. euboica* appeared to be morphologically intermediate between *Q. cerris* and *Q. trojana* (**Fig. S1-6**). Thus, *Q. euboica* clearly is a member of the *Cerris* core group established here. Based on the available molecular data, its taxonomic placement remains ambiguous, belonging either to subsection *Libani* or *Cerris*, in analogy to the geographic isolates *Q. castaneifolia* and *Q. look*. Further in-depth studies are needed to assess the amount of secondary homogenisation because of sub-recent hybridisation with or without introgression by *Q. cerris*.

Evolution of modern leaf morphologies in cork oaks

No known leaf fossils resemble the modern cork oak, *Q. suber*. The only potential fossil record for this lineage comes from silicified wood from Late Miocene Portugal (de Carvalho, 1958; Mai, 1995) that closely resembles the semi ring-porous wood with large rays of modern *Q. suber* (Sousa, 2009). Similarly, there is no convincing fossil record for subsection *Aegilops* (Avishai, 2016), which diverged by the end of the Early Miocene (*main-text Fig. 3*). Simeone *et al.* (2018) investigated chloroplast differentiation within section *Cerris* and found evidence for lineage-mixing between *Q. ilex* (*Quercus* sect. *Ilex*) and *Q. suber* in the western Mediterranean (*Q. suber* with “Euro-Med” haplotypes, representing a plastome lineage only found in western *Q. ilex*; Simeone *et al.*, 2016; Vitelli *et al.*, 2017), introgression that is not evidence in our nuclear analysis (→ *Tests for Inter-Species Gene Flow*) perhaps because it is sufficiently ancient. In view of the absence of a reliable fossil record of *Q. suber* prior to the Late Miocene, the sister group relationship with *Q. crenata* with estimated divergence during or after the Middle Miocene, and the highly specialized ecological niche of *Q. suber*, it appears that

Q. suber is substantially younger than the early Cenozoic origin hypothesized for this species by Magri *et al.* (2007).

The *Cerris* core clade diversified from the Middle Miocene onwards. Subsection *Cerris* includes three morphologically distinct species (excluding *Q. euboica;* but see Simeone *et al.*, 2018). While *Q. castaneifolia* leaves are similar to leaf morphotypes from Middle Miocene strata of Western Eurasia, *Q. cerris* has the most variable and deeply lobed leaves among modern members of section *Cerris* (*main-text Fig. 5*). Its sister lineage, subsection *Libani*, is characterized by relatively primitive leaf morphologies—narrowly elliptic with triangular teeth or reduced teeth with long bristles—and includes a species of possible hybrid origin (*Q. trojana*; Simeone *et al.*, 2018). While lobed leaves are exclusively found in *Q. cerris*, their fossil record extends only to the Pliocene/Pleistocene (Denk *et al.*, 2021a). High morphological intraspecific and intraindividual variability of leaves and cups in *Q. cerris* is associated with notable intra-specific nuclear variation, perhaps pointing to pseudo-cryptic speciation (*main-text Figs 1, 3*). *Quercus cerris* is the only species of the section that extends into northern Europe as an invasive species (de Rigo *et al.*, 2016). Considering its natural distribution (Fig. 2) and high morpho-logical, ecological (*Supplementary Data 3*) and genetic variability, contemporary *Q. cerris* may represent a highly reticulate species conglomerate with roots in (Late) Miocene radiations (*main-text Fig. 3*; MRCA of *Q. cerris* = MRCA subsect. *Cerris*). Further in-depth, broad-sampled studies are needed on the genetic and morphological structure of the Cerris core clade.

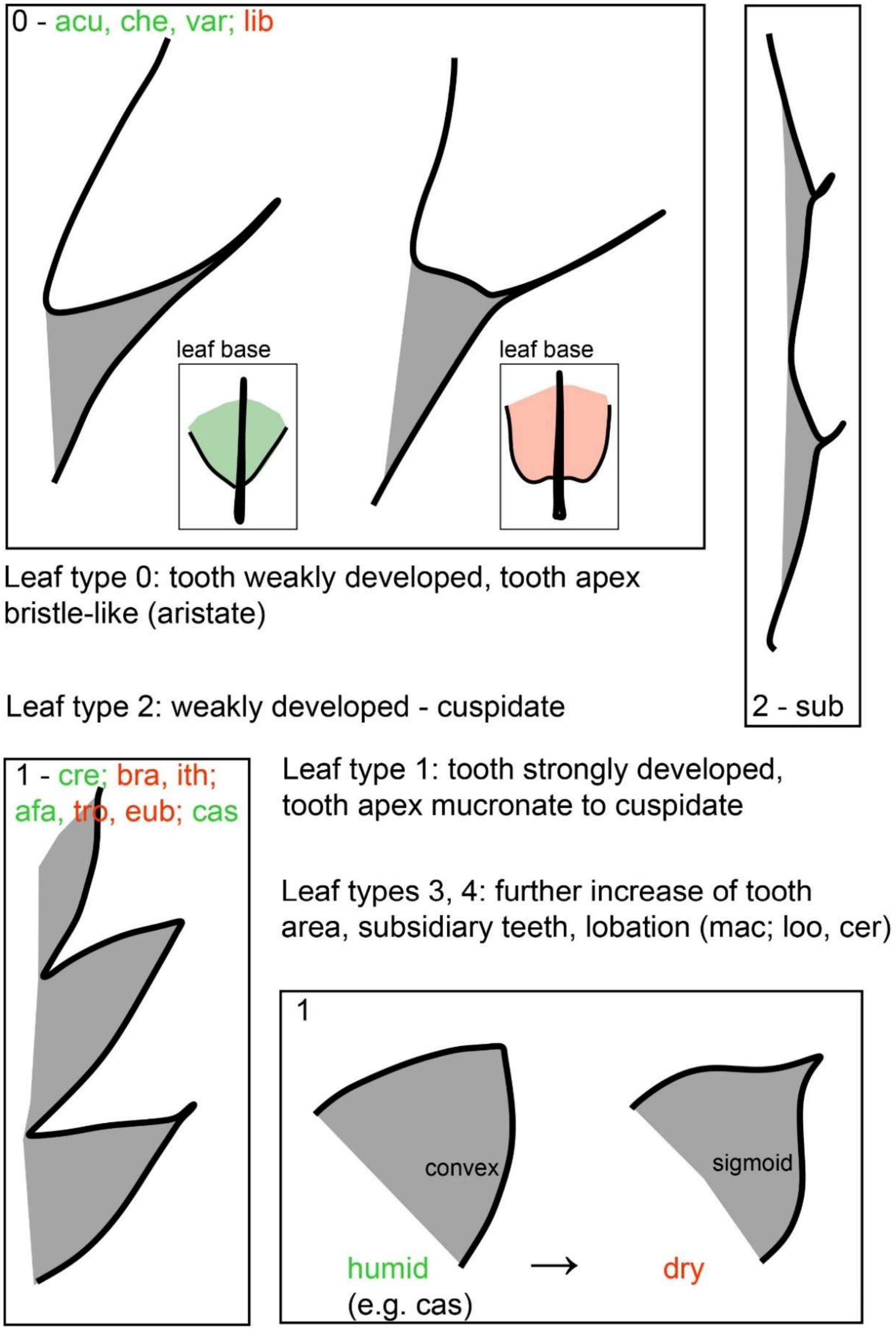

Figure S1-7. Classification of general leaf types found in section *Cerris* introducing the main discriminative features scored for the morphological matrix included in **Data File S1-1** (cf. **Table S1-2**). Specific character suites (see also **Fig. S1-6**) were used to assign leaf fossils to clades to date the phylogeny (see main text). Species names are abbreviated by the first three letters.

Table S1-2. Leaf traits scored for the morphological matrix included in **Data File S1-1**.

Character	[1]	1	2	[3]	3	4	[5]	5
Species	[Leaf size (length, cm)]	**Leaf size**	**Leaf organisation**	**Secondary veins**	**Secondary veins**	**Intersec. Veins**	[Petiole]	**Petiole**
Q. acutissima	8-19	**Large**	Simple	13-18	>>10	Absent	>>1cm (to 5 cm)	**Long**
Q. chenii	7-12	Medium	Simple	12-16	>>10	Absent	1cm (to 1.5 cm)	Short
Q. variabilis	8-15(-20)	**Large**	Simple	13-18	>>10	Absent	>>1cm (to 5 cm)	**Long**
Q. crenata	4-9	Medium	Simple	5-8	<10	**Present**	1-1.5 cm	Short
Q. suber	3-7	Small	Simple	5-6	<10	Absent	0.5-1 cm	Short
Q. brantii	6-10(-13)	Medium	Simple	8-14	>>10	Absent	0.5-2 cm	Short
Q. ithaburensis	4-9	Medium	Simple	5-8	<10	Absent	1-2 cm	Short
Q. macrolepis	5-9	Medium	Simple	5-8	<10	Absent	1-3.5 cm	Medium
Q. afares	7-14	Medium	Simple	8-17	>>10	Absent	1-2 cm	Short
Q. libani	7-12	Medium	Simple	11-16	>>10	Absent	0.8-2 cm	Short
Q. trojana	3-8(-10)	Medium	Simple	9-10	c.10	Absent	0.5-1 cm	Short
Q. euboica	5-9(-11)	Medium	Simple	8-10	c.10	Absent	0.5-1 cm	Short
Q. castaneifolia	10-20	**Large**	Simple	8-14	>>10	Absent	1-2.5 cm	Short
Q. look	5-7.5	Small	Simple	4-8	<10	**Present**	0.5-1.5 cm	Short
Q. cerris	5.5-14(-20)	**Large**	**Simple to deeply lobed**	5-9	<10	**Present**	0.3-2cm	Short

Character	6	7	8	9	10		
Species	**Leaf base**	**Leaf shape**	**Leaf margin**	**Tooth type**	**Basal tooth side**	**Tooth type categories**	
Q. acutissima	Obtuse, acute	**Narrow ovate, lanceolate**	Dentate	**Bristle-like**	**Weakly developed, Straight**	plesiomorph 1	**0**
Q. chenii	Obtuse, acute	**Narrow ovate, lanceolate**	Dentate	**Bristle-like**	**Weakly developed, Straight**	plesiomorph 1	**0**
Q. variabilis	Obtuse, acute	**Narrow ovate, lanceolate**	Dentate	**Bristle-like**	**Weakly developed, Straight**	plesiomorph 1	**0**
Q. crenata	Obtuse, acute, subauriculate	Narrow ovate, elliptic	Coarsely dentate	Coarse triangular	Convex to sigmoid	plesiomorph 2	**1**
Q. suber	Obtuse, acute	Ovate, elliptic	Dentate	Cuspidate	**Weakly developed**	derived 1 sclerophyllous	**2**
Q. brantii	Cordate	Ovate, elliptic	Dentate	Cuspidate	Convex to sigmoid	plesiomorph 2	**1**
Q. ithaburensis	Obtuse to cordate	Ovate, elliptic	Dentate	Cuspidate to **bristle-like**	Convex to sigmoid	plesiomorph 2	**1**
Q. macrolepis [incl. *Q. vallonea*]	Cordate	Ovate to broad ovate	Coarsely dentate	Coarse triangular	Convex	derived 2	**3**
Q. afares	Obtuse, acute	Narrow ovate, elliptic	Dentate	Coarse triangular to cuspidate	Convex to sigmoid	plesiomorph 2	**1**
Q. libani	Cordate, obtuse	Narrow ovate, elliptic, oblong	Dentate	**Bristle-like**	**Weakly developed, Straight**	plesiomorph 1	**0**
Q. trojana	Cordate	Narrow ovate, elliptic, oblong	Dentate	Cuspidate	Convex to sigmoid	plesiomorph 2	**1**
Q. euboica	Cordate	Narrow ovate, elliptic	Dentate	Cuspidate	Convex to sigmoid	plesiomorph 2	**1**
Q. castaneifolia	Obtuse, acute	Narrow ovate, elliptic	Coarsely dentate to dentate	Coarse triangular to cuspidate	Convex to sigmoid	plesiomorph 2	**1**
Q. look	Obtuse, acute, (sub)auriculate	Narrow ovate, elliptic	Coarsely dentate	Coarse triangular	Convex to sigmoid	derived 2	**3**
Q. cerris	Obtuse, acute, (sub)auriculate	Narrow to broad elliptic, obovate	Coarsely dentate	Coarse triangular	Convex	derived 3	**4**

Ad char. 10: Weakly developed = tooth area close to 0, tooth reduced to cusp or bristle-like extension (see **Fig. S1-7**).

Abbreviations used in legend of Fig. S1-7 (preceeding page): acu – *Quercus acutissima* , afa – *Q. afares* , bra – *Q. brantii* , cas – *Q. castaneifolia* , cer – *Q. cerris* , che – Q. chenii, cre – *Q. crenata* , eub – *Q. euboica* , ith – *Q. ithaburensis* , lib – Q. libani, loo – *Q. look* , mac – *Q. macrolepis* , sub – Q. suber, tro – *Q. trojana* , var – *Q. variabilis* .

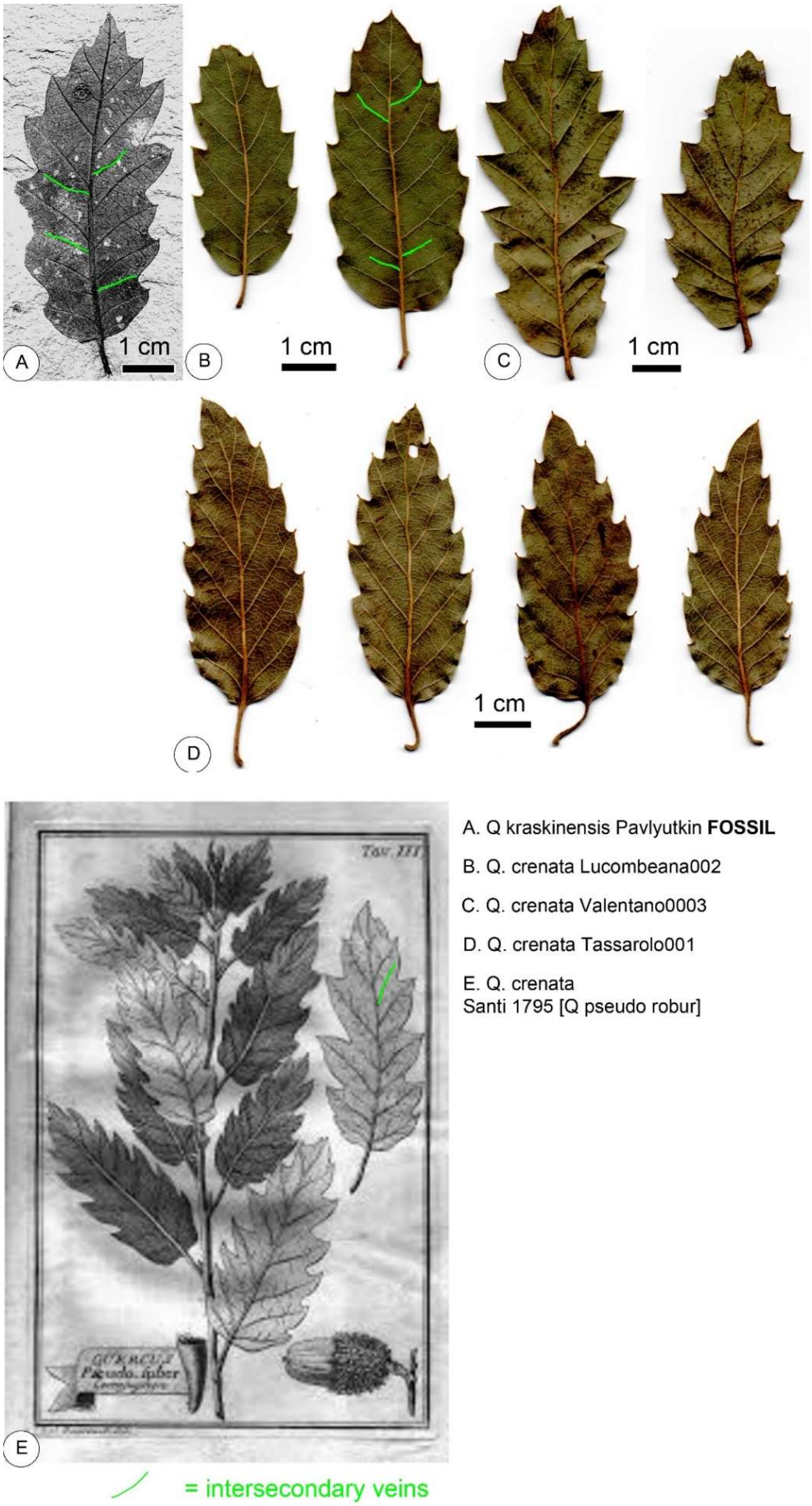

Plate S1-1. Leaf morphologies shared by modern-day *Q. crenata* and fossil-species *Q. kraskinensis* (Oligocene to Miocene, Central Asia to Europe); representing a likely symplesiomorphic leaf type of section *Cerris.*

References

Ashlock PD. 1971. Monophyly and associated terms. *Systematic Zoology* **20**:63–69.

Avishai M. 2016. Vallonea or Aegilops Oaks, a short review. *International Oak Society*, https://www.internationaloaksociety.org/content/vallonea-or-aegilops-oaks-short-review. Published online June 4, 2016.

Bouchal JM, Güner TH, Denk T. 2018. Middle Miocene climate of southwestern Anatolia from multiple botanical proxies. *Climates of the Past* **14**:1427–1440.

Bouckaert R, Heled J, Kühnert D, Vaughan T, Wu C-H, Xie D, Suchard MA, Rambaut A, Drummond AJ. 2014. BEAST 2: A Software Platform for Bayesian Evolutionary Analysis. *PLOS Computational Biology* **10**: e1003537.

Browicz K, Zieliński J. 1982. *Chorology of trees and shrubs in South-West Asia and adjacent regions*. Vol. 1. Warsaw: Polish Scientific Publishers.

Camus A. 1936–1938. Les Chênes. Monographie du genre *Quercus*. Tome I. Genre *Quercus*, sous-genre *Cyclobalanopsis*, sous-genre *Euquercus* (sections *Cerris* et *Mesobalanus*). Texte. Paris: Paul Lechevalier.

de Carvalho A. 1958. Identificação de um possível fóssil de sobreiro (*Quercus suber* L.) proveniente de solos do Mioceno lacustre do Alenjento. *Boletim da Sociedade Broteriana* **32**: 75–78.

Caudullo G, Welk E, San-Miguel-Ayanz J. 2017. Chorological maps for the main European woody species. *Data in Brief* **12**: 662–666.

Cohen KM, Finney SC, Gibbard PL, Fan J-X. 2013 (updated). The ICS International Chronostratigraphic Chart. *Episodes* **36**: 199–204. http://www.stratigraphy.org/index.php/ics-chart-timescale

Denk T, Grimm GW. 2010. The oaks of western Eurasia: Traditional classifications and evidence from two nuclear markers. *Taxon* **59**: 351–366.

Denk T, Grimm GW, Grímsson F, Zetter R. 2013. Evidence from "Köppen signatures" of fossil plant assemblages for effective heat transport of Gulf Stream to subarctic North Atlantic during Miocene cooling. *Biogeosciences* **10**: 7927–7942.

Durand EY, Patterson N, Reich D, Slatkin M. 2011. Testing for ancient admixture between closely related populations. *Molecular Biology and Evolution* **28**: 2239–2252.

Denk T, Grimm GW, Manos PS, Deng M, Hipp AL. 2017. An updated infrageneric classification of the oaks: review of previous taxonomic schemes and synthesis of evolutionary patterns. In: Gil-Pelegrín E, Peguero-Pina JJ, Sancho-Knapik D, eds. *Oaks Physiological Ecology. Exploring the Functional Diversity of Genus* Quercus *L.* Cham, Switzerland: Springer, 13–38.

Eaton DAR, Overcast I. 2020. ipyrad: Interactive assembly and analysis of RADseq datasets. *Bioinformatics* **36**: 2592–2594.

Escudero A, del Arco JM. 1987. Ecological significance of the phenology of leaf abscission. *Oikos* **49**: 11–14.

Fang J, Wang Z, Tang Z. 2009. *Atlas of Woody Plants in China.* Volumes 1 to 3 and index. Beijing: Higher Education Press.

Fick SE, Hijmans RJ. 2017. WorldClim 2: new 1km spatial resolution climate surfaces for global land areas. *International Journal of Climatology* **37**: 4302–4315.

Grimm GW. 2022. Just a single, easily overlooked tip. Res.I.P. blogpost, posted 1/7/2022. https://researchinpeace.blogspot.com/2022/07/just-single-easily-overlooked-tip.html

Grímsson F, Grimm GW, Potts AJ, Zetter R, Renner SS. 2018. A Winteraceae pollen tetrad from the early Paleocene of western Greenland, and the fossil record of Winteraceae in Laurasia and Gondwana. *Journal of Biogeography* **45**: 567–581.

le Hardÿ de Beaulieu A, Lamant T. 2010. *Guide Ilustré des Chênes*. Edilens: Geeer, Belgium.

Hipp AL, Manos PS, Hahn M, Avishai M, …[17 more authors]… Valencia-Avalos S. 2020. The genomic landscape of the global oak phylogeny. *New Phytologist* **226**: 1198–1212.

Huang C, Zhang Y, Bartholomew B. 1999. *Fagaceae*. In: Wu Z-Y, Raven PH, eds. *Flora of China*, *Vol. 4: Cycadaceae through Fagaceae*. Beijing and St. Louis: Science Press and Missouri Botanical Garden Press, 314–400.

Kottek M, Grieser J, Beck C, Rudolf B, Rubel F. 2006. World map of the Köppen-Geiger climate classification updated. *Meteorologische Zeitschrift* **15**: 259–263.

Maddison WP, Maddison DR. 2011. *Mesquite: a modular system for evolutionary analysis.* Version 2.75. Available at http://www.mesquiteproject.org.

Magri D, Fineschi S, Bellarosa R, *et al.* 2007. The distribution of *Quercus suber* chloroplast haplotypes matches the palaogeographical history of the western Mediterranean. *Molecular Ecology* **16**: 5259–5266.

Maire R. 1961. Flore de l'Afrique du Nord (Maroc, Algérie, Tunisie, Tripolitaine, Cyrénaïque et Sahara). Vol 7. Paris: Editions Paul Lechevalier, 117–120.

Manos PS, Zhou ZK, Cannon CH. 2001. Systematics of Fagaceae: phylogenetic tests of reproductive trait evolution. *International Journal of Plant Sciences* **162**: 1361–1379.

McVay JD, Hipp AL, Manos PS. 2017b. A genetic legacy of introgression confounds phylogeny and biogeography in oaks. *Proceedings of the Royal Society B* **284**: 20170300.

Mhamdi S, Brendel O, Montpied P, Ghouil-Amimi H, Hasnaoui I, Dreyer E. **2013**. Leaf morphology displays no detectable spatial organisation in the relict *Quercus afares* Pomel compared to the co-occurring parental species *Q. canariensis* Willd. and *Q. suber* L. *Annals of Forest Science* **70**: 675–684.

Menickij JL. 1984. *Duby Asii*. Leningrad: Nauka. English translation published as: **Menitsky YL. 2005.** *Oaks of Asia*. Boca Raton: Science Publishers, CRC Press.

Mir C, Toumi L, Jarne P, Sarda V, Di Giusto F, Lumaret R. 2006. Endemic North African *Quercus afares* Pomel originates from hybridisation between two genetically very distant oak species (*Q. suber* L. and *Q. canariensis* Willd.): evidence from nuclear and cytoplasmic markers. *Heredity* **96**: 175–184.

Ørsted AS. 1871. Bidrag til kundskab om Egefamilien. Det Kongelige Danske Videnskabernes Selskabs Skrifter, Serie 5, Naturvidenskabelig og Mathematisk Afdelning **9**: 331–538.

Olson DM, Dinerstein E, Wikramanayake ED, …[14 more authors]…, Kassem KR. 2001. Terrestrial ecoregions of the world: a new map of life on Earth. *BioScience* **51**: 933–938.

Peel MC, Finlayson BL, McMahon TA. 2007. Updated world map of the Köppen-Geiger climate classification. *Hydrology and Earth System Sciences* **11**: 1633–1644.

Pignatti S. 2017-2019. Flora d'Italia. Milano: Edagricole, New Business Media.

Piredda R, Grimm GW, Schulze E-D, Denk T, Simeone MC. 2020. High-throughput sequencing of 5S-IGS in oaks: Exploring intragenomic variation and algorithms to recognize target species in pure and mixed samples. *Molecular Ecology Resources* **21**:495–510.

de Rigo D, Enescu CM, Houston Durrant T, Caudullo G. 2016. *Quercus cerris* in Europe: distribution, habitat, usage and threats. In: San-Miguel-Ayanz J, de Rigo D, Caudullo G, Houston Durrant T, Mauri A, eds. *European Atlas of Forest Tree Species*. Publication Office EU, Luxembourg, 148–149.

Rubel F, Brugger K, Haslinger K, Auer I. 2017. The climate of the European Alps: Shift of very high resolution Köppen-Geiger climate zones 1800–2100. *Meteorologische Zeitschrift* **26**: 115–125.

San-Miguel-Ayanz J, de Rigo D, Caudullo G., Houston Durrant T, Mauri A, eds. 2016. *European Atlas of Forest Tree Species*. Luxembourg: Publication Office EU.

Schirone B, Spada F, Simeone MC, Vessella F. 2015. *Quercus suber* distribution revisited. In: Box EO, Fujiwara K, eds. *Geobotany studies - Warm-temperate deciduous forests around the Northern Hemisphere*. Chur, Switzerland: Springer, 181–212.

Schroeder G-F. 1998. *Lehrbuch der Pflanzengeographie.* Wiesbaden: Quelle & Meyer.

Schwarz O. 1936. Entwurf zu einem natürlichen System der Cupuliferen und der Gattung *Quercus* L. *Notizblatt des Königlichen Botanischen Gartens und Museums zu Berlin-Dahlem* **13 (116)**: 1–22.

Schwarz O. 1936–1939. *Monographie der Eichen Europas und des Mittelmeergebietes*. Feddes Repertorium regni vegetabilis. Berlin-Dahlem: Sonderbeiheft D.

Scotese CR, Boucot AJ, Chen Xu. 2014. *Atlas of Phanerozoic climatic zones (Mollweide Projection).* Volumes 1–6. Evanston, IL: PALEOMAP Project PaleoAtlas for ArcGIS, PALEOMAP Project.

Simeone MC, Grimm GW, Papini A, Vessella F, Cardoni S, Tordoni E, Piredda R, Franc A, Denk T. 2016. Plastome data reveal multiple geographic origins of *Quercus* Group *Ilex*. *PeerJ* **4**: e1897.

Simeone MC, Cardoni S, Piredda R, Imperatori F, Avishai M, Grimm GW, Denk T. 2018. Comparative systematics and phylogeography of *Quercus* section *Cerris* in western Eurasia: inferences from plastid and nuclear DNA variation. *PeerJ* **6**: e5793.

Sousa VB, Leal S, Quilhó T, Pereira H. 2009. Characterization of cork oak (*Quercus suber*) wood anatomy. *IAWA Journal* **30**: 149–161.

Trewartha GT, Horn LH. 1980. *Introduction to Climate*, 5th ed. New York, USA: McGraw-Hill.

Welter S, Bracho-Nuñez A, Mir C, Zimmer I, Kesselmeier J, Lumaret R, Schnitzler JP, Staudt M. 2012. The diversification of terpene emissions in Mediterranean oaks: lessons from a study of *Quercus suber*, *Quercus canariensis* and its hybrid *Quercus afares*. *Tree Physiology* **32**:1082–1091.

Yang Y-Y, Qu X-J, Zhang R, Stull GW, Yi T-S. 2021. Plastid phylogenomic analyses of Fagales reveal signatures of conflict and ancient chloroplast capture. *Molecular Phylogenetics and Evolution* **163**:107232.

Zhou BF, Yuan S, Crowl AA, Liang YY, Shi Y, Chen XY, An Q-Q, Kang M, Manos PS, Wang B. 2022. Phylogenomic analyses highlight innovation and introgression in the continental radiations of Fagaceae across the Northern Hemisphere. *Nature Communications* **13**: 1–14.
