# Supplementary material for: Niche evolution in a northern temperate tree lineage: biogeographical legacies in cork oaks (Quercus section Cerris)
Source: Ann Bot. 2023 Feb 20;131(5):769–87. doi: 10.1093/aob/mcad032 (PMC10184457; doi:10.1093/aob/mcad032)
Supplement: mcad032_suppl_Supplementary_Data [file mcad032_suppl_supplementary_data.zip › Supplementary Data 3/Supplementary Data 3 BiomeClimateNichesCerris final.pdf]

# Supplementary Data 3:

**Description of Köppen-Geiger climate symbols** and defining criteria (Kottek et al. 2006; Peel et al., 2007; Rubel et al., 2017; Cui et al., 2021)

| 1st | 2nd | 3rd | Description and criteria |
|---|---|---|---|
| *A* | | | **Equatorial** / tropical—MTCM ≥ 18 °C) |
| | *f* | | Rainforest, fully humid—$P_{dry}$ ≥ 60 mm) |
| | *m* | | Monsoonal—not *Af* AND $P_{dry}$ ≥ 100 − MAP/25 |
| | *s* | | Savannah with dry summer—$P_{sdry}$ < 60 mm |
| | *w* | | Savannah with dry winter—$P_{wdry}$ < 60 mm |
| *B* | | | **Arid**—MAP < 10 × $P_{threshold}$ |
| | *W* | | Desert—MAP < 5 × $P_{threshold}$ |
| | *S* | | Steppe—MAP ≥ 5 × $P_{threshold}$ |
| | | *h* | Hot arid—MAT ≥ 18 °C |
| | | *k* | Cold arid—MAT < 18 °C |
| *C* | | | **Warm temperate** (subtropical to temperate)—$T_{hot}$ > 10 °C AND 0 °C < MTCM < 18 °C |
| *D* | | | **Snow** (cold-temperate to boreal)—$T_{hot}$ > 10 °C AND MTCM ≤ 0 °C |
| | *s* | | Summer dry—$P_{sdry}$ < 40 mm AND $P_{sdry}$ < $P_{wwet}$/3 |
| | *w* | | Winter dry—$P_{wdry}$ < $P_{swet}$/10 |
| | *f* | | Fully humid, without a dry season, i.e. not *s* or *w* |
| | | *a* | Hot summer—$T_{hot}$ ≥ 22 °C |
| | | *b* | Warm summer—not *a* AND $T_{mon10}$ ≥ 4 |
| | | *c* | Cool/ cold and short summer—not *a/b* AND 1 ≤ $T_{mon10}$ < 4 |
| | | *d* | Extremely continental: cold/very short or no summer and very cold winter—not *a/b* AND MTCM < −38 °C |
| *E* | | | **Polar**—$T_{hot}$ < 10 °C |
| | *T* | | Tundra—$T_{hot}$ > 0 °C |
| | *F* | | Ice, permanent frost (‘frost desert’)—$T_{hot}$ ≤ 0 °C |

Abbrev.: MAP = mean annual (cumulative) precipitation; MAT = mean annual temperature; MTCM = mean temperature of the coldest month; $P_{dry}$ = precipitation of the driest month; $P_{sdry}$ = precipitation of the driest month in summer; $P_{swet}$ = precipitation of the wettest month in summer; $P_{wdry}$ = precipitation of the driest month in winter; $P_{wwet}$ = precipitation of the wettest month in winter; $T_{hot}$ = mean temperature of the hottest month; $T_{mon10}$ = number of months with a monthly mean temperature above 10 °C.

$P_{threshold}$ (generic threshold): If 70% of MAP occurs in winter then $P_{threshold}$ = 2 × MAT; if 70% of MAP occurs in summer then $P_{threshold}$ = 2 x MAT + 28; otherwise $P_{threshold}$ = 2 × MAT + 14.

Summer (winter) is defined as the warmer (cooler) six months period of October to March and April to September.

**Köppen-Geiger climate map colour coding**

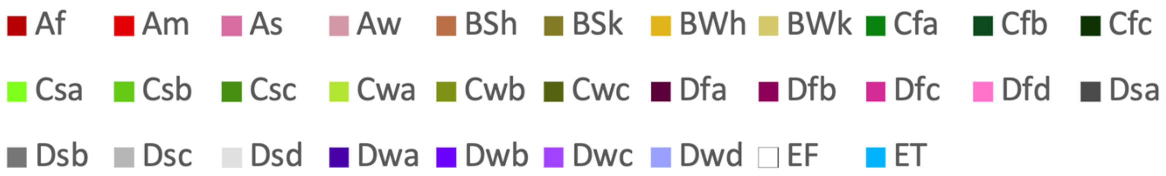


**Biome map colour coding** using the biome categories of Olson et al. (2001)

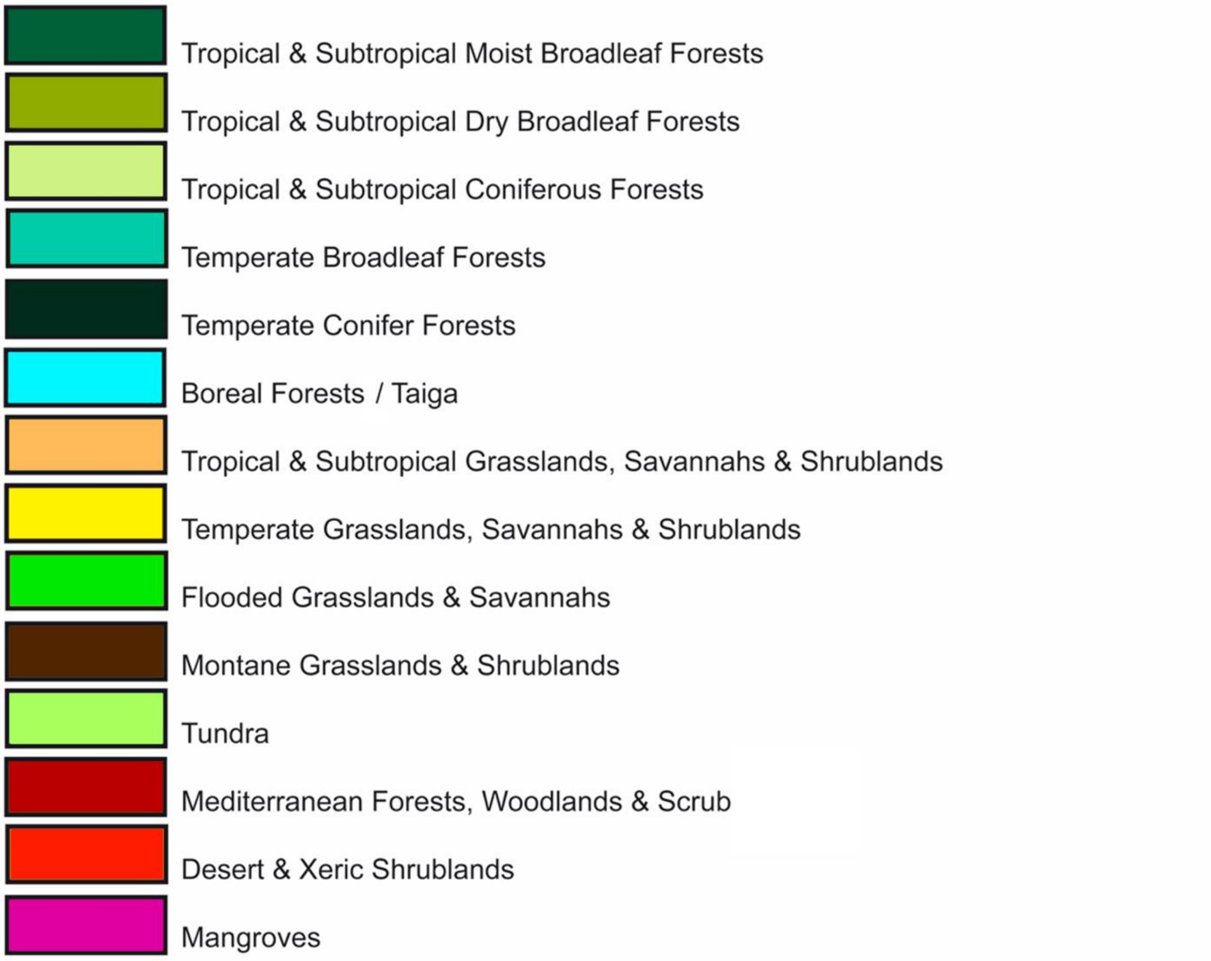

## Phylogenetic framework

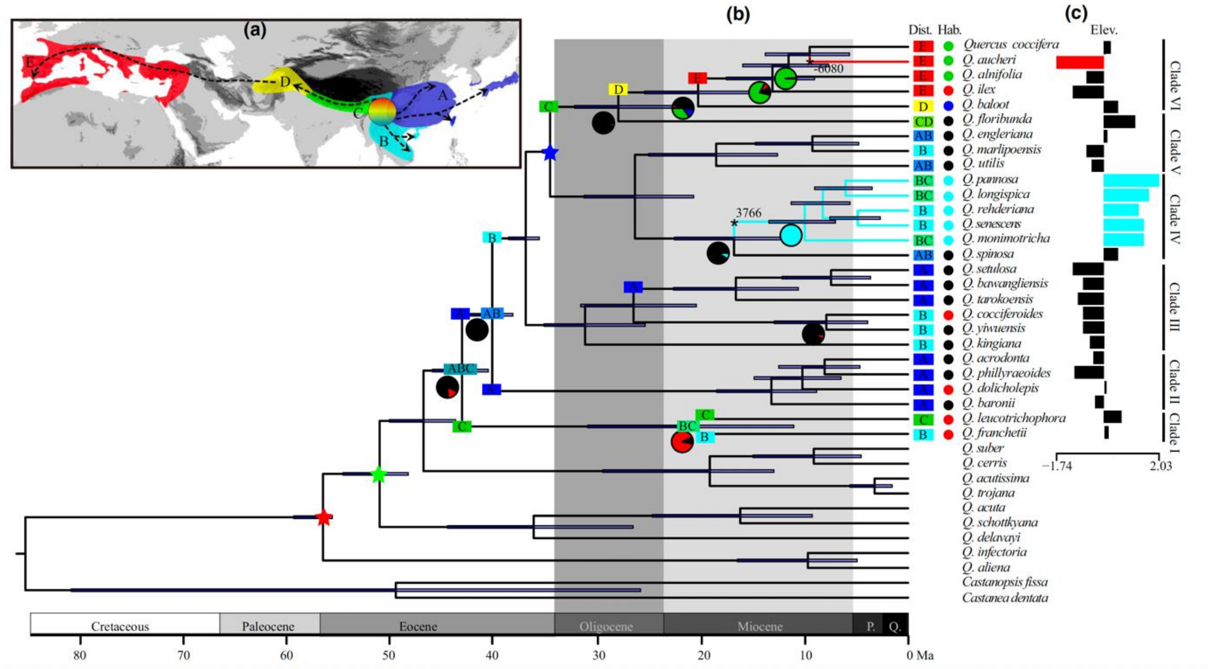


Historical biogeography and phylogenetic relationships within *Quercus* sect. *Ilex* after Jiang et al. (2019, fig. 2)

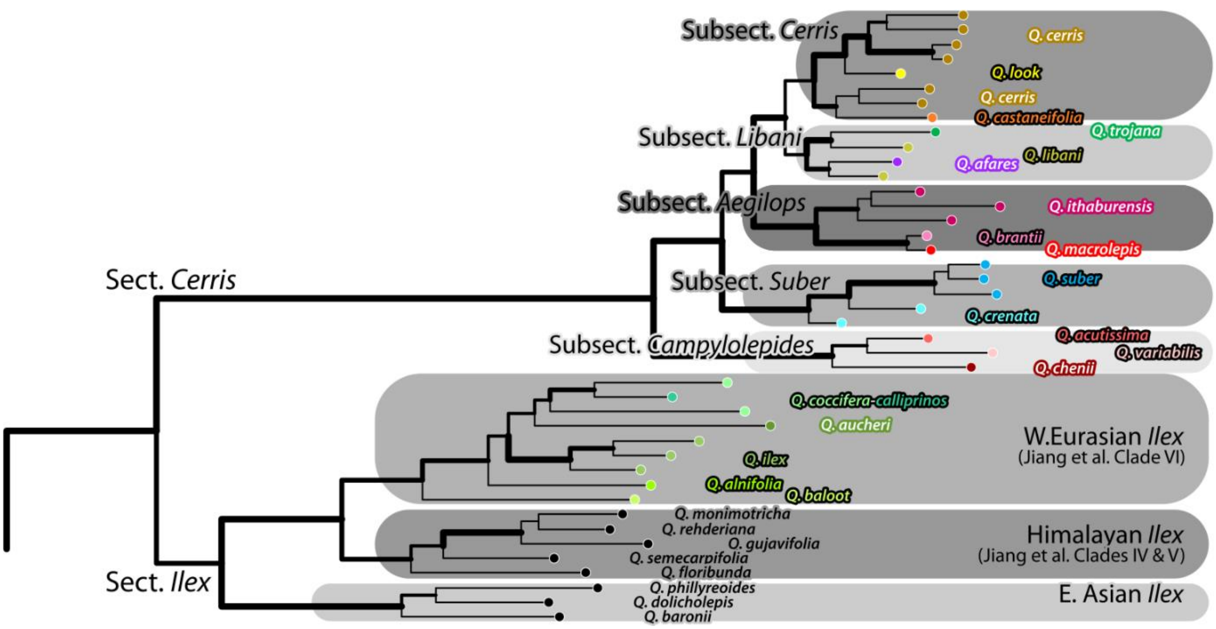


Tip-reduced phylogenetic tree used in this study.

# Overview: *Quercus* sect. *Cerris*

## KÖPPEN MAP—Western Eurasia: subsects *Aegilops, Cerris, Libani* and *Suber*

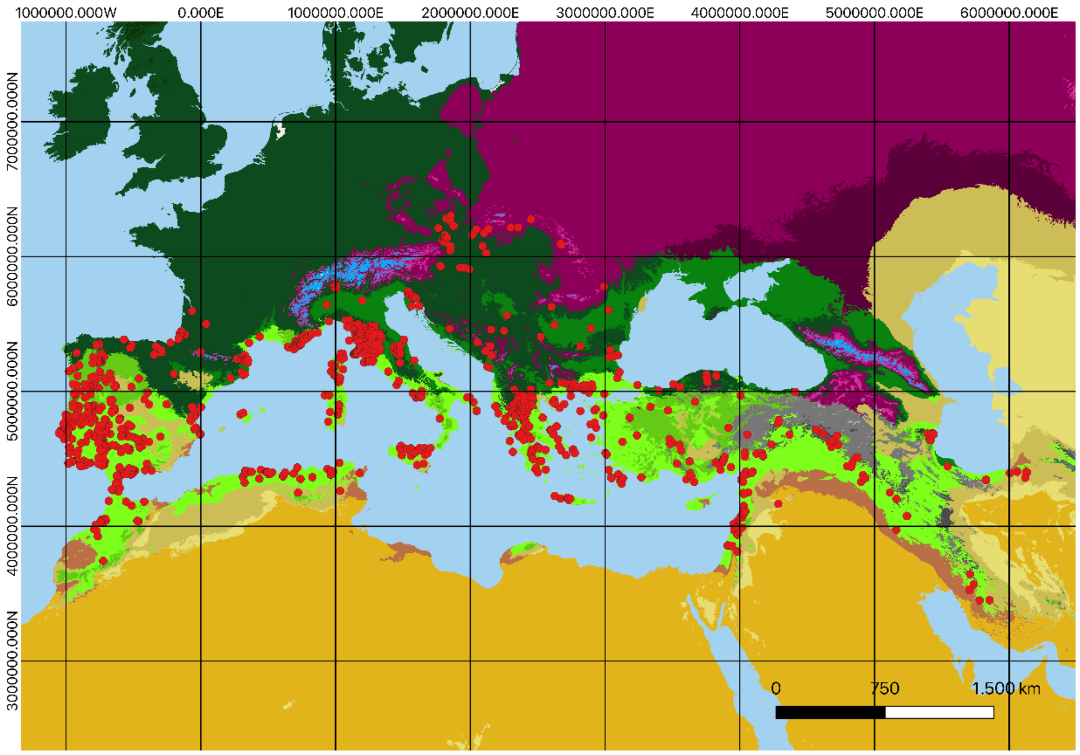


## KÖPPEN MAP—East Asia: subsect. *Campylolepides*

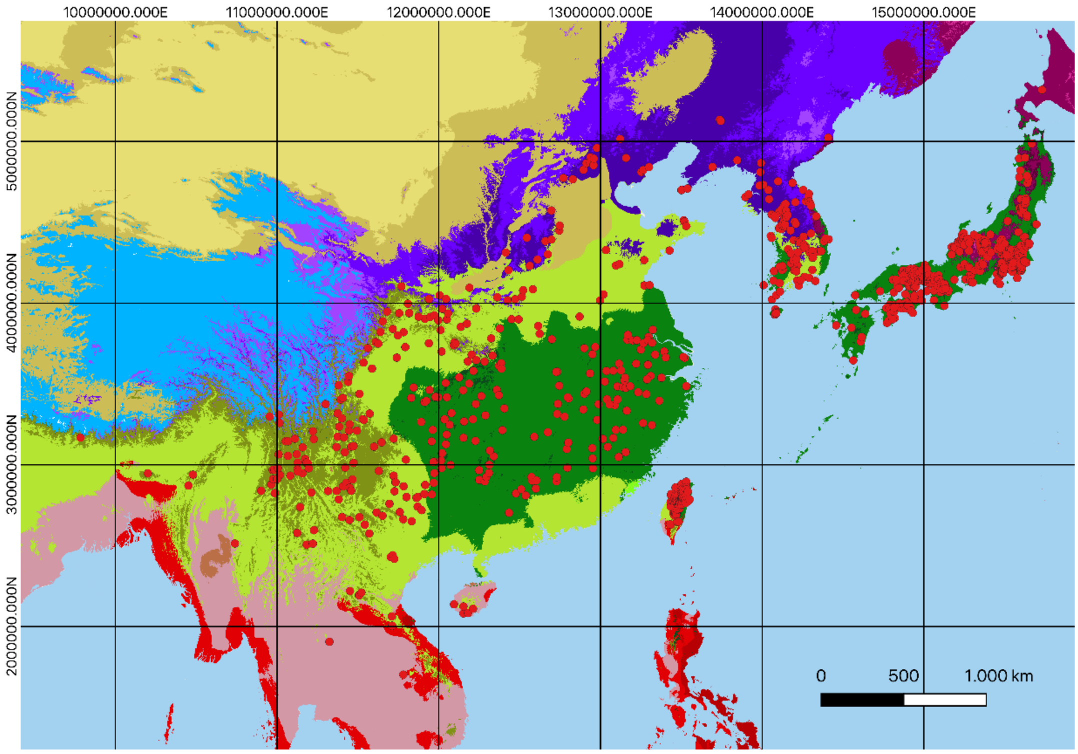

## BIOME MAP—Western Eurasia

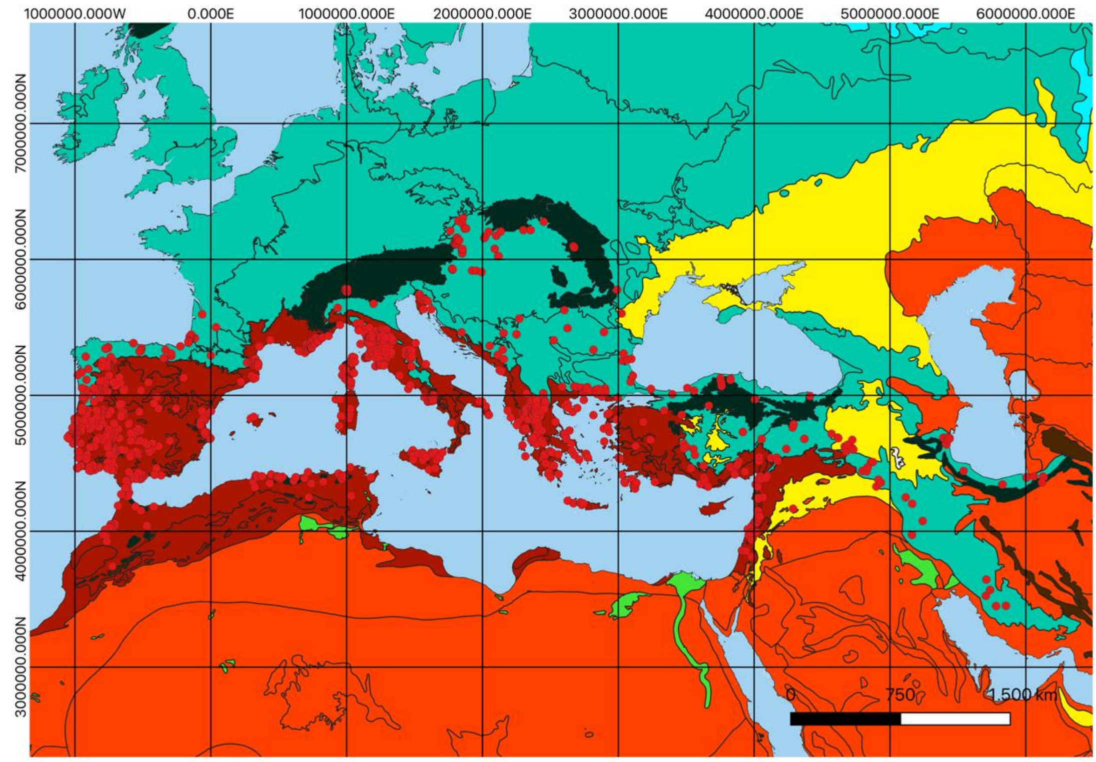


## BIOME MAP—East Asia

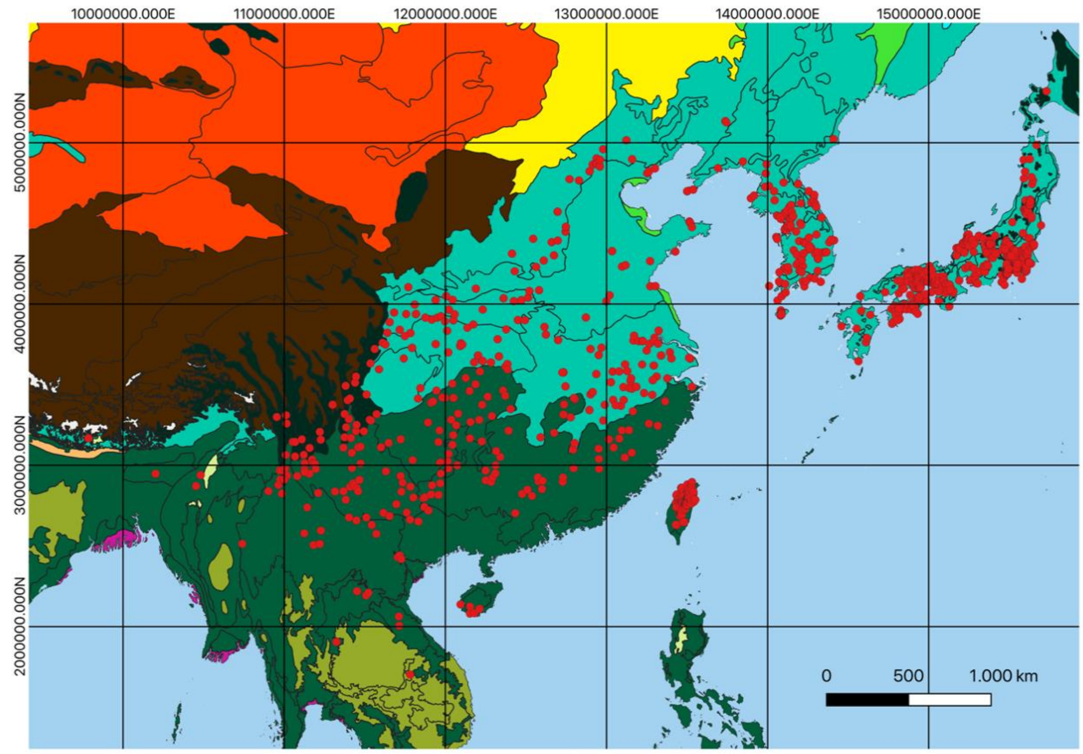

**Mean temperature of coldest month (MTCM, y-axis) vs precipitation of coldest quarter (PCQ, x-axis) for all members sect. *Cerris* (n = 2779).** MTCM ≤ −3 °C: ‘snow’ (*D*) climates, pronounced winter with permanent snow cover; MTCM = −3–7 °C, PCQ > 50 mm: warm temperate (*C*) climates with ± mild winters; frosts relatively frequent, no permanent snow cover; PCQ < 50 mm: continental arid (*B*: steppe, desert) or snow climates in Western Eurasia, in East Asia including summer-monsoon, winter-dry warm temperate (*Cw*) climates with or without (MTCM ≥ 4°C) winter. MTCM = 7–18 °C: warm temperate or arid climates without pronounced winter (very few or no night frosts) in the Mediterranean and south of the alpine mountain chains of Asia. MTCM ≥ 18 °C: tropical *A*-climates, no winter (no/little temperature seasonality).

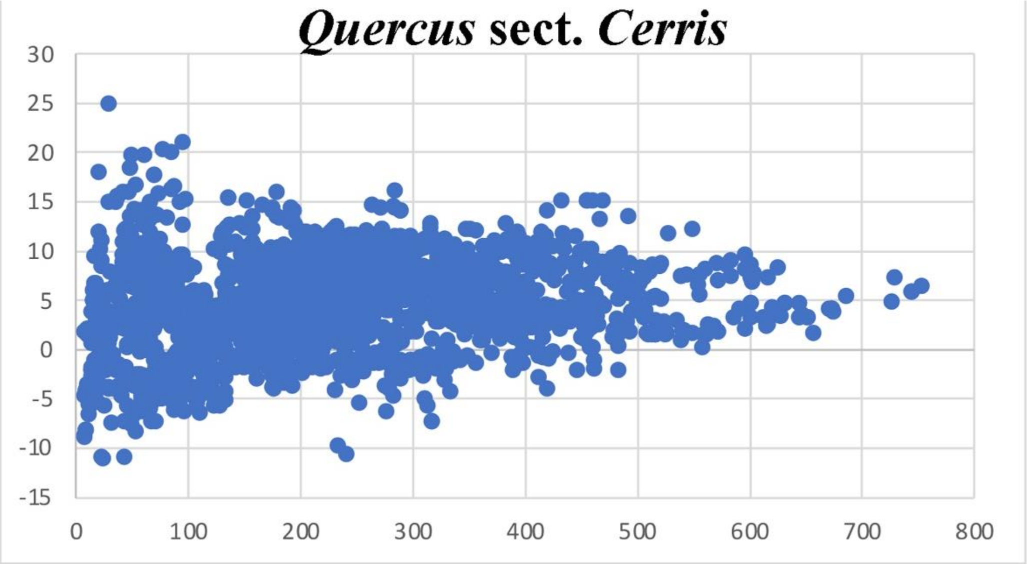


The total niche of sect. *Cerris* includes a wide range of climates with substantially different winter conditions ranging from tropical-subtropical monsoonal (very dry winters, very moist summers; *Aw* and *Cwa*/*Cwb*) to boreal, winter-dry (*Dwa*/*Dwb*) or snow-prone (*Dfa*/*Dfb*), or distinctly continental (*BSk*) climates in East Asia, and fully Mediterranean (dry summers, rainy winters; *Csa*) to oceanic, fully temperate and perhumid (*Cfa*, *Cfb*) as well as continental, summer-dry climates with snowy winters in Western Eurasia (*Dsa*, *Dsb*). Aside from these extremes, sect. *Cerris* thrives best in climates with mild winters (MTCM = 0–10 °C) and relatively high winter precipitation (PCQ = 100–500 mm).

## Quercus *sect.* Cerris *subsect.* Cerris

Cumulative niche of *Q. castaneifolia* and *Q. cerris* (no distribution data on *Q. look*).

**KÖPPEN MAP** (n = 681)

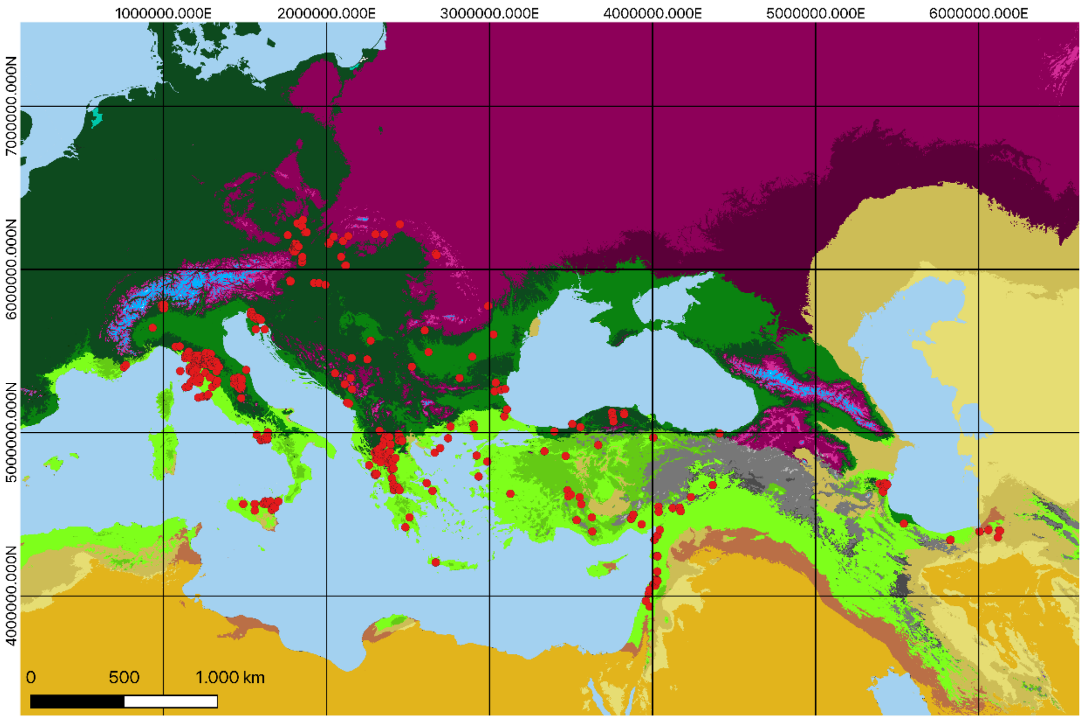


MTCM (x-axis) vs PCQ (y-axis) for members of subsect. *Cerris* (n = 451). This *Cerris* core clade lineage prefers the section's core niche with mild and relatively moist winters.

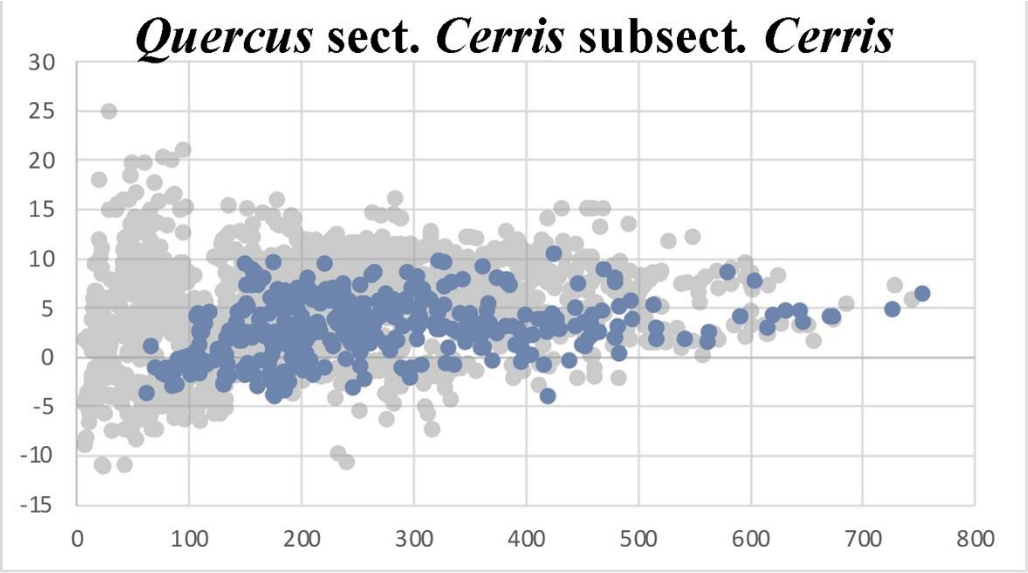

**KÖPPEN PROFILES – *Quercus castaneifolia* C.A.Mey., 1831**
GBIF localities of *Q. castaneifolia* (preserved specimen)

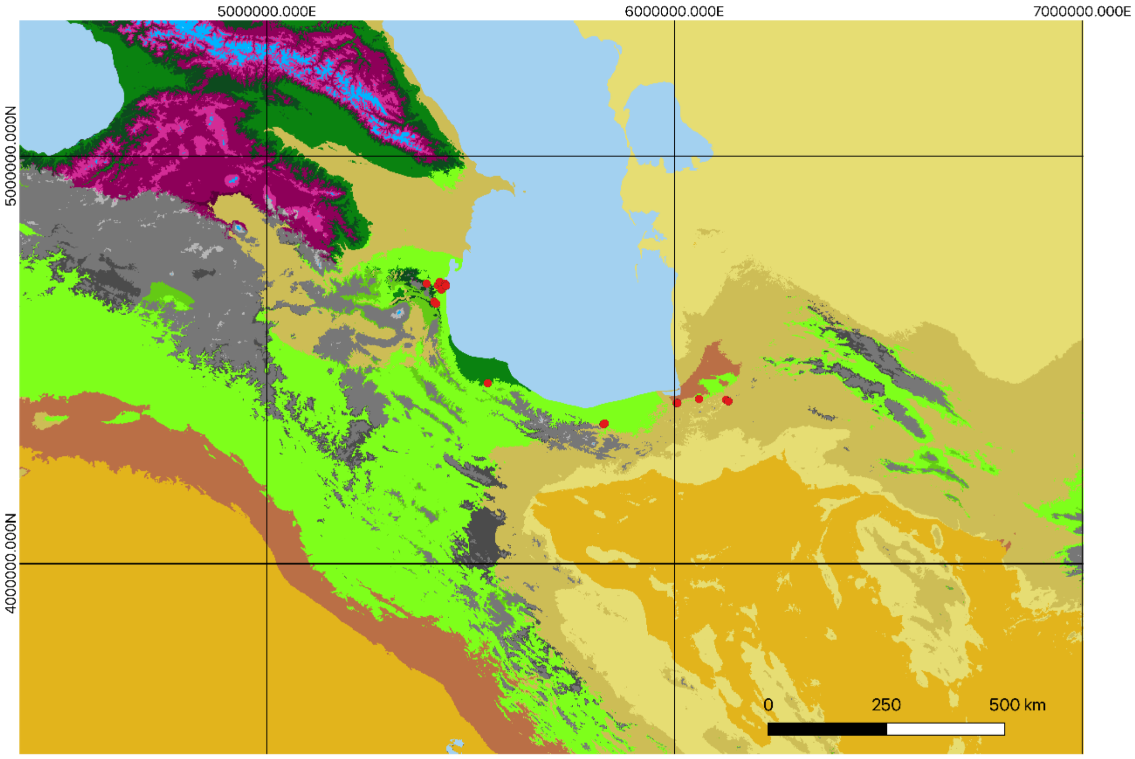


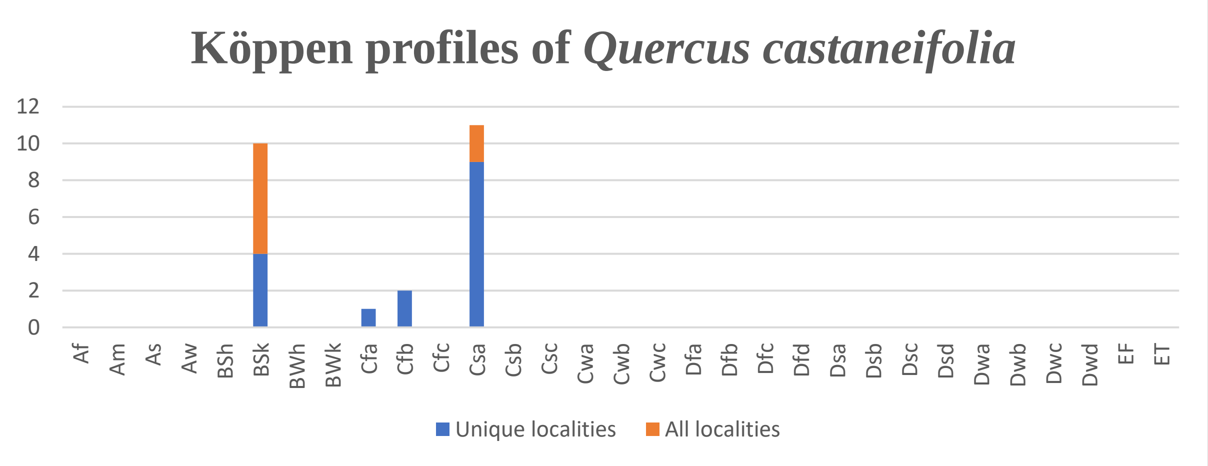


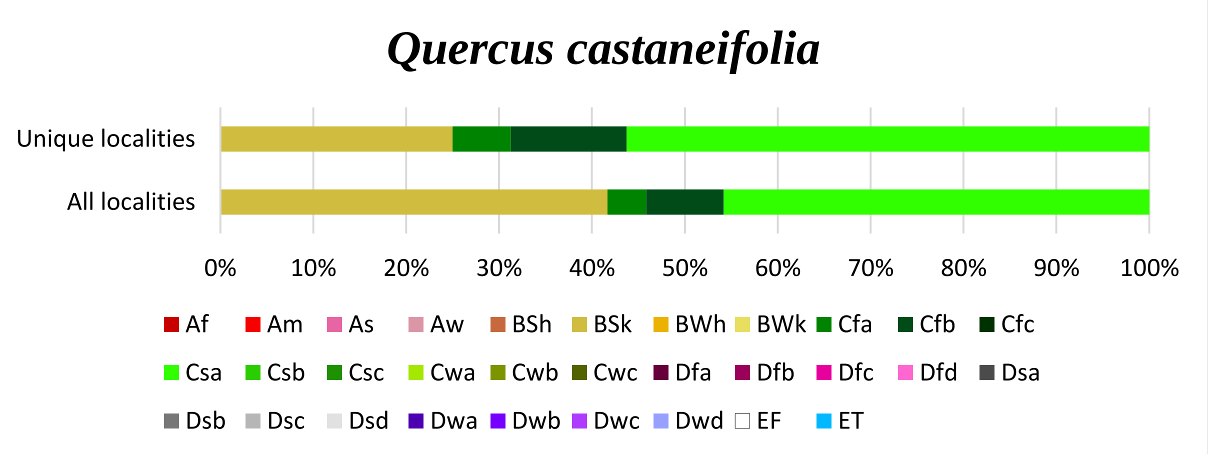


All localities (n = 24); unique localities (n = 16)

**BIOMES – *Quercus castaneifolia***
GBIF localities of *Q. castaneifolia* (preserved specimen)

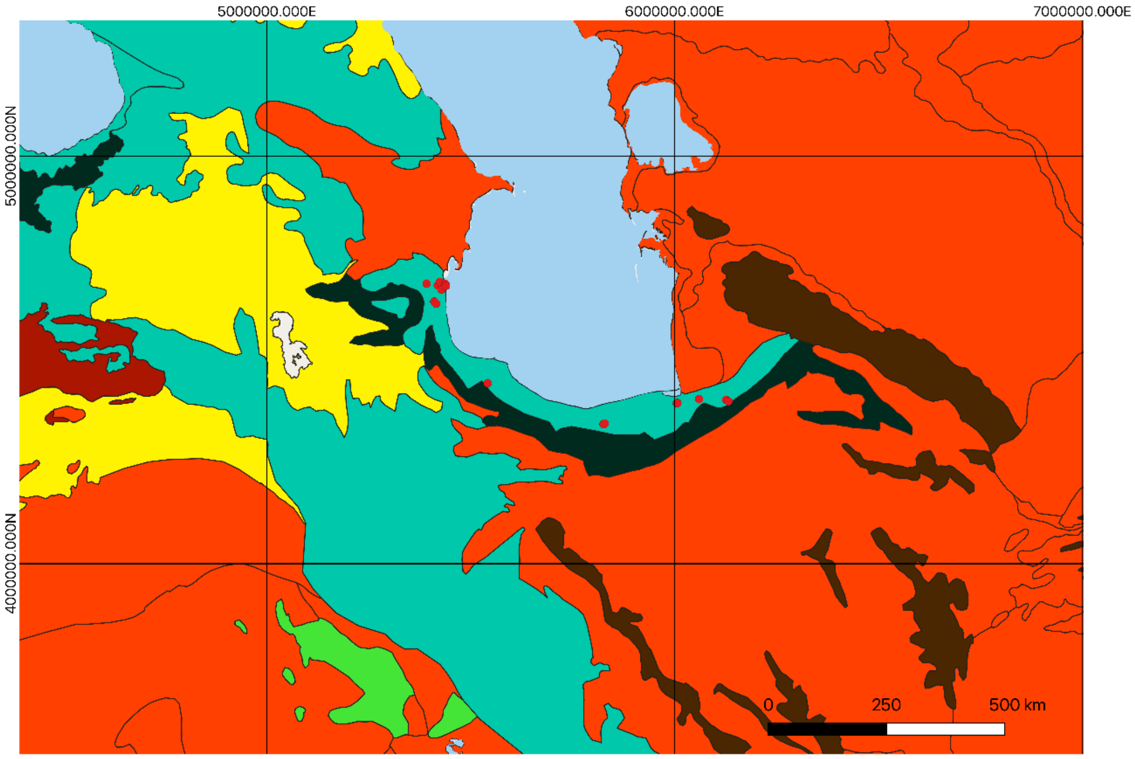


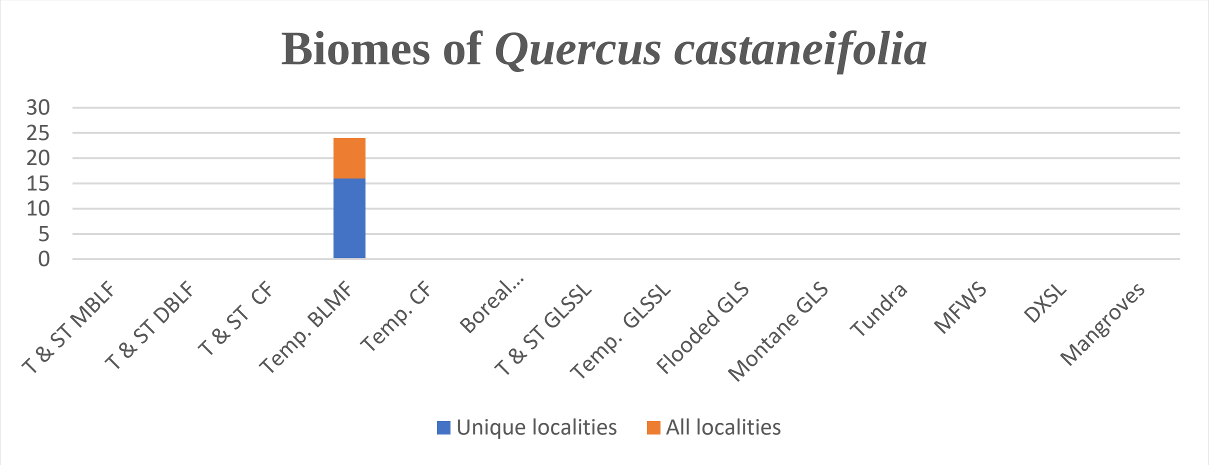


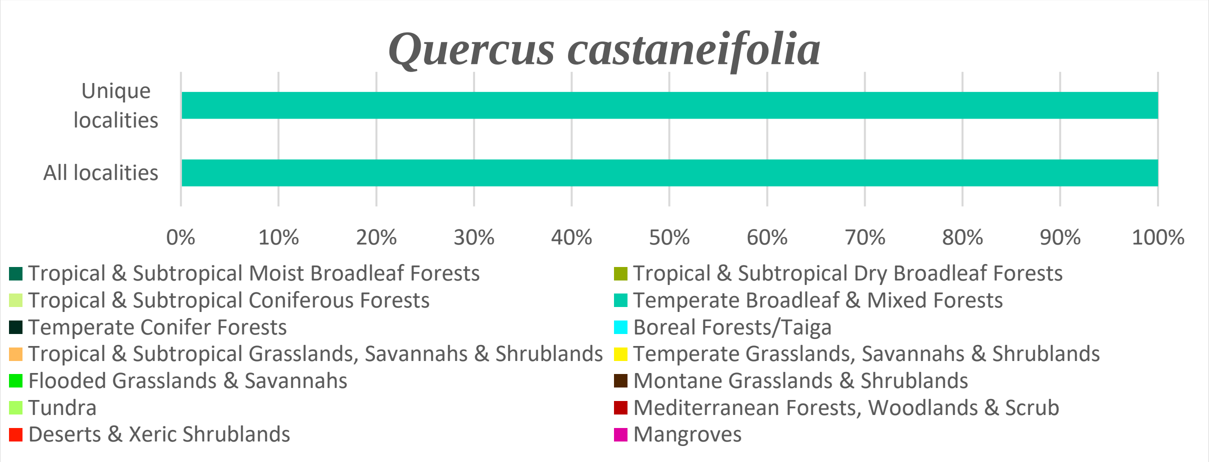


All localities (n = 24); unique localities (n = 16)

**MTCM (x-axis) vs PCQ (y-axis) for *Quercus castaneifolia* (n = 16).** A strongly restricted endemic thriving in the section's core niche, affected by a monsoon-like precipitation pattern in autumn (→ MMP boxplots) and facing little risk of frost (→ $MT_{min}$ boxplots).

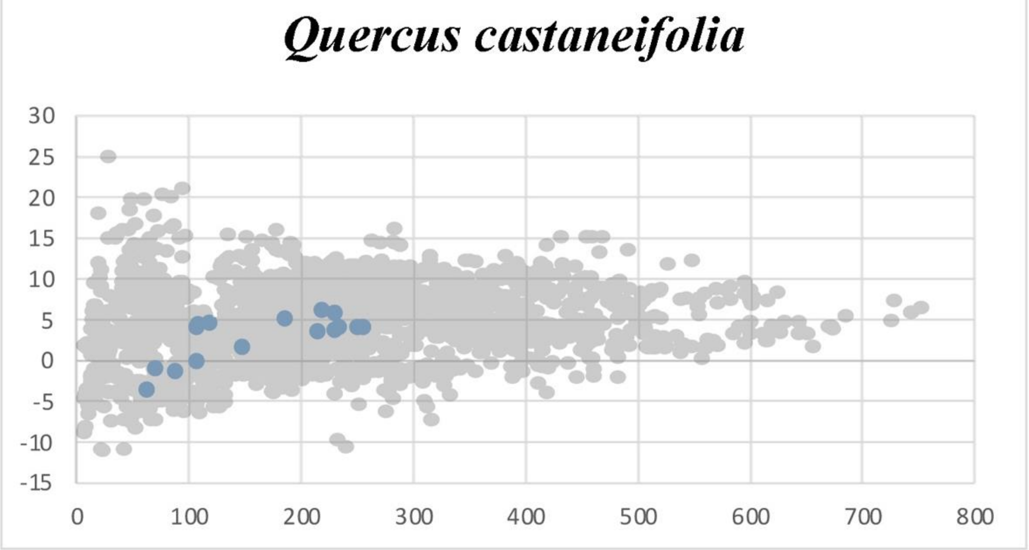


**Monthly mean precipitation (MMP) boxplots for *Quercus castaneifolia* (n = 16)**

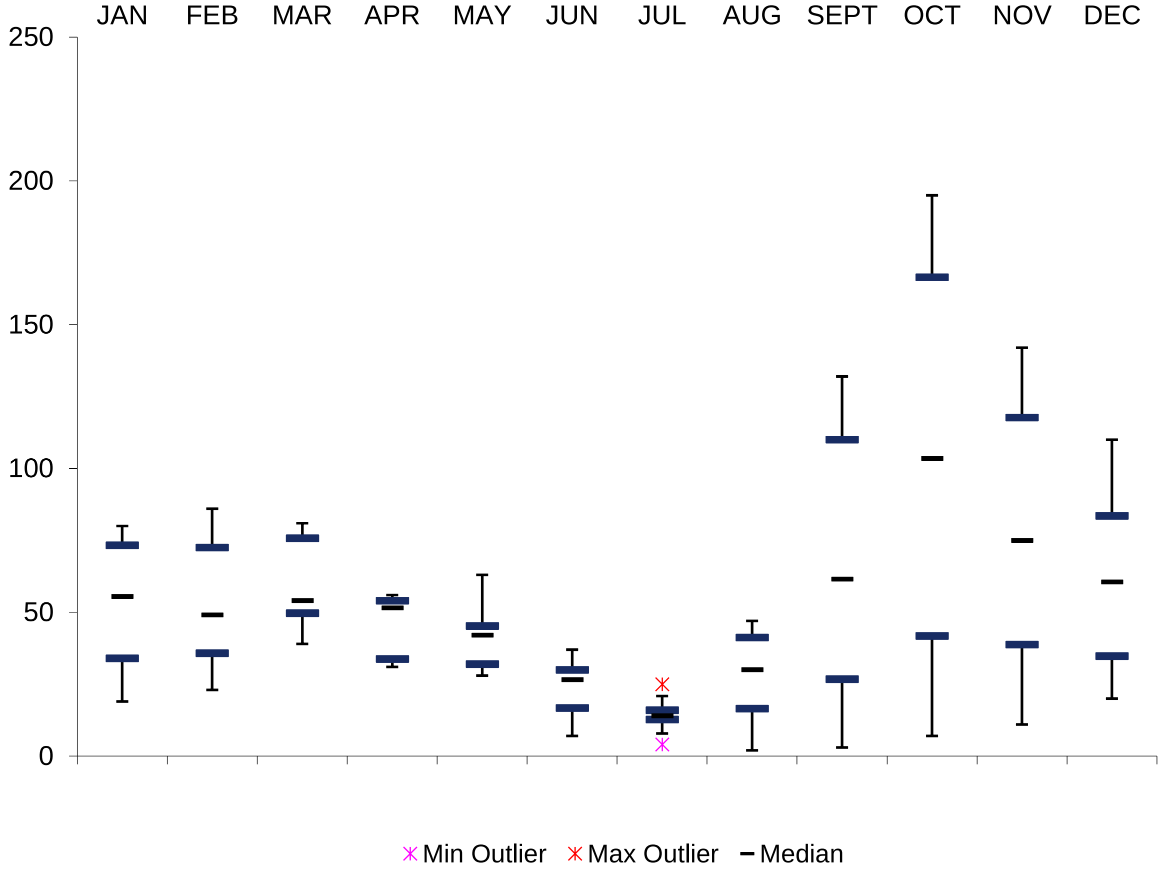

Monthly mean temperature (MMT) boxplots for *Quercus castaneifolia* (n = 16)

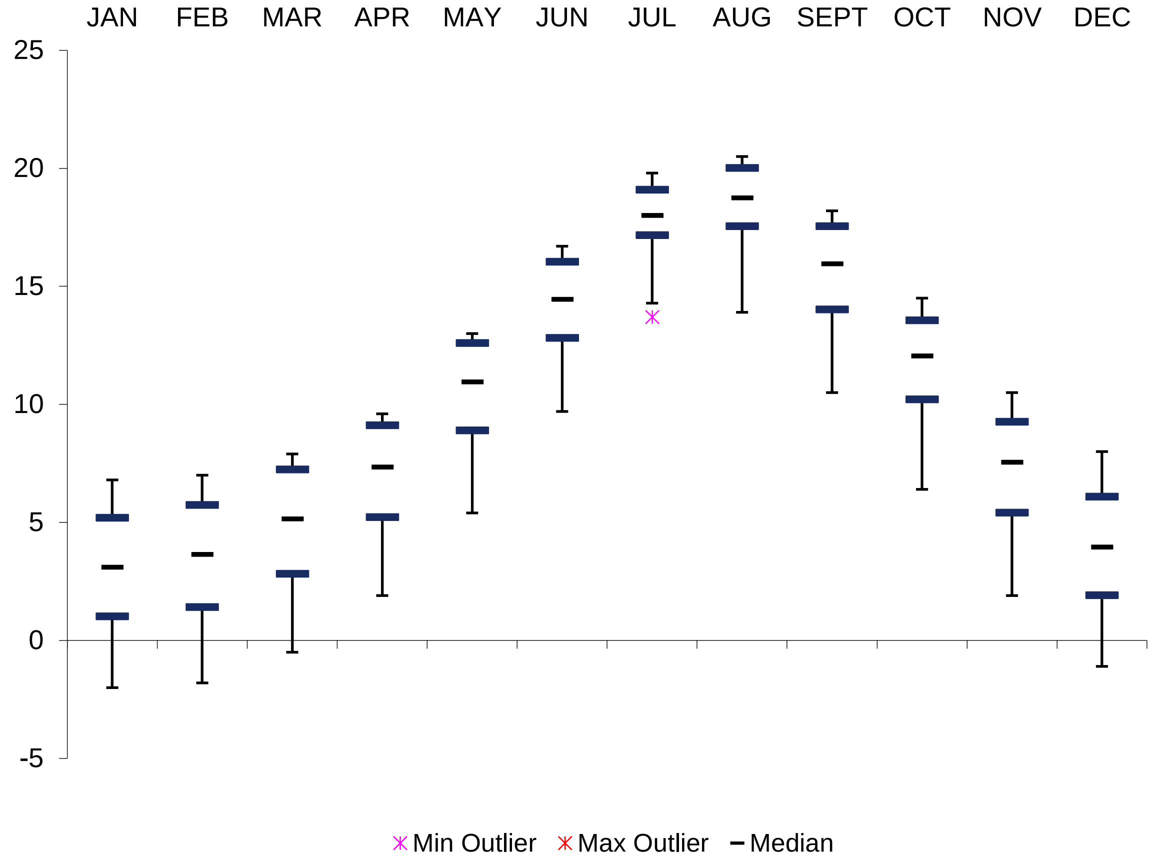


(Absolute) Monthly temperature minima ($MT_{min}$) boxplots for *Quercus castaneifolia* (n = 16)

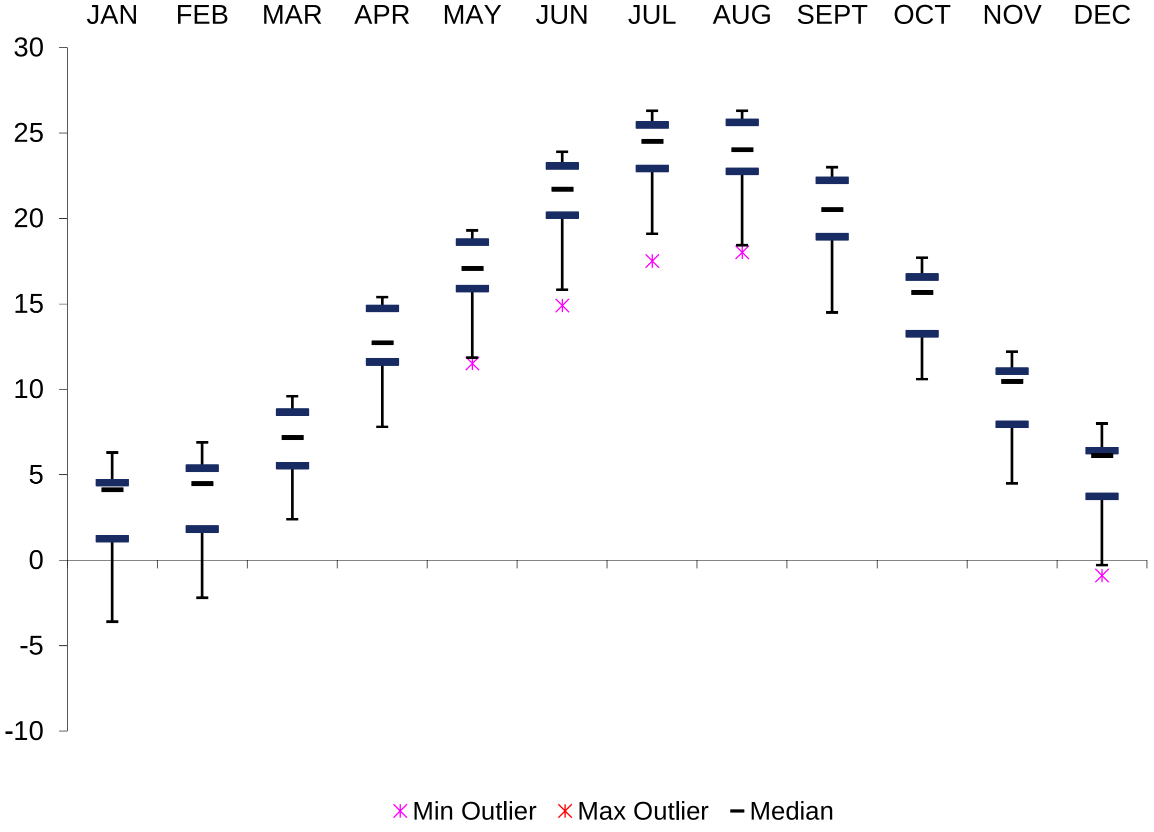

**KÖPPEN PROFILES – *Quercus cerris* L., 1753**

GBIF localities of *Q. cerris* (preserved specimen)

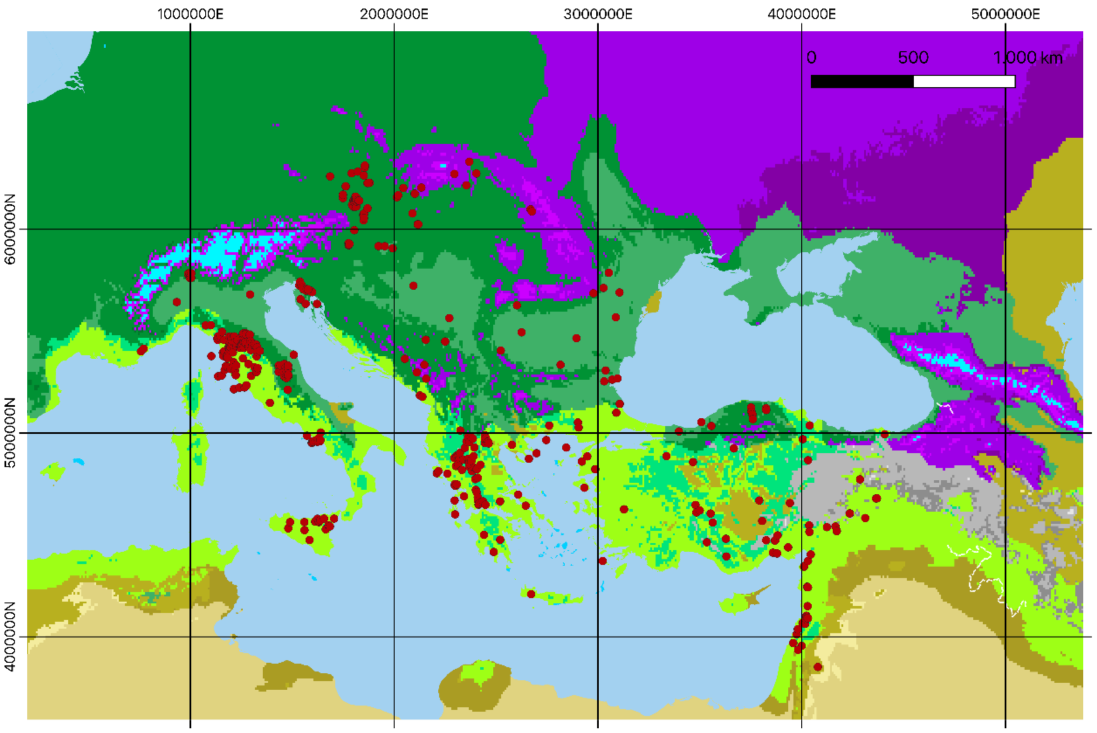


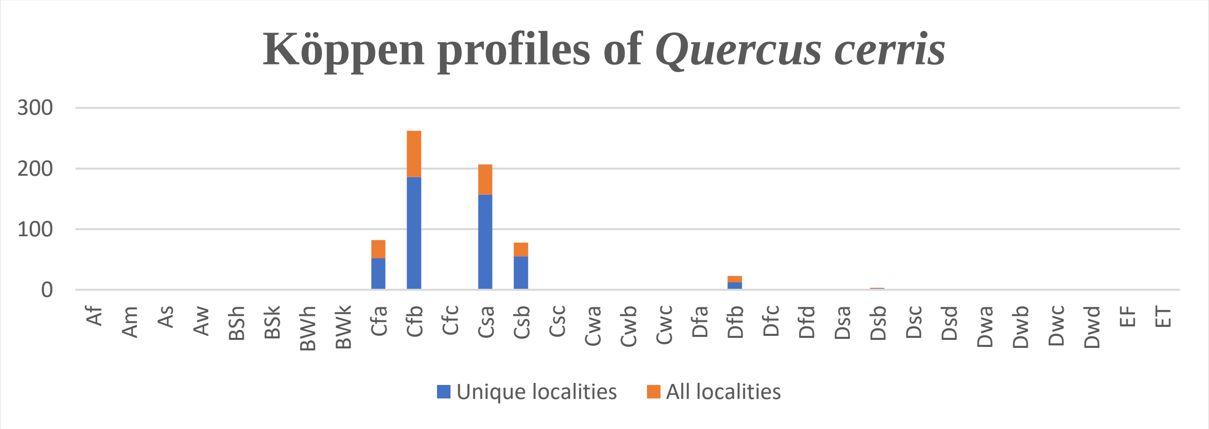


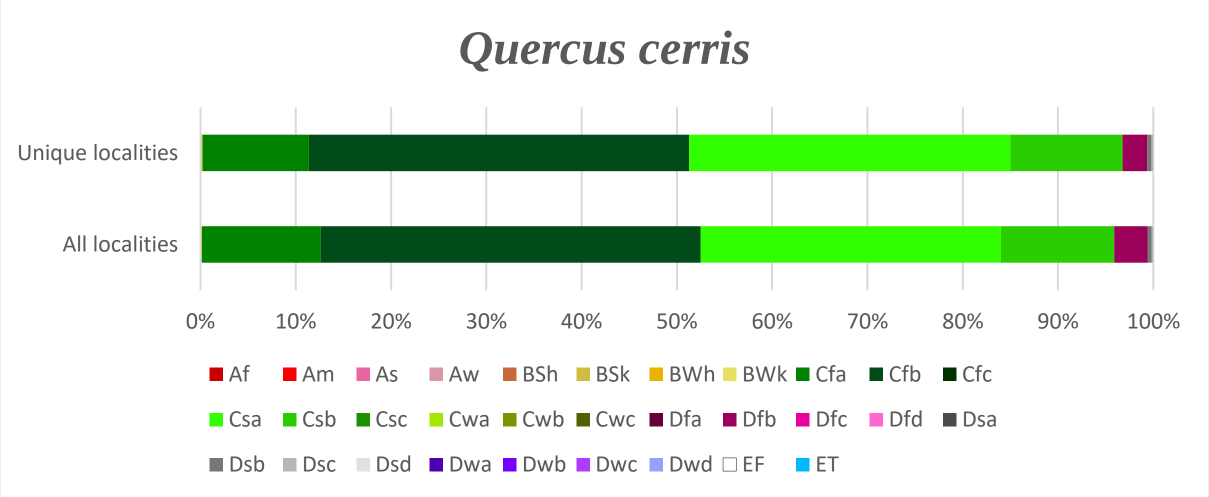


All localities (n = 657); unique localities (n = 466)

**BIOMES – *Quercus cerris***
GBIF localities of *Q. cerris* (preserved specimen)

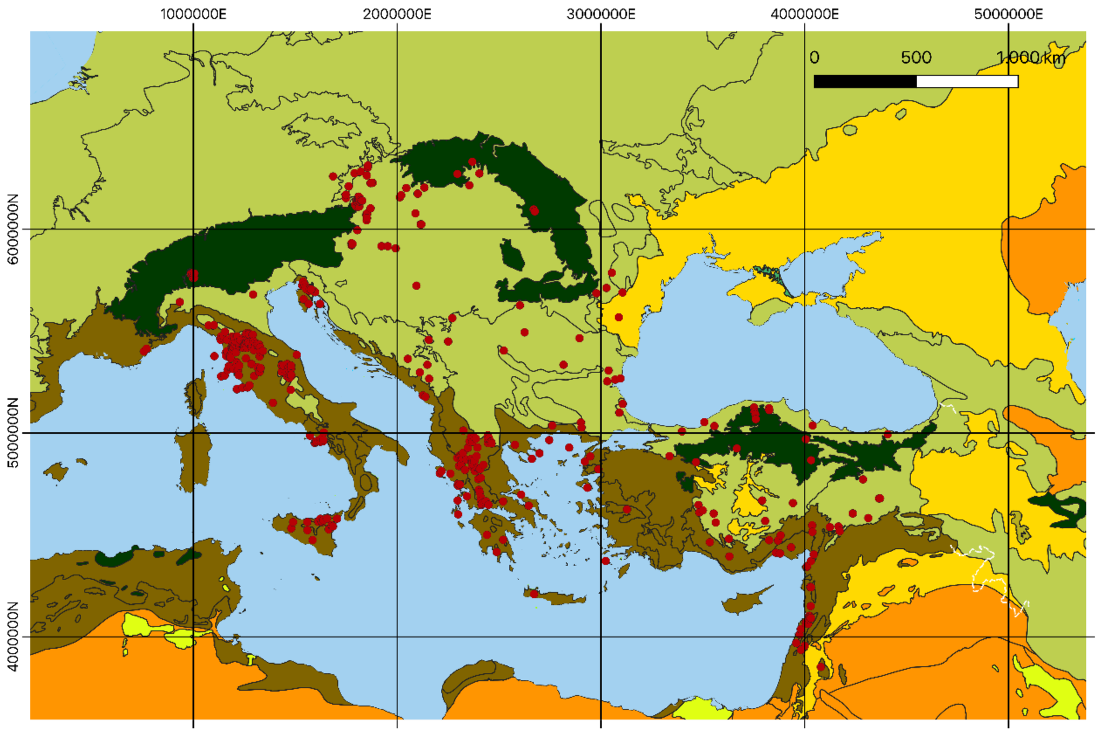


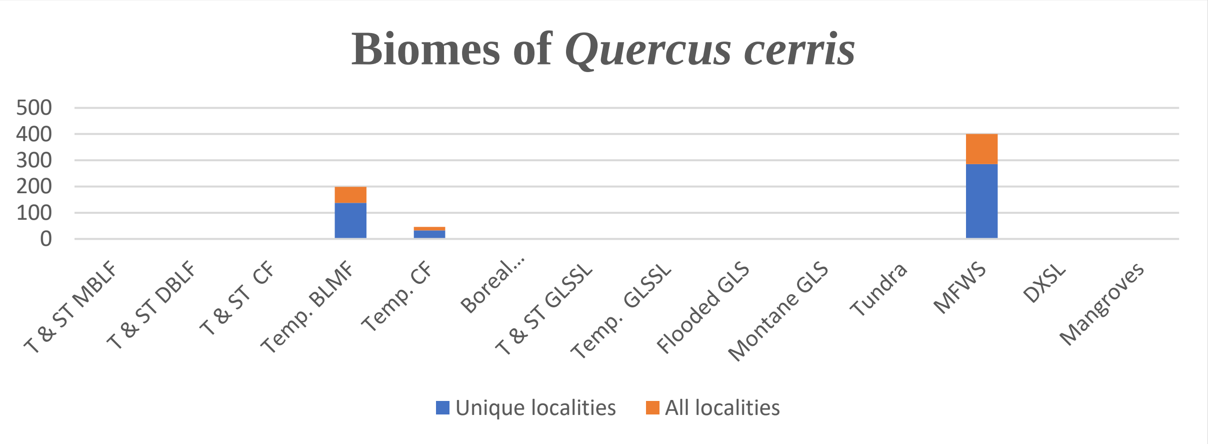


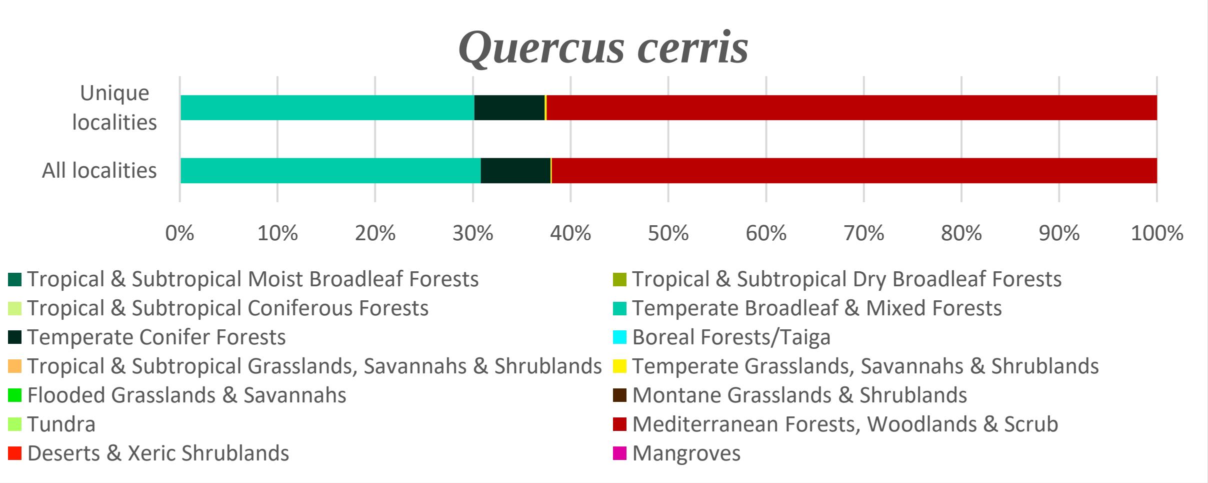


All localities (n = 646); unique localities (n = 458)

**MTCM (x-axis) vs PCQ (y-axis) for *Quercus cerris* (n = 435).** A meridional species (cf. Schroeder 1998) occupying the section's core niche in Western Eurasia and including that of its eastern (Iranian) sister species, *Q. castaneifolia*. Like its Iranian sister, *Q. cerris* avoids the fully Mediterranean habitats with very mild (MTCM > 10 °C), potentially very wet, winters as well as habitats with relatively harsh (MTCM < −5 °C) or very dry winters (PCQ < 50 mm; typical for arid climates of Western Eurasia). It can tolerate ±frequent frosts and/or snow covers (→ MMP and $MT_{min}$ boxplots).

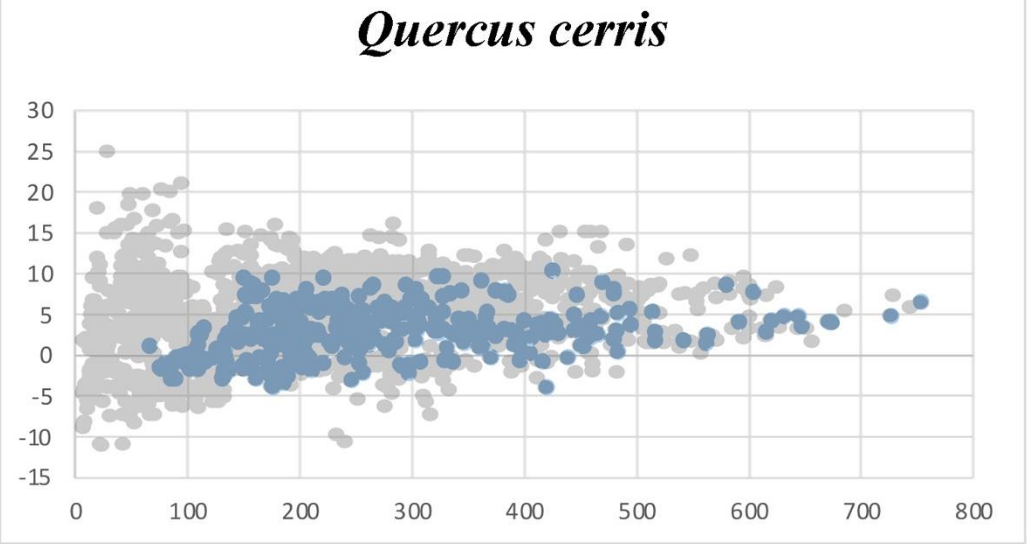


**MMP boxplots for *Quercus cerris* (n = 435)**

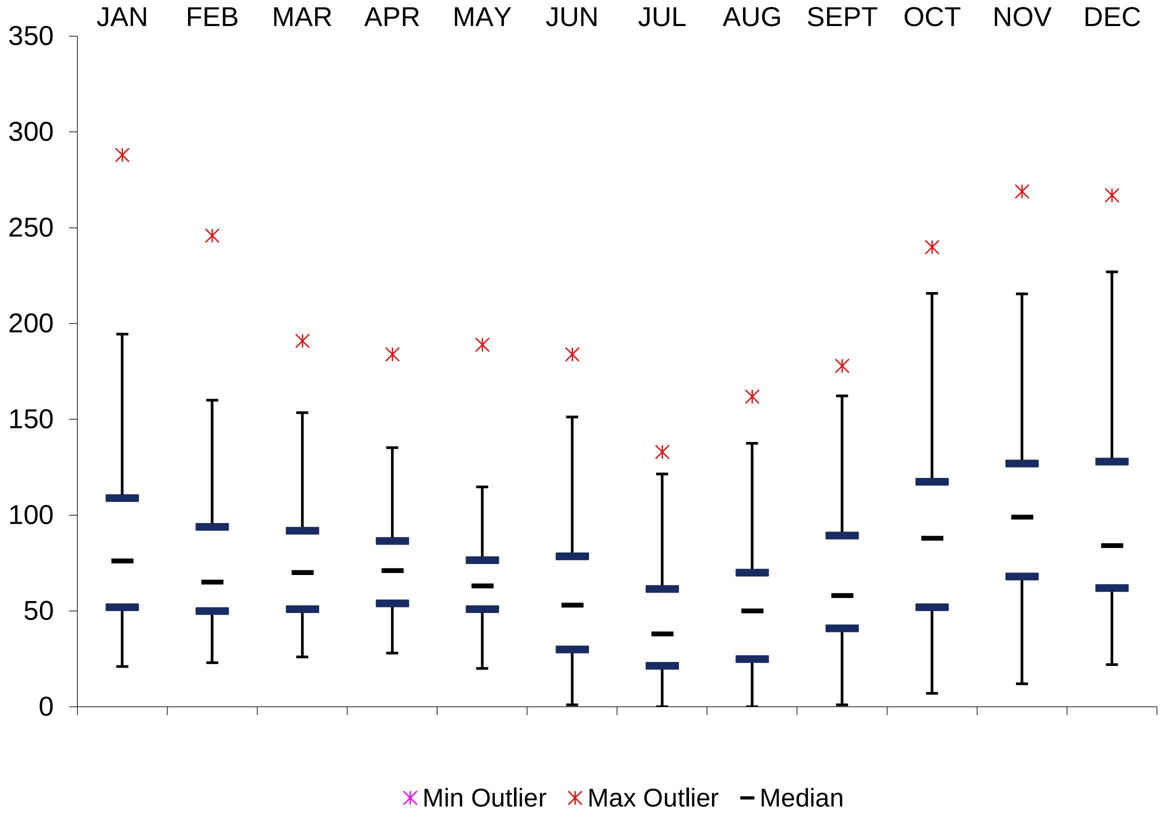

**MMT boxplots for *Quercus cerris* (n = 435)**

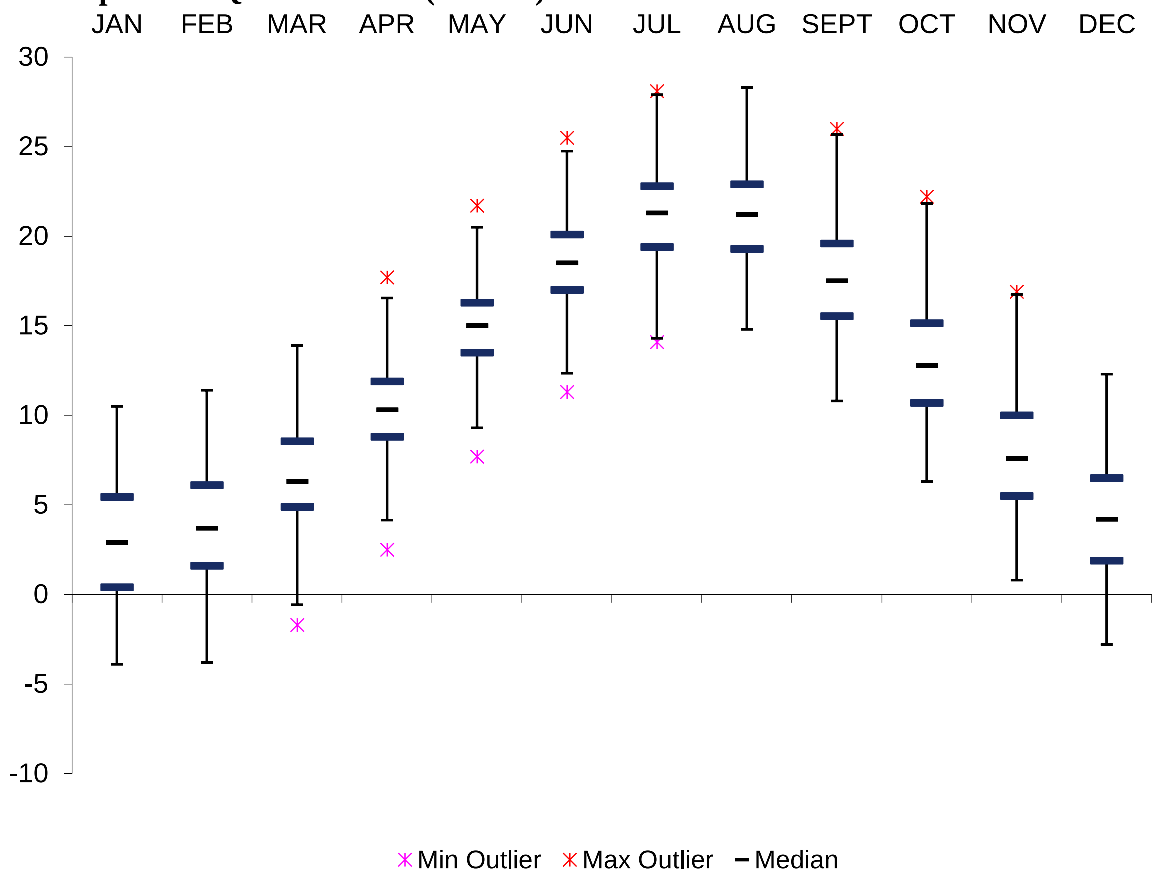


**MT$_{min}$ boxplots for *Quercus cerris* (n = 435)**

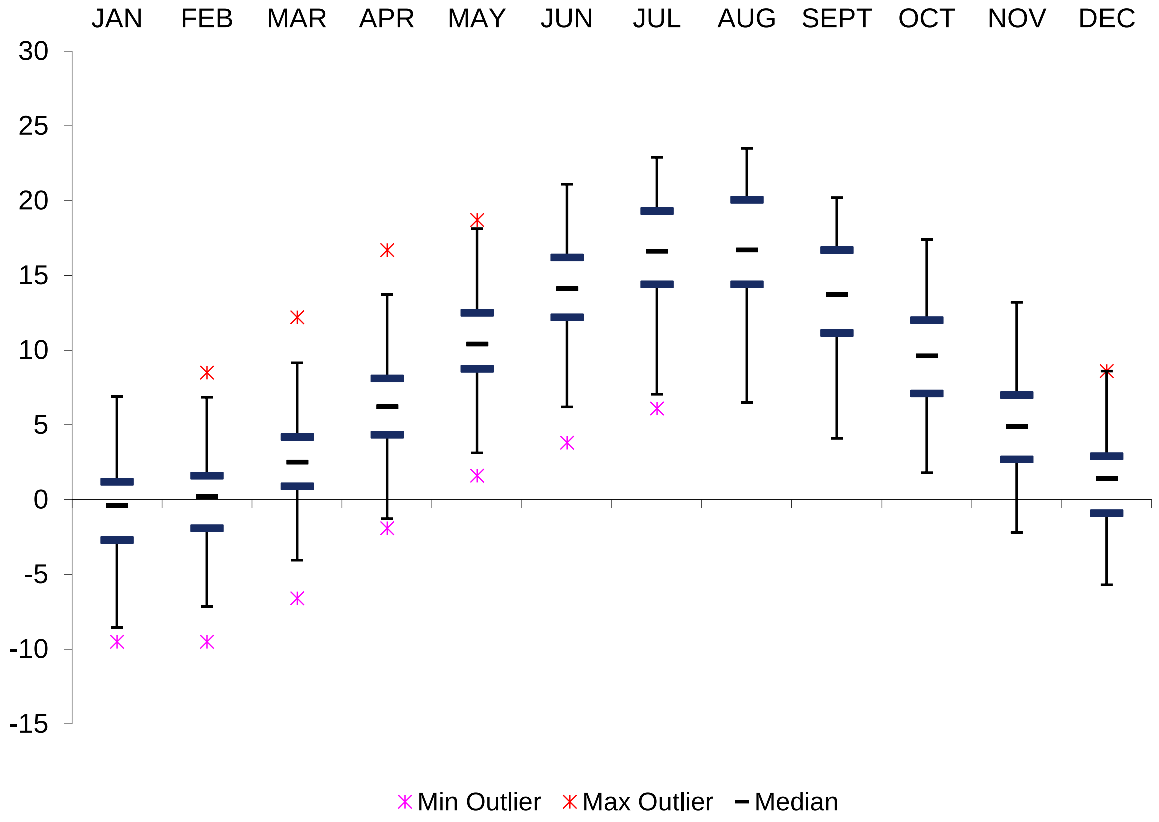

## Quercus *sect.* Cerris *subsect.* Libani

Cumulative niche of *Q. afares, Q. euboica, Q. libani, Q. trojana* (s.str.)

**KÖPPEN MAP** (n = 227)

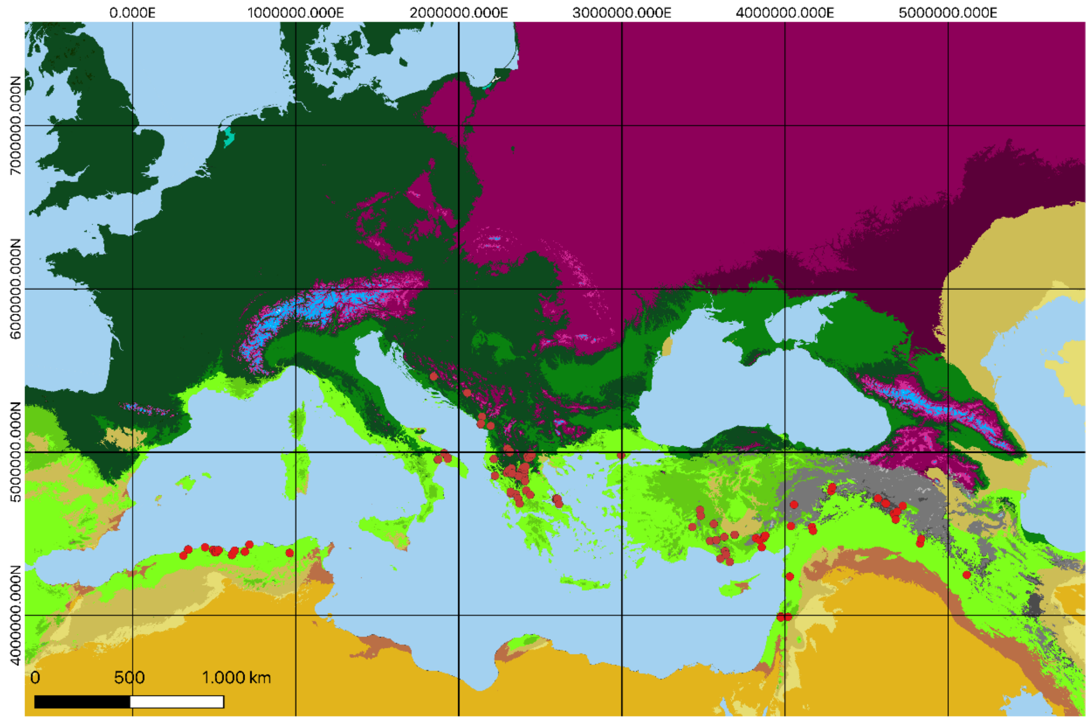


**MTCM (x-axis) vs PCQ (y-axis) for members subsect. *Libani* (n = 141).** In contrast to its sister lineage, the species of subsect. *Libani* prefer different niches and extend into fringe/marginal habitats. Like subsect. *Cerris*, they prefer climates with moderate to very wet winters while avoiding fully Mediterranean habitats with very mild winters (MTCM > 10 °C)

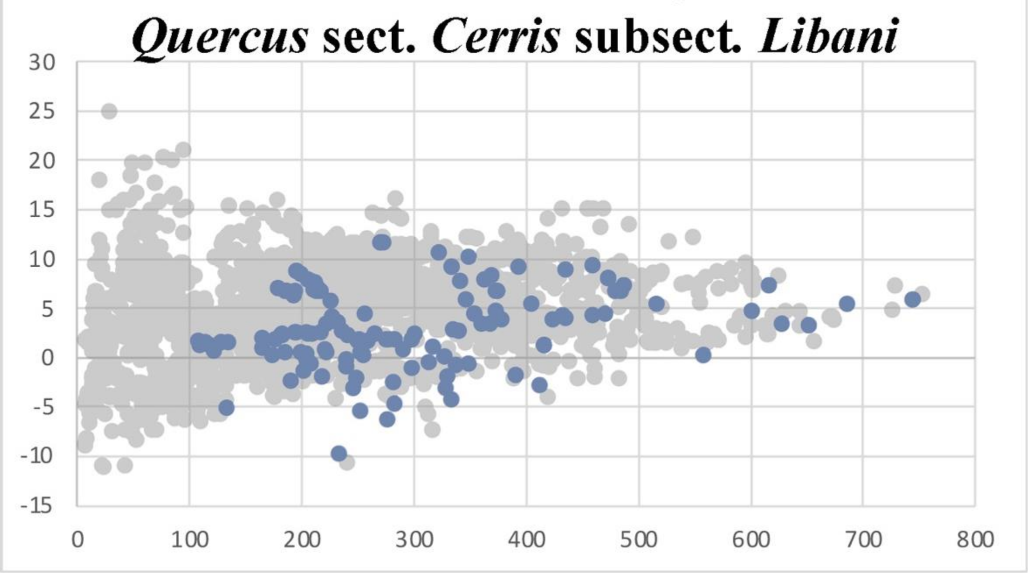

**KÖPPEN PROFILES – *Quercus afares*** Pomel, 1875
GBIF localities of *Q. afares* (preserved specimen)

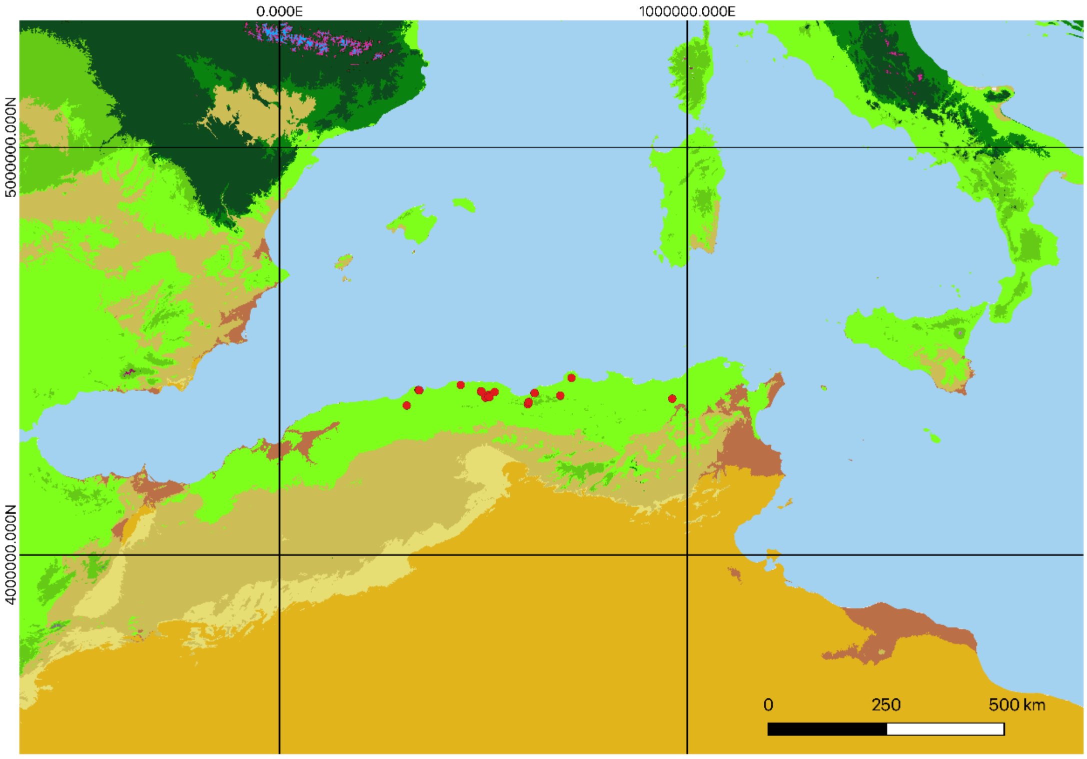


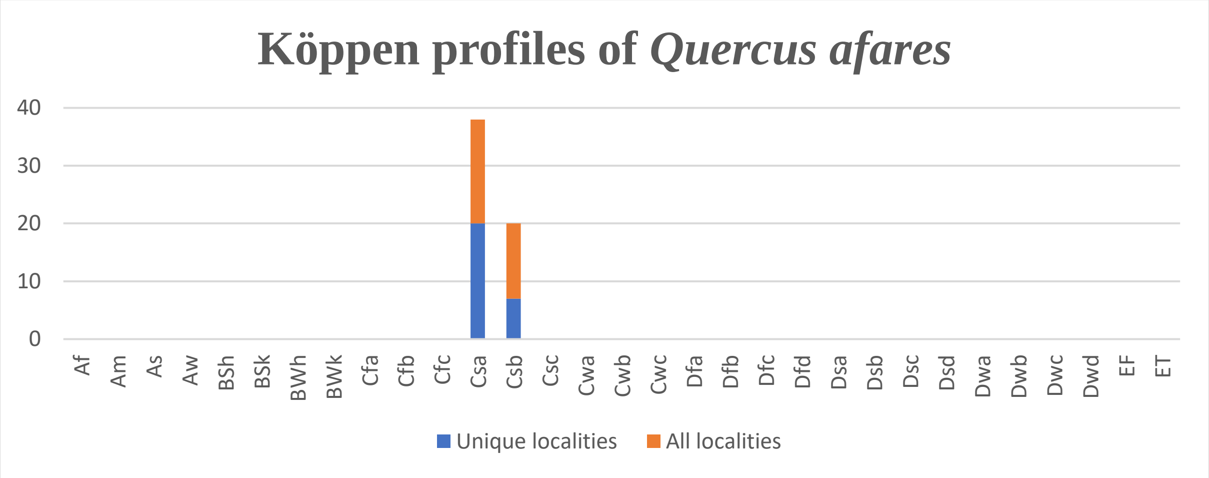


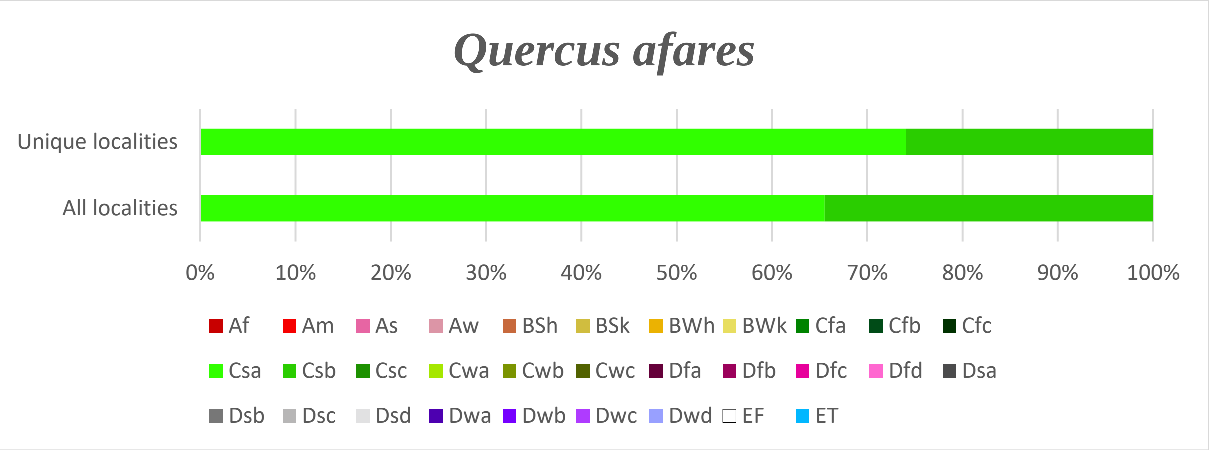


All localities (n = 58); unique localities (n = 27)

**BIOMES – *Quercus afares***

GBIF localities of *Q. afares* (preserved specimen)

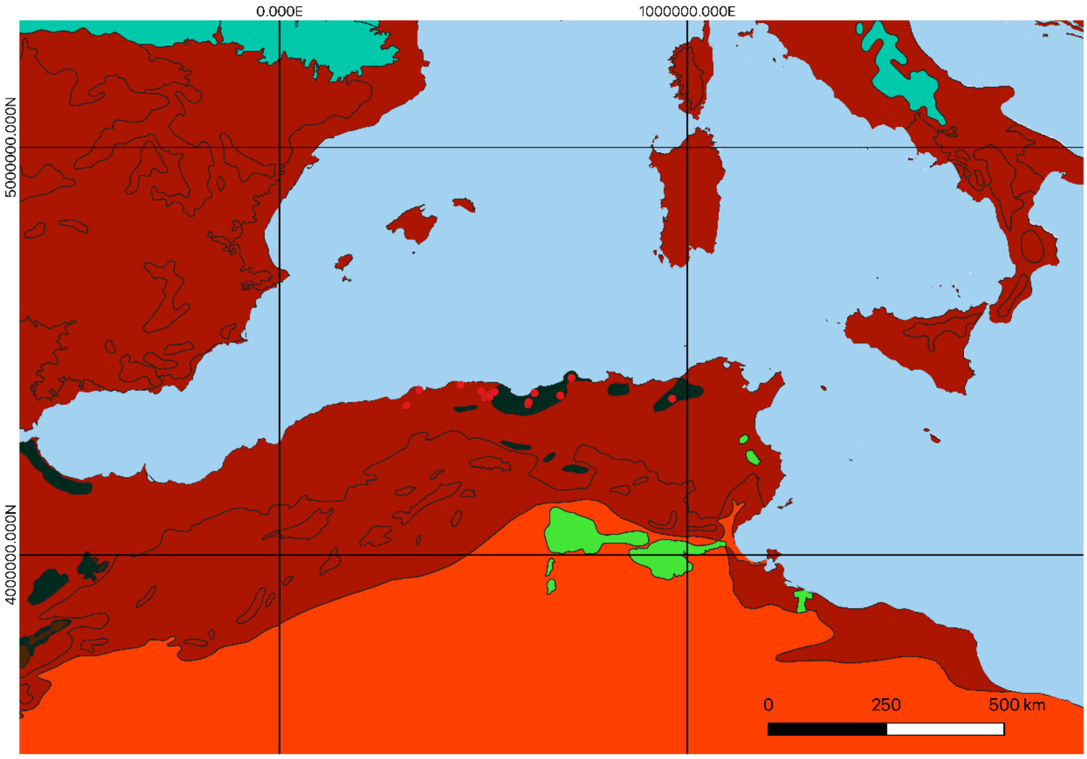


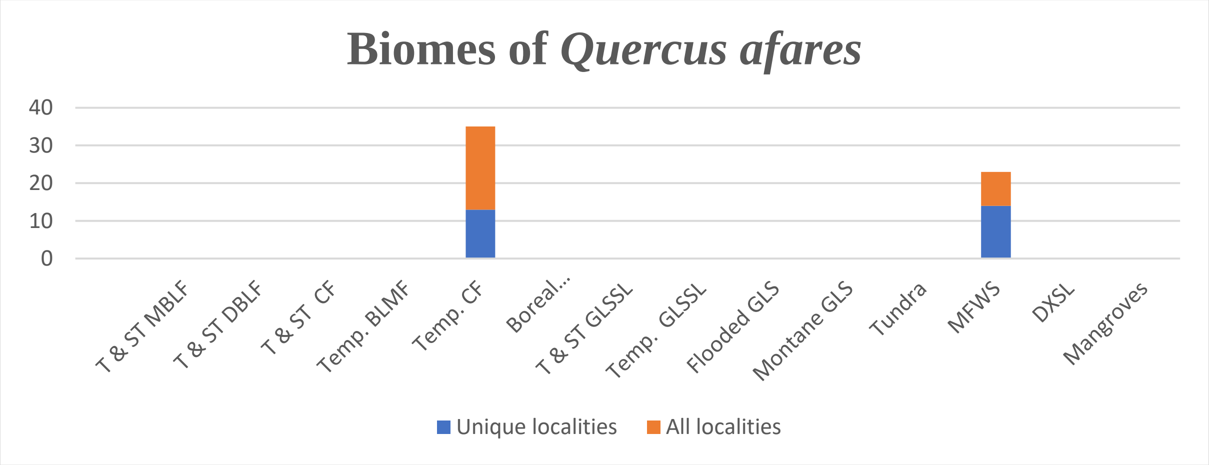


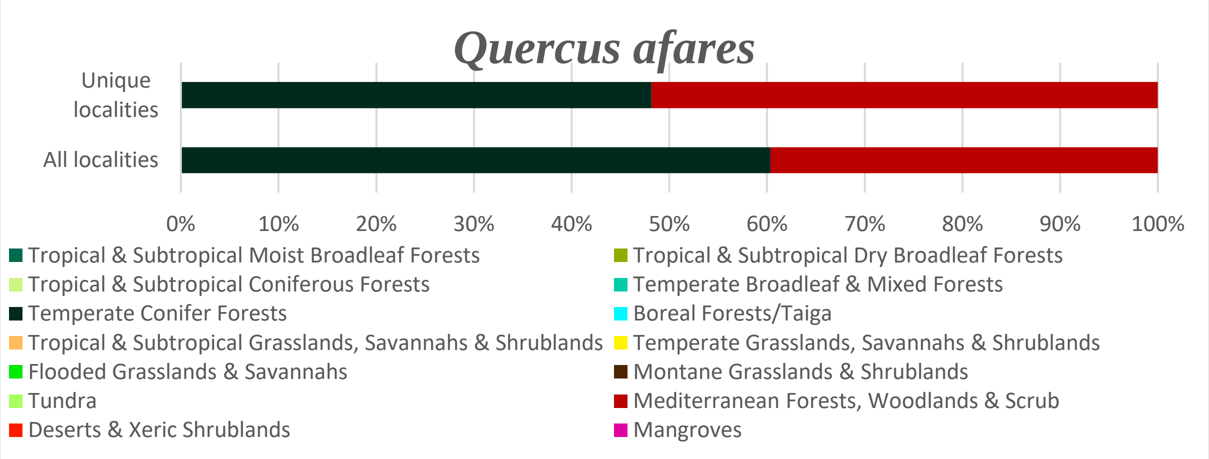


All localities (n = 58); unique localities (n = 27)

**MTCM (x-axis) vs PCQ (y-axis) for *Quercus afares* (n = 26).** In contrast to the equally geographically restricted *Q. castaneifolia*, *Q. afares* shows a wider range and stronger preference for localities with substantial winter precipitation (PCQ > 250 mm) and very little risk for frosts or snow (MTCM > 0 °C; → $MT_{min}$ boxplot).

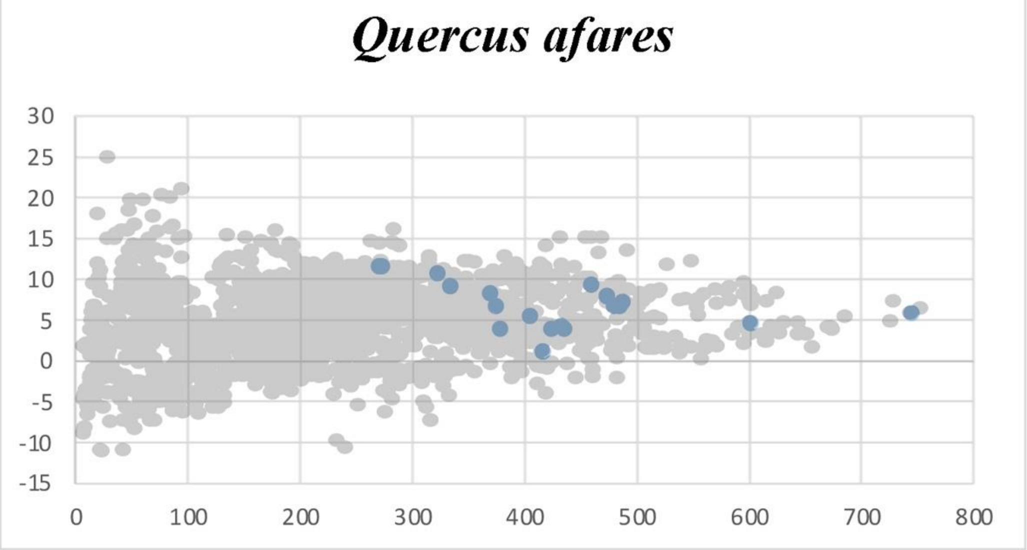


**MMP boxplots for *Quercus afares* (n = 26)**

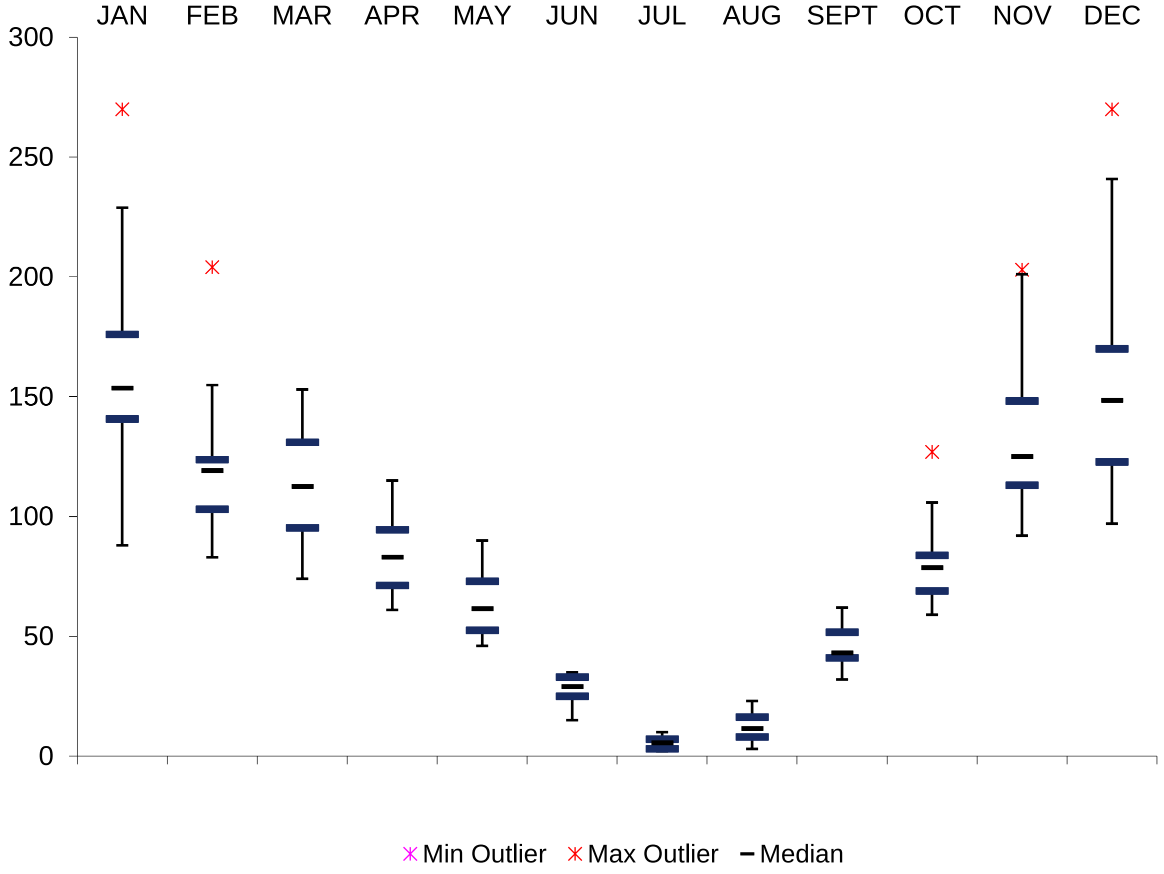

**MMT boxplots for *Quercus afares* (n = 26)**

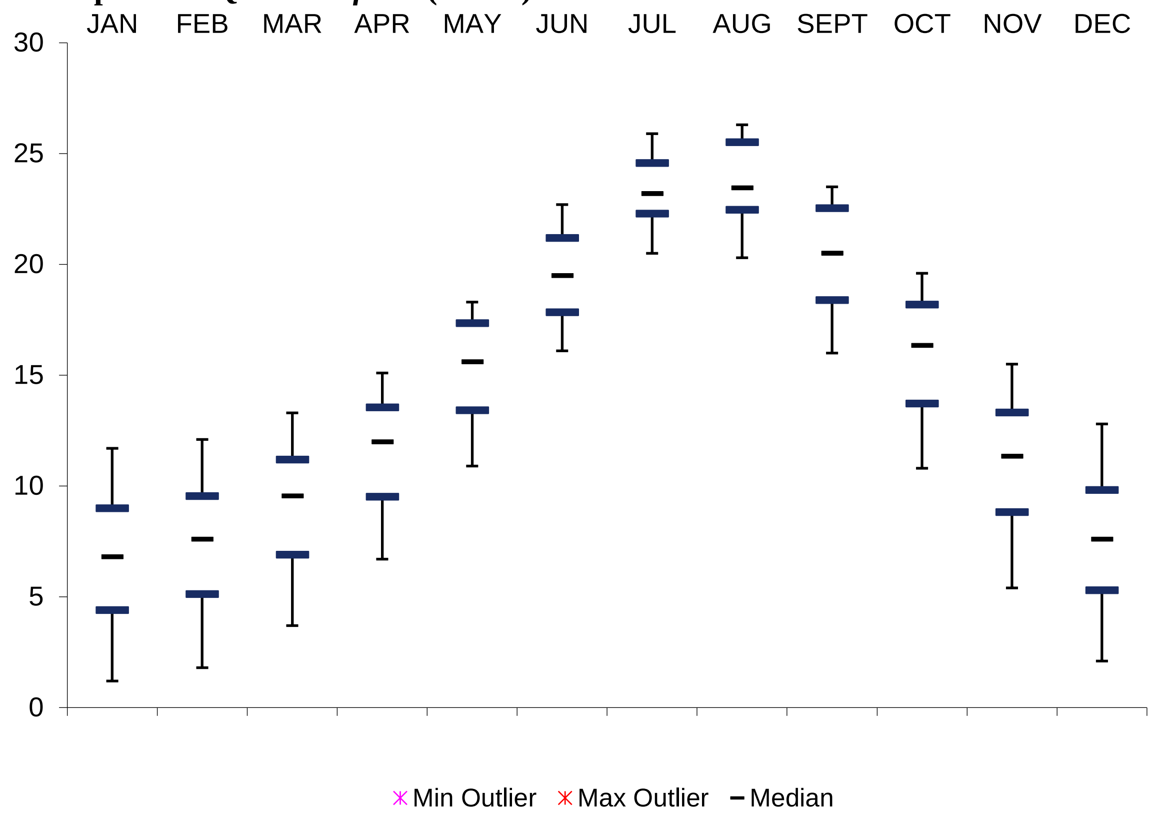


**MT$_{min}$ boxplots for *Quercus afares* (n = 26)**

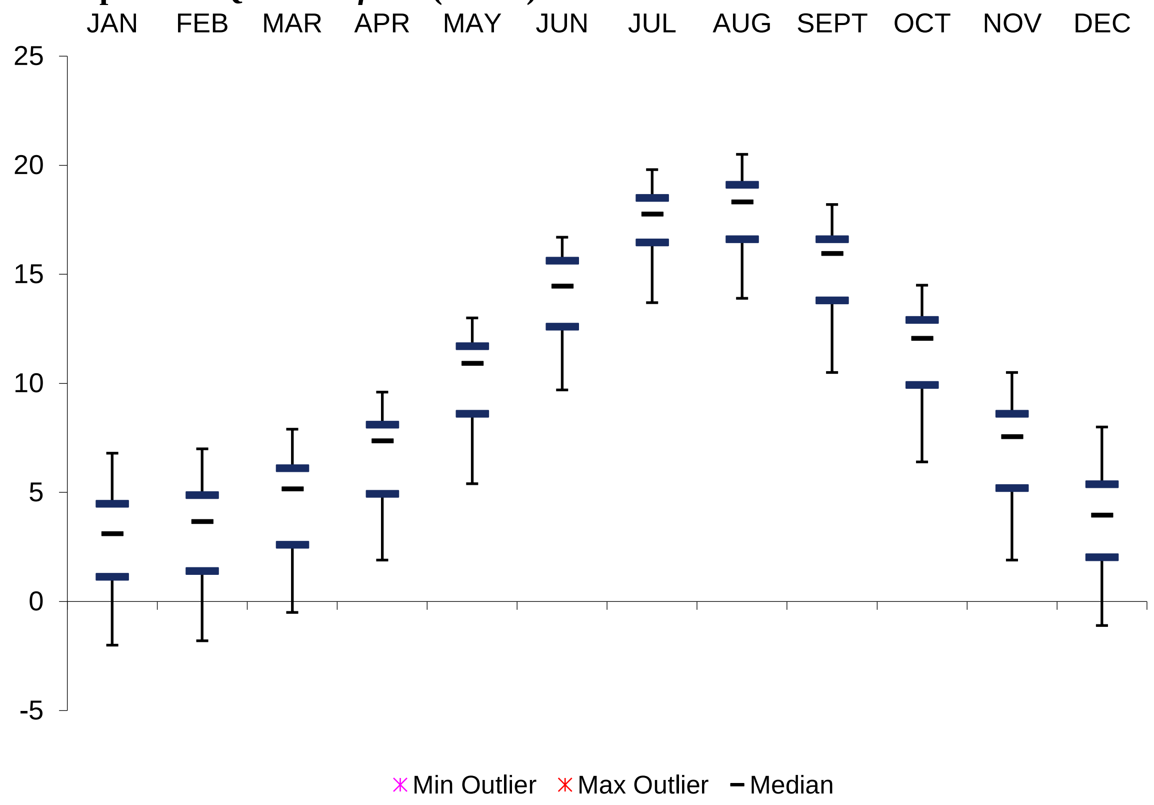

**KÖPPEN PROFILES – *Quercus euboica* Papaioannou, 1949**
GBIF localities of *Q. euboica* (preserved specimen; extracted from *Q. trojana* GBIF data)

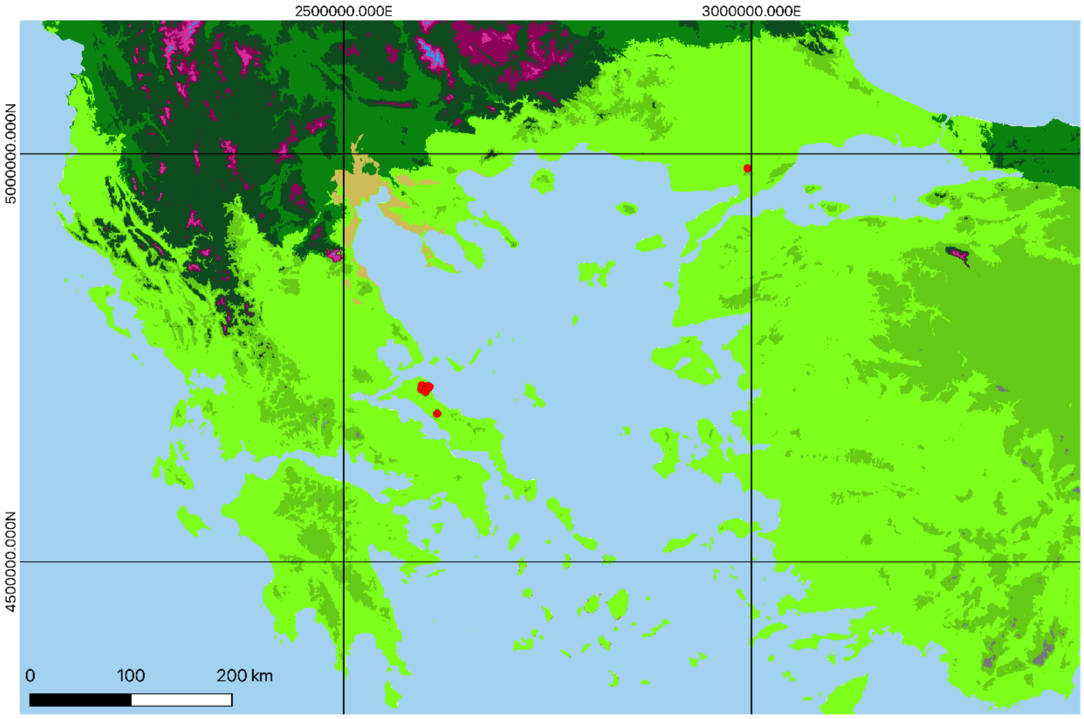


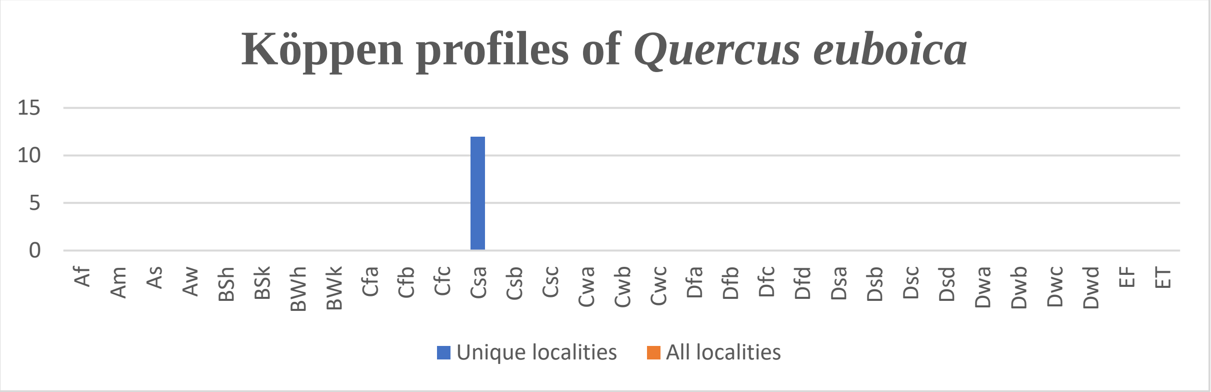


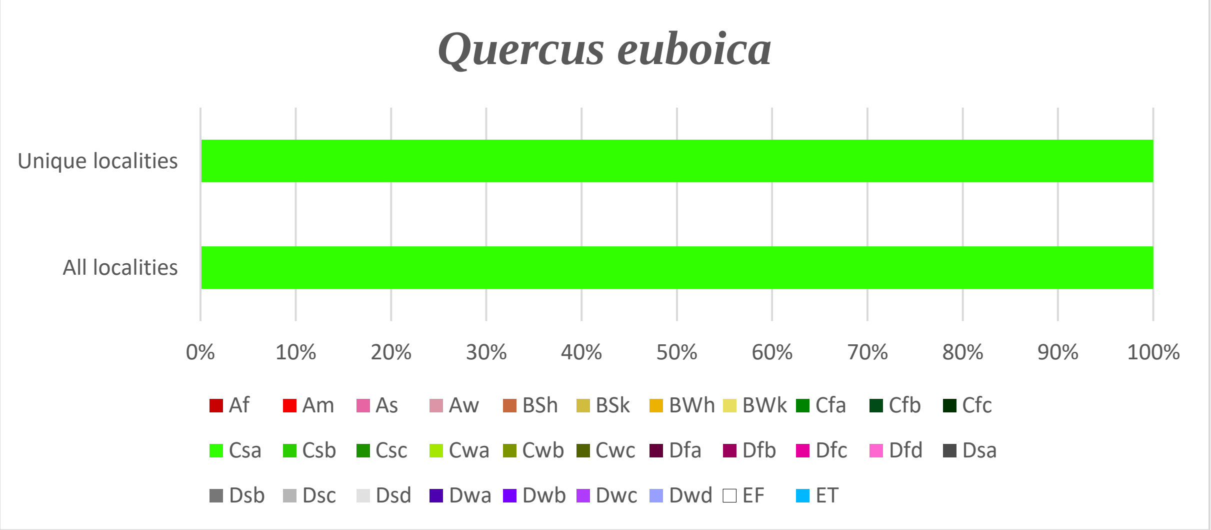


All localities (n = 12); unique localities (n = 12)

**BIOMES – *Quercus euboica***
GBIF localities of *Q. euboica* (preserved specimen)

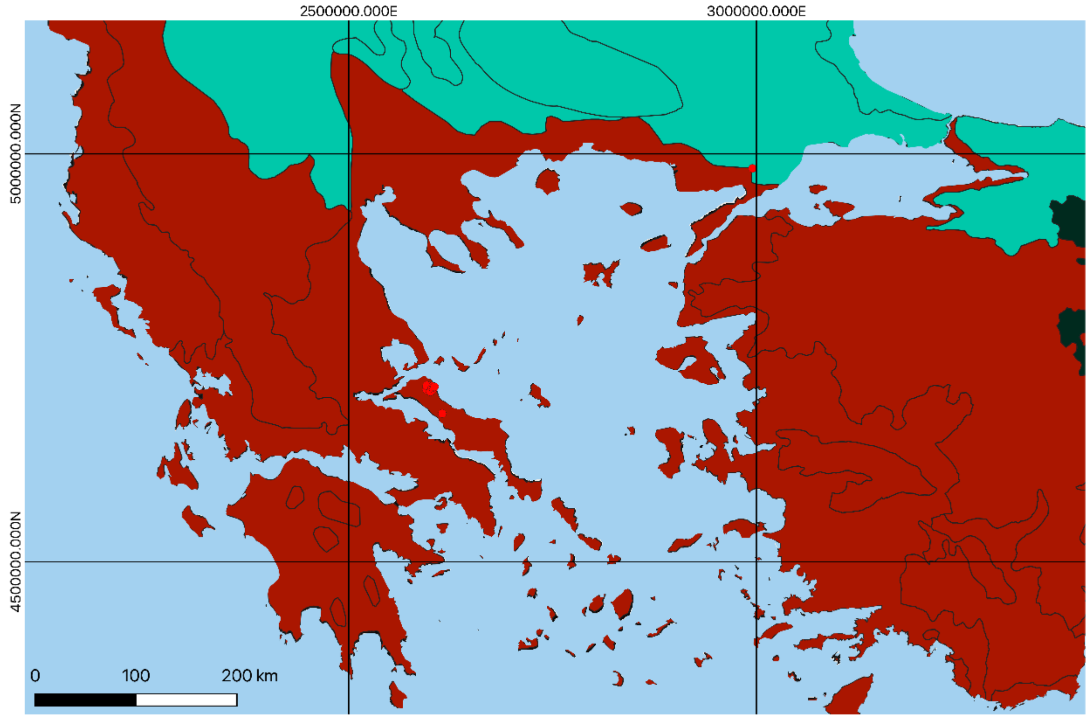


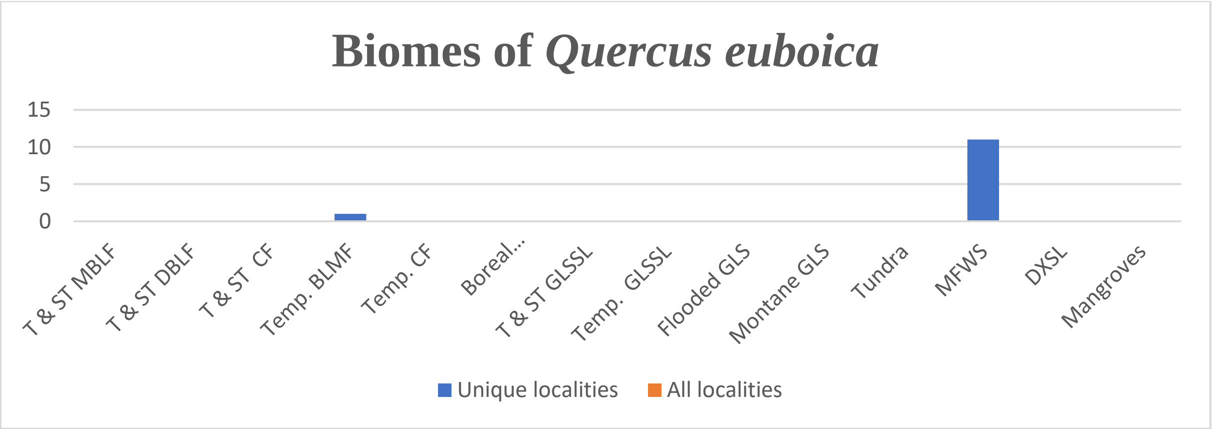


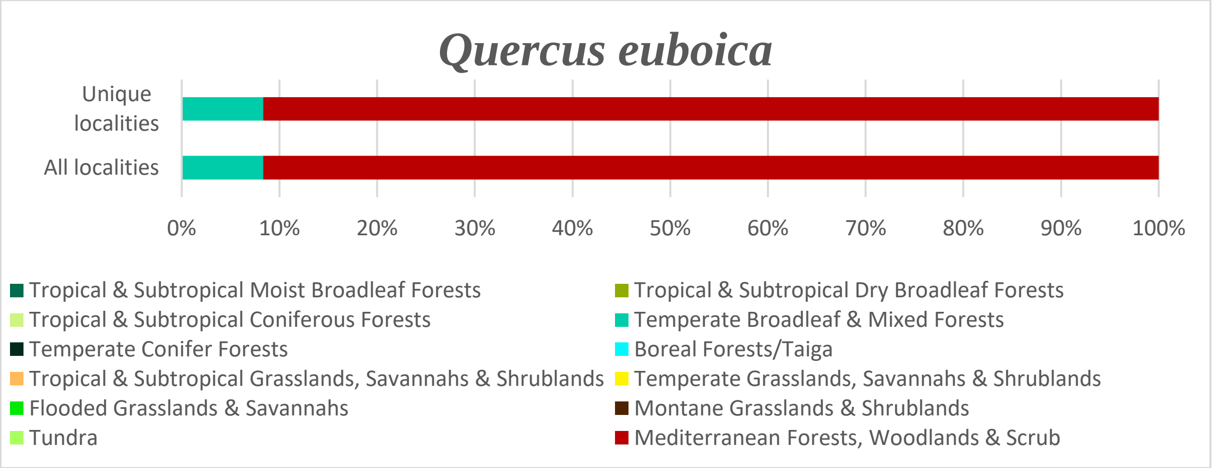


All localities (n = 12); unique localities (n = 12)

**MTCM (x-axis) vs PCQ (y-axis) for *Quercus euboica* (n = 12).** Being restricted to the island of Euboea in western Greece, *Q. euboica* thrives in a typical (moist-)Mediterranean climate with (very) mild winters. Summers are dry but there is no extensive draught period (→ MMP boxplot; all months > ~10 mm)

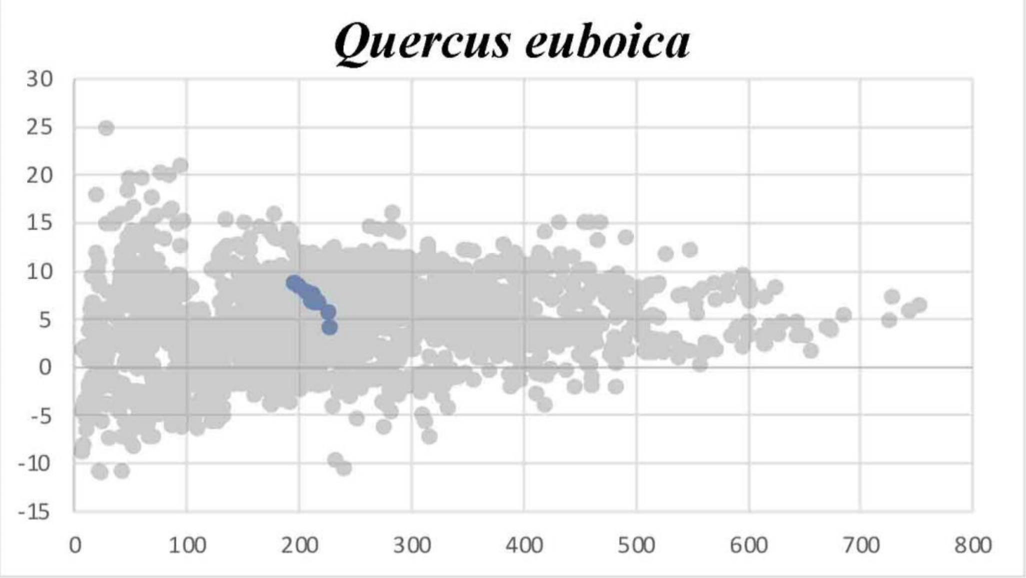


**MMP boxplots for *Quercus euboica* (n = 12)**

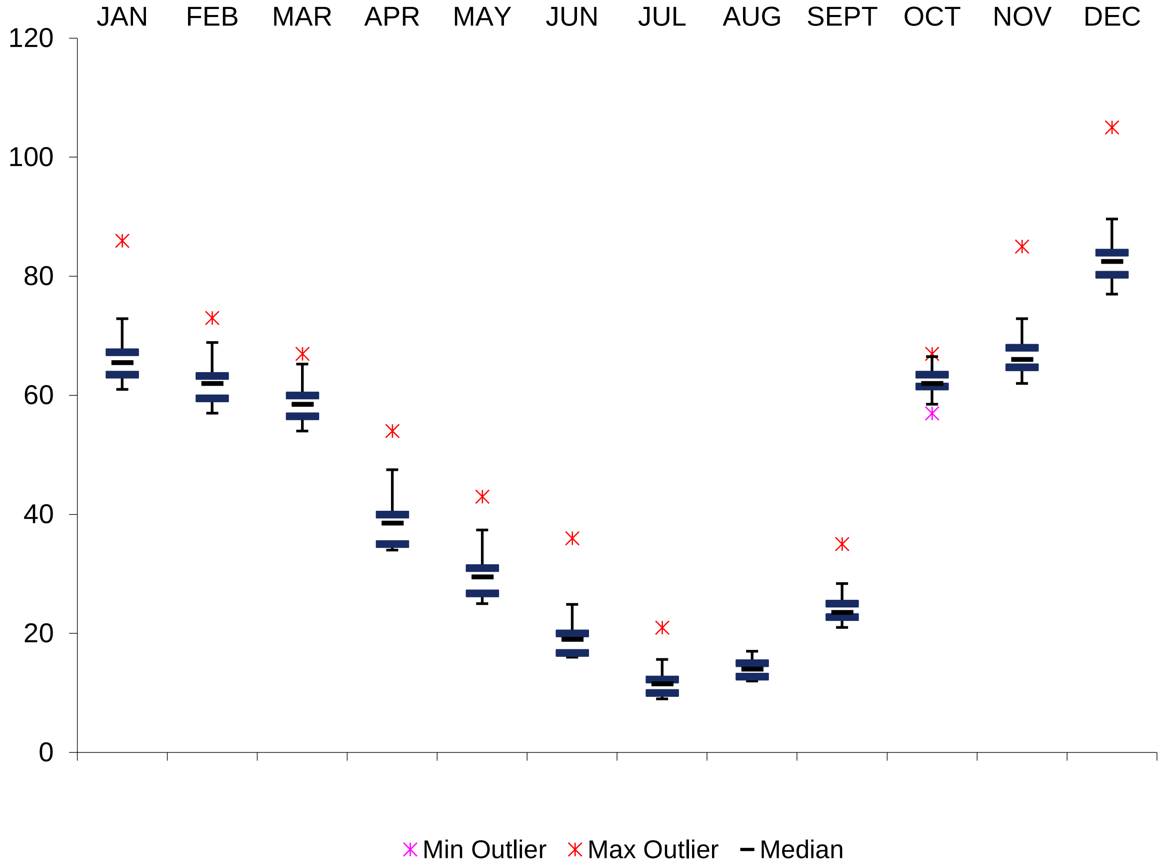

**MMT boxplots for *Quercus euboica* (n = 12)**

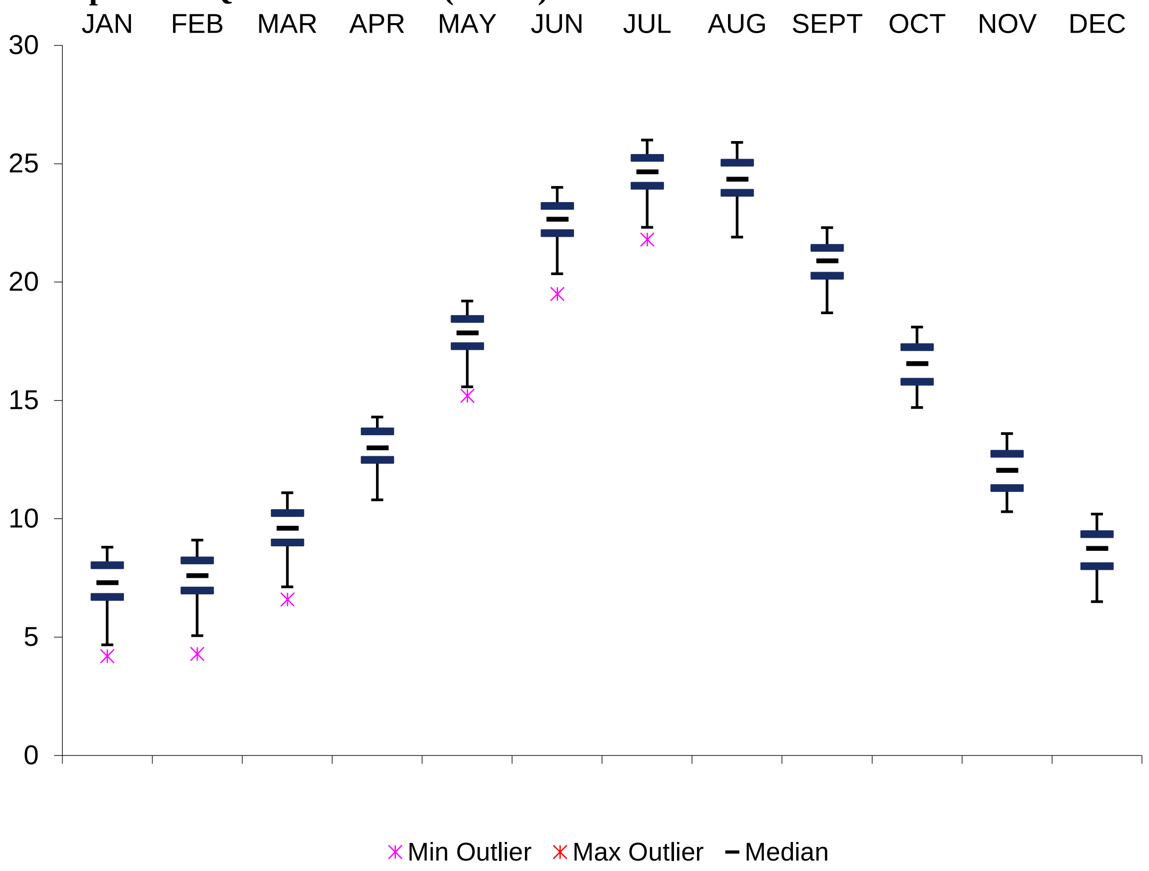


**$MT_{min}$ boxplots for *Quercus euboica* (n = 12)**

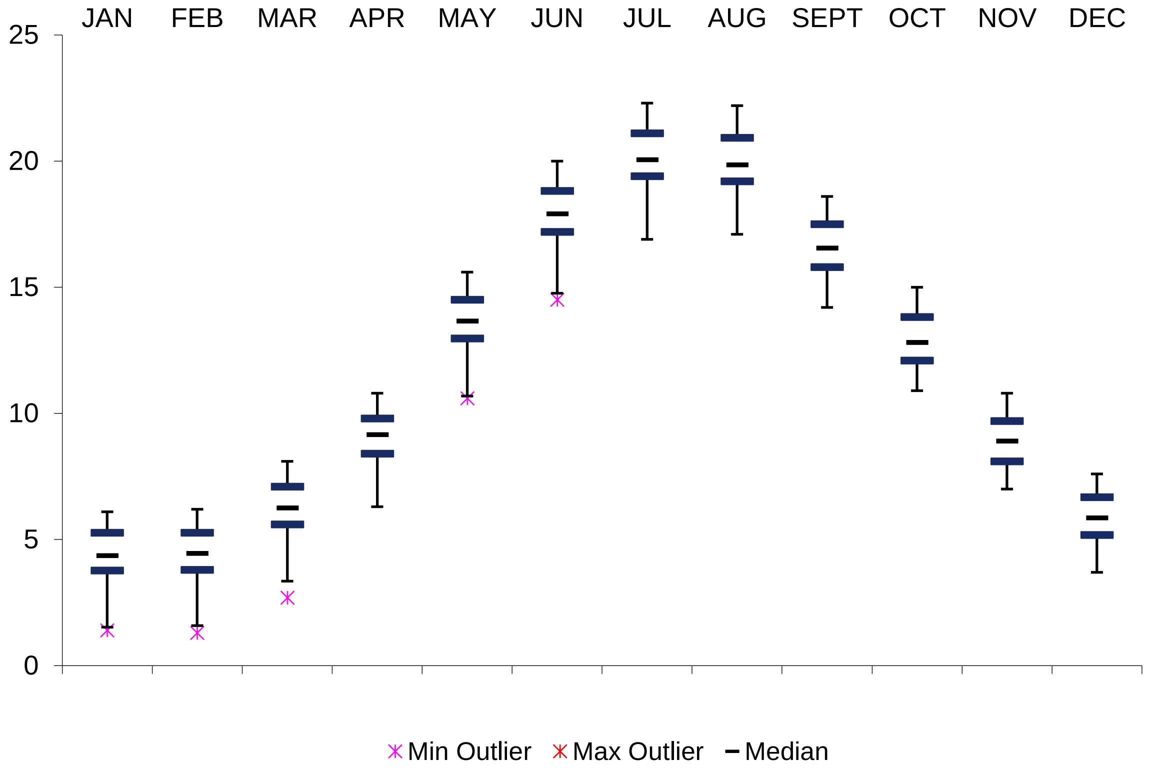

**KÖPPEN PROFILES – *Quercus libani* Oliv., 1804**
GBIF localities of *Q. libani* (preserved specimen)

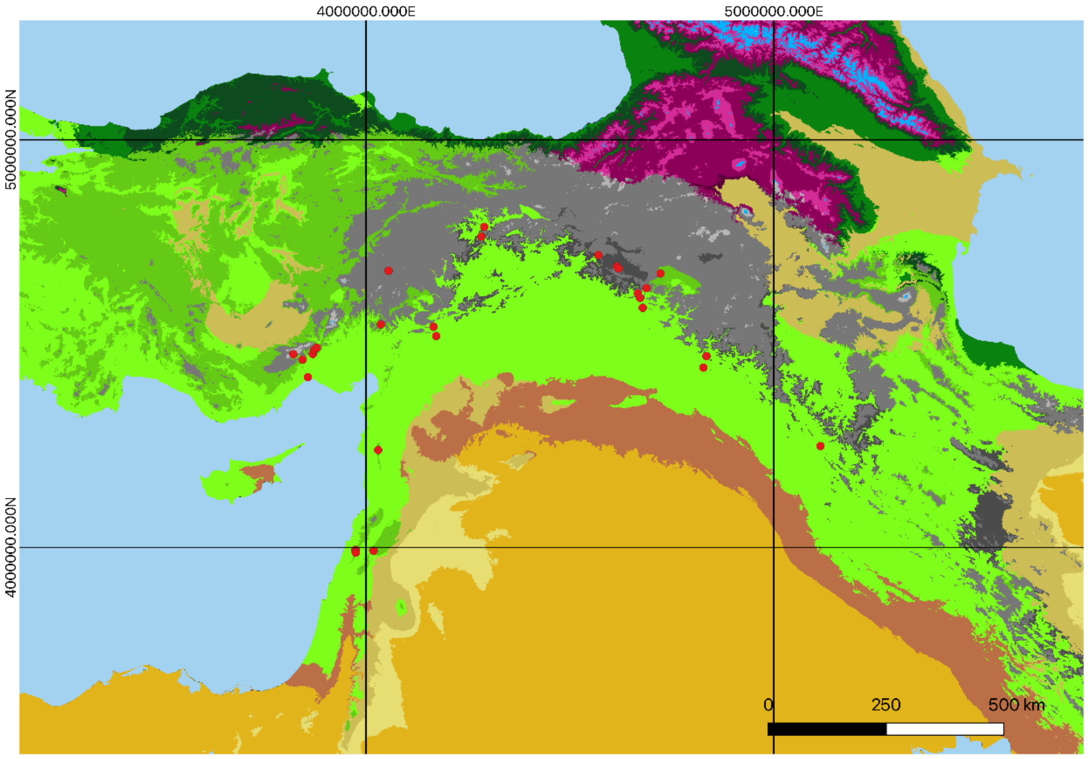


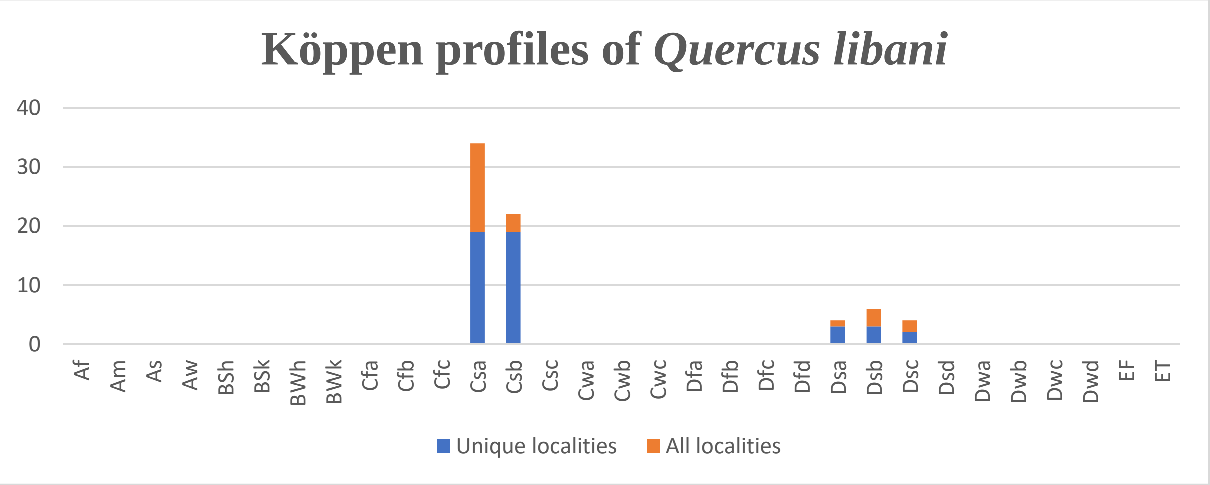


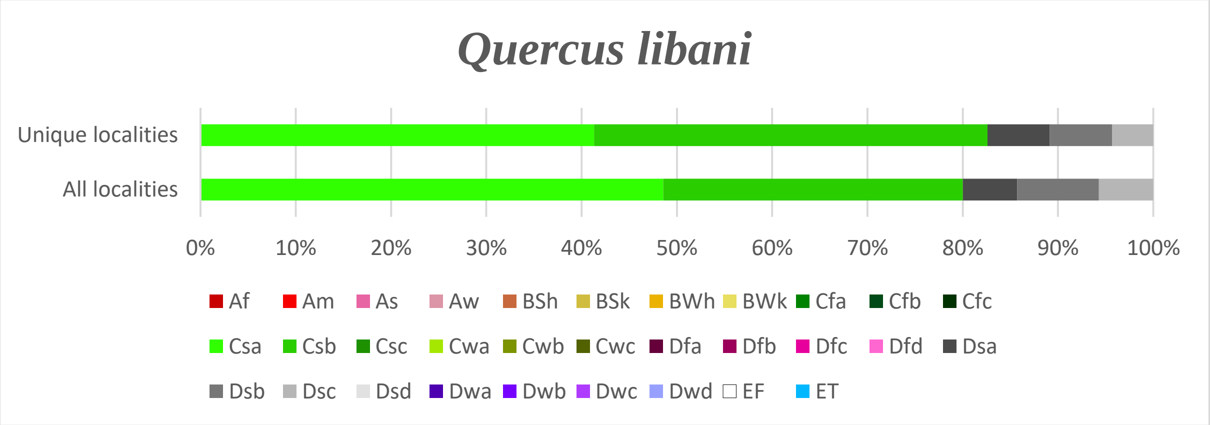


All localities (n = 70); unique localities (n = 46)

**BIOMES – *Quercus libani***
GBIF localities of *Q. libani* (preserved specimen)

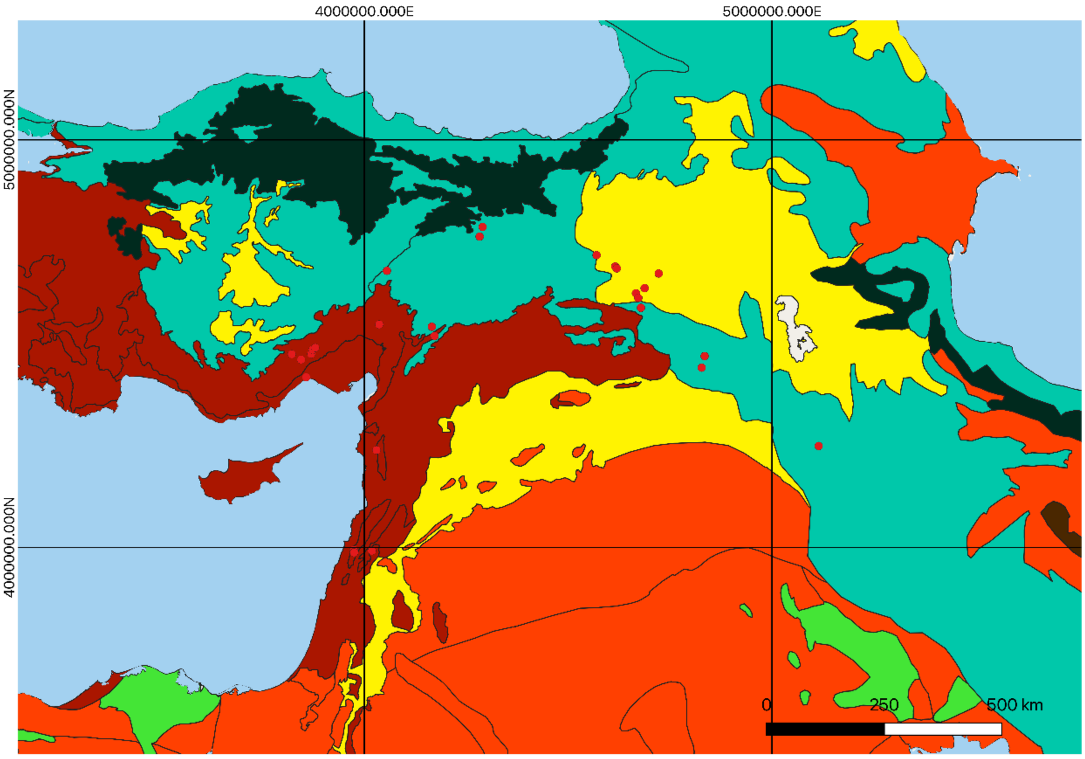


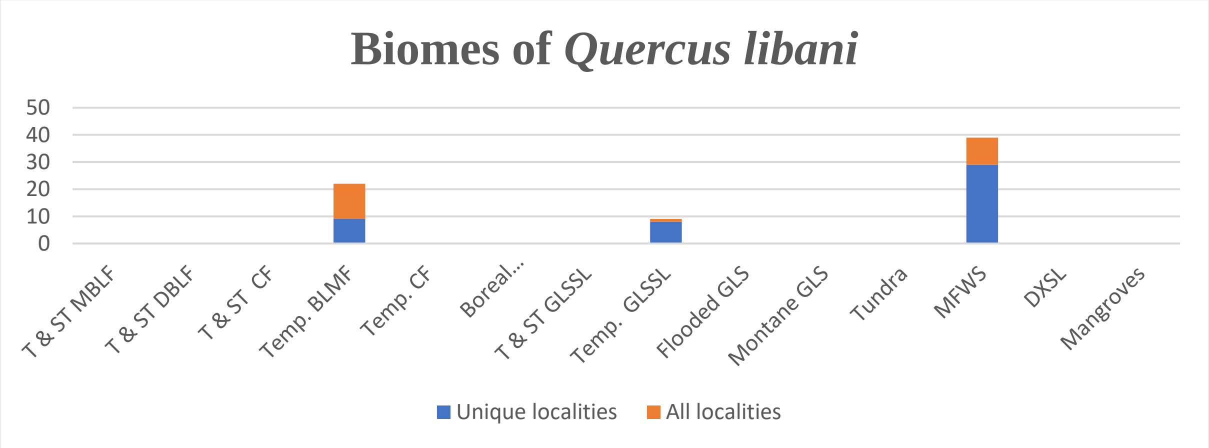


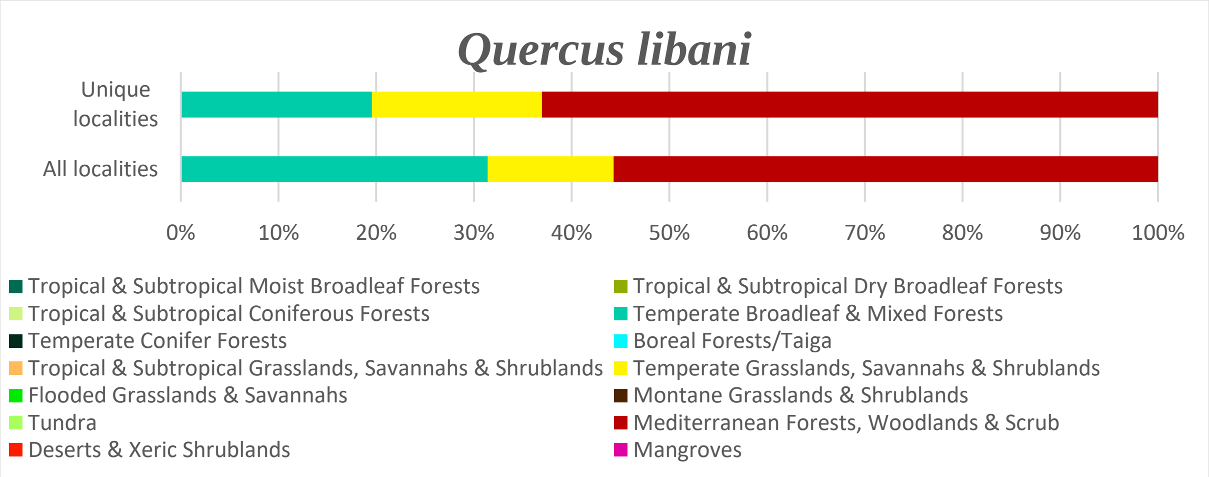


All localities (n = 70); unique localities (n = 46)

**MTCM (x-axis) vs PCQ (y-axis) for *Quercus libani*, (n = 29).** This species appears to be the most frost and winter-cold resistant member of sect. *Cerris* in Western Eurasia, adapted to profound summer-draught and frosty/ snowy winters (→ MMP and $MT_{min}$ boxplots). Climatically it has only very little overlap with its sibling *Q. afares*.

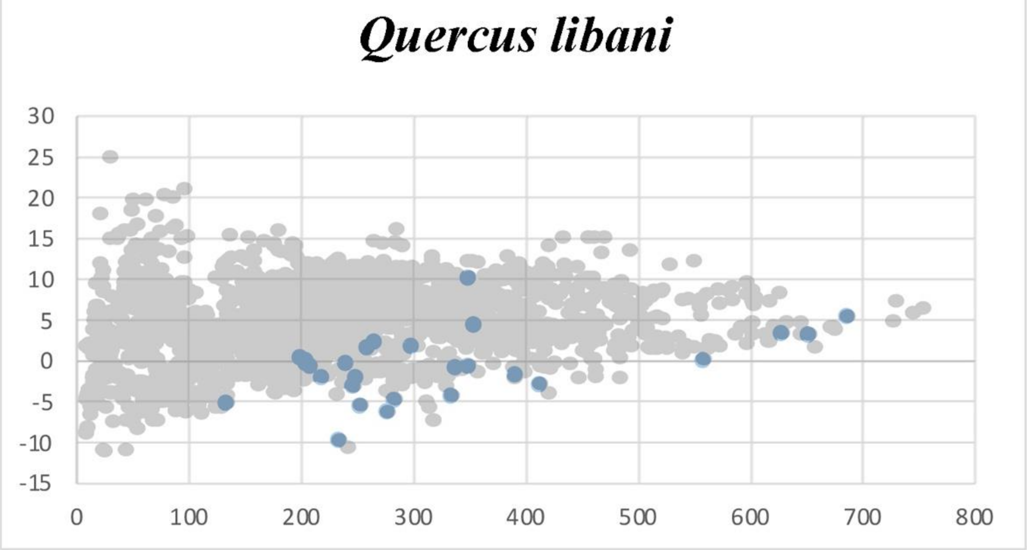


**MMP boxplots for *Quercus libani* (n = 29)**

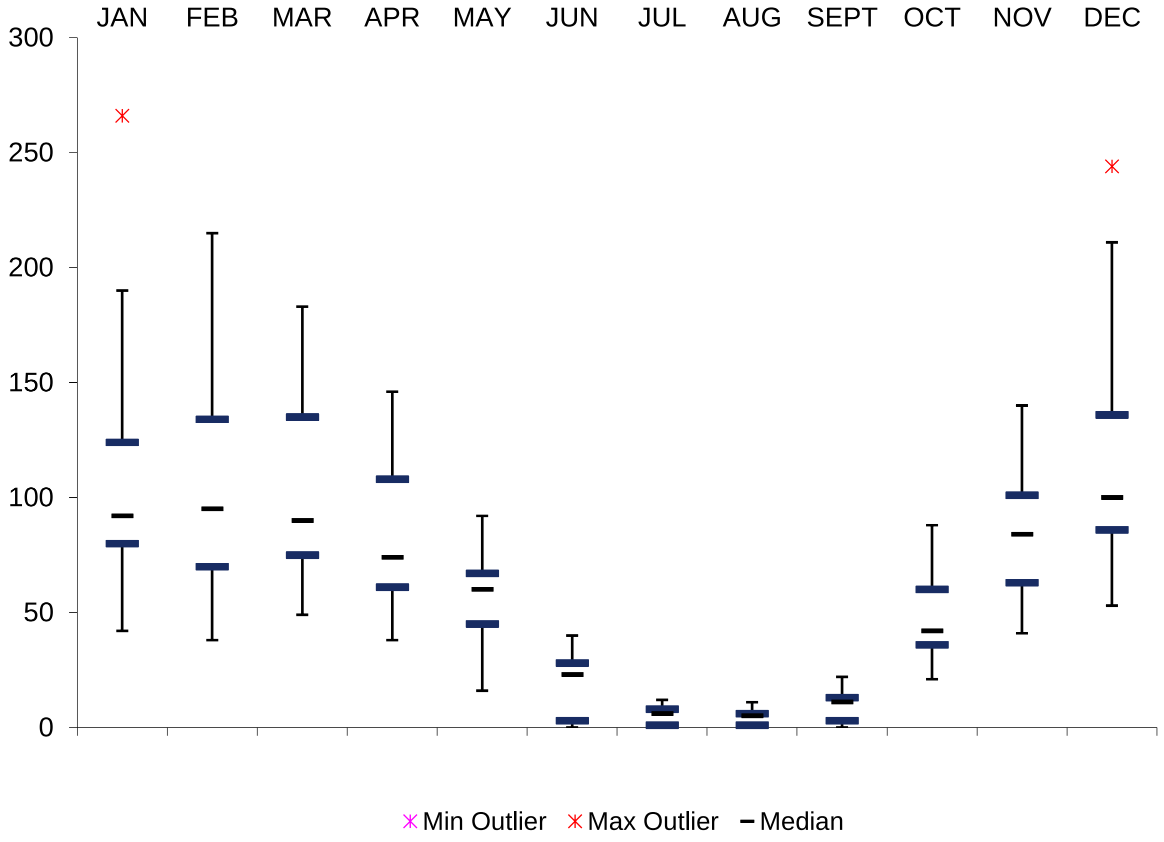

**MMT boxplots for *Quercus libani* (n = 29)**

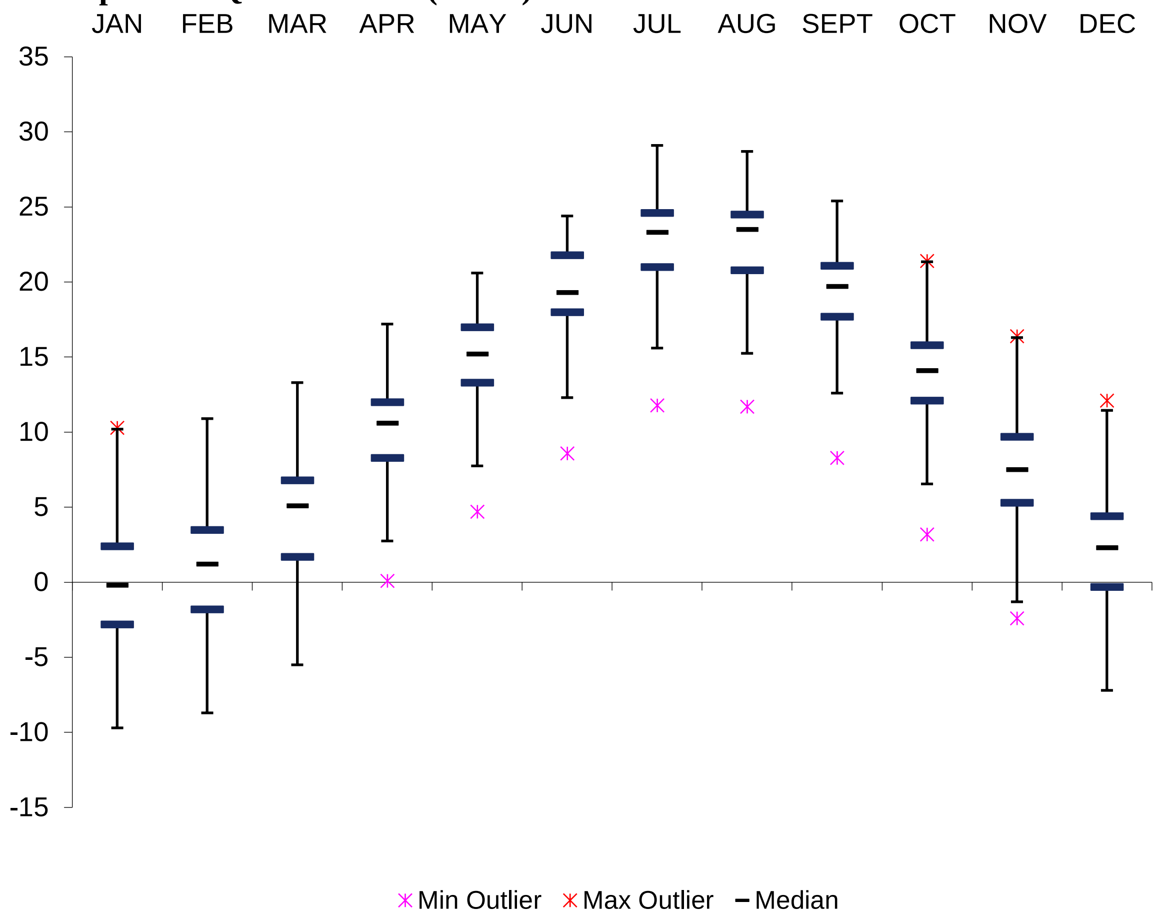


**$MT_{min}$ boxplots for *Quercus libani* (n = 29)**

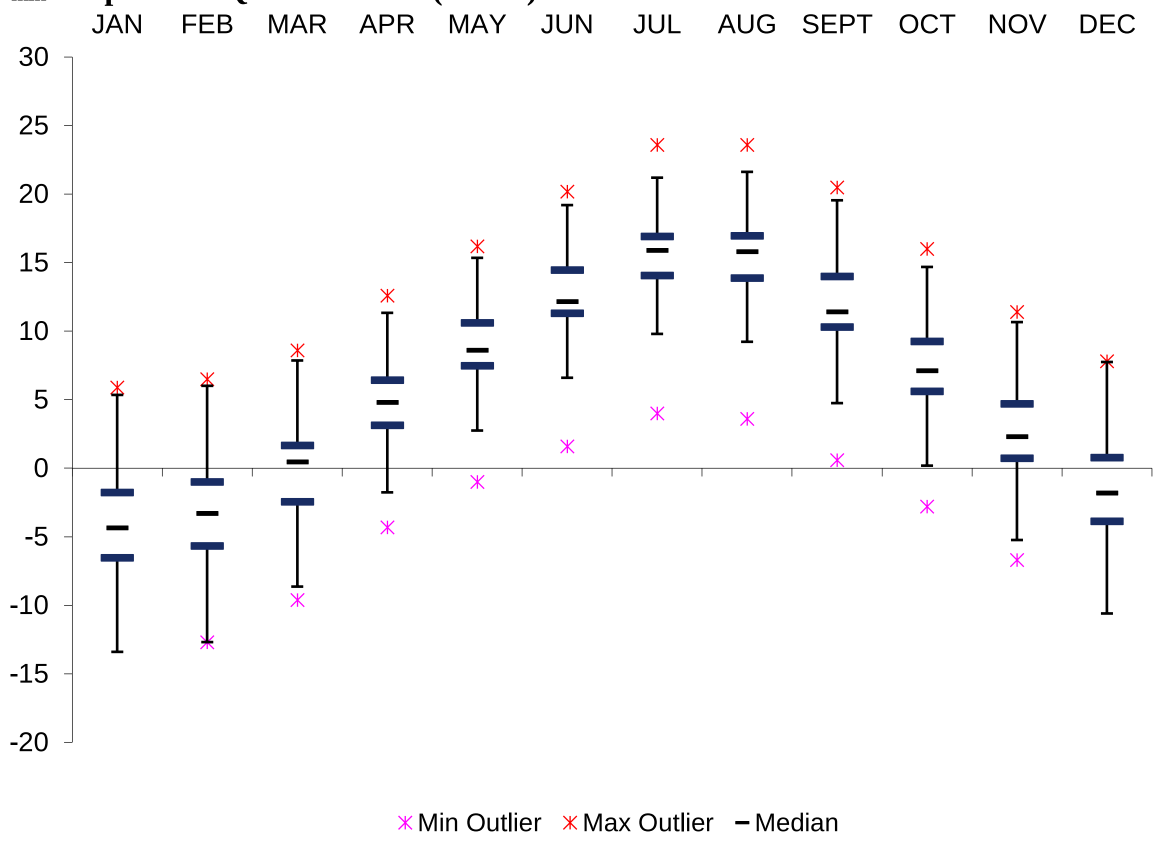

**KÖPPEN PROFILES – *Quercus trojana* Webb, 1839**

GBIF localities of *Q. trojana* (s.str., i.e. excluding Euboean populations, → *Q. euboica*; preserved specimen)

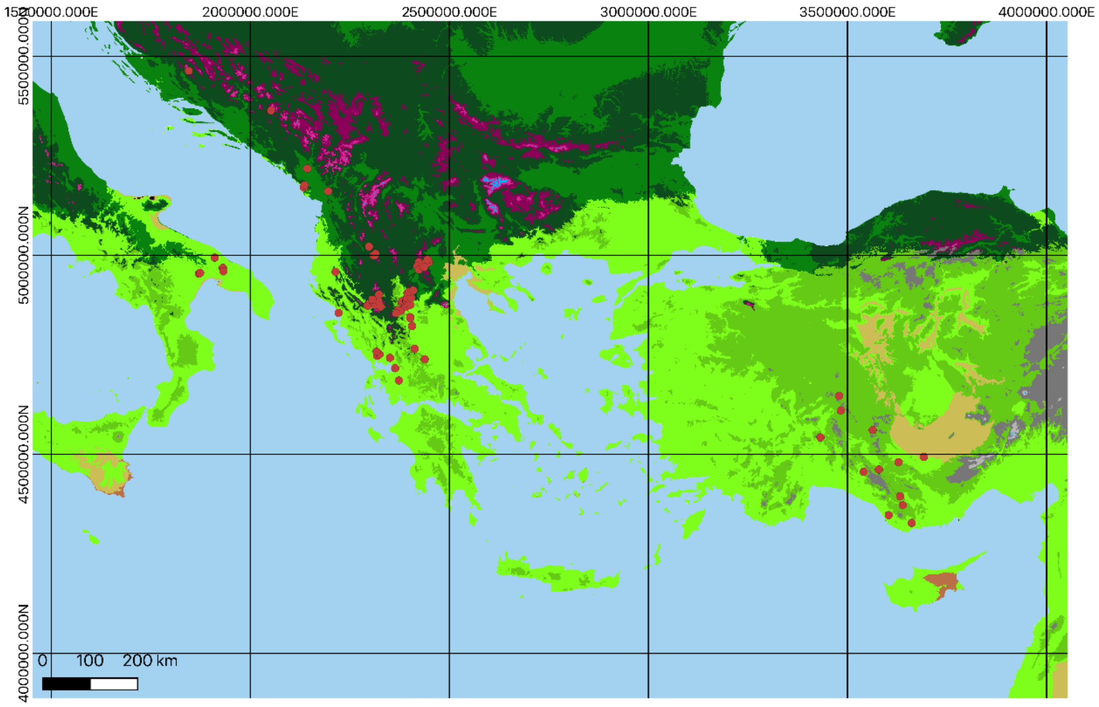


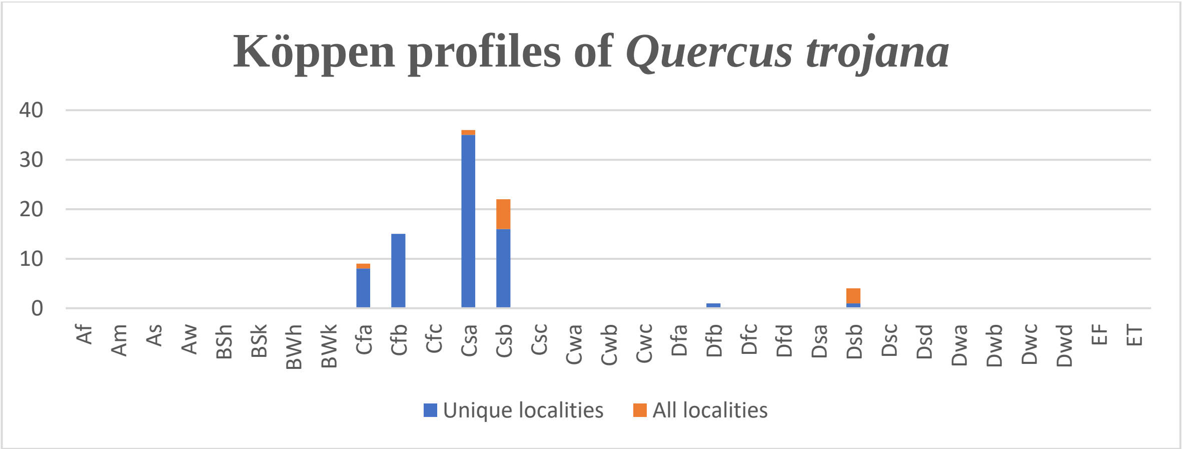


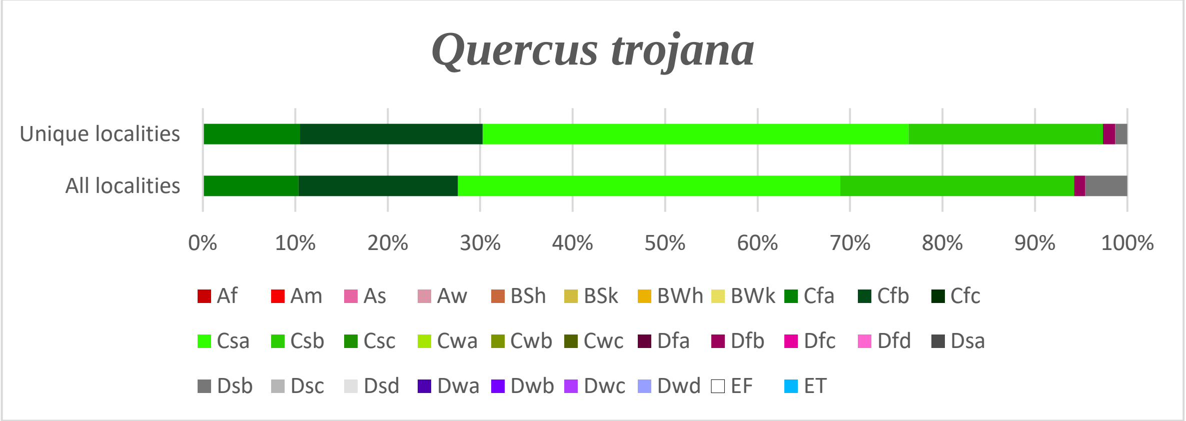


All localities (n = 87); unique localities (n = 76)

**BIOMES – *Quercus trojana***
GBIF localities of *Q. trojana* (preserved specimen)

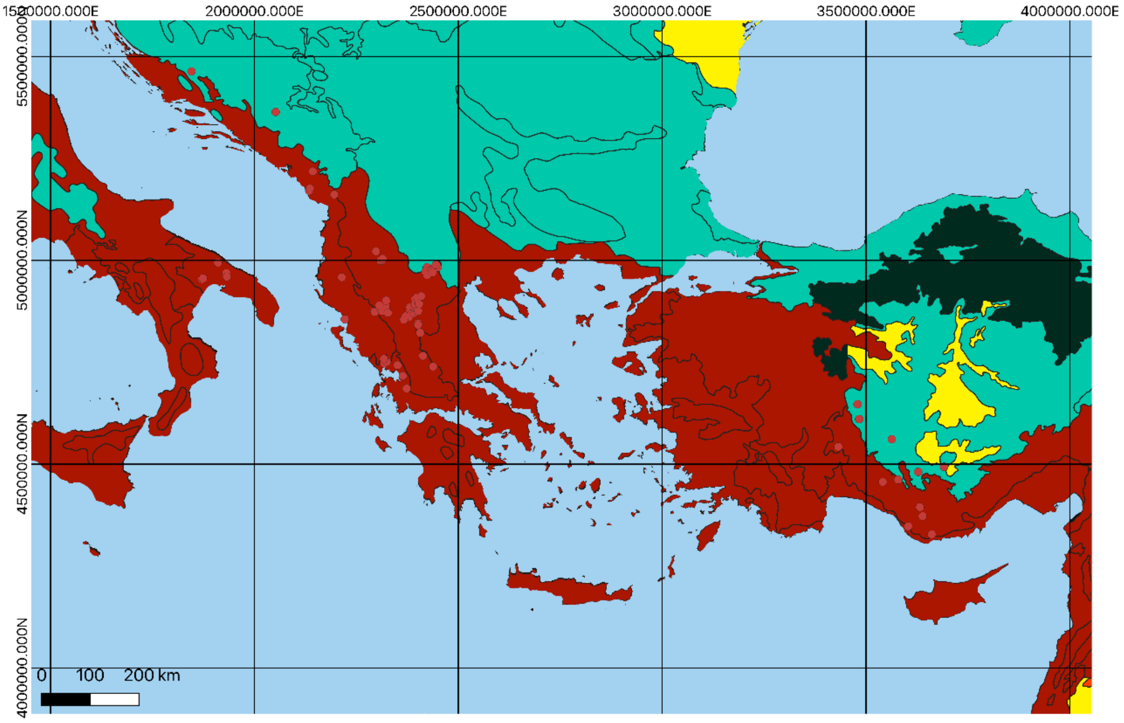


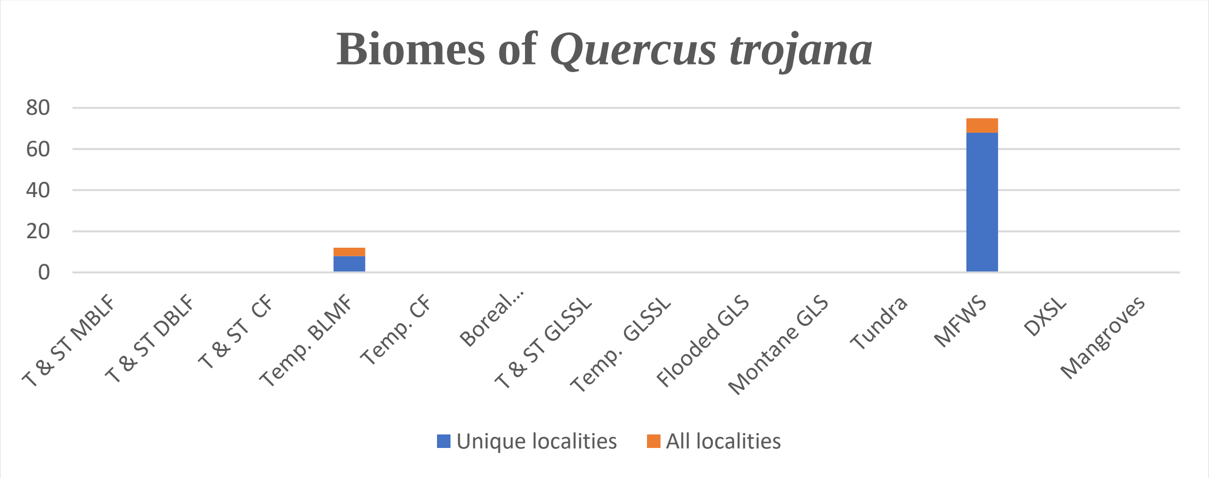


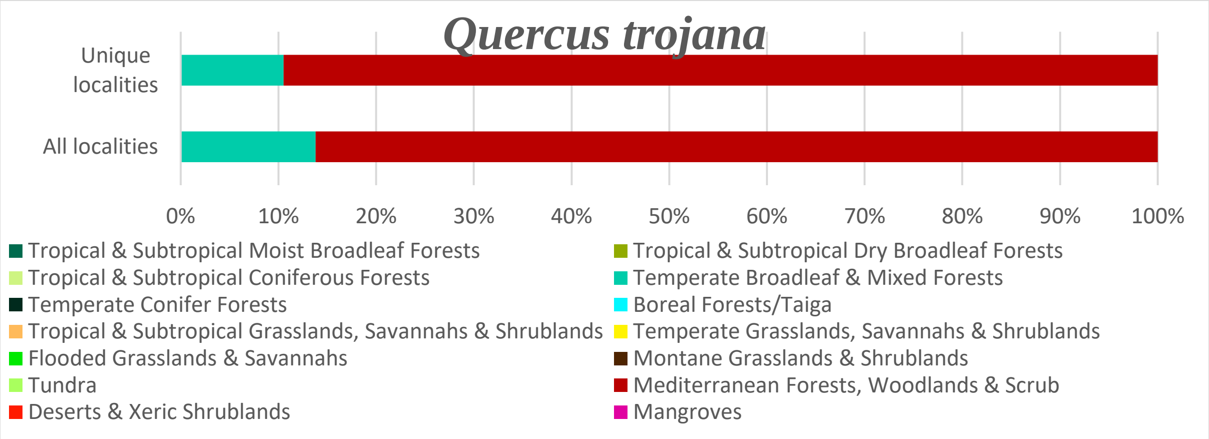


All localities (n = 87); unique localities (n = 76)

**MTCM (x-axis) vs PCQ (y-axis) for *Quercus trojana* (n = 76).** Regarding its bioclimatic niche, *Q. trojana* bridges between *Q. libani* and subsect. *Cerris*, showing a higher preference for mild winters accumulating most of a year's precipitation (fully Mediterranean *Csa* climates; → MMP boxplot). Like other members of the *Cerris* core clade, it avoids habitats with extreme summer draught (MMP ~ 0 mm).

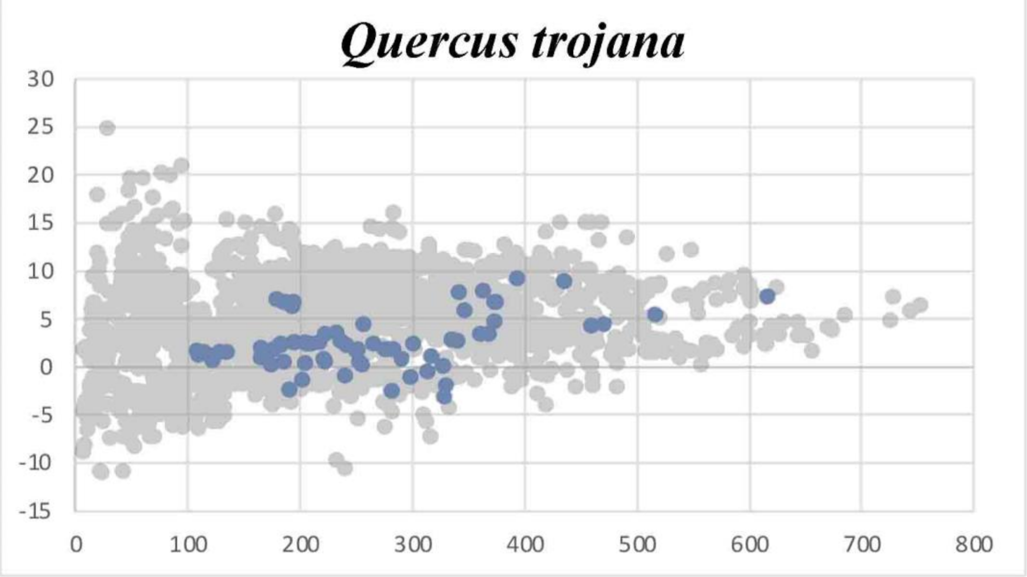


**MMP boxplots for *Quercus trojana* (n = 76)**

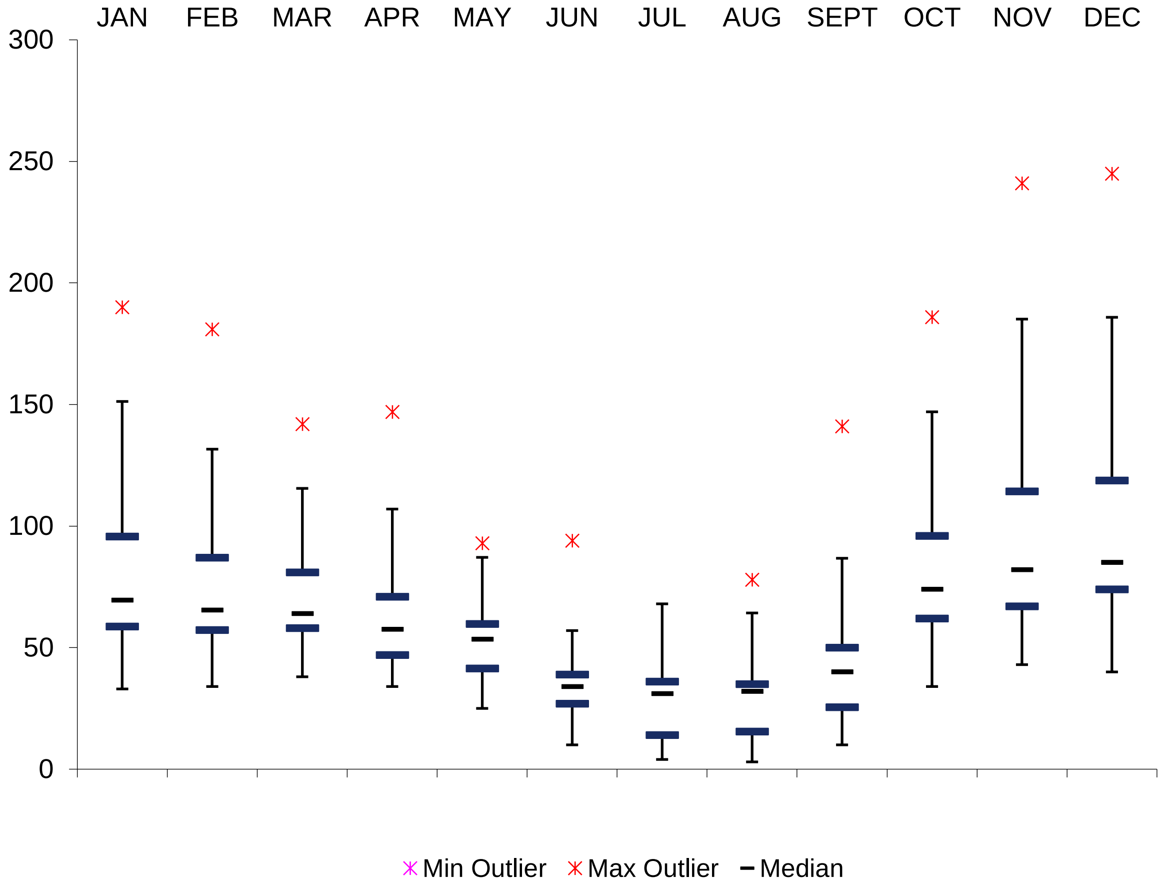

**MMT boxplots for *Quercus trojana* (n = 76)**

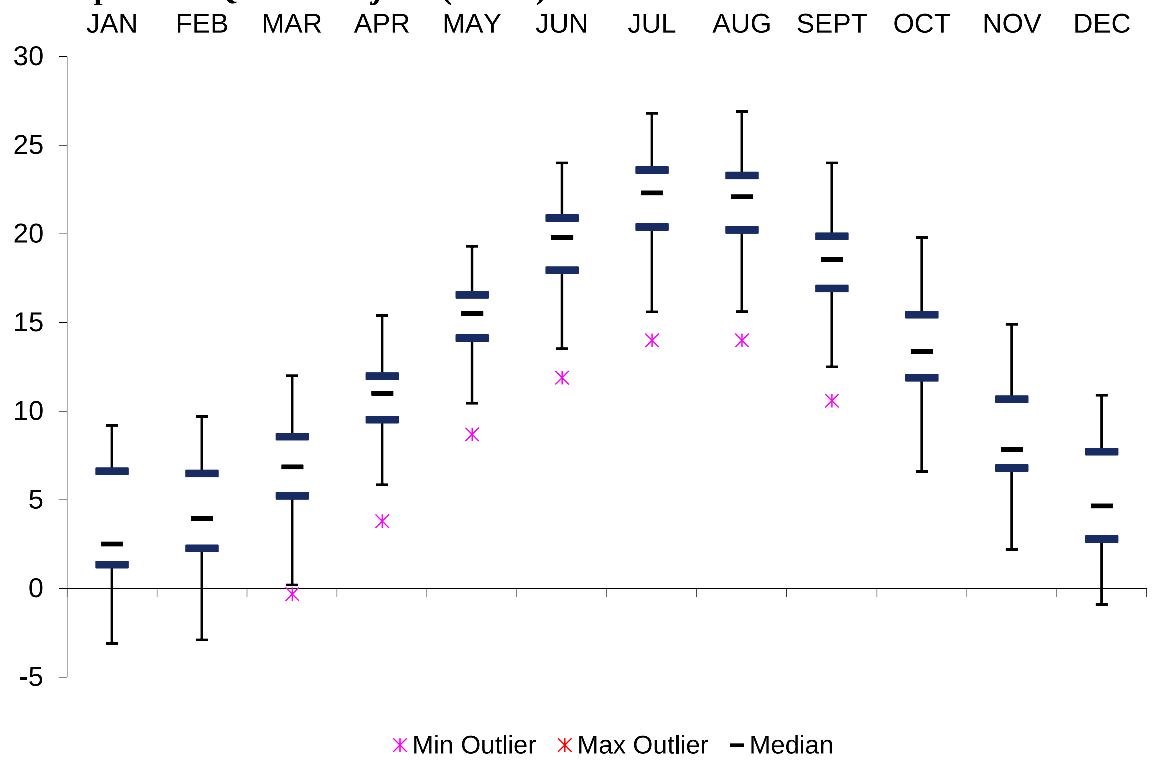


**MT$_{min}$ boxplots for *Quercus trojana* (n = 76)**

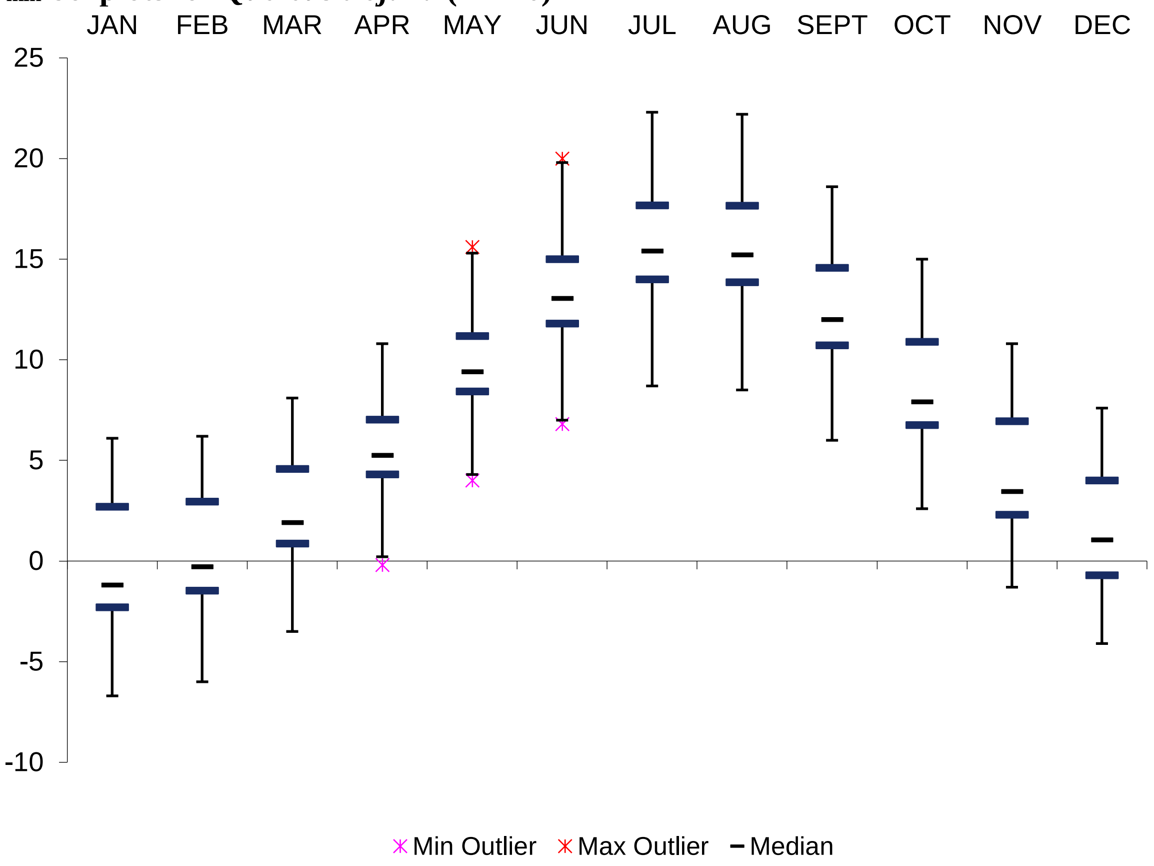

## Quercus *sect.* Cerris *subsect.* Aegilops

Cumulative niche of *Q. brantii*, *Q. ithaburensis* (incl. *Q. macrolepis* = *Q. ithaburensis* subsp**.** *macrolepis*)

**KÖPPEN MAP** (n = 214)

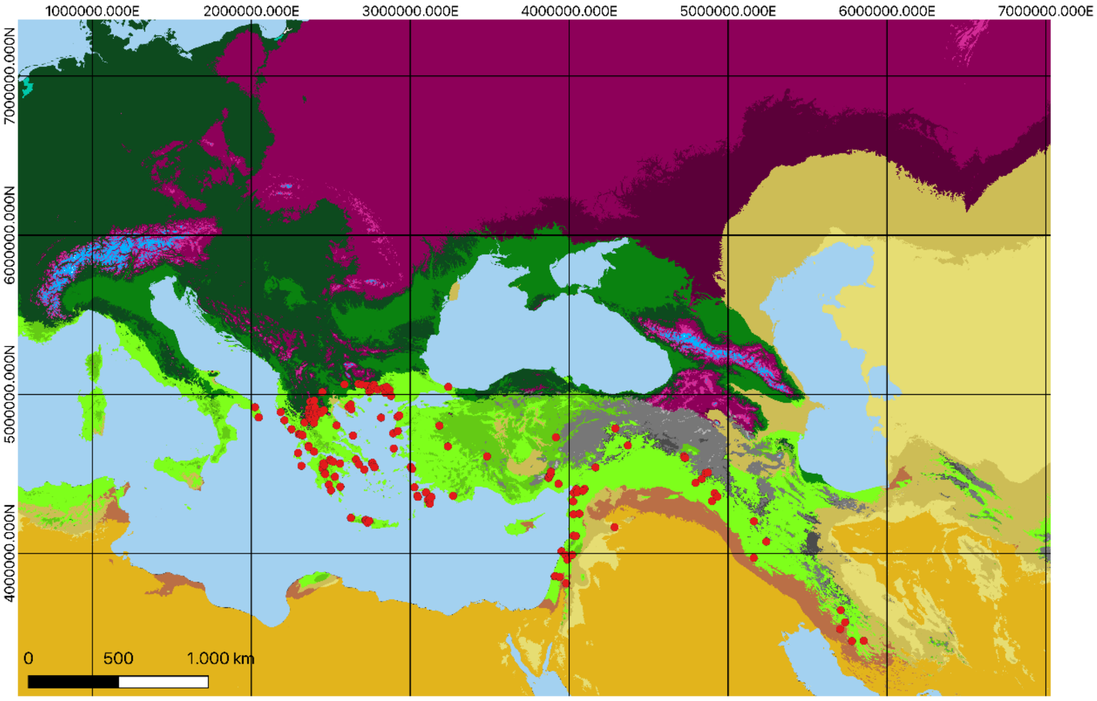


**MTCM (x-axis) vs PCQ (y-axis) for members subsect. Aegilops (n = 175).** This subsection occupies the distinctly Mediterranean niches in the Eastern Mediterranean, paralleling *Q. suber* in the Western Mediterranean with PCQ > 100 mm, and (very) mild winters (MTCM > 0 °C). The exception is *Q. brantii*, tolerating harsher continental climates charaterizing the Levant and Zagros Mts.

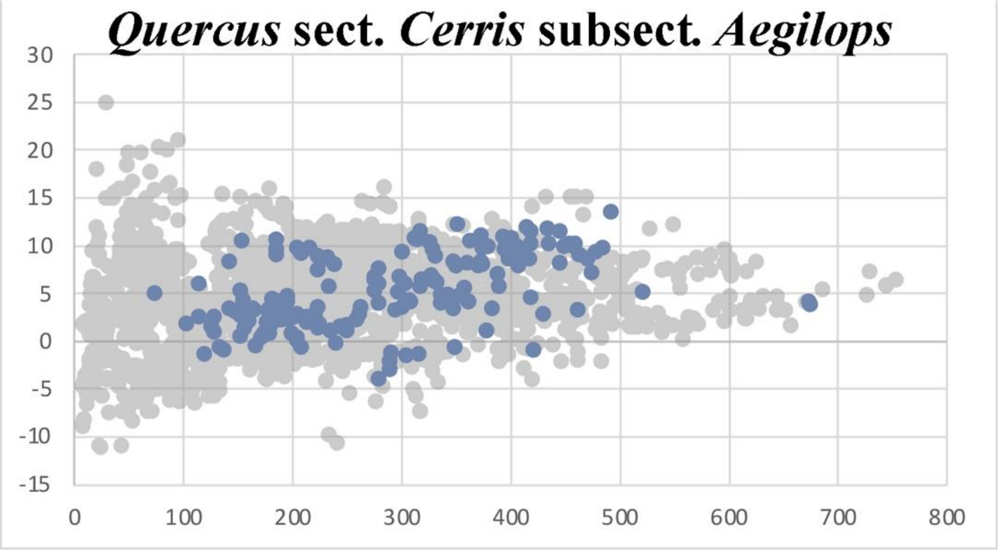

**KÖPPEN PROFILES – *Quercus brantii* Lindl., 1840**

GBIF localities of *Q. brantii* (preserved specimen)

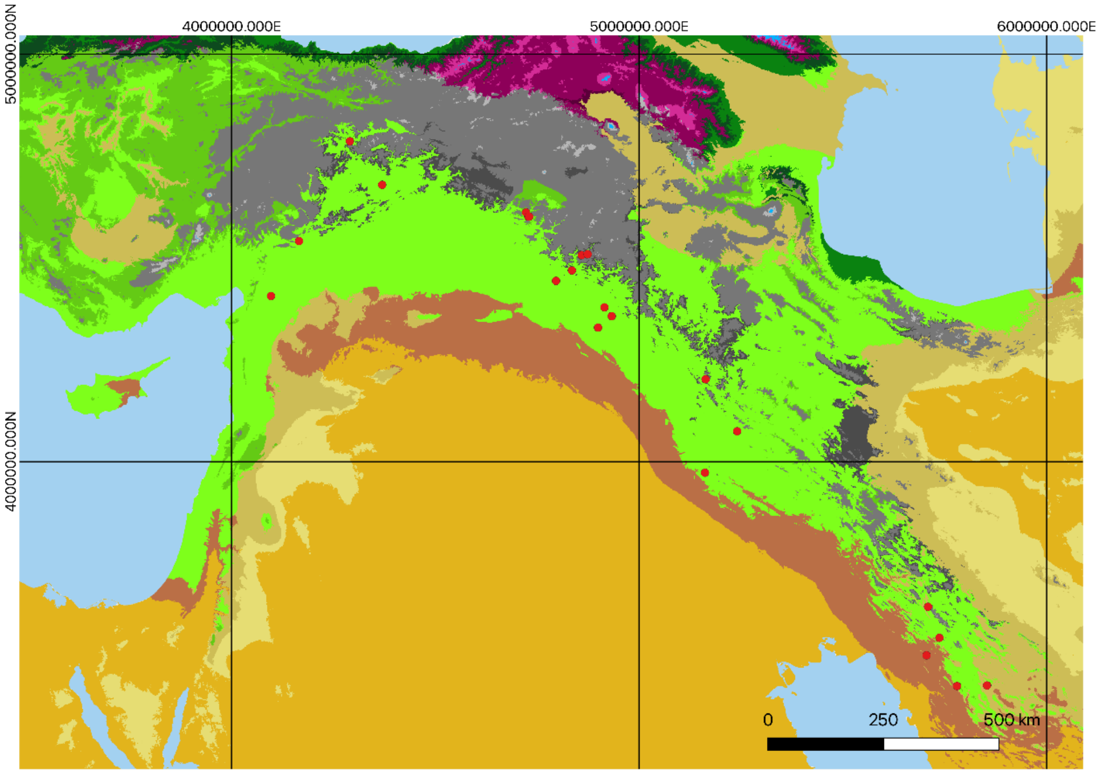


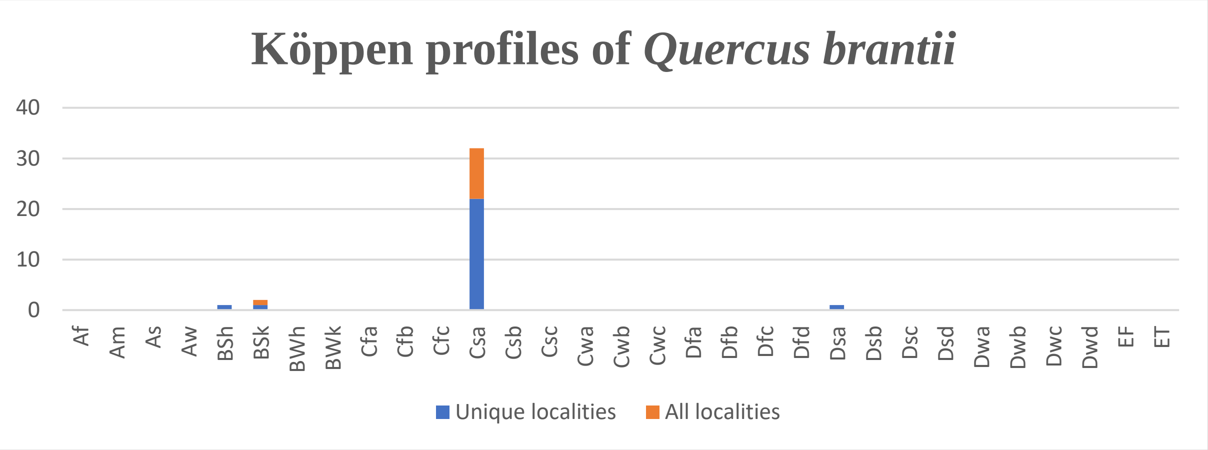


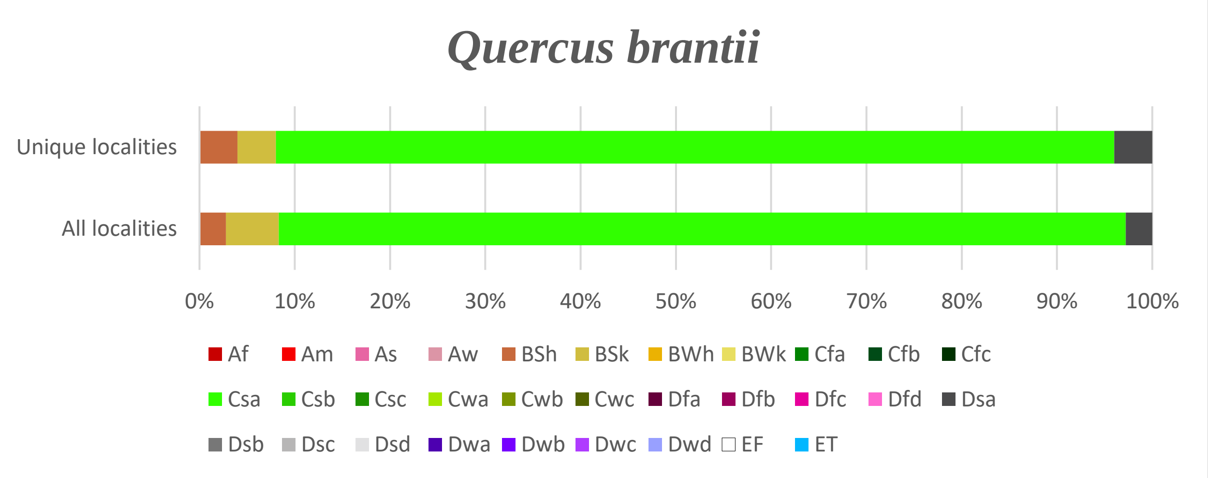


All localities (n = 36); unique localities (n = 22)

**BIOMES – *Quercus brantii***

GBIF localities of *Q. brantii* (preserved specimen)

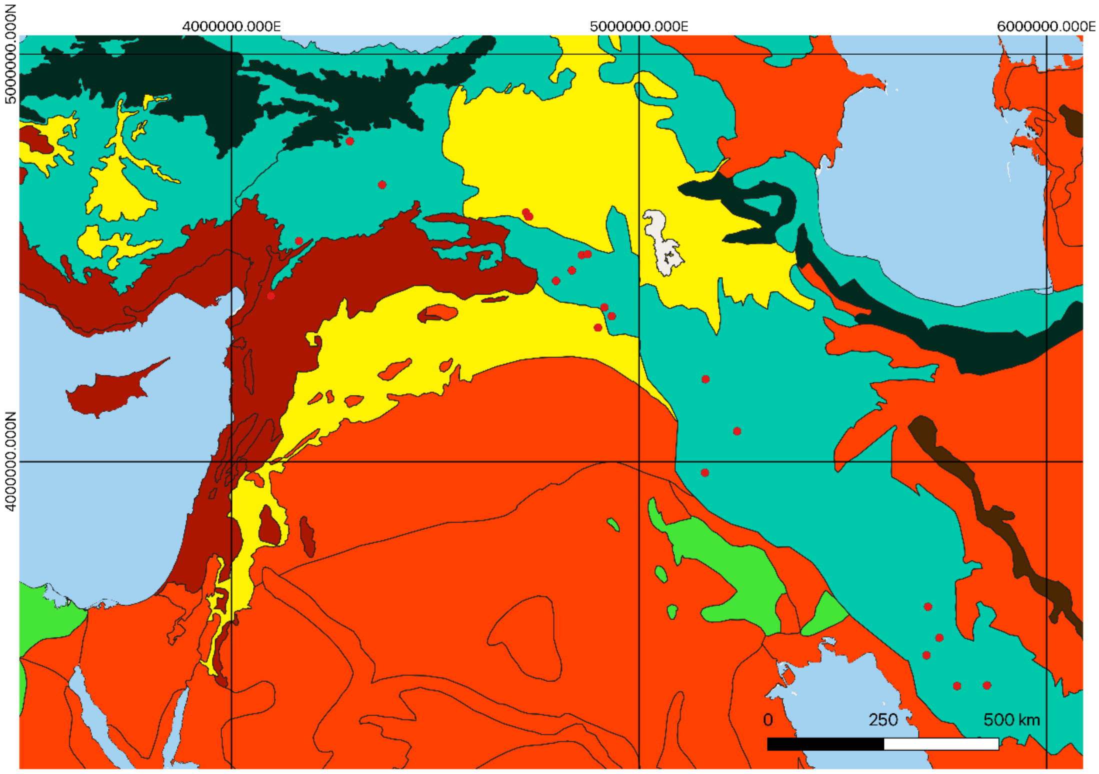


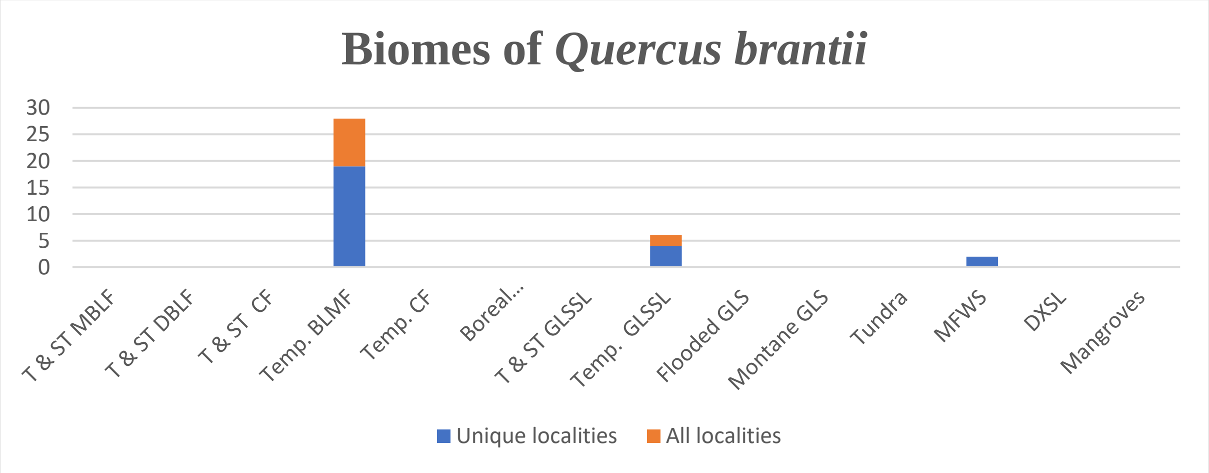


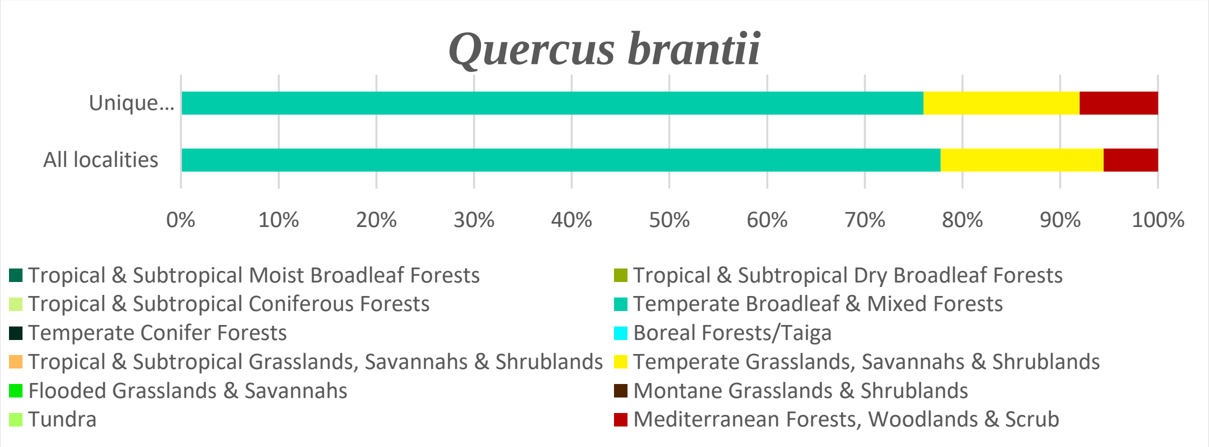


All localities (n = 36); unique localities (n = 25)

**MTCM (x-axis) vs PCQ (y-axis) for *Quercus brantii* (n = 23)**. In contrast to its Mediterranean sisters, *Q. brantii* can tolerate distinctly continental settings with moist (snowy) and ± cold winters (MTCM < 0 °C; PCQ ≈ 300 mm; → MTCM/$MT_{min}$ boxplots). Like its sisters, it endures long and profound summer draughts (MMP ~ 0 mm from June to September), hence, represents a fully Mediterranean ('semihumid-meridional' according Grímsson et al., 2016, fig. 2), species. It is not as hardy as *Q. libani*, the subsect. *Libani* species replacing *Q. brantii* to the west and north in Alpine mountain chains of (south-)eastern Anatolia.

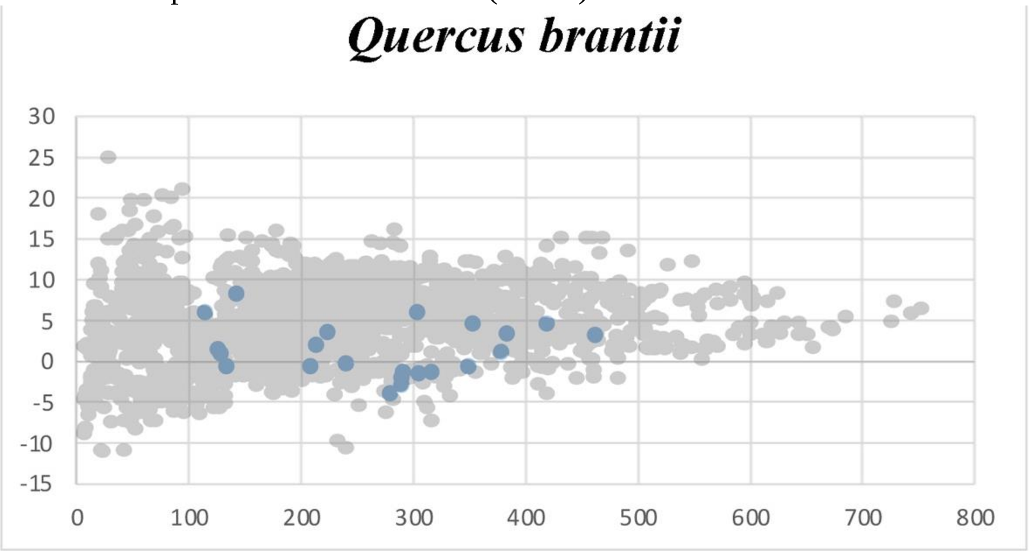


**MMP boxplots for *Quercus brantii*, (n = 23)**

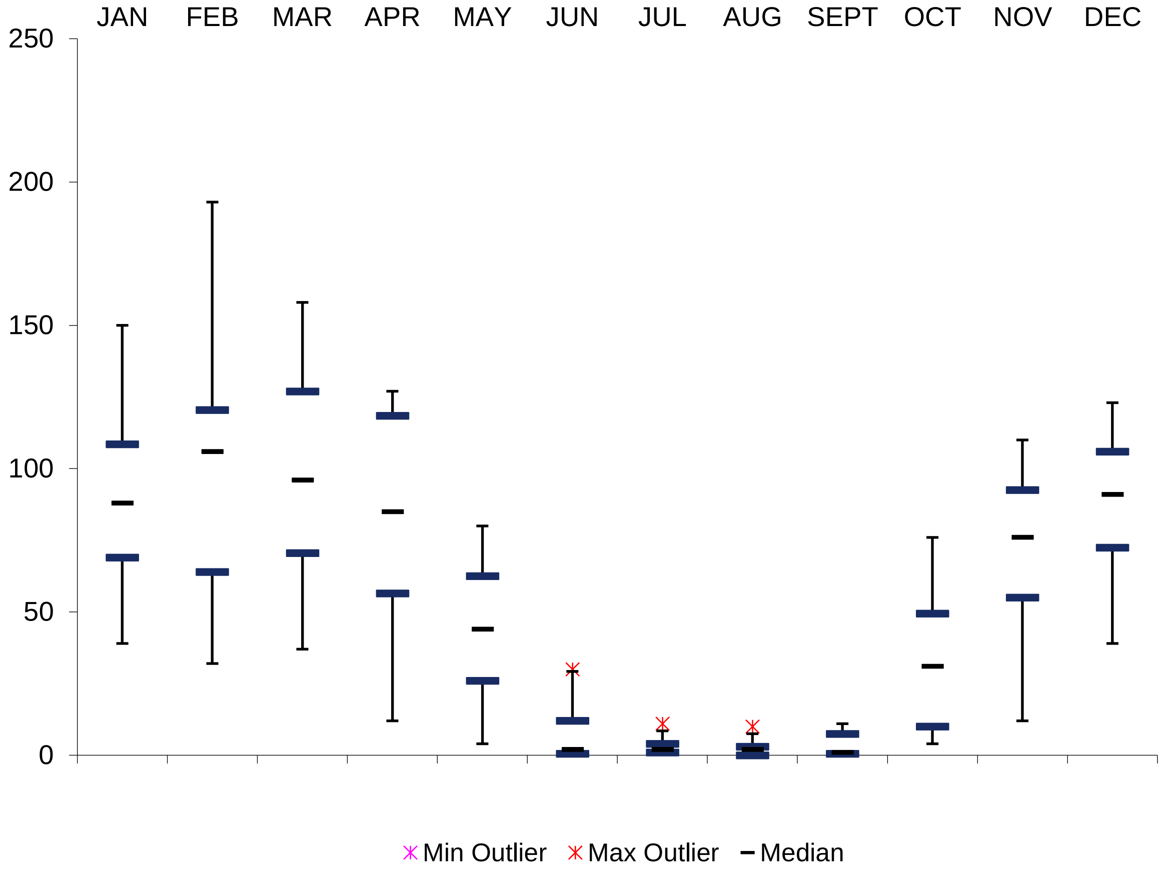

**MMT boxplots for *Quercus brantii* (n = 23)**

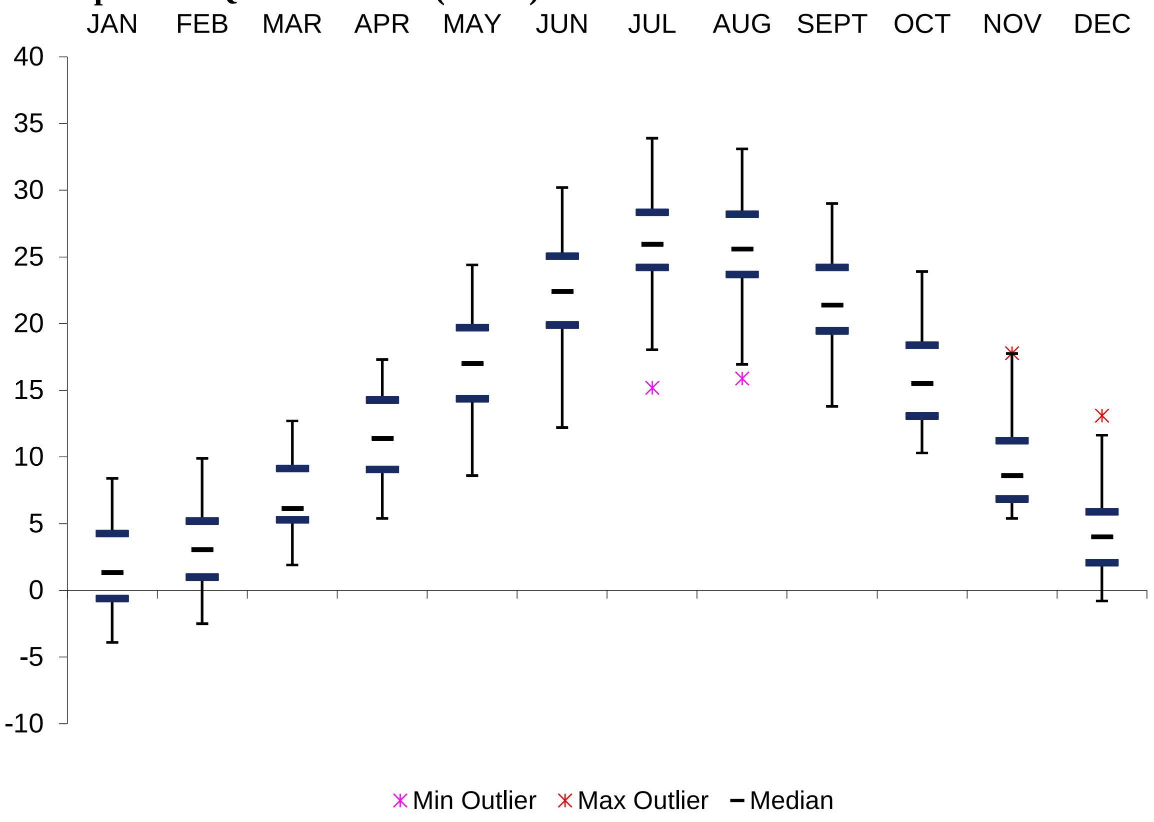


**MT$_{min}$ boxplots for *Quercus brantii* (n = 23)**

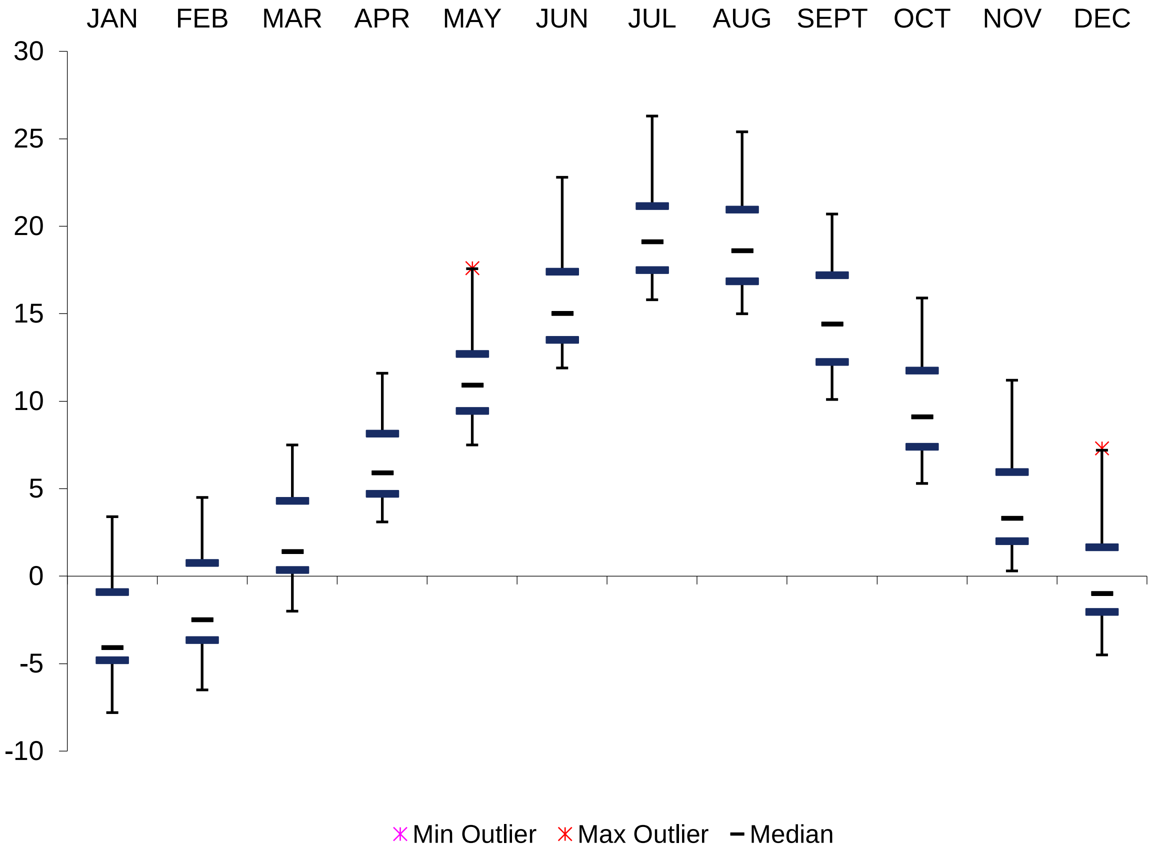

**KÖPPEN PROFILES – *Quercus ithaburensis* s.l. Decne., 1835, incl. *Q. macrolepis* (= *Q. ithaburensis* subsp. *macrolepis*)**

GBIF localities of *Q. ithaburensis* s.l. (preserved specimen)

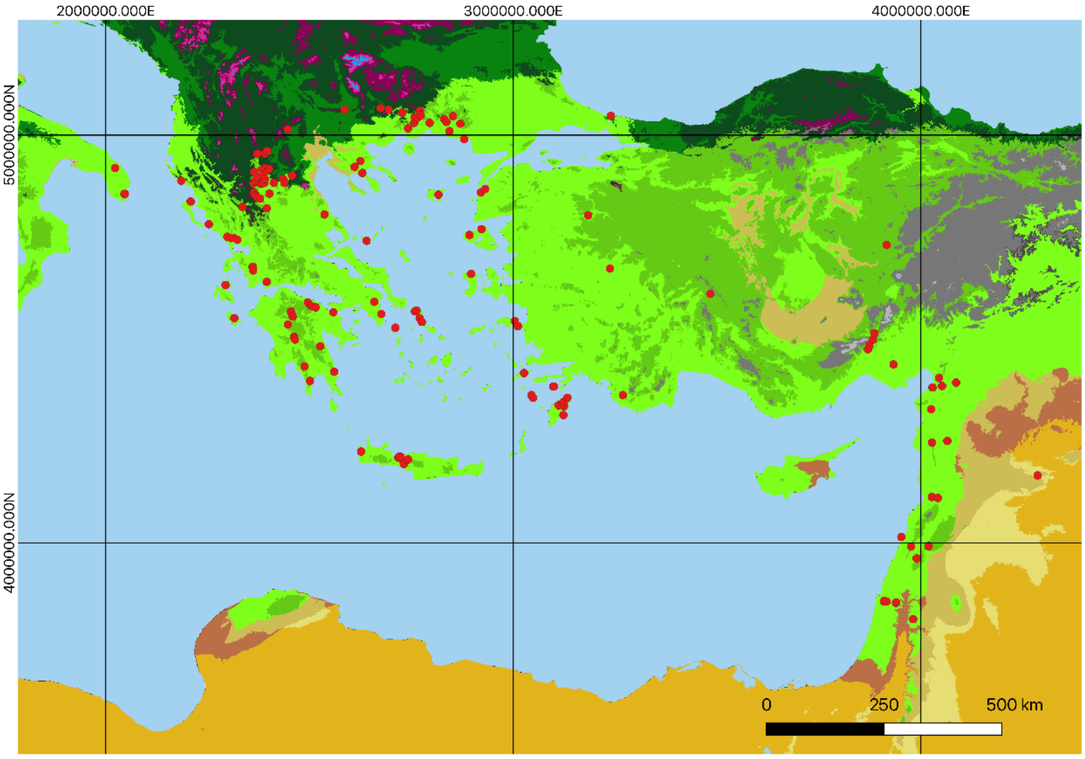


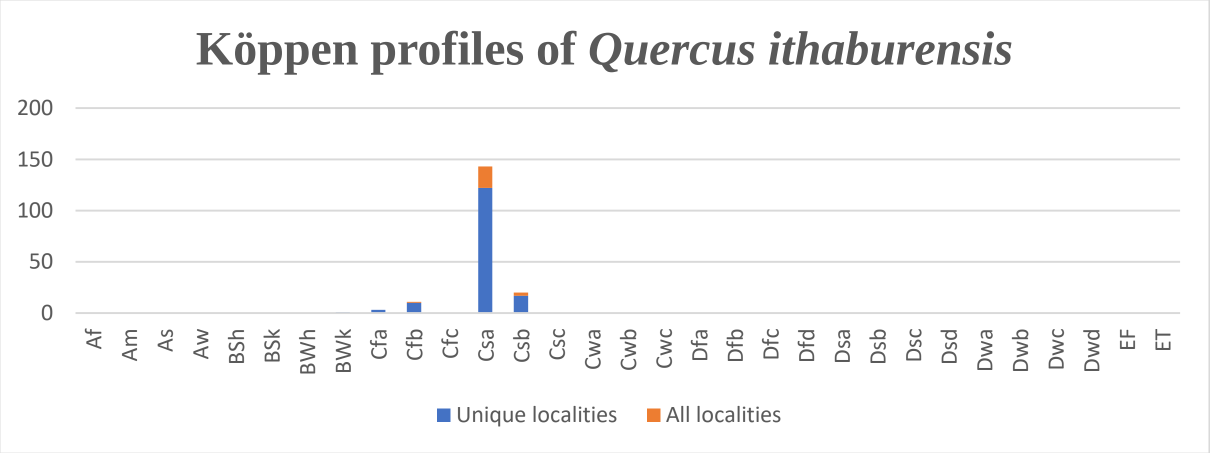


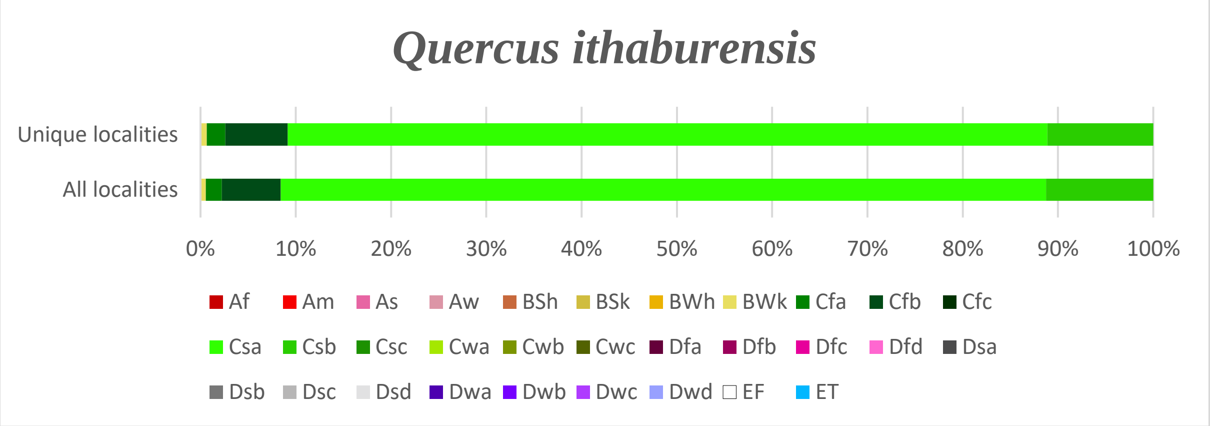


All localities (n = 178); unique grid cells (n = 153)

**BIOMES – *Quercus ithaburensis* s.l.**
GBIF localities of *Q. ithaburensis* s.l. (preserved specimen)

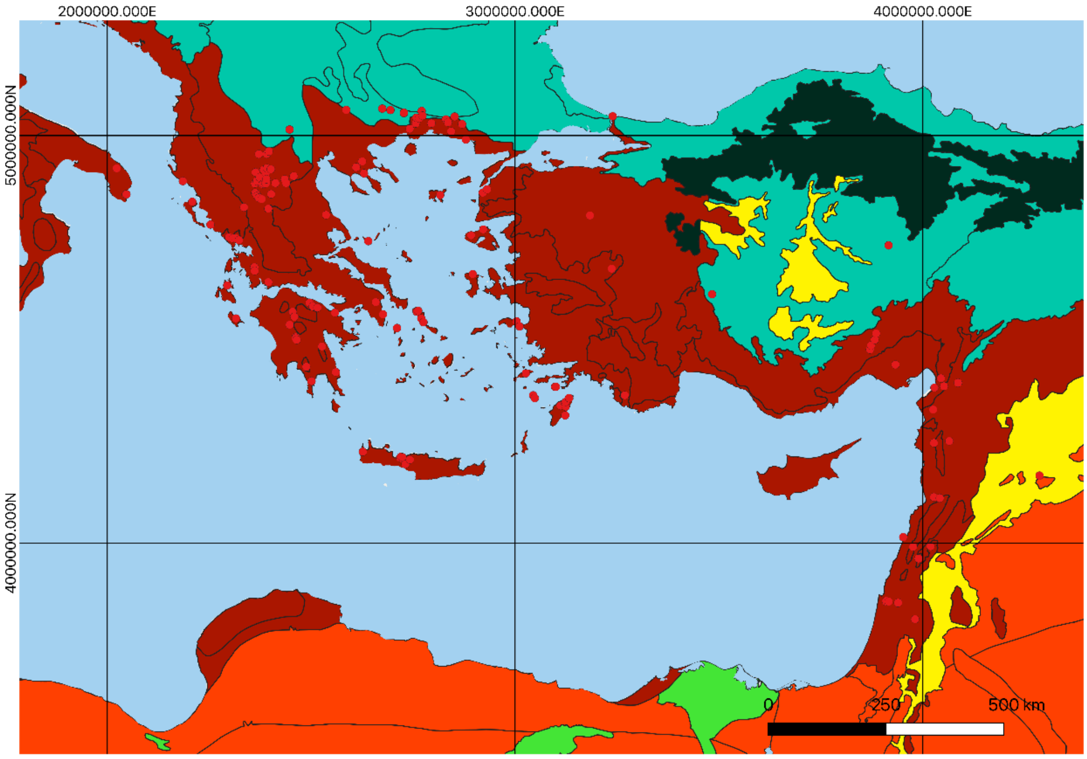


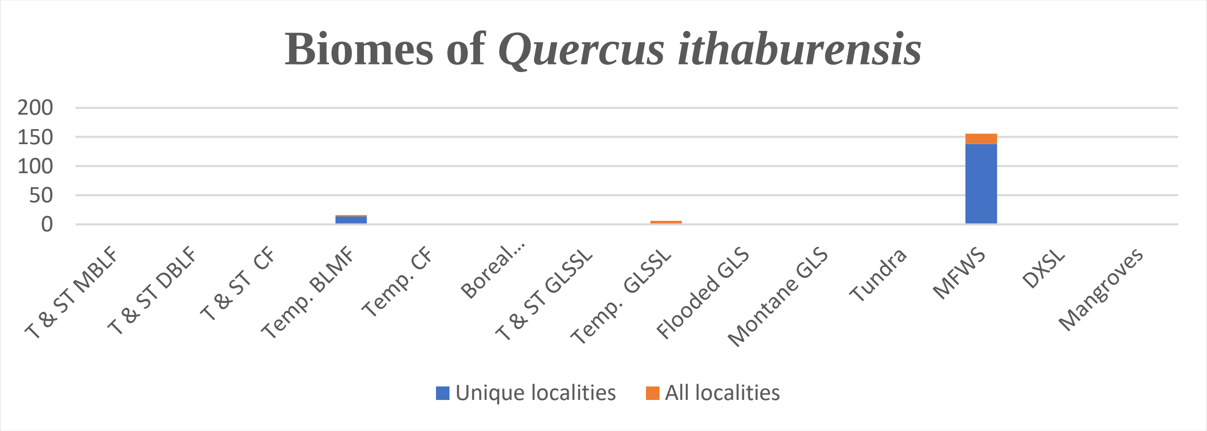


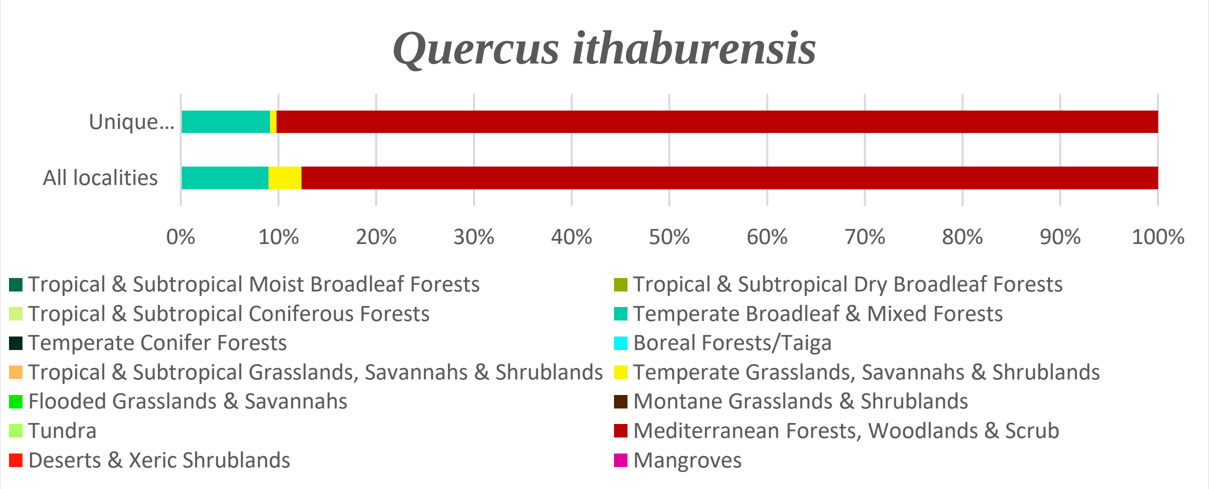


All localities, n = 178; unique localities, n = 153

**MTCM (x-axis) vs PCQ (y-axis) for *Quercus ithaburensis* s.l. (incl. *Q. macrolepis*; n = 153)**. The sister species *Q. ithaburensis* and *Q. macrolepis* (not differentiated in GBIF) are fully Mediterranean, lowland species that cannot thrive in montane/ continental settings being intolerant to cold (MTCM < 0 °C) winters with snow (*D*-climates) or dry winters (PCQ < 100 mm). They need habitats where most of the year's precipitation is concentrated in the winter quartal/half (→ MMP boxplot).

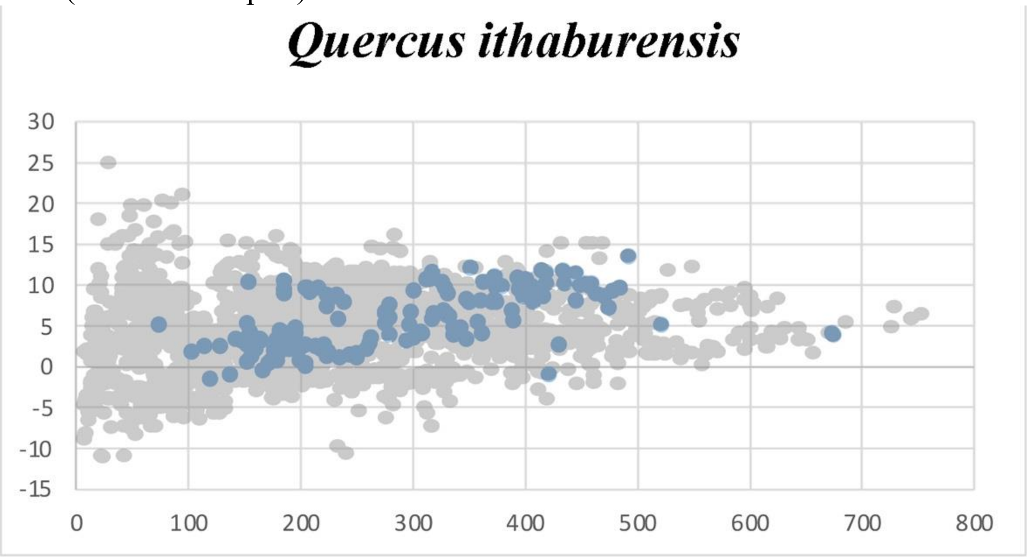


**MMP boxplots for *Quercus ithaburensis* (n = 153)**

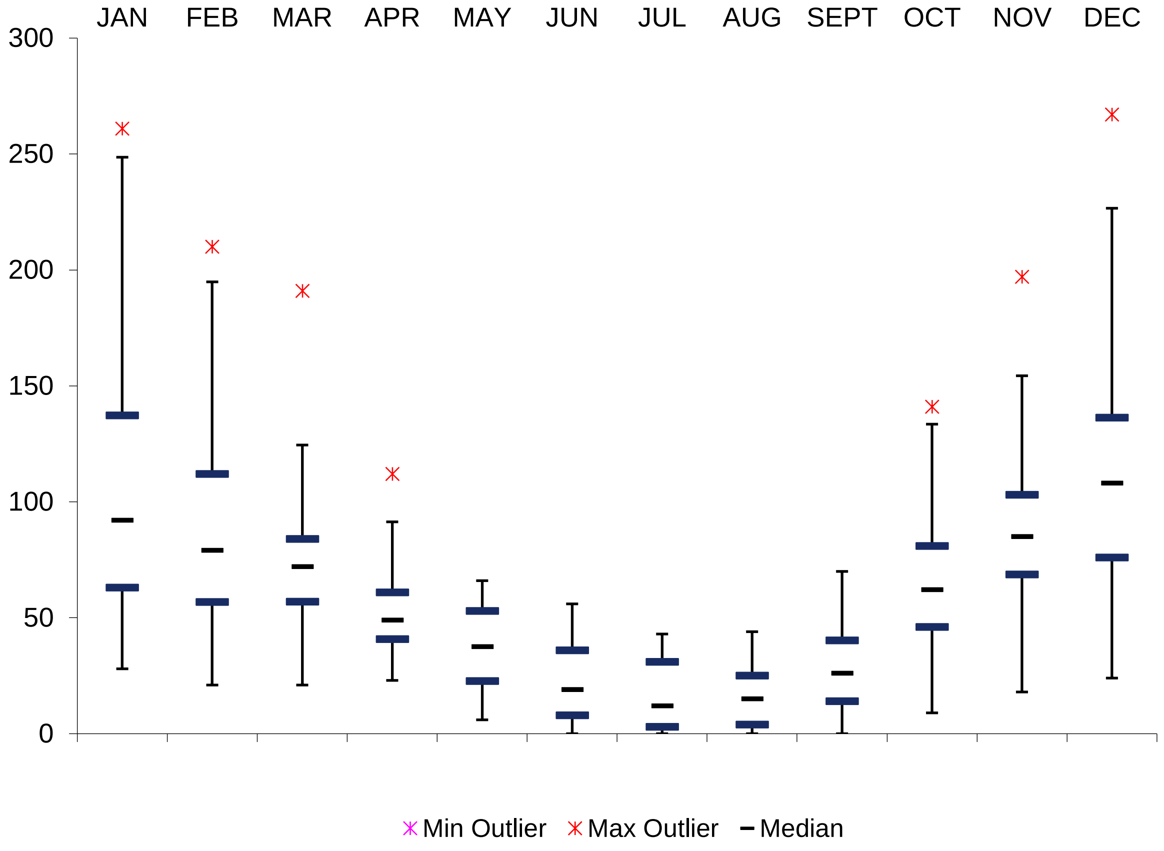

**MMT boxplots for *Quercus ithaburensis* (n = 153)**

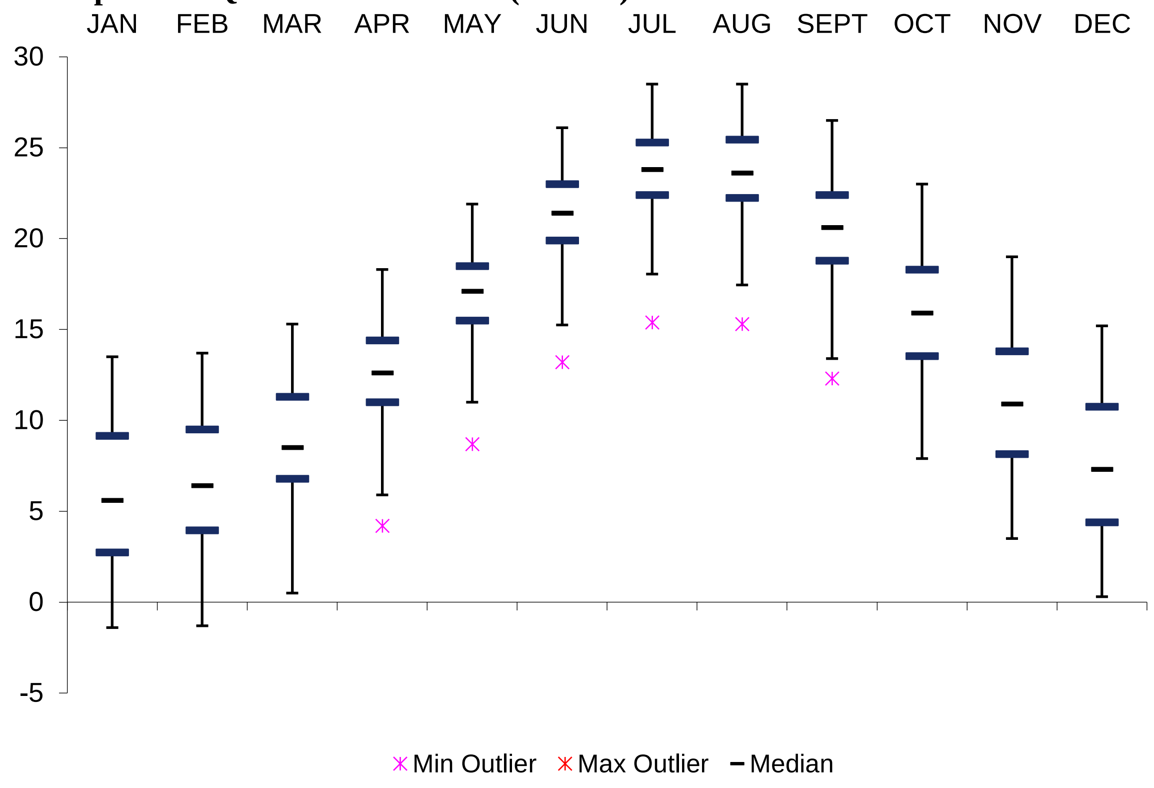


**$MT_{min}$ boxplots for *Quercus ithaburensis* (n = 153)**

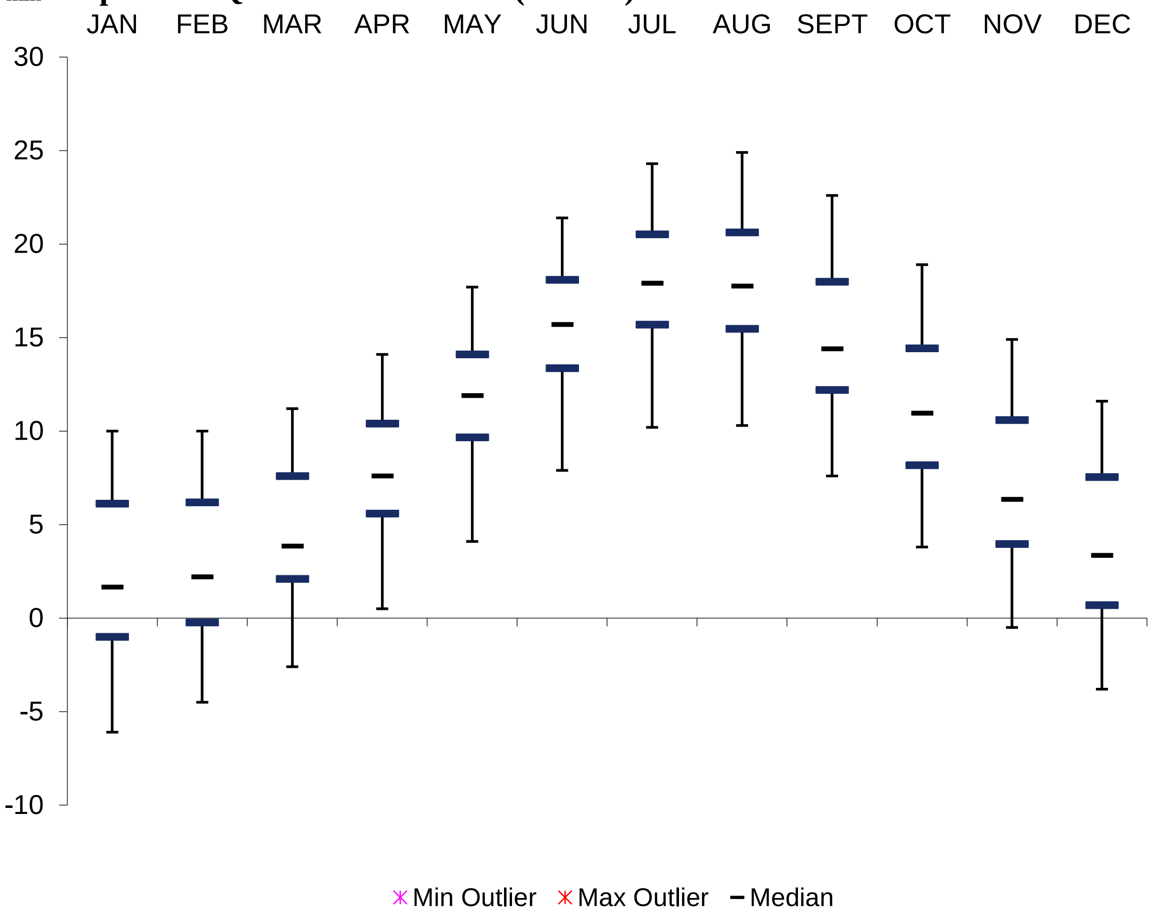

## Quercus *sect.* Cerris *subsect.* Suber

Cumulative niche of *Q. crenata* and *Q. suber*

**KÖPPEN MAP** (n = 1193)

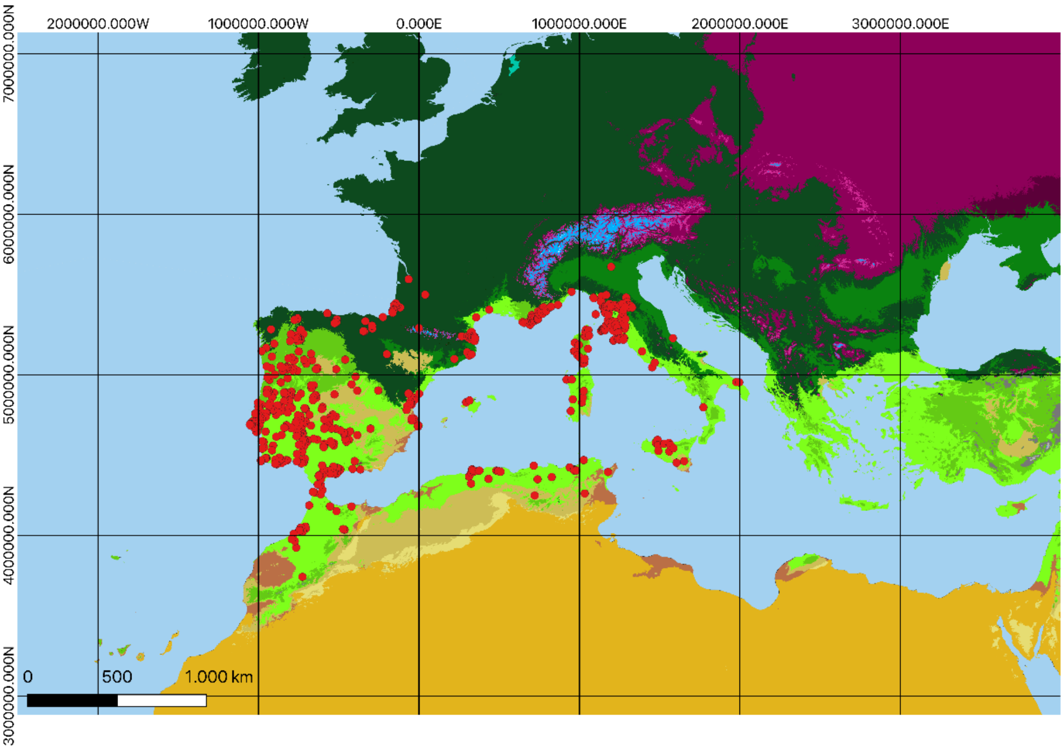


**MTCM (x-axis) vs PCQ (y-axis) for members subsect. *Suber* (n = 713).** This subsection represents the climatic and ecological antithesis to subsect. *Cerris*, well-illustrated in their respective winter preferences. While subsect. *Cerris* prefers habitats with cool/cold winters (MTCM < 5 °C), subsect. *Suber* prefers habitats with (very) mild winters (MTCM > 5 °C). Like subsect. *Cerris*, subsect. *Suber* covers a wide winter precipitation regime range. The two species of this subsection show a striking differentiation in their climatic preferences.

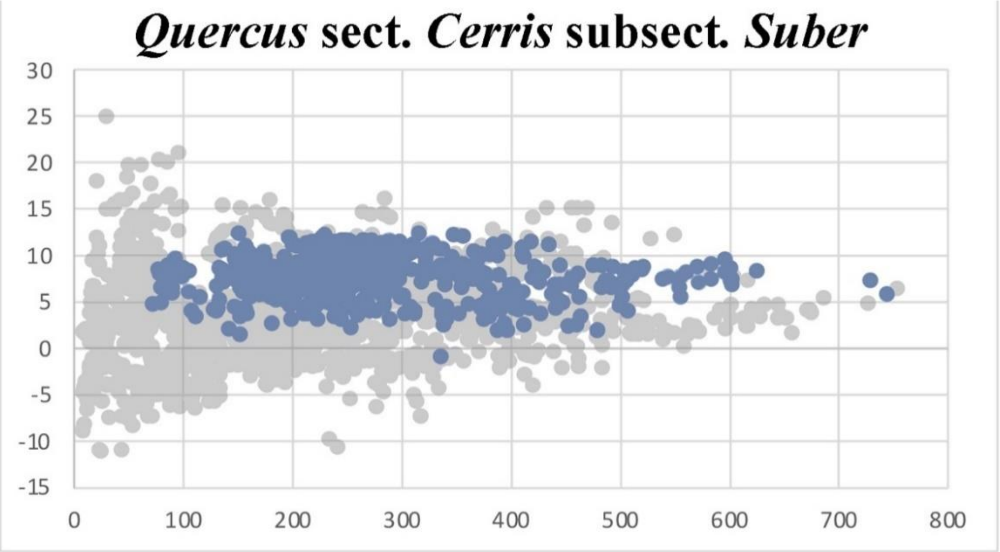

**KÖPPEN PROFILES – *Quercus crenata* Lam., 1785**
GBIF localities of *Q. crenata* (preserved specimen)

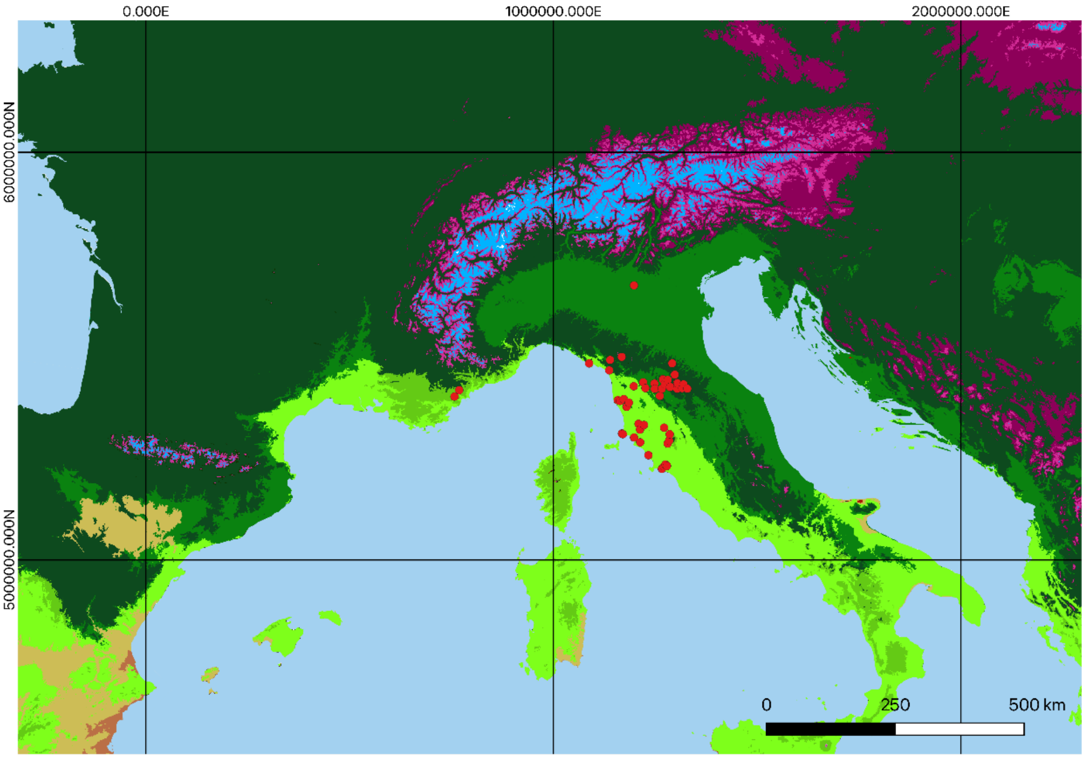


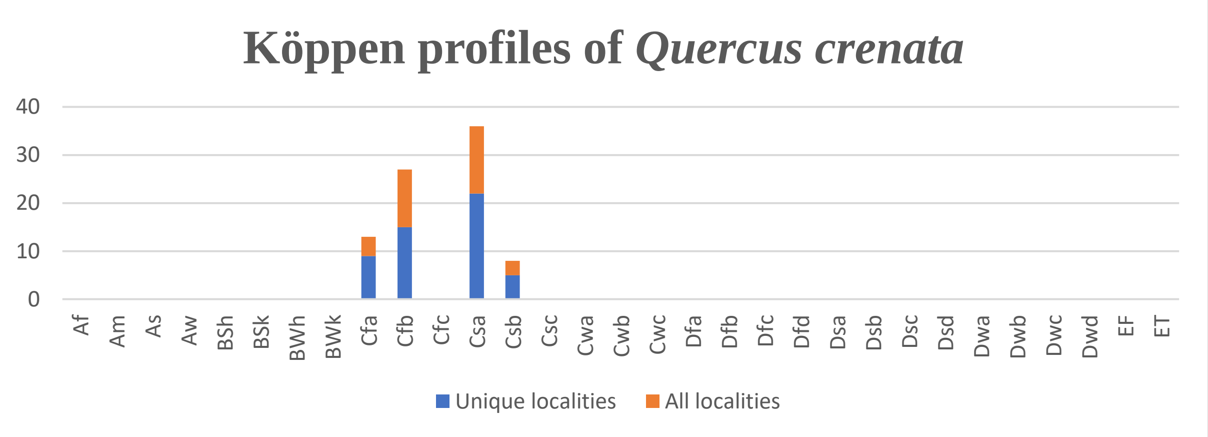


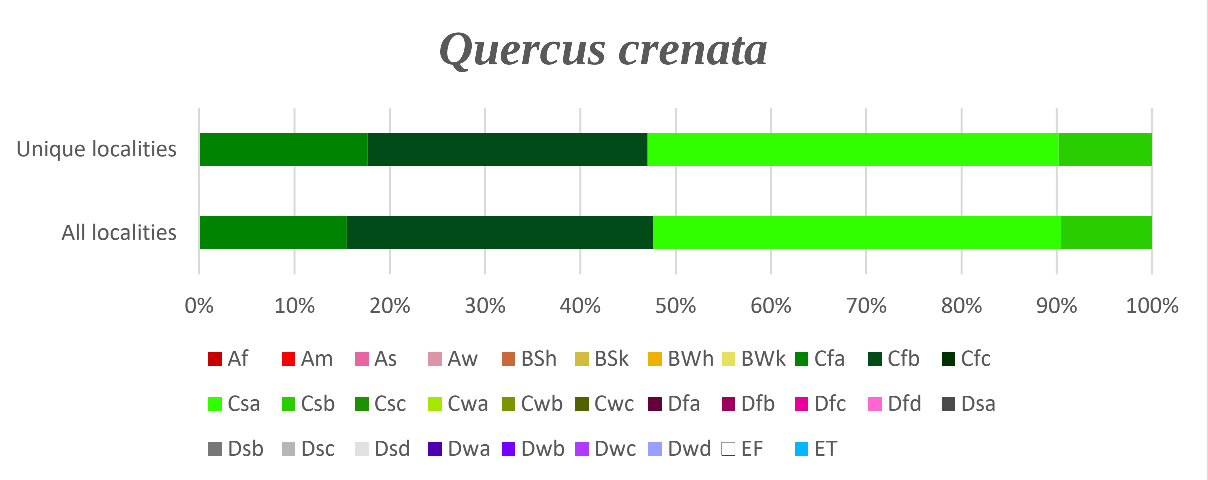


All localities (n = 84); unique grid cells (n = 52)

**BIOMES – *Quercus crenata***
GBIF localities of *Q. crenata* (preserved specimen)

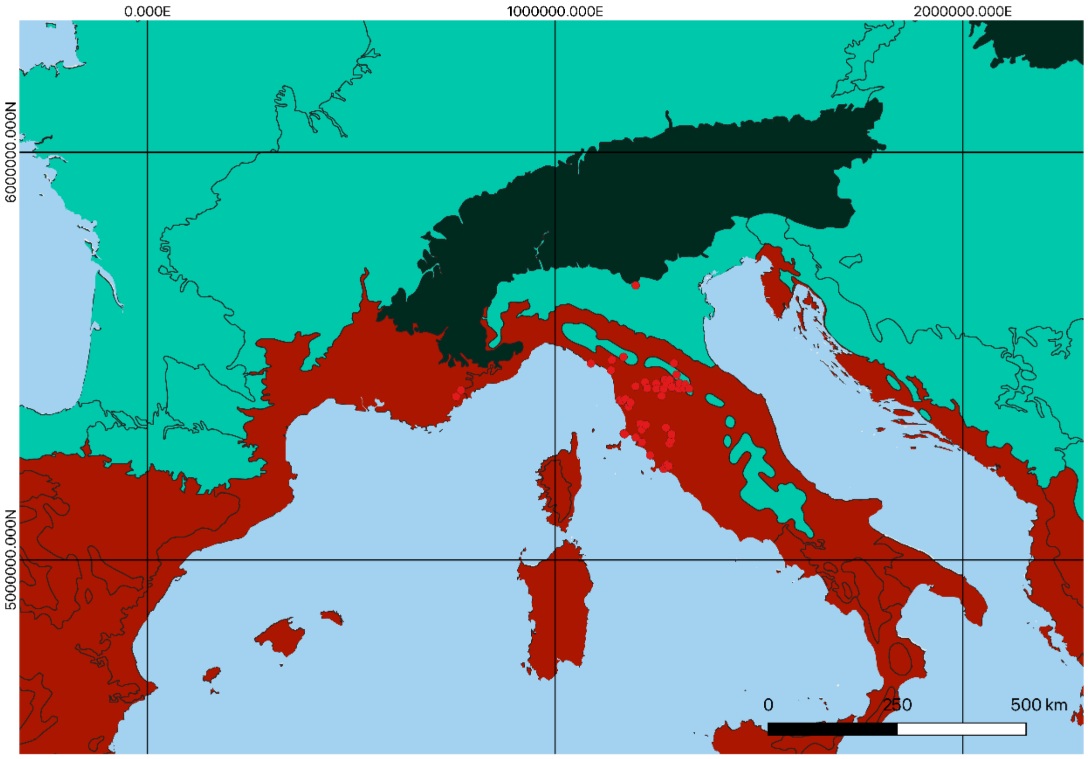


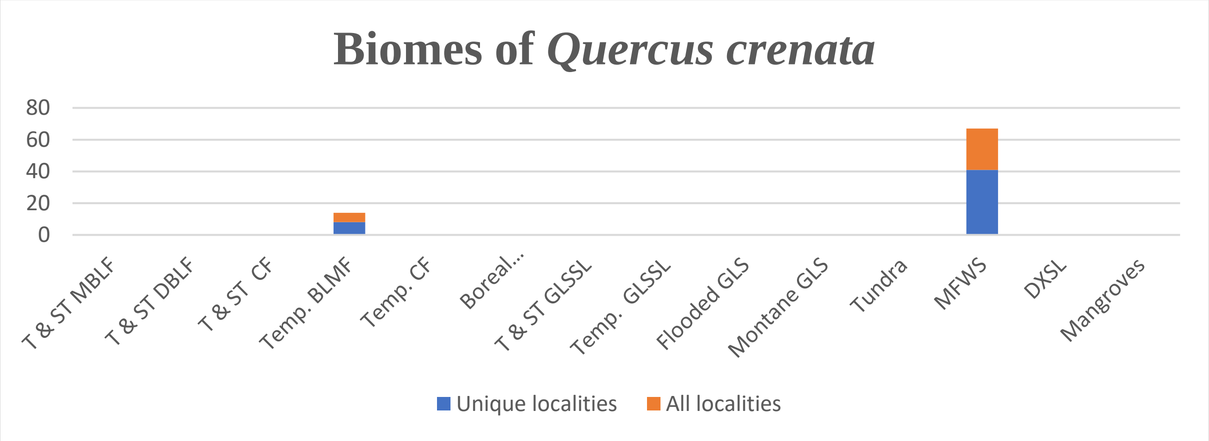


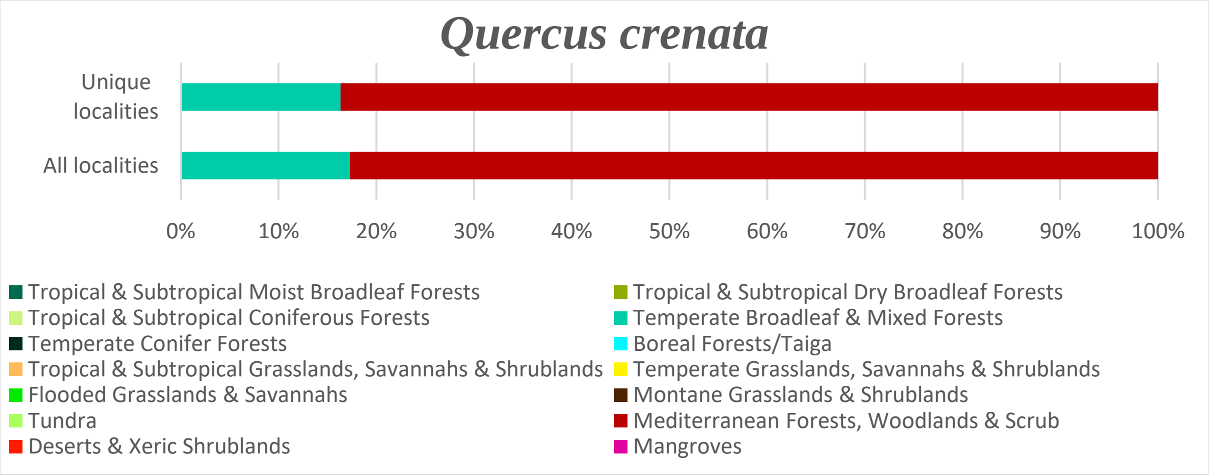


All localities (n = 81); unique localities (n = 50)

**MTCM (x-axis) vs PCQ (y-axis) for *Quercus crenata* (n = 50).** Restricted to the northern and eastern side of the Ligurian Sea, *Q. crenata* (a '[meridio-]nemoral' species according Denk et al., 2013) marks the bioclimatic and geographic contact zone between its sister *Q. suber* and *Q. cerris*, the most widespread and only 'nemoral' cork oak species in Western Eurasia. Like the Iranian relict *Q. castaneifolia*, *Q. crenata* thrives in monsoon-like situations marked by an autumn precipitation maximum (→ MMP boxplot).

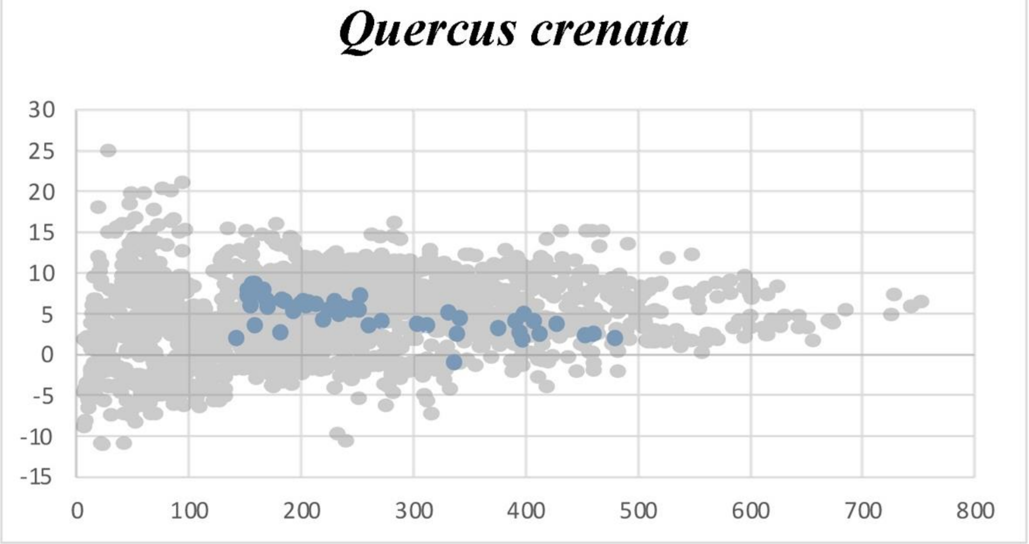


**MMP boxplots for *Quercus crenata* (n = 50)**

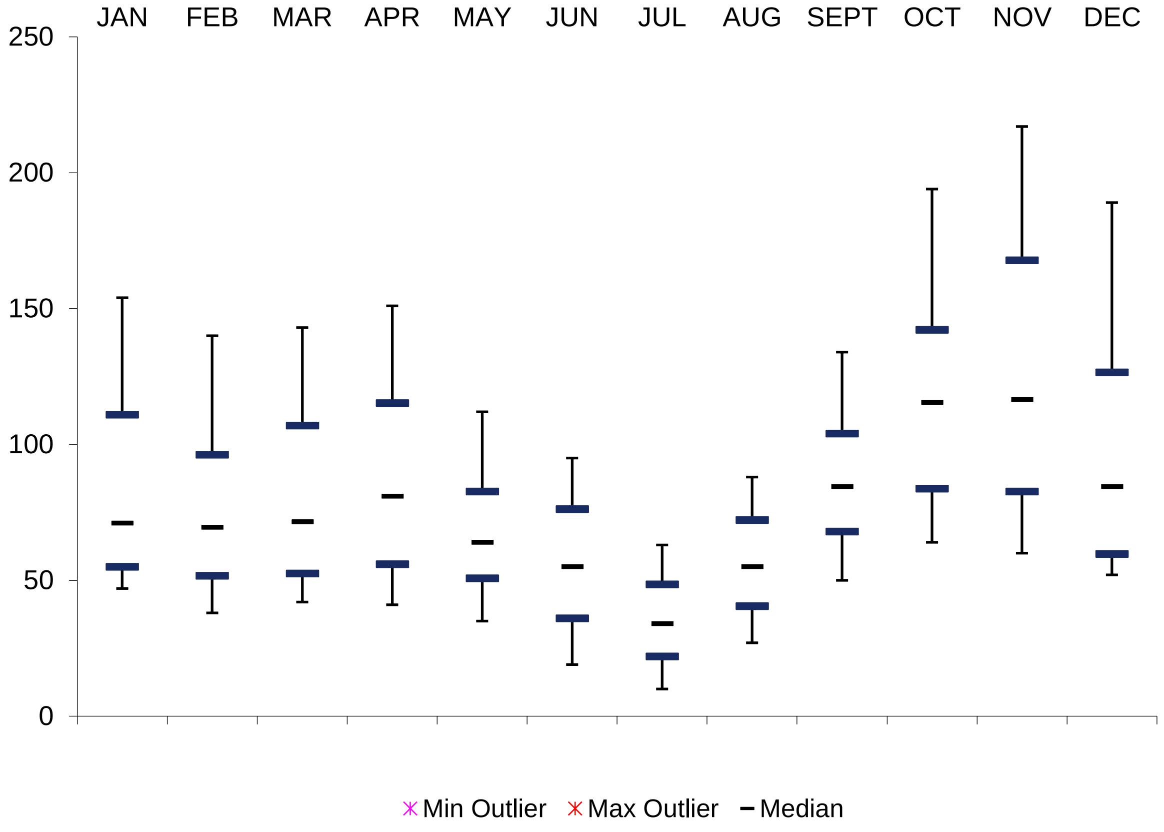

**MMT boxplots for *Quercus crenata* (n = 50)**

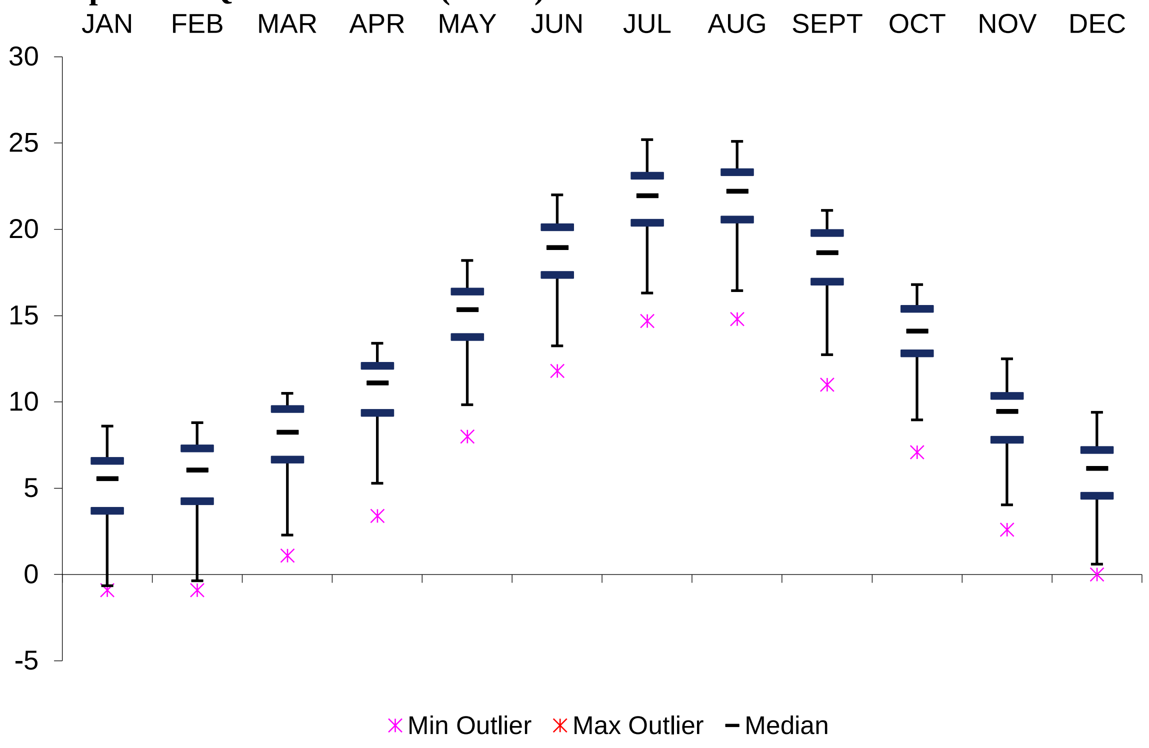


**$MT_{min}$ boxplots for *Quercus crenata* (n = 50)**

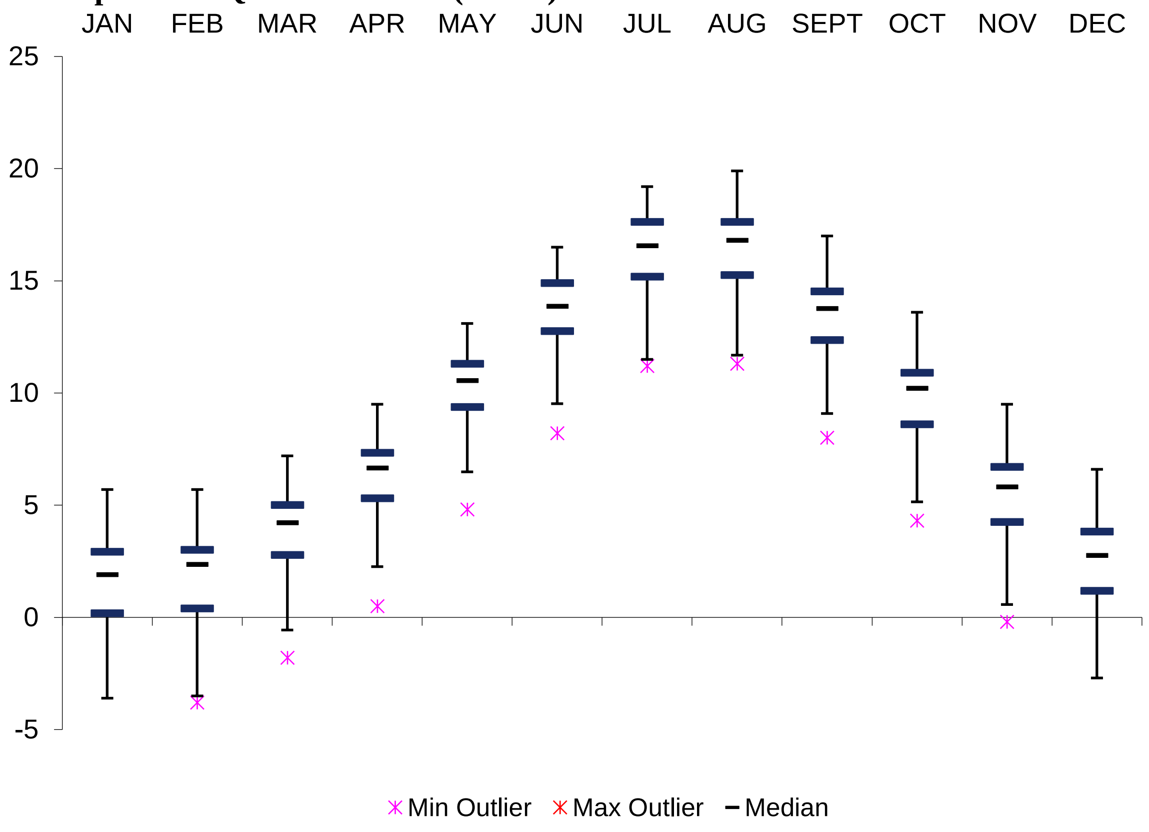

**KÖPPEN PROFILES – *Quercus suber*** L., 1753
GBIF localities of *Quercus suber* (preserved specimen)

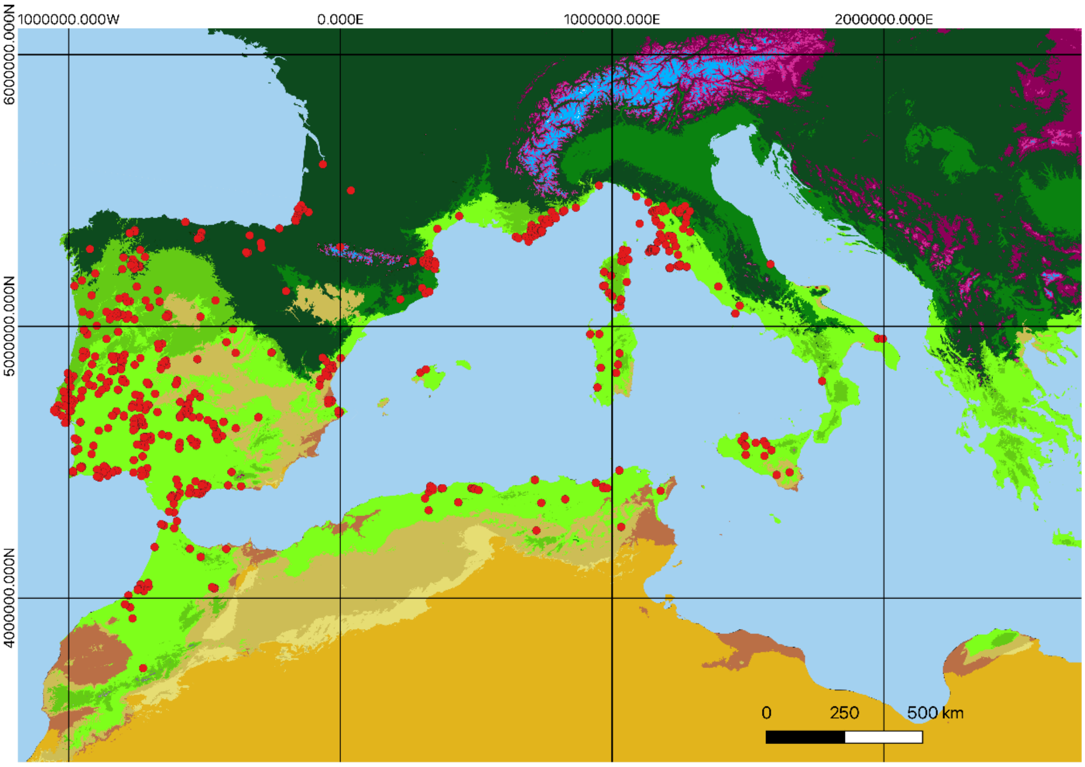


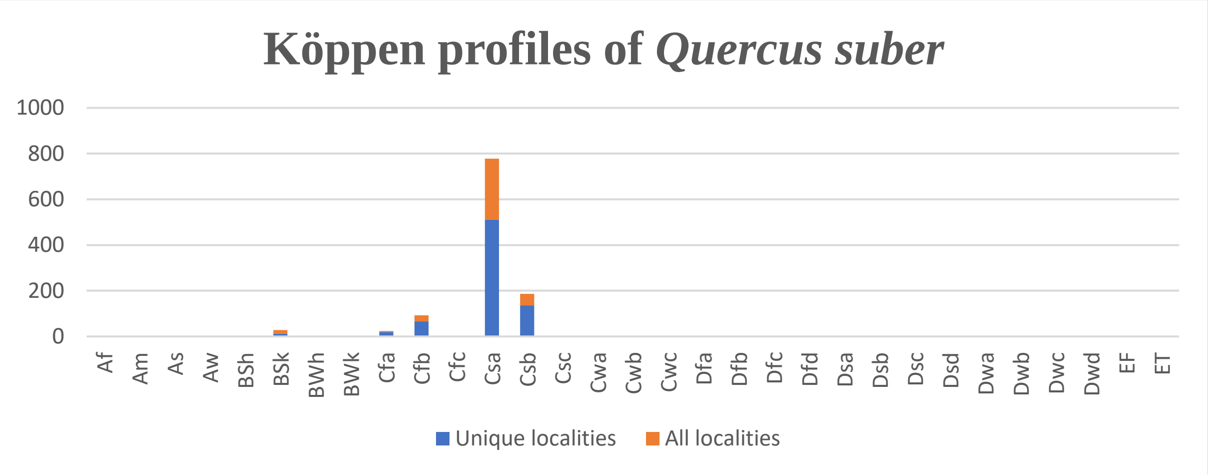


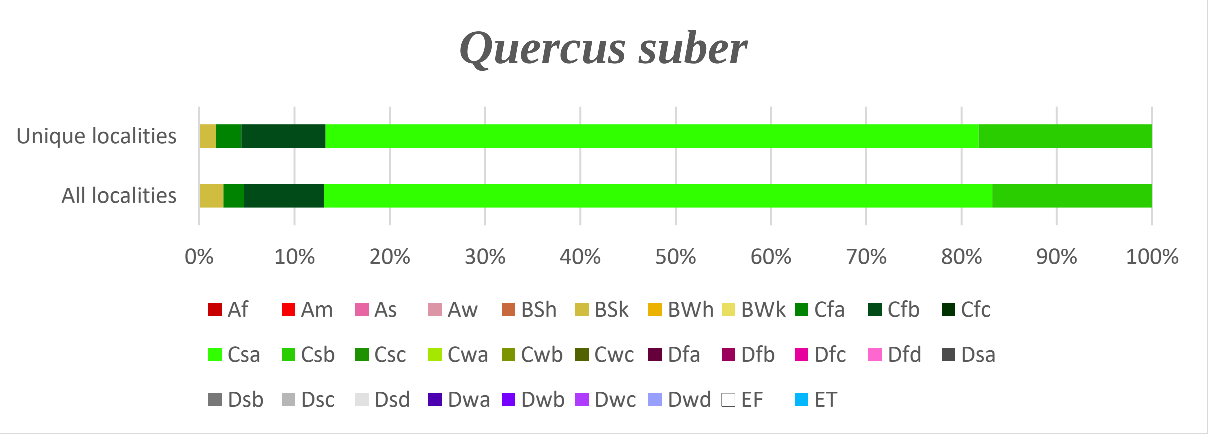


All localities (n = 1109); unique localities (n = 747)

**BIOMES – *Quercus suber***

GBIF localities of *Quercus suber* (preserved specimen)

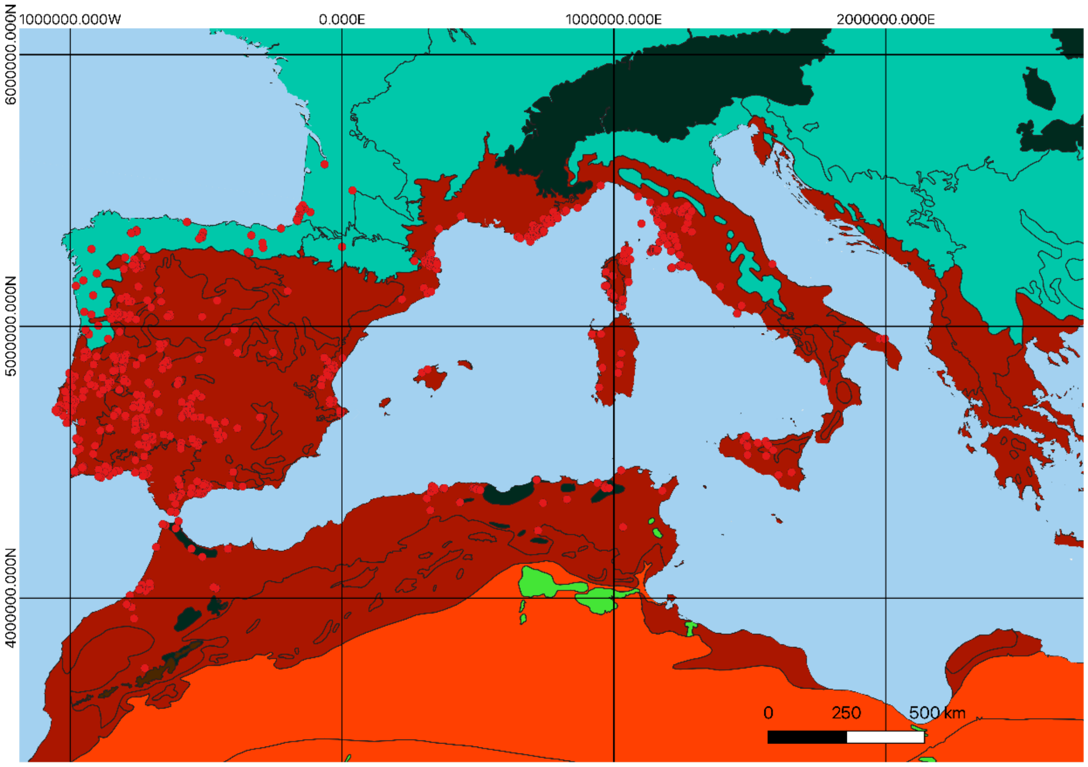


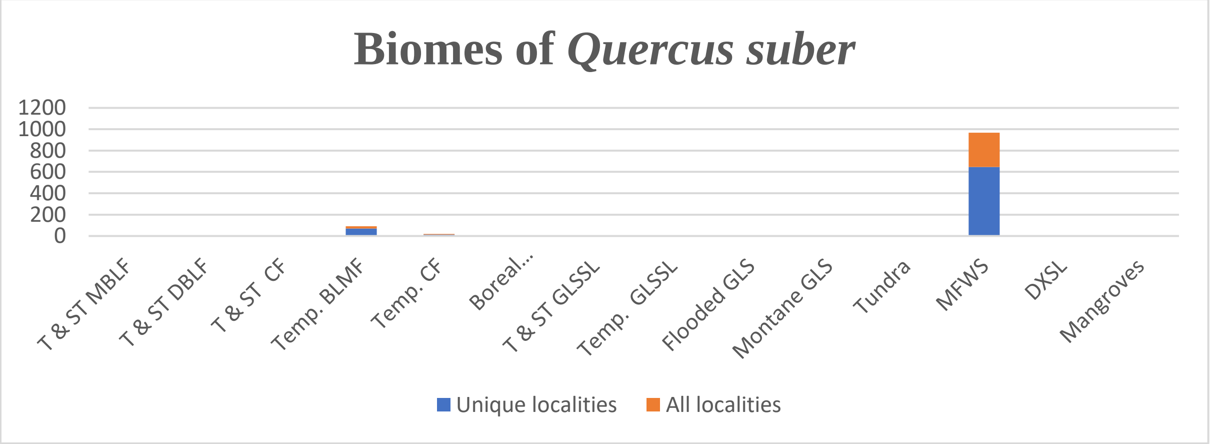


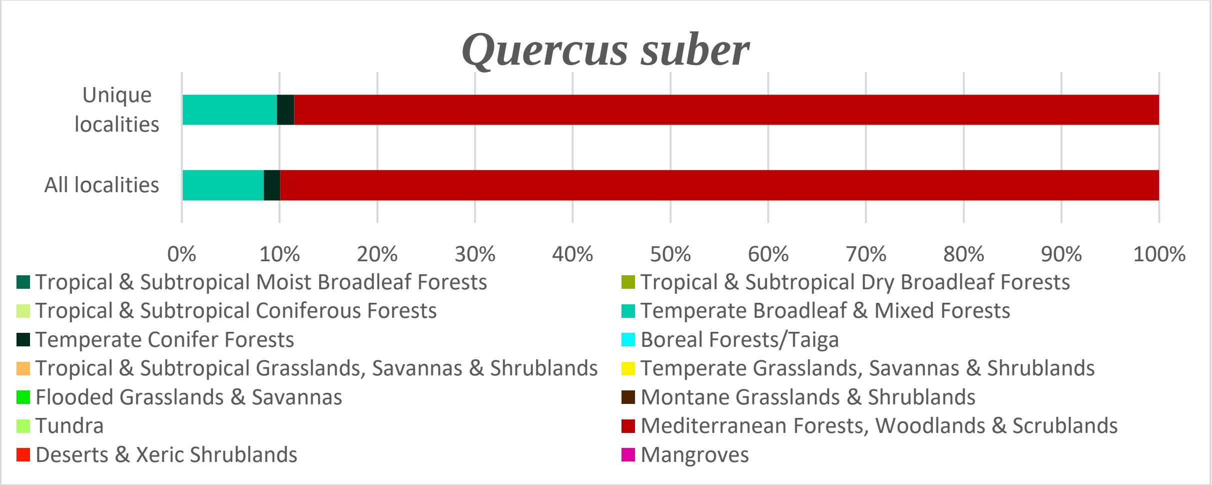


All localities (n = 1075); unique localities (n = 730)

**MTCM (x-axis) vs PCQ (y-axis) for *Quercus suber* (n = 661)**. In contrast to its less summer-draught tolerant sister, *Q. suber* prefers distinctly Mediterranean, lowland climates with very mild winters, no snow covers (→ MMT and MMP boxplots) and only very rare frosts (→ $MT_{min}$ boxplot). It is the most dry-adapted species of the entire section (MMP rarely > 150 mm/month).

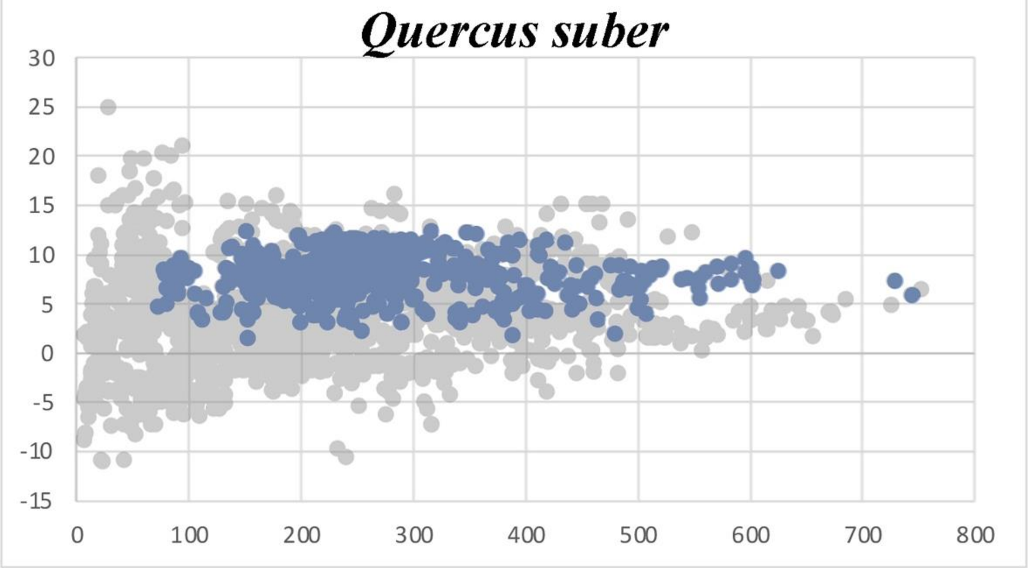


**MMP boxplots for *Quercus suber* (n = 661)**

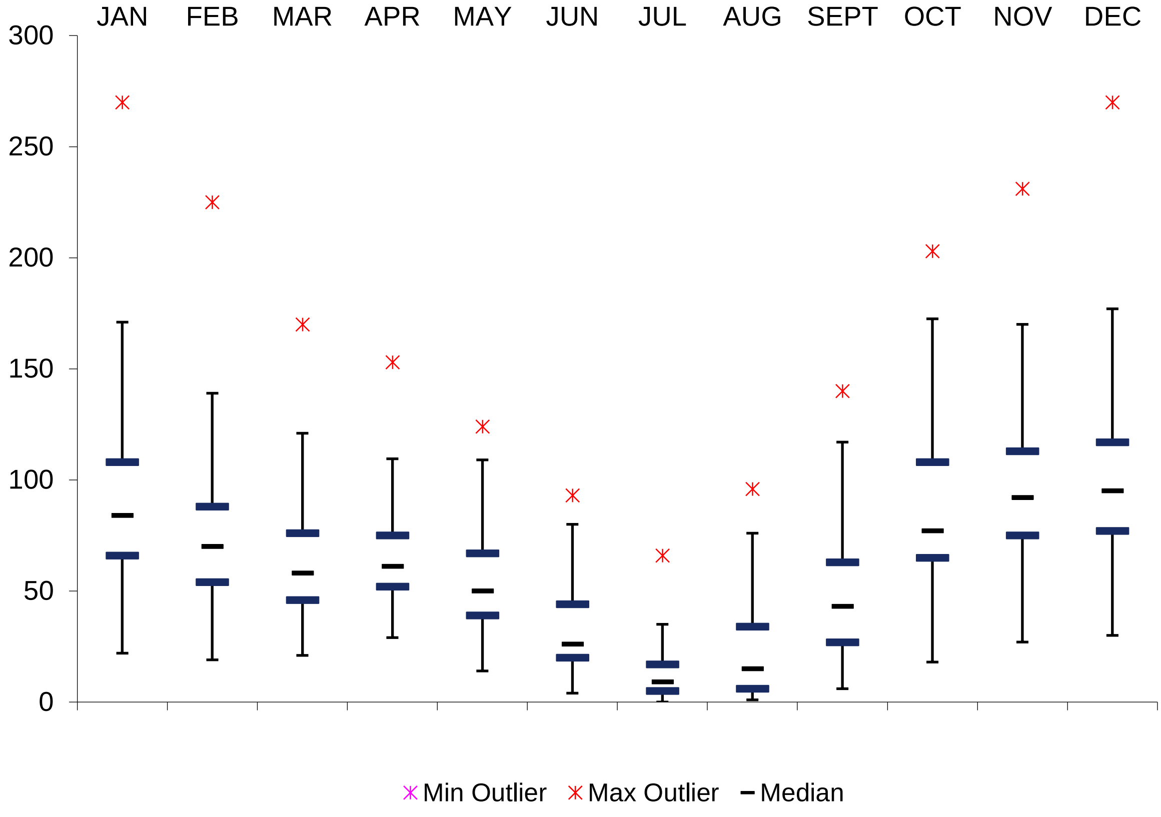

**MMT boxplots for *Quercus suber* (n = 661)**

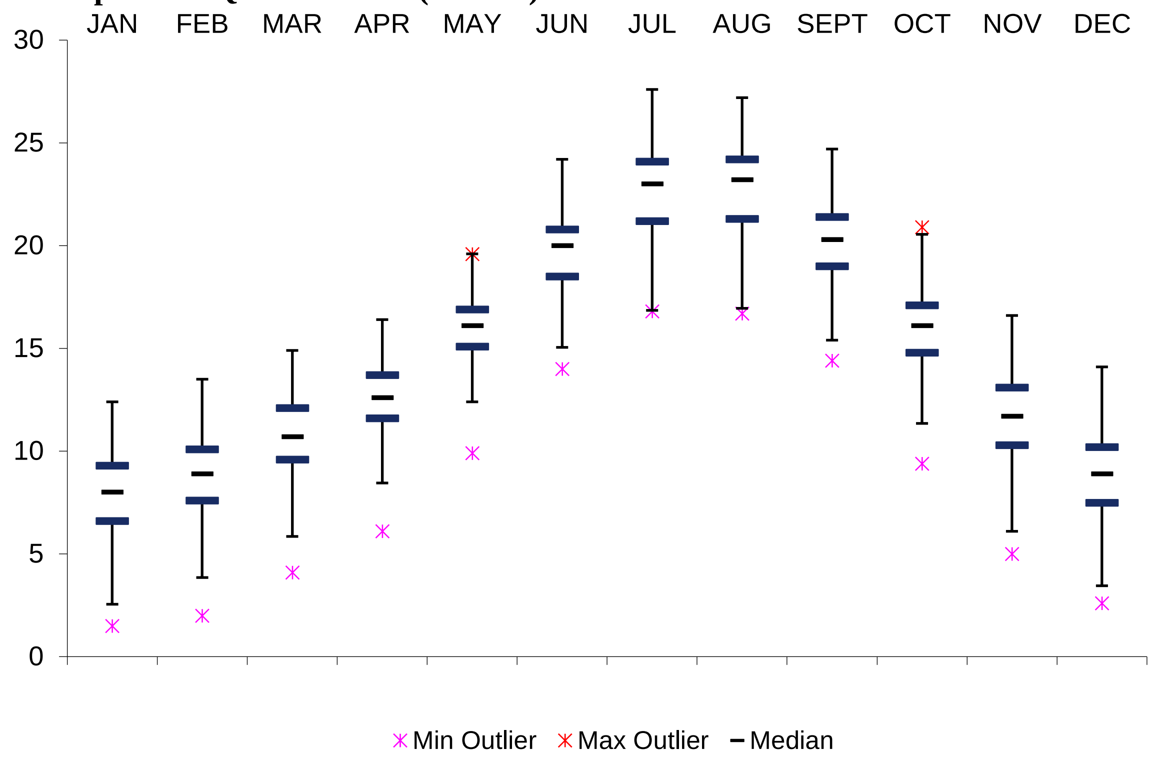


**$MT_{min}$ boxplots for *Quercus suber* (n = 661)**

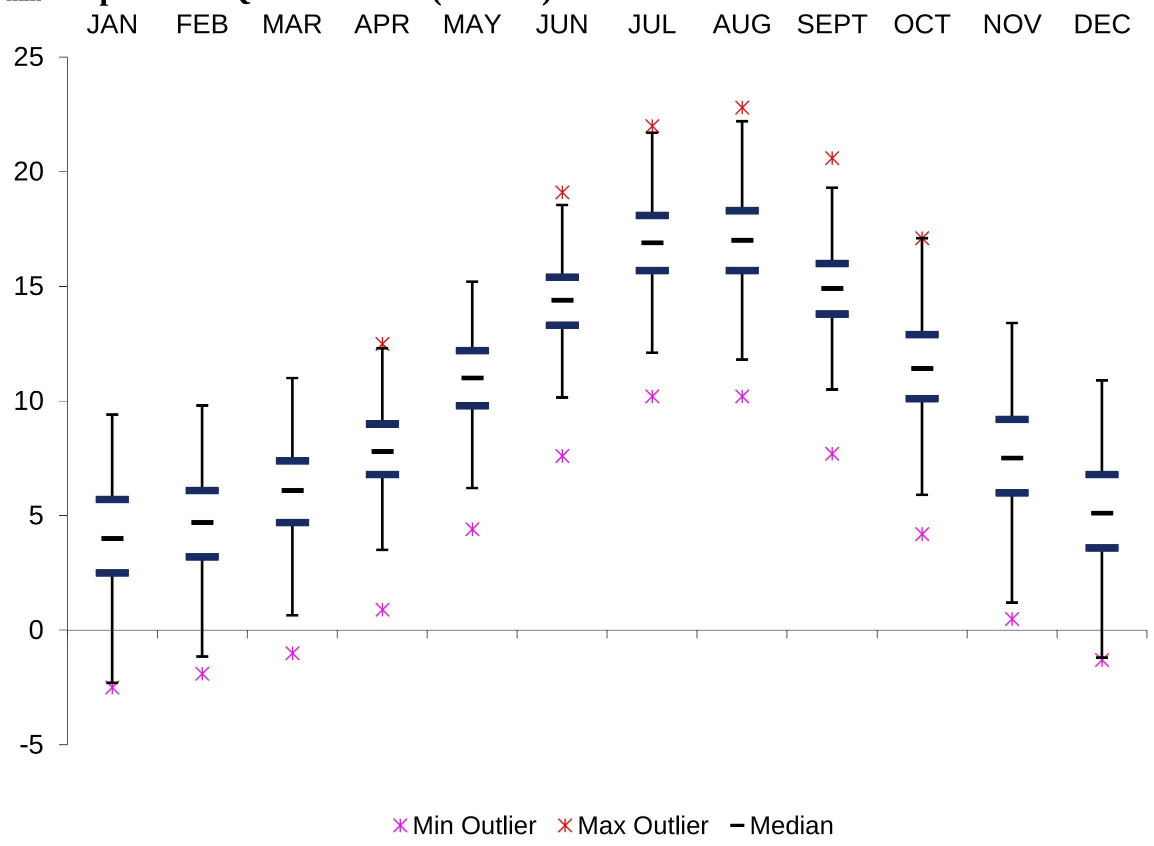

## Quercus *sect.* Cerris *subsect.* Campylolepides

Cumulative niche of *Q. acutissima*, *Q. chenii*, and *Q. variabilis*

**KÖPPEN MAP** (n = 2534)

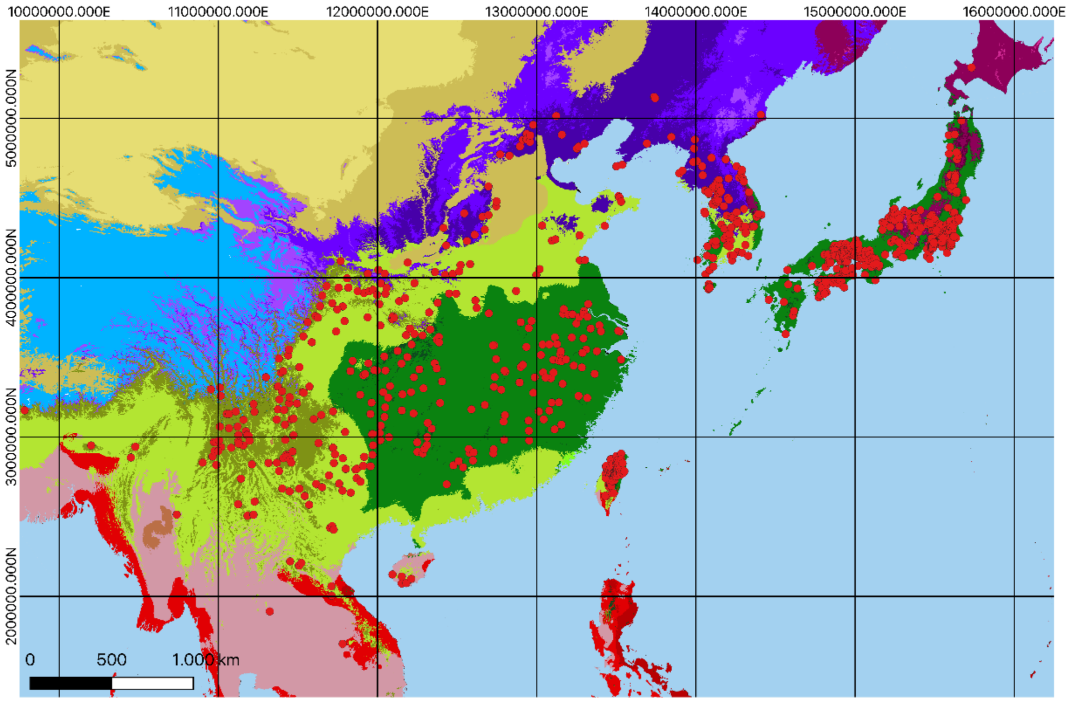


**MTCM (x-axis) vs PCQ (y-axis) for members subsect. *Campylolepides* (n = 1298)**. Covering the largest area of all subsections of sect. *Cerris*, the only East Asian cork oak lineage thrives under different situations than most of its western siblings, including (marginal) tropical (MTCM > 18 °C) and hot subtropical (MTCM > 10 °C) with dry winters (PCQ < 100 mm) and generally moist, perhumid climates (central China), while being nearly absent from continental arid (*BS*) or snow (*D*) climates of central and northern East Asia.

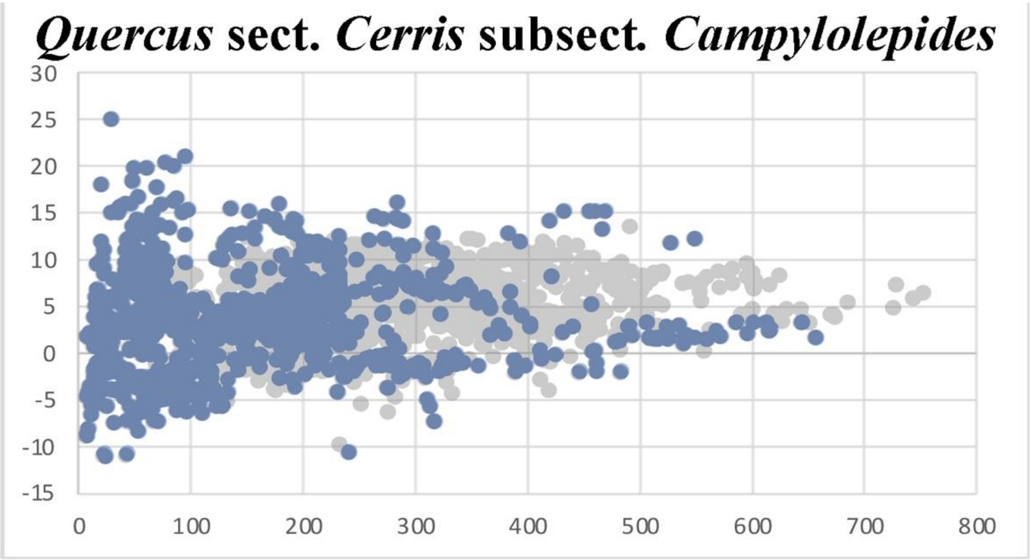

**KÖPPEN PROFILES – *Quercus acutissima* Carruth., 1861**
GBIF localities of *Q. acutissima* (preserved specimen)

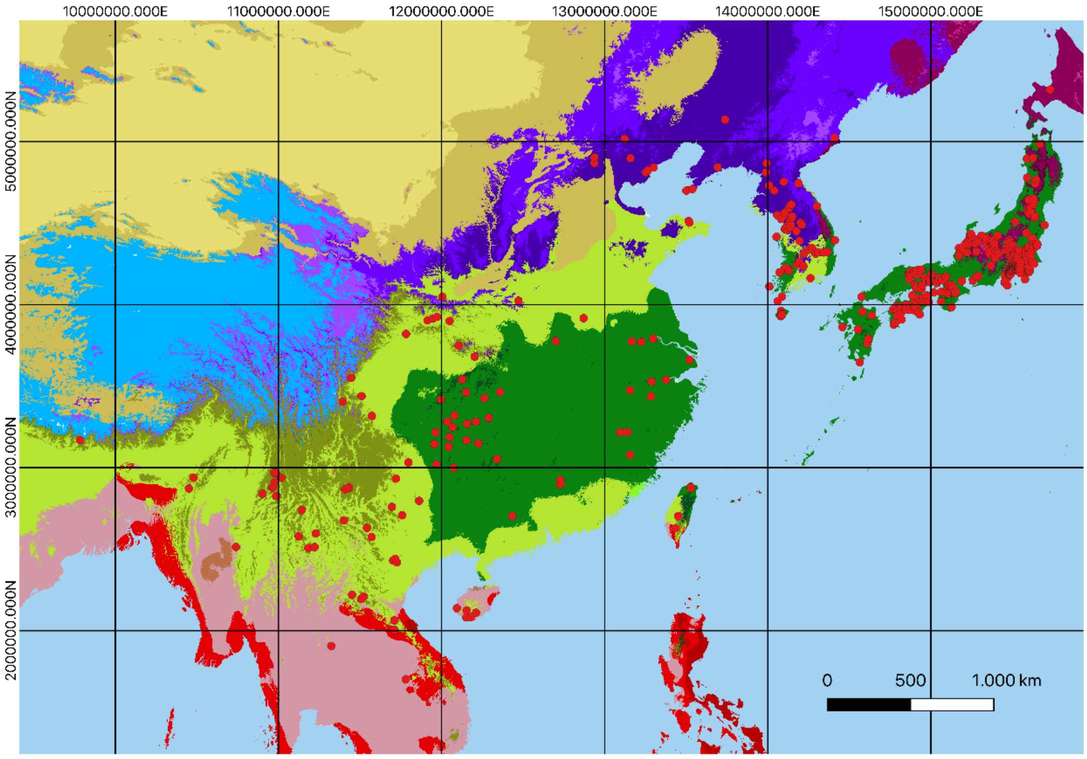


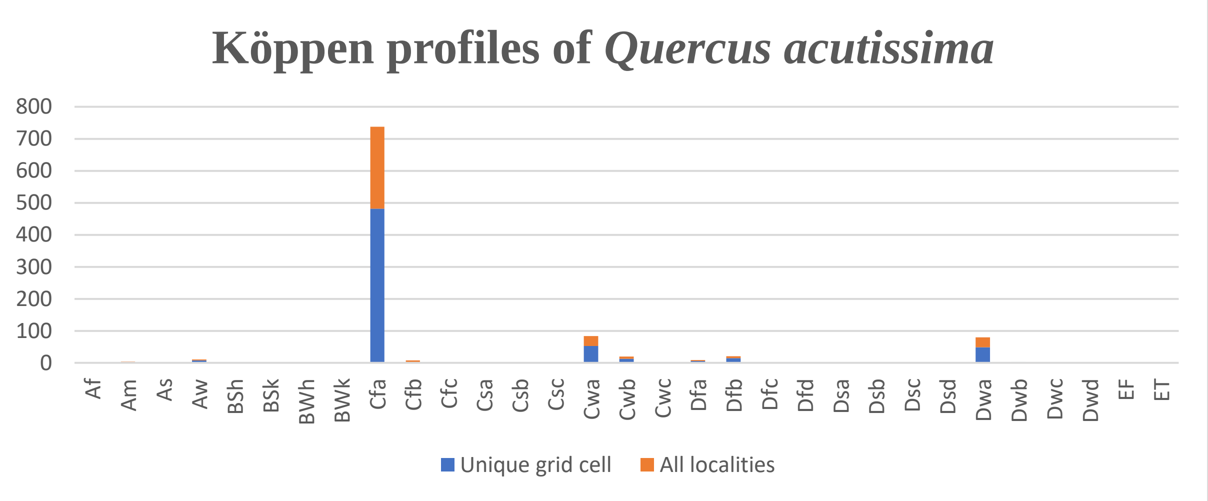


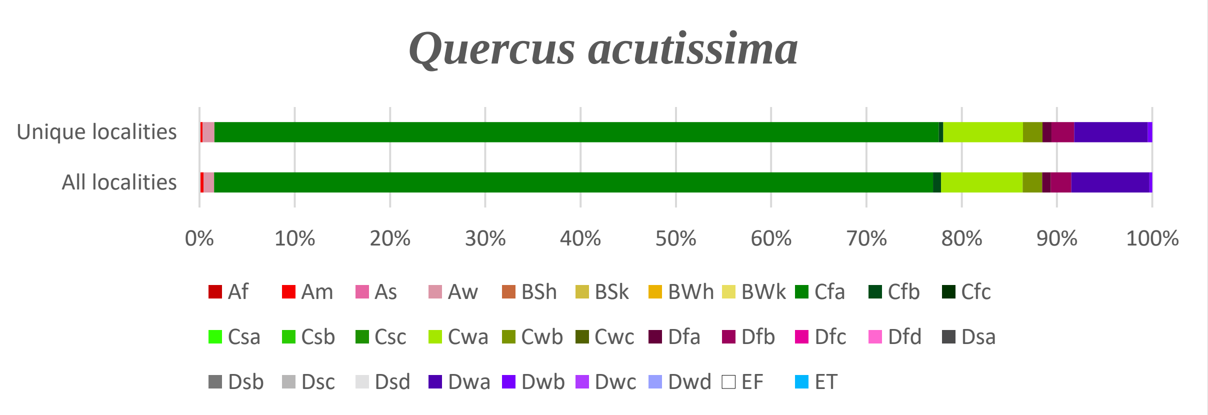


All localities (n = 978); unique localities (n = 634)

**BIOMES – *Quercus acutissima***

GBIF localities of *Q. acutissima* (preserved specimen)

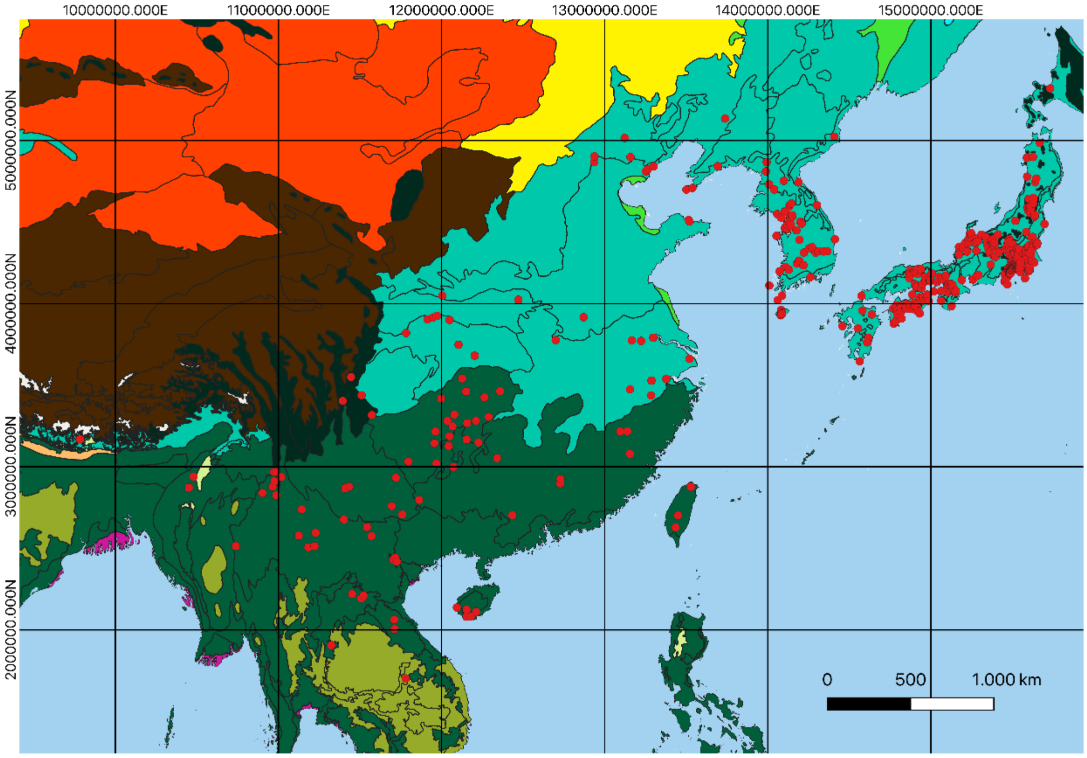


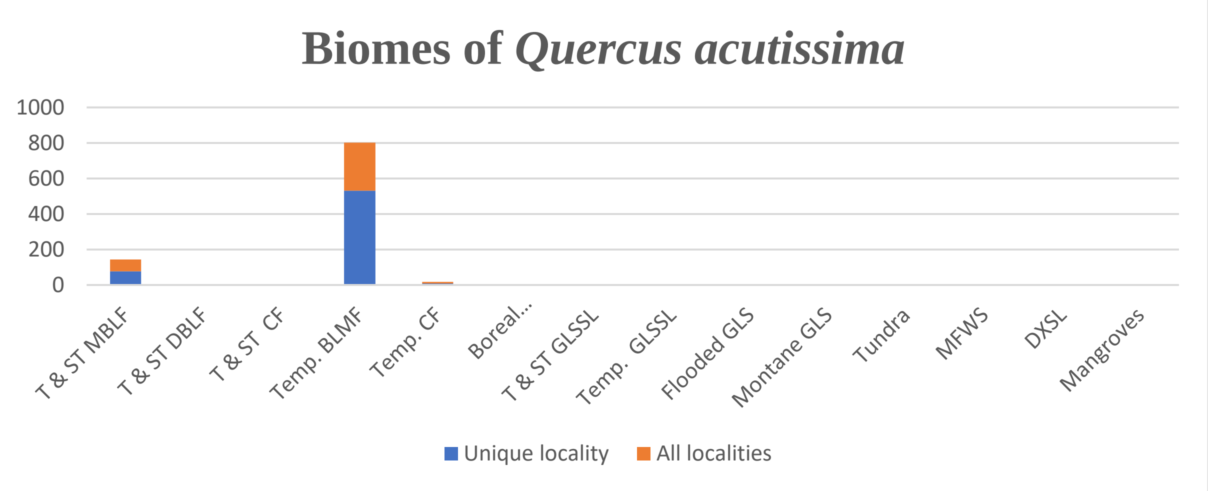


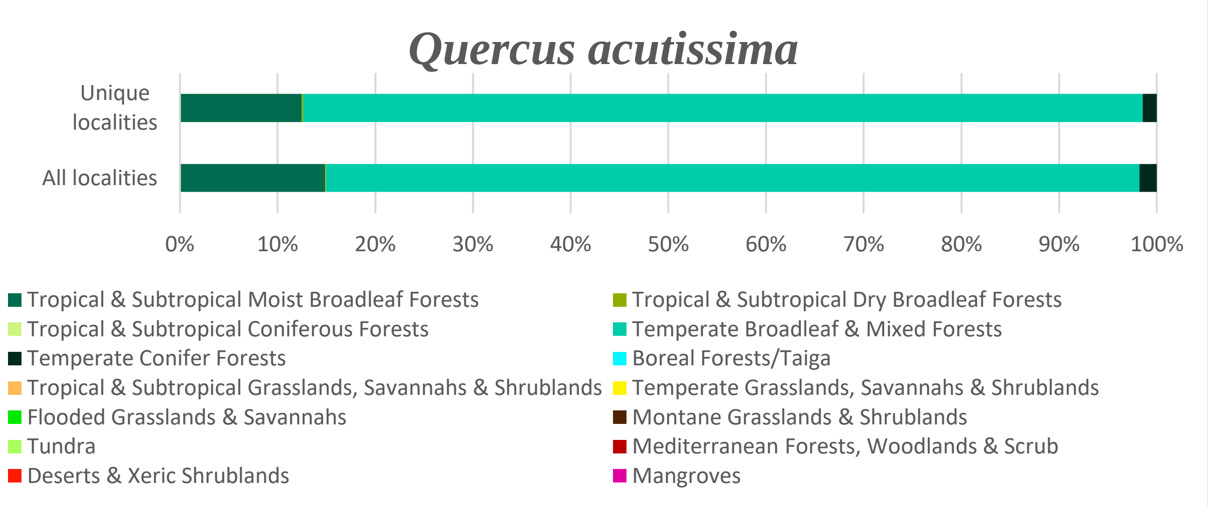


All localities (n = 962); unique localities (n = 619)

**MTCM (x-axis) vs PCQ (y-axis) for *Quercus acutissima* (n = 603).** A prototypical 'meridio-nemoral' species according Denk et al. (2013), thriving in perhumid (PCQ = 50–250 mm) or winter-dry (PCQ < 300 mm; station data from Lieth et al., 1999), snow-free climates with typically hot and (very) moist summers (East Asian Monsoon), and without severe frosts (→ $MT_{min}$ boxplot).

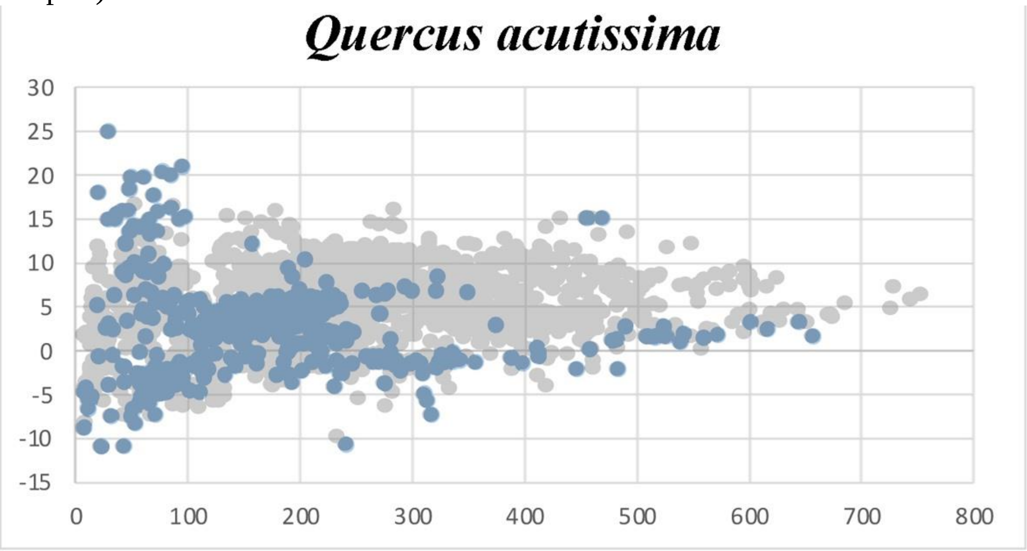


**MMP boxplots for *Quercus acutissima* (n = 603)**

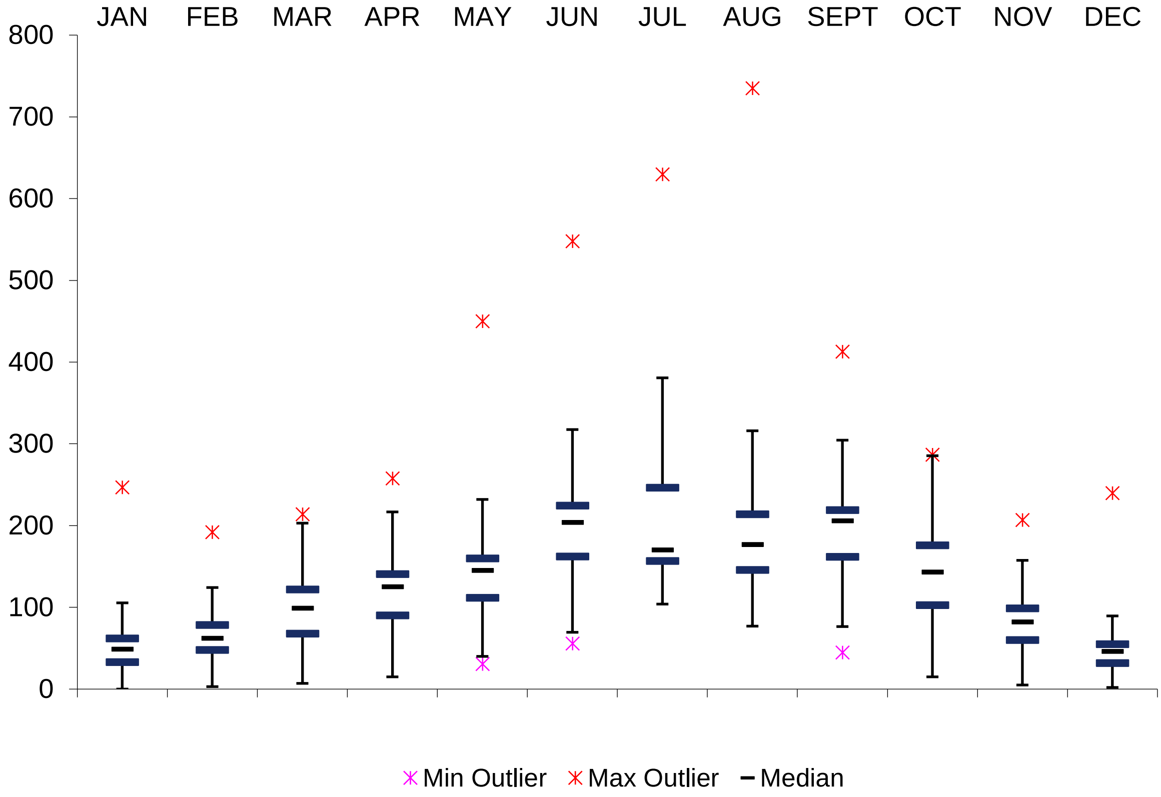

**MMT boxplots for *Quercus acutissima* (n = 603)**

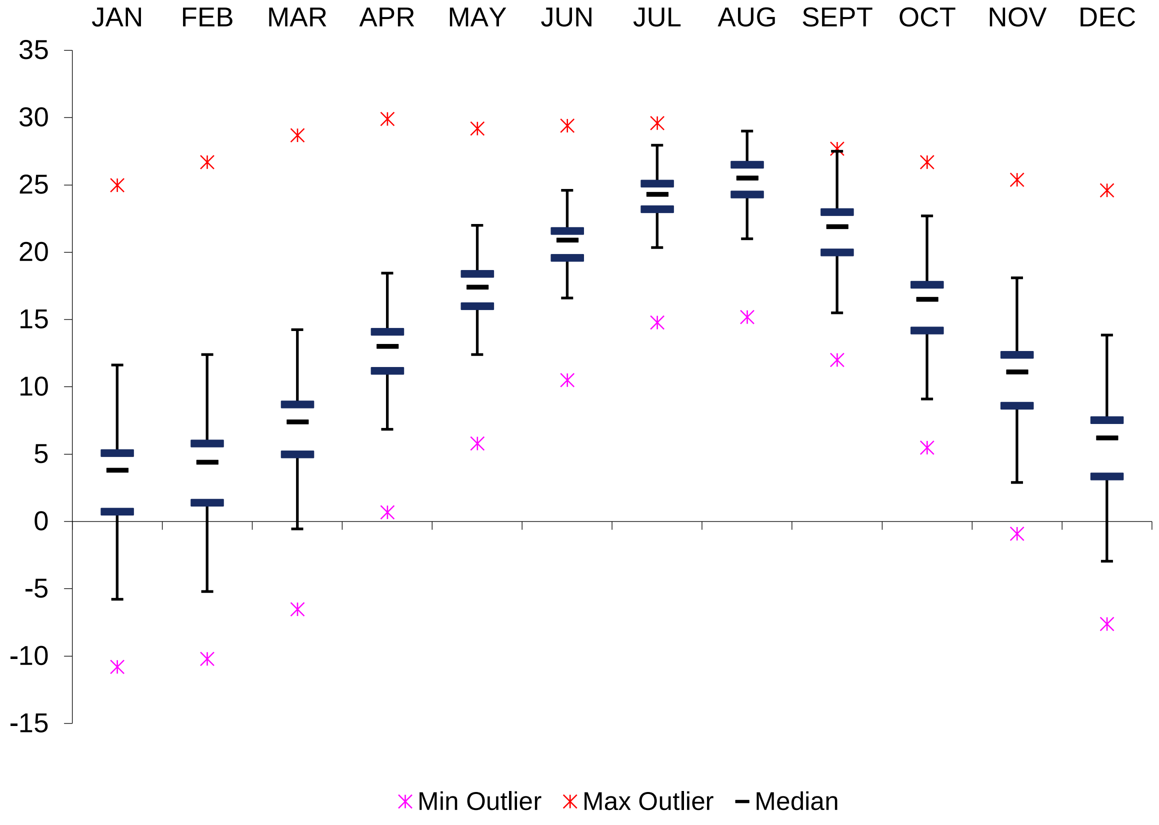


**MT$_{min}$ boxplots for *Quercus acutissima* (n = 603)**

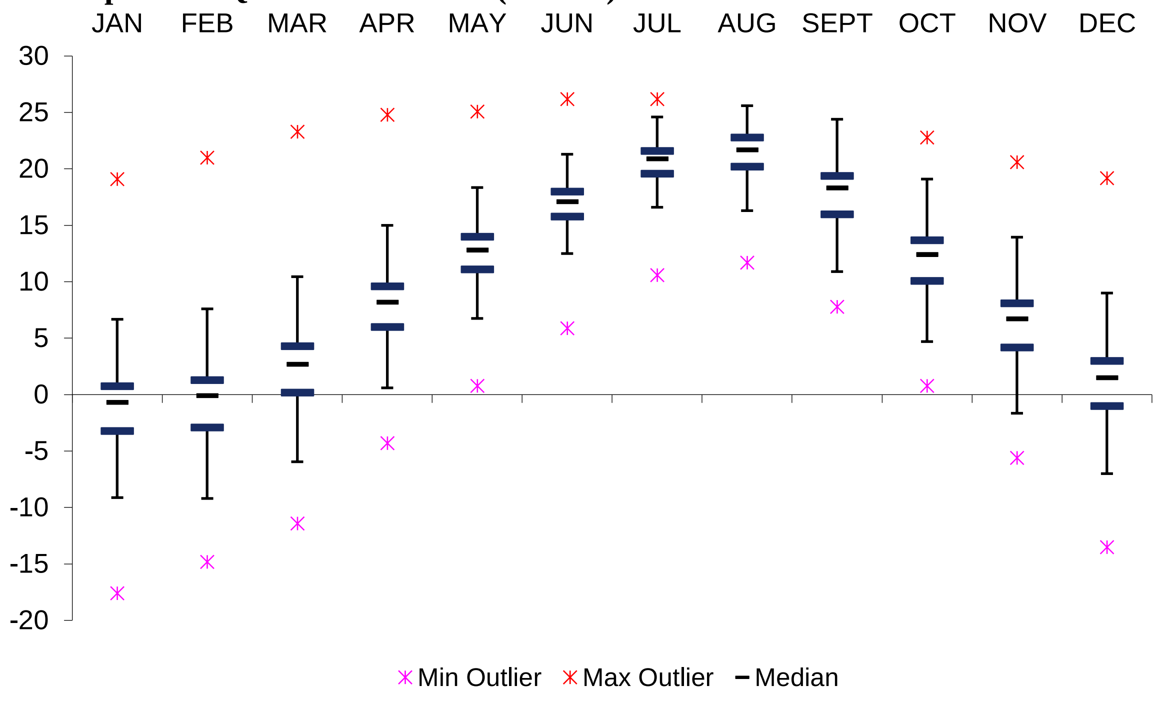

**KÖPPEN PROFILES – *Quercus chenii* Nakai, 1924**
GBIF localities of *Q. chenii* (preserved specimen)

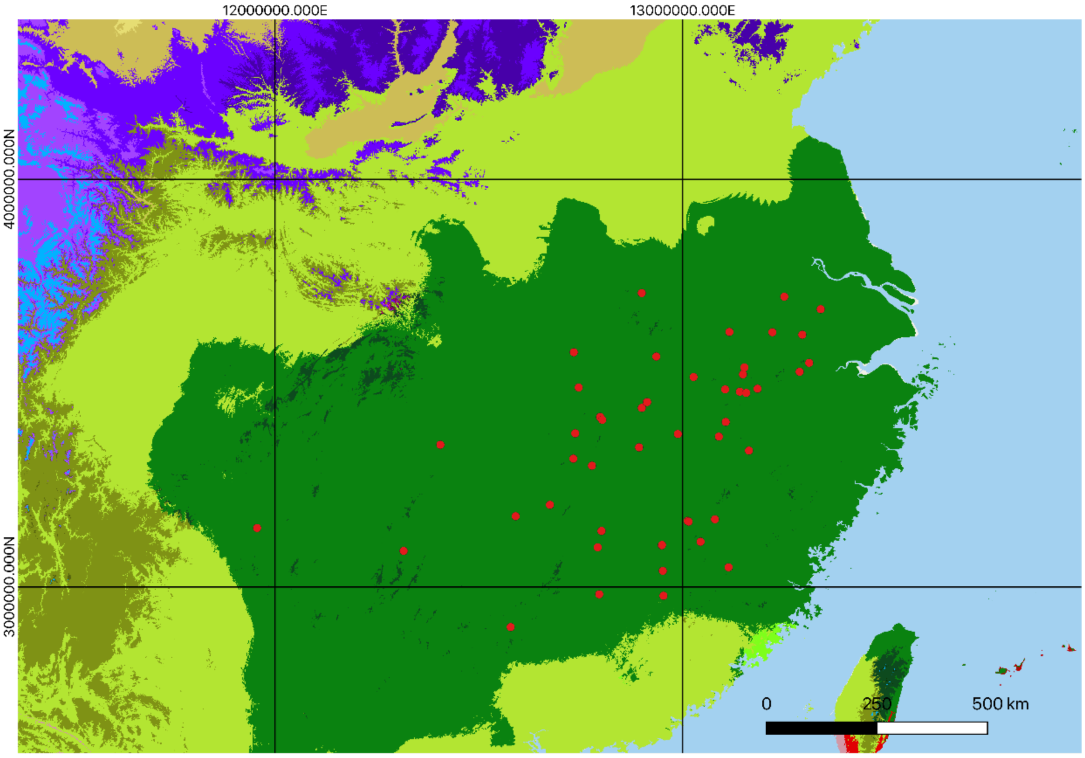


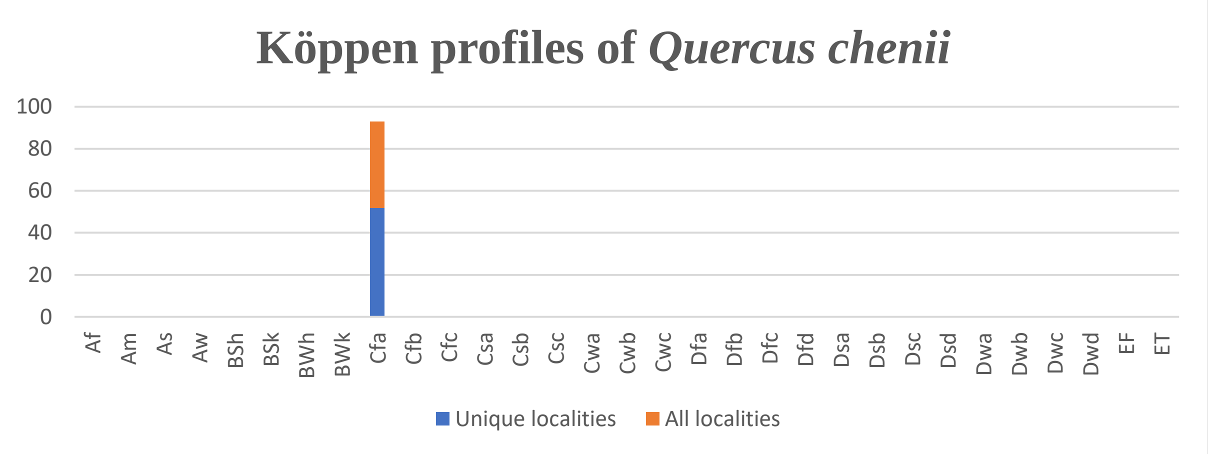


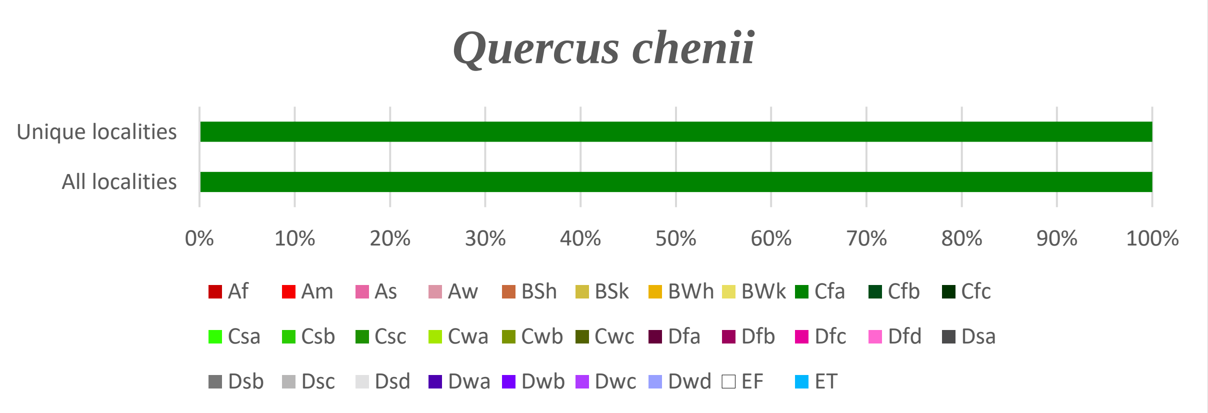


All localities (n = 93); localities (n = 52)

**BIOMES – *Quercus chenii***
GBIF localities of *Q. chenii* (preserved specimen)

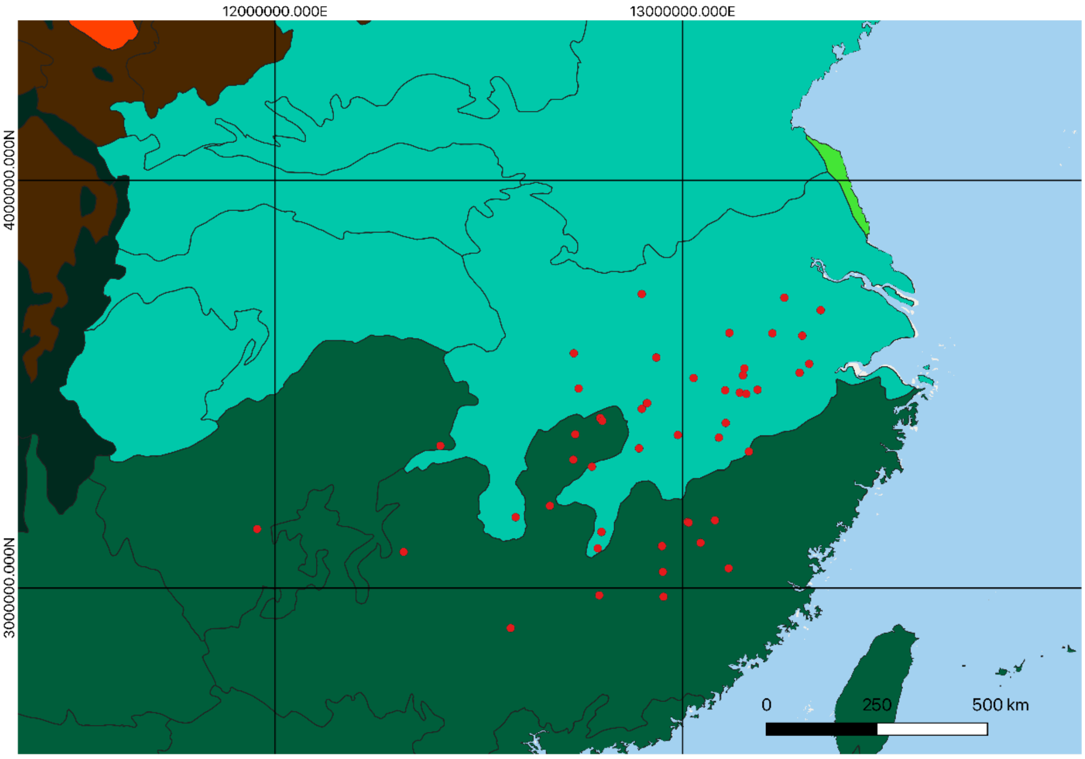


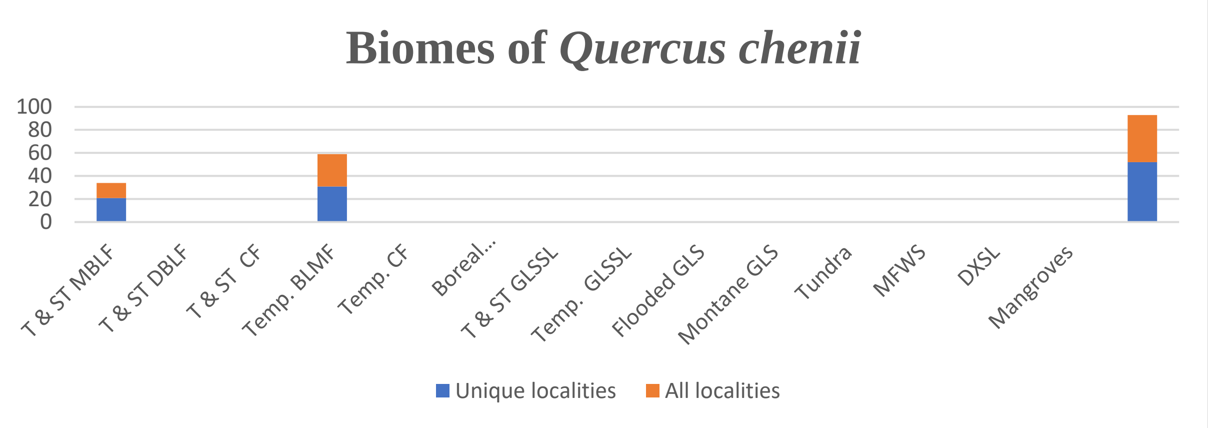


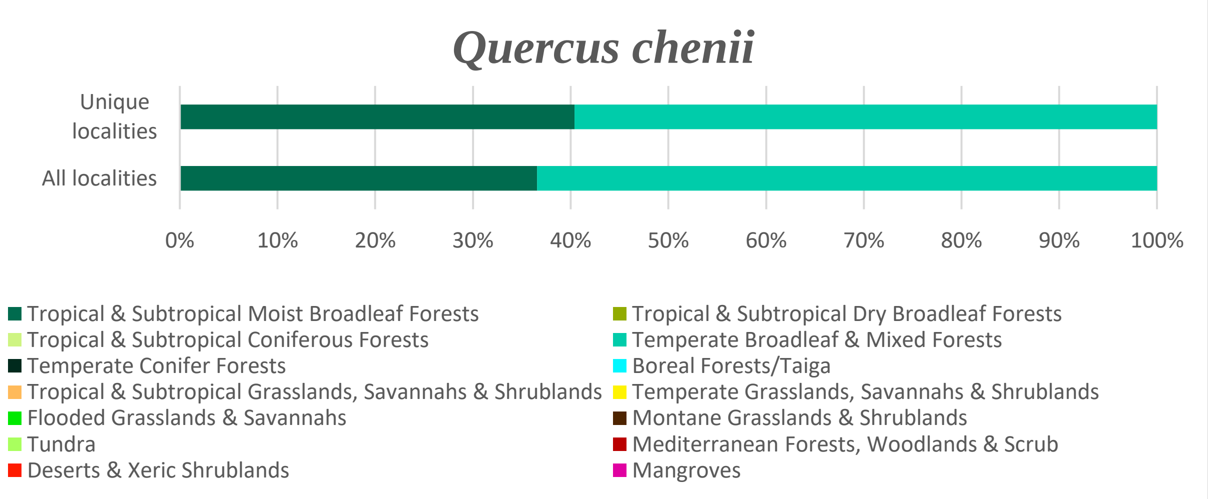


All localities, n = 93; unique localities, n = 52

**MTCM (x-axis) vs PCQ (y-axis) for *Quercus chenii* (n = 52).** A geographically restricted, strongly niched species, covering the core niche of subsect. *Campylolepides* with winter and temperature situations similar to those of the Iranian relict *Q. castaneifolia*. In contrast to *Q. castanifolia*, *Q. chenii* has its precipitation maximum in late spring/early summer (April to June) and a 2–3-times higher monthly amount (→ MMP boxplot).

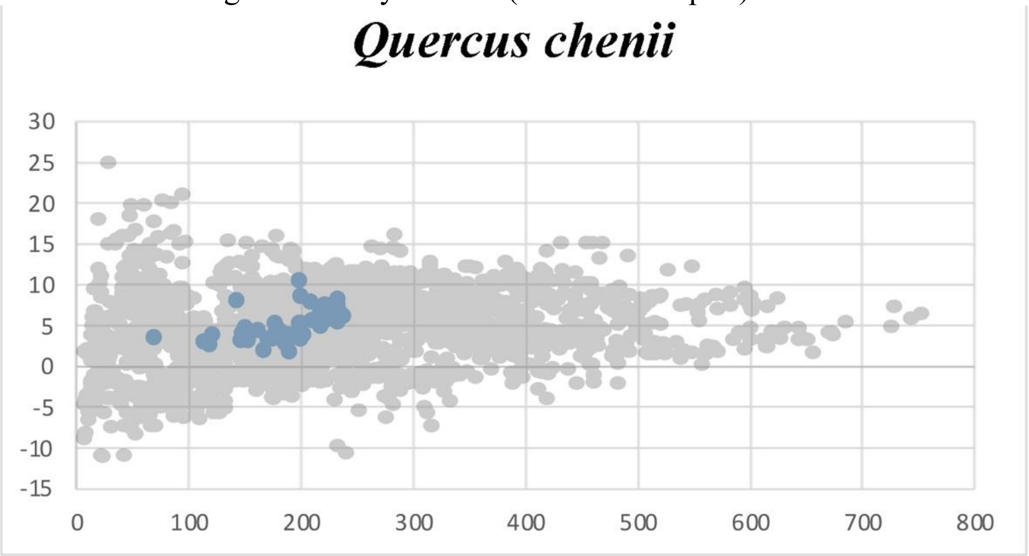


**MMP boxplots for *Quercus chenii*, n = 52**

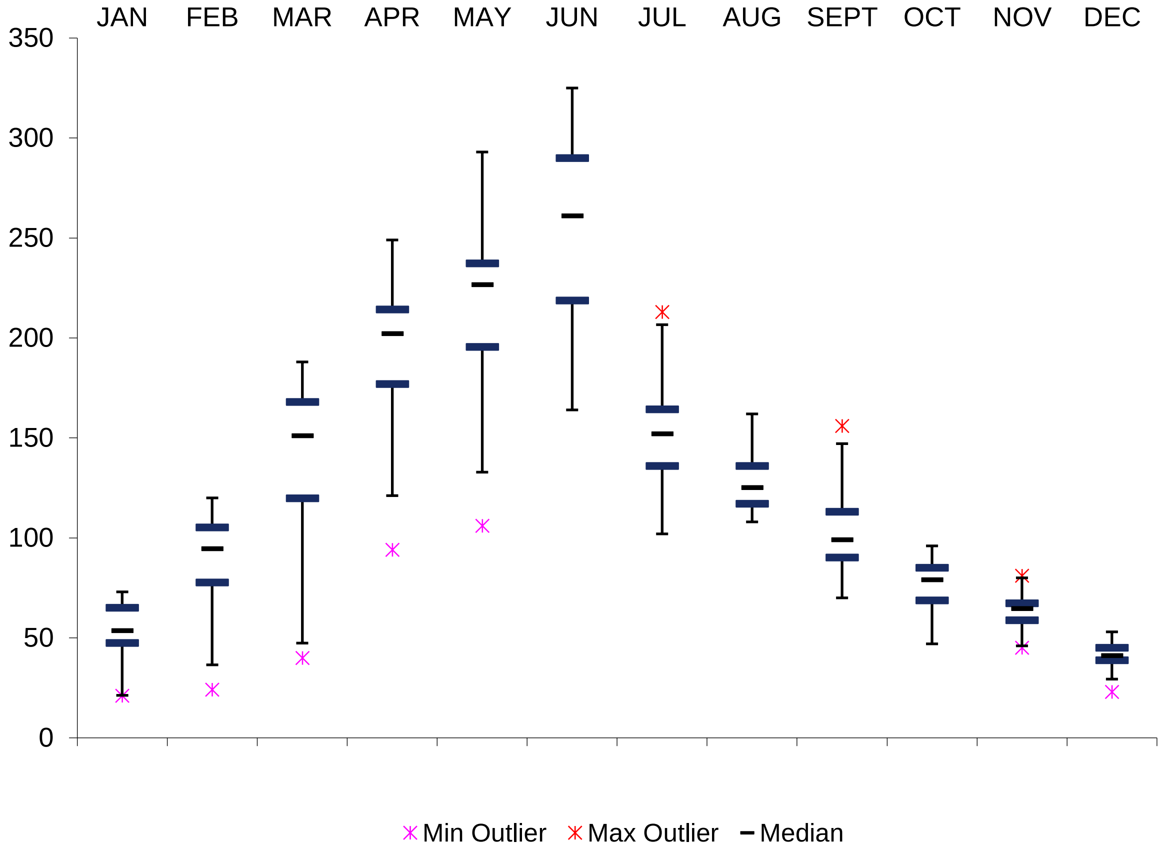

**MMT boxplots for *Quercus chenii*, n = 52**

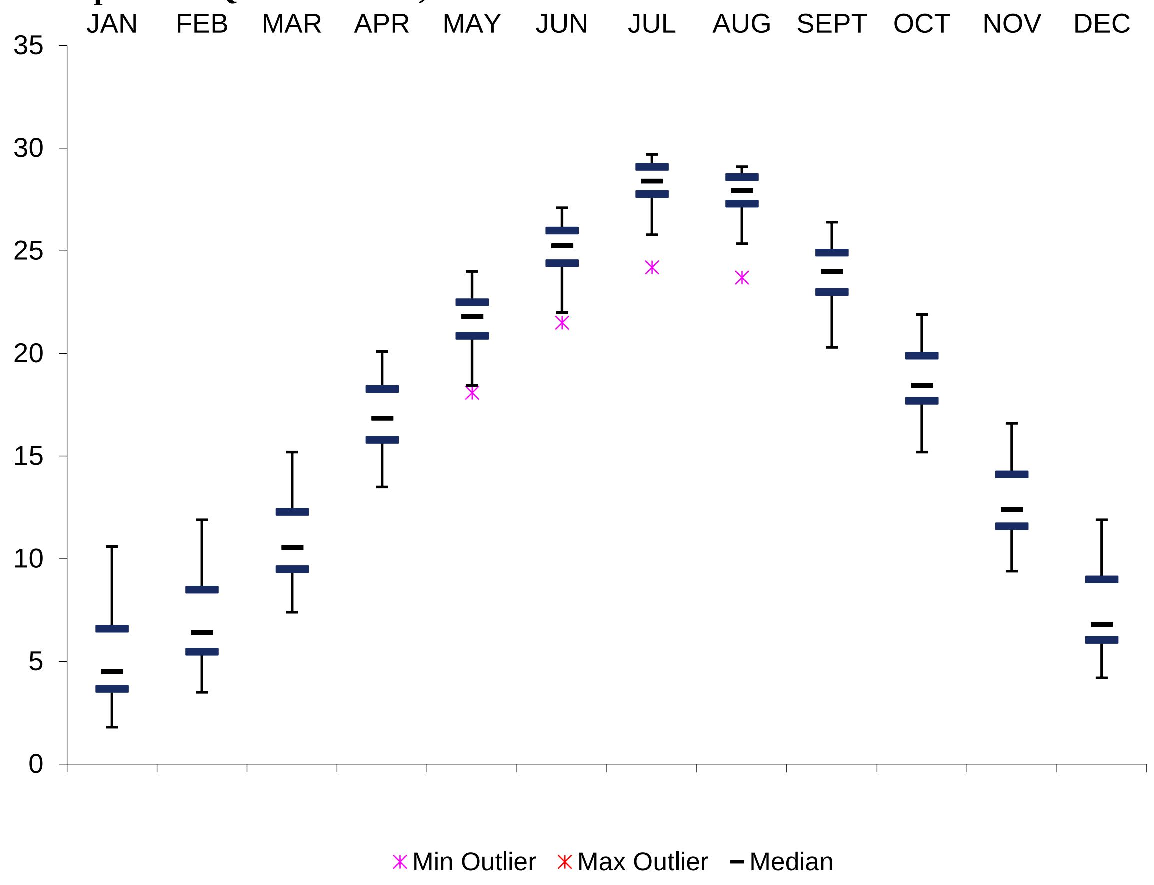


**$MT_{min}$ boxplots for *Quercus chenii*, n = 52**

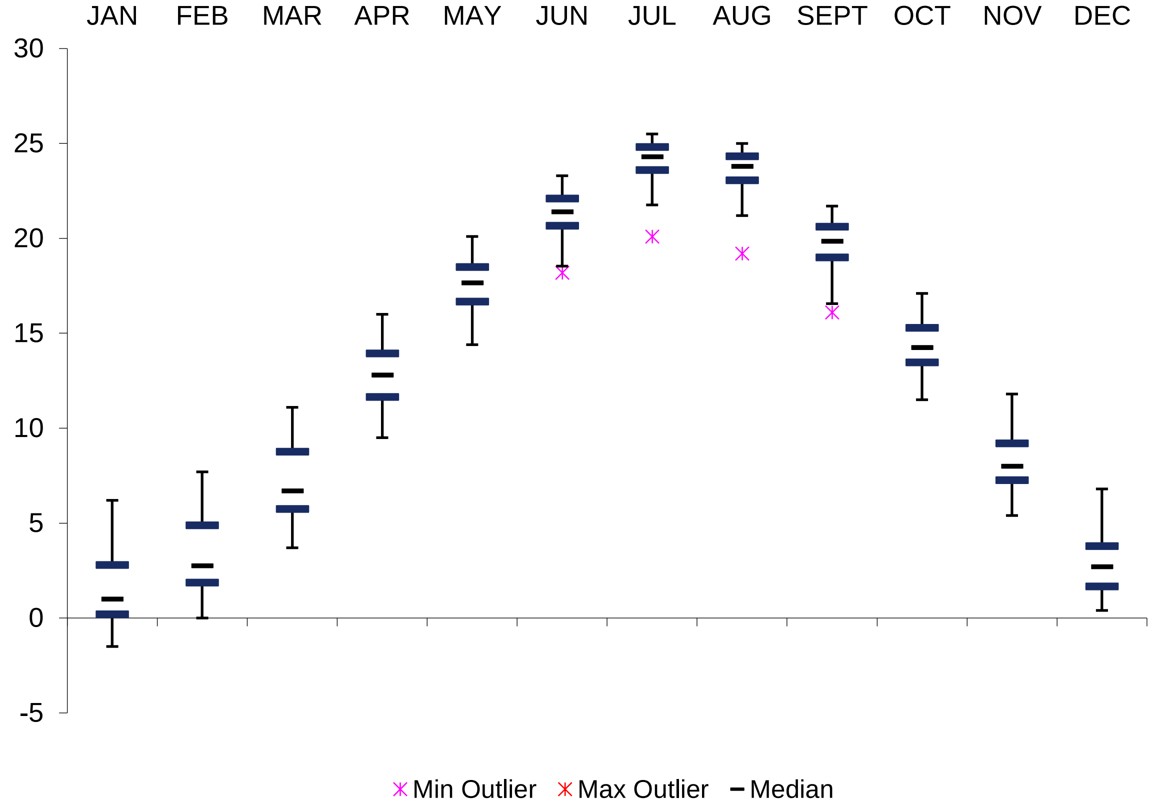

**KÖPPEN SIGNATURES – *Quercus variabilis*** Blume, 1851
GBIF localities of *Q. variabilis* (preserved specimen)

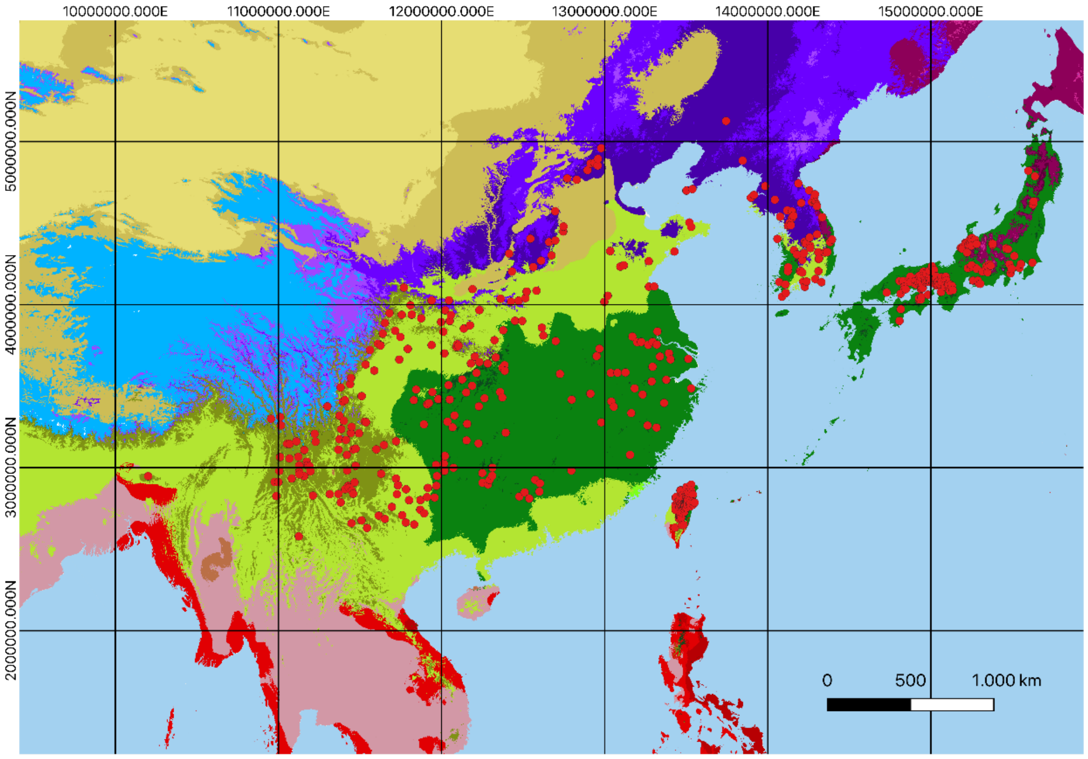


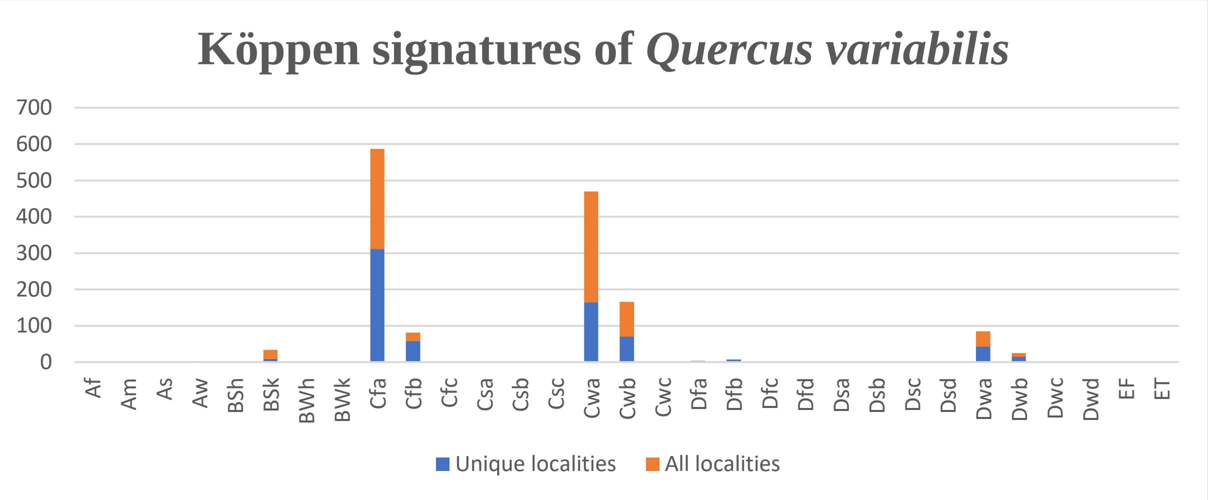


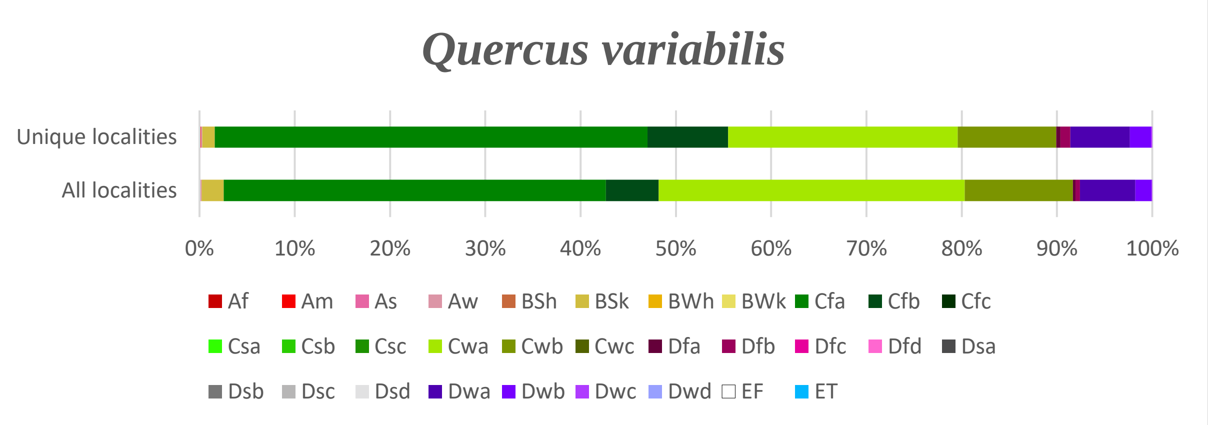


All localities, n = 1463; unique grid cells, n = 685

**BIOMES – *Quercus variabilis***

GBIF localities of *Q. variabilis* (preserved specimen)

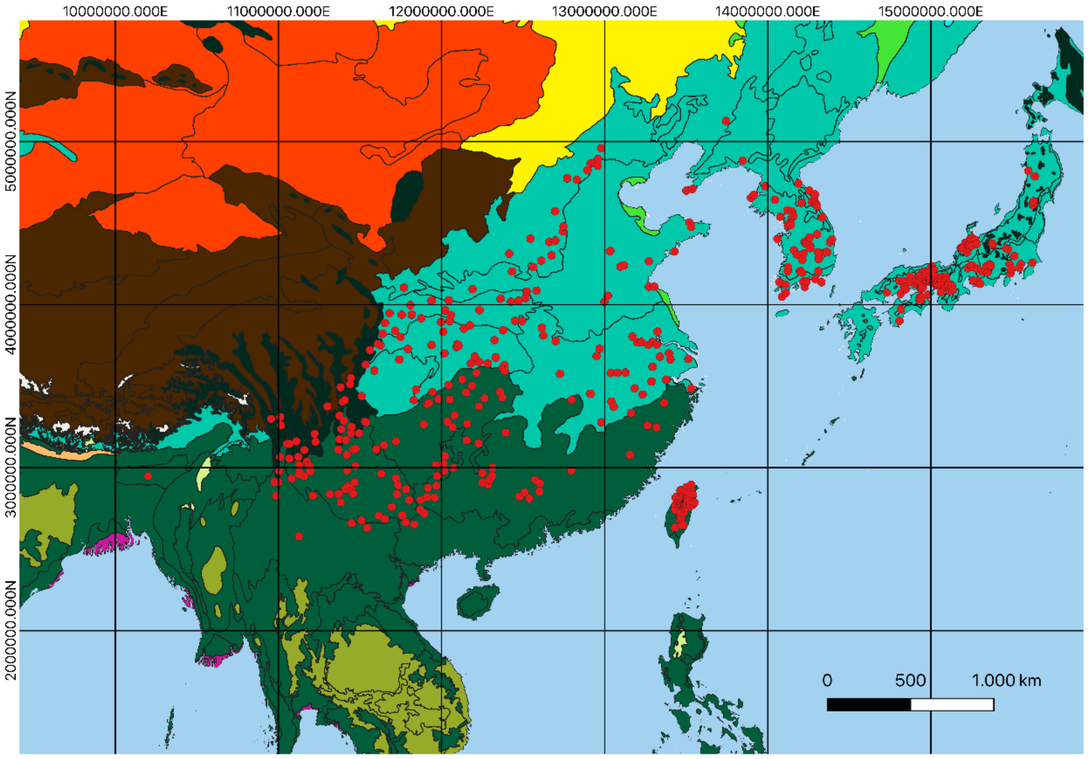


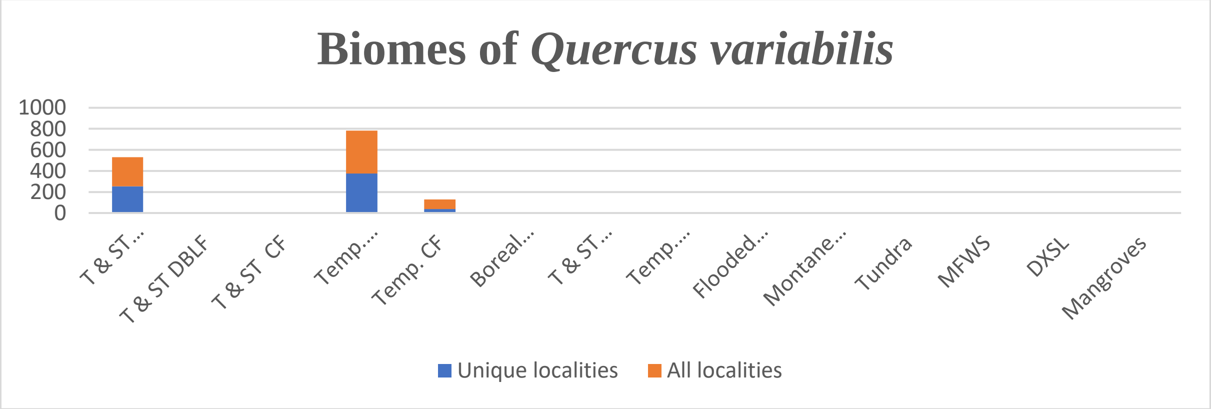


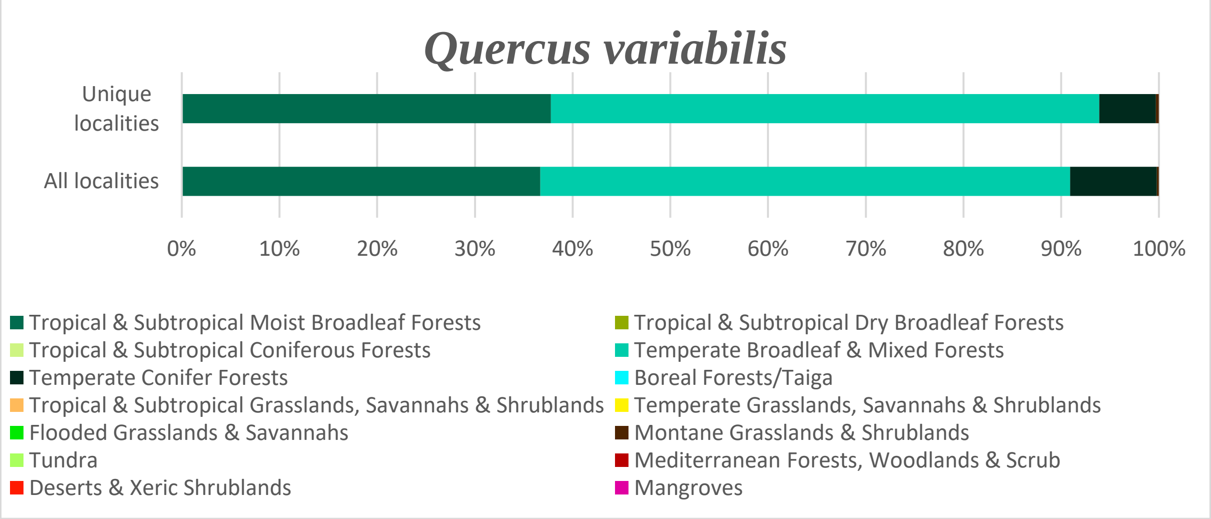


All localities, n = 1444; unique localities, n = 670

**MTCM (x-axis) vs PCQ (y-axis) for *Quercus variabilis* (n = 643).** The most widespread and climatically most variable of all East Asian species. Its niche incorporates those of its sisters, except that it leaves out tropical situations (MTCM > 18 °C, PCQ < 100 mm, i.e. *Aw* climates) in contrast to *Q. acutissima*. Temperature-wise is nearly equals the tolerance of *Q. libani* by enduring distinctly cold, while relatively dry winters.

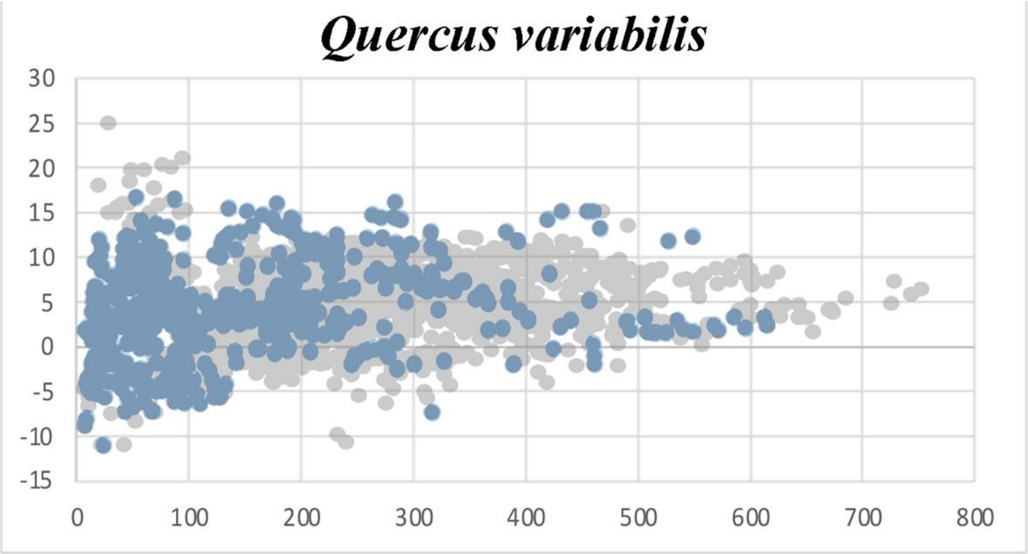


**MMP boxplots for *Quercus variabilis*, n = 643**

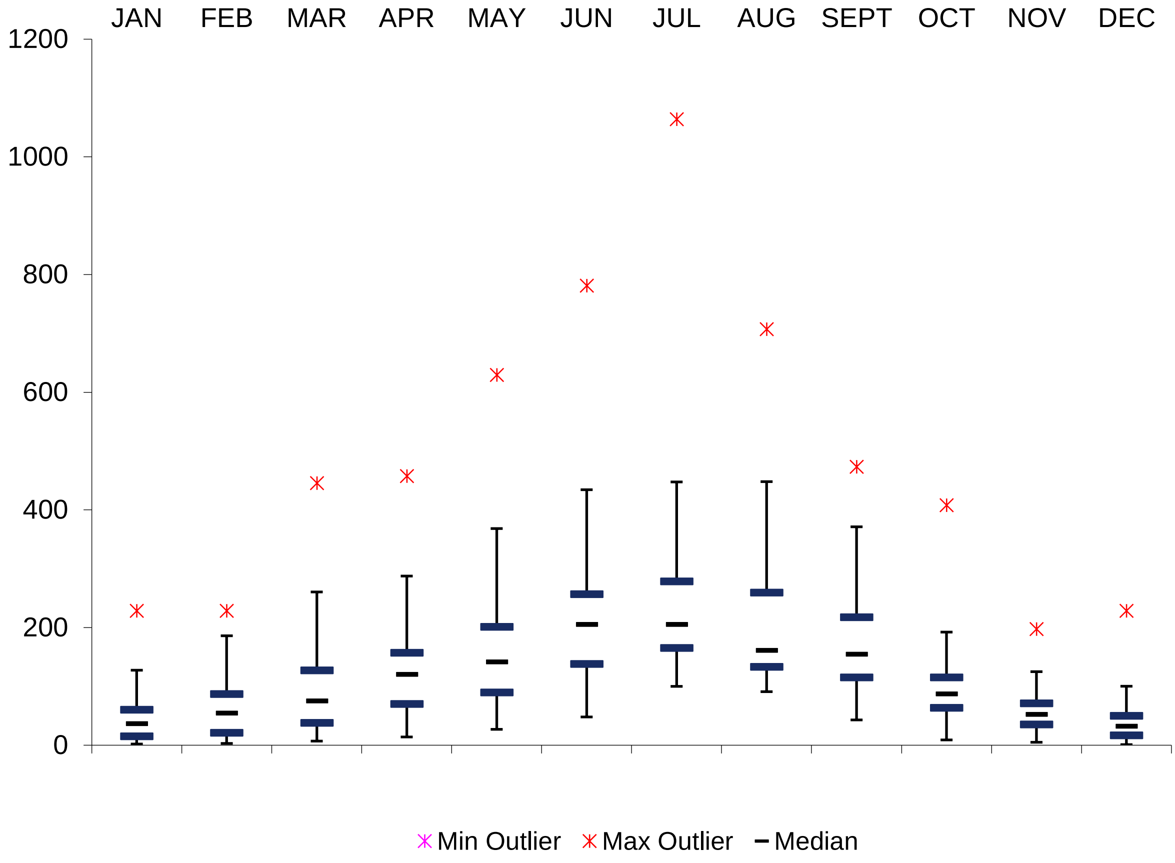

**MMT boxplots for *Quercus variabilis*, n = 643**

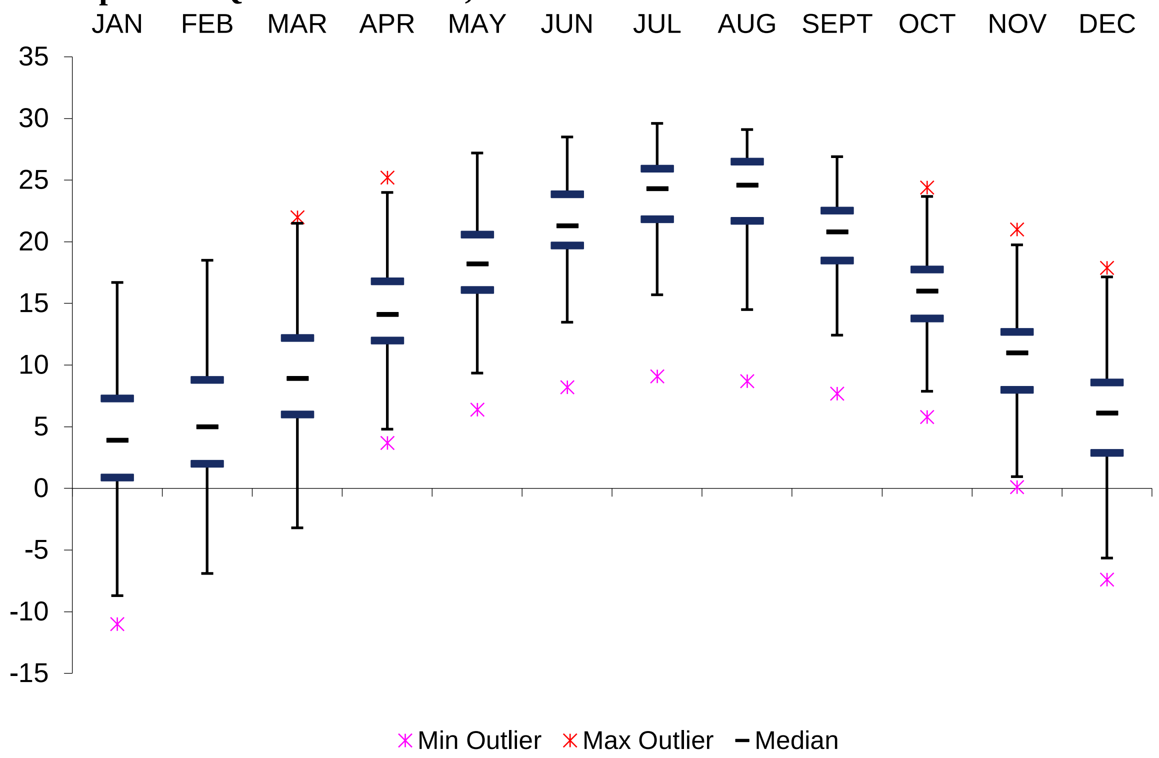


**MT$_{min}$ boxplots for *Quercus variabilis*, n = 643**

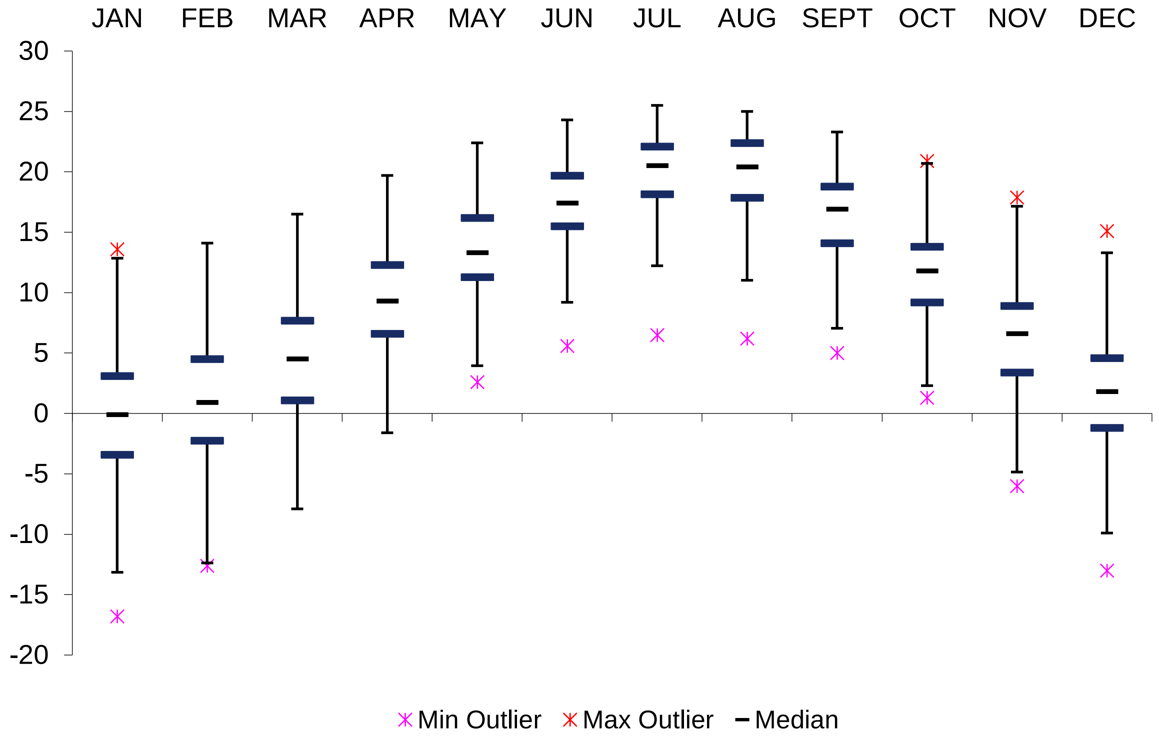

## References


**Cui D, Liang S, Wang D, Liu Z. 2021.** A 1 km global dataset of historical (1979–2013) and future (2020–2100) Köppen–Geiger climate classification and bioclimatic variables. *Earth System Science Data* 13: 5087–5114.

**Denk T, Grimm GW, Grímsson F, Zetter R. 2013.** Evidence from "Köppen signatures" of fossil plant assemblages for effective heat transport of Gulf Stream to subarctic North Atlantic during Miocene cooling. *Biogeosciences* **10**: 7927–7942.

**Grimm GW, Denk T. 2012.** Reliability and resolution of the coexistence approach — A revalidation using modern-day data. *Review of Palaeobotany and Palynology* **172**: 33–47.

**Grímsson F, Grimm GW, Meller B, Bouchal JM, Zetter R. 2016.** Combined LM and SEM study of the Middle Miocene (Sarmatian) palynoflora from the Lavanttal Basin, Austria: Part IV. Magnoliophyta 2 – Fagales to Rosales. *Grana* **55**: 101–163.

**Huang C, Zhang Y, Bartholomew B. 1999.** *Quercus*. In: Wu Z-Y, and Raven PH, eds. *Flora of China 4 Cycadaceae through Fagaceae*. Beijing, St. Louis: Science Press and Missouri Botanical Garden Press, 370–380. [accessed online at e-Floras: http://www.efloras.org/florataxon.aspx?flora_id=2&taxon_id=127839]

**Jiang X-L, Hipp AL, Deng M, Tao Su T, Zhou Z-K, Yan M-Y. 2019**. East Asian origins of European holly oaks (*Quercus* section *Ilex* Loudon) via the Tibet-Himalaya. *Journal of Biogeography* 46: 2203–2214.

**Kottek M, Grieser J, Beck C, Rudolf B, Rubel F. 2006**. World map of the Köppen-Geiger climate classification updated. *Meteorologische Zeitschrift* 15: 259–263.

**Olson DM, Dinerstein E, Wikramanayake ED, Burgess ND, Powell GVN, Underwood EC, D'Amico JA, Itoua I, Strand HE, Morrison JC, Loucks CL, Allnutt TF, Ricketts TH, Kura Y, Lamoreux JF, Wettengel WW, Hedao P, Kassem KR. 2001.** Terrestrial ecosystems of the world: A new map of life on Earth. *BioScience* 51: 933-938.

**Peel MC, Finlayson BL, McMahon TA. 2007**. Updated world map of the Köppen-Geiger climate classification. *Hydrology and Earth System Sciences* 11: 1633–1644, https://doi.org/10.5194/hess-11-1633-2007.

**Rubel F, Brugger K, Haslinger K, Auer I. 2017**. The climate of the European Alps: Shift of very high resolution Köppen- Geiger climate zones 1800–2100. *Meteorologische Zeitschrift* 26: 115–125, https://doi.org/10.1127/metz/2016/0816.

**Schroeder G-F. 1998**. *Lehrbuch der Pflanzengeographie.* Wiesbaden: Quelle & Meyer.

**Tabulated data**
All raw data used for the maps and plots are provided as XLSX-formatted spread-sheet files:

**Tabulated Data S3-1** includes coordinates (grid-cells), climate (Köppen-Geiger) and biome classification for GBIF occurrence data.

**Tabulated Data S3-2** gives the proportional composition per species with respect to climate (‘Köppen profiles’) and biome coverage.

**Tabulated Date S3-3** lists the (bio-)climate data for all data points (sheet *Bioclimate variables*) and the data extracted for the MTCM vs. PCQ x-y plots (sheet *XY plots*)

## References for GBIF distribution datasets

*Quercus* sect. *Cerris* datasets

*Quercus acutissima* GBIF dataset: https://doi.org/10.15468/dl.mfxgs9

*Quercus afares* GBIF dataset: https://doi.org/10.15468/dl.xkk4td

*Quercus branti* GBIF dataset: https://doi.org/10.15468/dl.ng2vna

*Quercus castaneifolia* GBIF dataset: https://doi.org/10.15468/dl.fexw7u

*Quercus cerris* GBIF dataset: https://doi.org/10.15468/dl.fe3qft

*Quercus chenii* GBIF dataset: https://doi.org/10.15468/dl.xdahkj

*Quercus ithaburense* GBIF dataset: https://doi.org/10.15468/dl.vg6jgs

*Quercus libani* GBIF dataset: https://doi.org/10.15468/dl.yat27m

*Quercus suber* GBIF dataset: https://doi.org/10.15468/dl.7hvbr5

*Quercus trojana* GBIF dataset: https://doi.org/10.15468/dl.cug7aa

*Quercus variabilis* GBIF dataset: https://doi.org/10.15468/dl.3sxva6
